# Gene Expression as a Dosimeter in Irradiated *Drosophila melanogaster*

**DOI:** 10.1089/cmb.2017.0170

**Published:** 2017-12-01

**Authors:** Samana Shrestha, Adam Vanasse, Leon N Cooper, Michael P. Antosh

**Affiliations:** ^1^ Department of Physics, University of Rhode Island, Kingston, Rhode Island.; ^2^ Department of Physics, Brown University, Providence, Rhode Island.; ^3^ Institute for Brain and Neural Systems, Brown University, Providence, Rhode Island.

**Keywords:** gene expression, radiation biology, radiation dosimetry

## Abstract

**Biological indicators would be of use in radiation dosimetry in situations where an exposed person is not wearing a dosimeter, or when physical dosimeters are insufficient to estimate the risk caused by the radiation exposure. In this work, we investigate the use of gene expression as a dosimeter. Gene expression analysis was done on 15,222 genes of *Drosophila melanogaster* (fruit flies) at days 2, 10, and 20 postirradiation, with X-ray exposures of 10, 1000, 5000, 10,000, and 20,000 roentgens. Several genes were identified, which could serve as a biodosimeter in an irradiated *D. melanogaster* model. Many of these genes have human homologues. Six genes showed a linear response (R^2^ > 0.9) with dose at all time points. One of these genes, inverted repeat-binding protein, is a known DNA repair gene and has a human homologue (*XRCC6*). The lowest dose, 10 roentgen, is very low for fruit flies. If the lowest dose is excluded, 13 genes showed a linear response with dose at all time points. This includes 5 of 6 genes that were linear with all radiation doses included. Of these 13 genes, 4 have human homologues and 8 have known functions. The expression of this panel of genes, particularly those with human homologues, could potentially be used as the biological indicator of radiation exposure in dosimetry applications.**

## 1. Introduction

In the occurrence of a large-scale nuclear event, such as those at Hiroshima, Nagasaki, Chernobyl, and Fukushima Daiichi, the measurement of radiation dose in exposed humans can be of crucial importance to survival (Chaudhry, 2008; Hall and Giaccia, 2012). However, in this situation, it is very likely that many people who are exposed will not be wearing dosimeters. Thus, a method of estimating radiation dose to a patient without a dosimeter would be a very useful procedure.

One possible methodology for this procedure is the use of gene expression [polymerase chain reaction (PCR), gene sequencing, microarray analysis, and other methods]. The hypothesis is that the expression of genes will change due to the absorbed radiation, and that this change can aid or even substitute for physical dosimeters and act as a biomarker to estimate the distributed dose or the overall exposure. It also helps then to predict the long-term risks of both acute and chronic exposure (Amundson et al., 2001, 2003; Omaruddin et al., 2013; Tucker et al., 2013).

In addition to not requiring equipment, such as a dosimeter, another potential advantage of a gene expression dosimeter is the time scale over which the measurements can be made. Even after the radiation exposure has taken place, the biological indicators for biodosimetry can still be determined. This would certainly be an advantage compared to the physical dosimetry (Streffer, 1996). Some biodosimetric techniques could be used long times after exposure (from 6 months to >50 years), making them unique compared to the requirements for methods used for immediate dose estimation (Simon et al., 2007).

Biological dosimetry not only provides information about the range of radiation dose but also along with this provides information about the individual radio sensitivity, which depends on age, smoking habits, or other environmental toxins. Thus, biological indicators are also a measure of the biological, medical radiation damage. Hence, we can predict about the possible radiation damage by the determination of biological indicators (Müller and Streffer, 1991; Streffer, 1996; Filiano et al., 2011; Tucker et al., 2013).

The possibility of using gene expression changes has been an exciting method to measure and predict the damage due to ionizing radiation. The exposure of cells or animals to ionizing radiation may cause DNA damage and trigger the highly complex molecular response, resulting in changes of gene expression. These molecular responses may provide the prospective indicator of exposure (Amundson et al., 2001; Chaudhry, 2008). Previous work in this area showed that the variation in the response of genes is due to dose, dose rate, radiation quality, and time after radiation exposure. This suggests that gene expression analysis may be an informative marker of radiation exposure and hence can be used as a potential biomarker. It is important to understand the cellular response to ionizing radiation or biological effects of radiation exposure to develop the predictive markers for the risk assessment due to radiation exposure on humans (Chaudhry, 2008). The rigorous research going on in genomics and bioinformatics enables the development of gene expression profiling as a useful biological indicator of radiation exposure (Amundson and Fornace, 2001; Filiano et al., 2011).

Work on this area until now has shown that the fold change in gene expression in response to radiation must be measured directly to develop a gene expression biomonitor. The expression of the genes would then be a suitable biomarker of radiation exposure (Omaruddin et al., 2013). The biodosimetry platform obtained by the experiment could also be used for personalized monitoring of radiotherapy treatments received by patients (Brengues et al., 2010).

Several studies have been done to identify the potential biomarkers of radiation exposure. Tucker et al. (2013) used reverse transcription real-time PCR to quantify the expression of selected 106 genes as a function of time up to 7 days postexposure and concluded that the gene expression analysis by qPCR shows a promising method for radiation biodosimetry. In their experiment, the mice were exposed to C0–60 γ rays source at doses from 0 to 10 Gy. The result showed that only 4–7 different genes explained the variance (R^2^) ≥0.69, whereas for the receiver operator characteristics (a measure of sensitivity and specificity) were ≥0.93 at each time point. At radiation doses up to 6 Gy, the dosimetry was very accurate. Above 6 Gy, the gene expression dosimetry had limitation. Similar analysis in humans could be done to assess exposures in mass casualty situations (Tucker et al., 2013).

Gene expression analysis in response to radiation was done in human lymphocytes and peripheral blood leukocytes using three different techniques: microarray, multiplex quantitative real-time PCR (MQRT-PCR), and nCounter Analysis System. A set of genes was found to be suitable for biological dosimetry using peripheral blood. Four of the genes (*CDKN1A*, *GADD45A*, *PHPT1*, and *CCNG1*) show good agreement among the three methods, and the upregulation of expression in blood and lymphocytes was detected by all the three techniques. These biomarkers could potentially be used for monitoring radiation exposure during radiotherapy and radiological incidents (Kabacik et al., 2011).

A novel study was done using blood from patients receiving targeted radiotherapy (^131^I-mIBG) to characterize biomarkers that may be useful for biodosimetry. As an alternative biodosimetry approach, real-time PCR analysis was done for the gene expression and the data showed that transcripts, which have already been proven as biomarkers of external exposures in radiotherapy patients, are also good early indicators of internal exposure. Three transcripts showed that modulation in gene expression was still significant enough to differentiate between exposed and unexposed samples after 96 hours of radiopharmaceutical treatment. A biodosimetry model for gene expression was developed to predict absorbed dose based on modulation of gene transcripts within whole blood. Thus, this biodosimetry for internal radiation dose or the panel of responsive genes obtained from this study could be used for establishing triage in affected areas due to dirty bombs or nuclear reactor accidents at least by rapidly sorting out the ^131^I-exposed from unexposed individuals. Thus, these selected genes could be strong biomarkers of both external and internal exposures to humans (Edmondson et al., 2016).

A comprehensive analysis of bone marrow endothelial cell (BMEC) gene expression over time in wild-type mice after total body irradiation of 5 Gy was done with a particular focus on the secreted gene products. This study is done to characterize the molecular response of BMECs to ionizing radiation to identify the cellular mechanisms and paracrine factors through which BMECs regulate hematopoietic regeneration. The result of a microarray experiment showed that the gene expression of BMECs is altered within 24 hours after total body irradiation of 5 Gy and by 14 days this molecular response is resolved. Also, a number of genes that encode secreted proteins are strongly upregulated (Inhbb, Ccl2, Ptn) and are downregulated (Chl1, Galnt10, Ryk, Pon2, Sdha) more than 10-fold in ECs in response to radiation after 6 hours (Himburg et al., 2016).

Amundson et al. (1999) showed the dose/response relationship for the induction of five genes (CDKN1A, GADD45, MDM2, ATF3, and BAX) exposed to γ rays between the doses of 2–50 cGy. As a follow-up, Amundson et al. (2003) studied the dose/response relationships by reducing the dose rate over three orders of magnitude and found some protection against the induction of apoptosis. They studied the response of 10 cGy and less exposure of γ rays in the ML-1 human cell line and showed that the gene expression could be triggered by the low doses. At different dose responses between 2 and 50 cGy, a linear increase in expression of three genes CDKN1A, GADD45A, and MDM2 was observed in the cell line ML-1, whereas dose rate effect was observed only for GADD45A and CDKN1A. The data obtained from the microarray analysis on RNA samples 2 hours postirradiation with low-dose Y-rays indicate that some genes show a dose rate effect while others do not. This indicates the potential usefulness of gene expression as a biomarker for radiation exposure (Amundson et al., 2003).

Stassen et al. (2003) examined 1176 genes expressed by MCF-7 human mammary carcinoma cells exposed to 2 and 6 Gy of X-rays and found that six of them were radiation-induced gene targets over 1 (3 genes), 2 (2 genes), and 3 (1 gene) days, which was confirmed by quantitative reverse transcription PCR (RTQ-PCR). Of those six (GLUT-1, PCKI, WAF-1, ISGF3G, MRP8, PSME3), the last three were novel gene targets showing a correlation between radiation dose and clonogenicity, which suggested an individual dose dependency for all selected genes.

Omaruddin et al. (2013) examined the gene expression of the genes MADH7, SEC PRO, and CC3 using relative quantitative RT-PCR in blood samples of patients before and after undergoing radiation therapy. This gave a wide range of values stating the complexity of the response. SEC PRO was found to be downregulated, while the gene MADH7 was found to be upregulated in most of the patients. Therefore, the gene MADH7 could be used as a molecular marker for radiation exposure.

Filiano et al. (2011) performed gene expression analysis using real-time quantitative PCR in blood samples from cancer patients undergoing total body irradiation. A set of eight biodosimetry genes (ACTA2, BBC3, CCNG1, CDKN1A, GADD45A, MDK, SERPINE1, and TNFRSF10B) was identified. In addition, gene expression analysis was done in C57BL/6 mice at doses 0–8 Gy and times 5, 12, 23, and 48 hours after irradiation. The results showed a significant increase in the expression of five of the above genes (BBC3, CCNG1, CDKN1A, SERPINE1, and TNFRSF10B).

This article focuses on gene expression analysis of *Drosophila melanogaster* (fruit flies). Compared to humans, biodosimetry information can be obtained in a more controlled manner in animal models because the dose received in humans is usually not known, the exposures may be nonuniform, and the dose rates may not be known. Data collection may not be reliable and uniform postirradiation because a lot of variables have to be taken into consideration such as age, health, sex, genotype, time since exposure to radiation, personal lifestyle such as cigarette smoking, tobacco, and alcohol habits (Tucker, 2008; Tucker et al., 2013). *D. melanogaster* is a model organism with a useful life span (∼2 months) and a long history in radiation experiments. Its genome has been sequenced, and many genes in *Drosophila* are homologous with human genes (2016; Gramates et al., 2017).

This article makes use of a previous gene expression analysis done by Antosh et al. (2014). The experiment was performed to discover the biological effects at different levels of ionizing radiation in *D. melanogaster*. The results showed a threshold effect in response to the radiation, both in gene expression and in survival. The gene expression results suggest stress, metabolism, reproduction, and mitochondrial function as mechanisms involved in the radiation response (Antosh et al., 2014). The data were taken for five radiation doses (plus a control), at 3 time points. The setup of these data allows them to be repurposed for a new analysis that examines the response of genes as a function of radiation dose.

The aim of this study is to secure a set of genes that are responsive to radiation in a predictable way. These genes, particularly if homologous to human genes, have potential uses in radiation dosimetry.

## 2. Methodology

The data used in this article are obtained from data submitted to the gene expression omnibus by Antosh et al. (2014; posted under the reference number GSE47999). Normalized data were calculated using the DESeq (Anders and Huber, 2010) package in Bioconductor (Gentleman et al., 2004).

The data were obtained from an RNA-sequencing gene expression experiment on *D. melanogaster*, at ages 2, 10, and 20 days after irradiating them. Flies were irradiated with X-ray exposures of 0, 10, 1000, 5000, 10,000, and 20,000 roentgen (a 1 roentgen radiation exposure is ≈0.01 gray; here we will use the terms “exposure” and “dose” equivalently). The irradiation came inside a chamber containing cesium-137. Samples were taken at 2, 10, and 20 days after irradiation, with 3 samples per experimental condition (except for sample for 0R, day 20, where one sample failed quality check). Our reanalysis of these data was done to identify the genes that changed in a predictable way from control, as a function of dose. Genes that behave in a predictable way could potentially be used in a future biodosimeter.

The fold changes in the expression of genes depending on dose and time after exposure were measured in the fruit fly model. To calculate the fold change, average value of the gene expression of the samples at each time point and radiation dose was divided by the average value of gene expression of control at the same time point (control being zero added radiation). Fold changes were ignored (in a present/absent cutoff) if the average expression in both experimental and control samples was less than a bottom quartile cutoff (∼18–20 counts).

One analysis performed was based on linear regression. For each time point, the R^2^ value for a linear fit (fold change vs. radiation dose) was calculated for each gene. Genes with R^2^ > 0.9 were selected as behaving linearly. In a secondary analysis, the data for 10 roentgen flies were removed (since this is a very small dose of radiation for fruit flies). The linear analysis described above was run again. In both of these analyses, genes were only selected as linear if at least four radiation doses passed the present/absent cutoff (described in the paragraph immediately before this).

As an additional analysis, gene expression data were examined for “spikes” in fold change. For each gene, at each time point, a set of fold changes was examined (one fold change for each radiation dose). Genes were marked as having a spike if the largest fold change was at least five times greater than the second largest fold change. In addition, genes were only counted as having a spike if the fold change of the spike was >1 (meaning that the average expression at the spike dose was greater than average gene expression in the corresponding control).

For each time point, and for overlaps between time points, genes found to be significant (meaning, linear or spiking) were analyzed as a group using GOStat (Beißbarth and Speed, 2004) to see if any biological functions had a statistically significant amount of genes in the group. Gene ontologies with a corrected *p* value <0.05 were selected.

Genes were examined for human homologues using HomoloGene (2016) and functional information was found using FlyBase (Gramates et al., 2017).

## 3. Results

### 3.1. Analysis of linear behavior with full data set

**FIG. 1.**
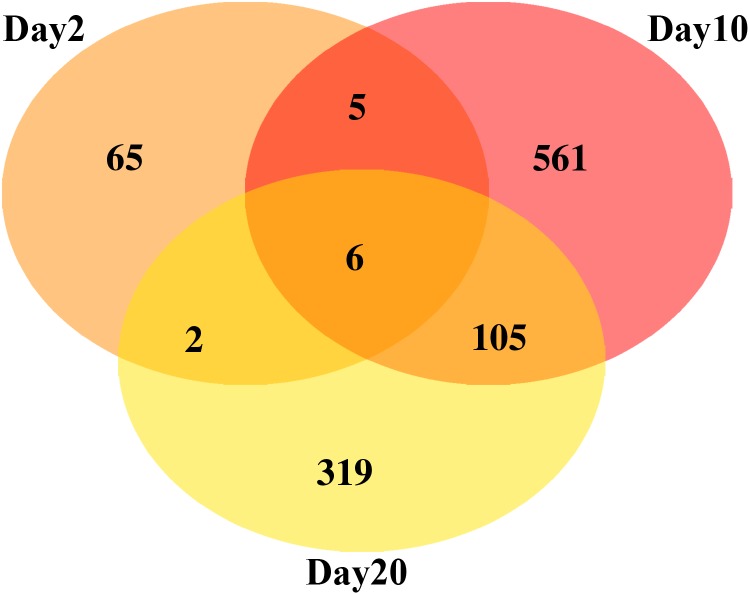
Number of genes with linear response at each time point, with overlaps.

Figure 1 shows the number of genes with a linear response in fold change as a function of radiation dose, at each of the three time points (2, 10, and 20 days postirradiation). Seventy-eight genes showed a linear response at day 2 after irradiation; 677 genes showed a linear response at day 10 after irradiation; 432 genes showed linear response at day 20 after irradiation. A full list of genes for each time point and each overlap is given in Supplementary Table S1A–G. A set of six genes (*FBgn0011774*, *FBgn0030189*, *FBgn0031713*, *FBgn0032393*, *FBgn0037020*, and *FBgn0051864*) was found to have a linear response in all time points. Table 1 shows the set of those six genes, including homology to human genes (2016) and functional information (Gramates et al., 2017). Four of these six genes have homologues in humans. Genes found to behave linearly across a fairly wide range of times are perhaps most promising for a possible radiation dosimeter. The median life span of control flies in this experiment was ∼50 days; our time range here is 18 days.

**Table 1.** Name, Homology, and Functional Information on Six Genes Found to Respond Linearly to Radiation at All Time Points Examined

*FlyBase ID*	*Chromosome (Gramates et al. 2017)*	*Gene symbol (from FlyBase) (Gramates et al., 2017)*	*Human gene homologue (from HomoloGene) (2016)*	*Biological function (Gramates et al., 2017)*	*Molecular function (Gramates et al., 2017)*
*FBgn0011774*	3R	*Irbp*	*XRCC6*	Double-strand break repair via nonhomologous end joining, telomere maintenance	Contributes to DNA binding, protein heterodimerization activity, ATP-dependent DNA helicase activity, Damaged DNA binding, telomeric DNA binding inferred
*FBgn0030189*	X	*CG2909*	None	Not known	Not known
*FBgn0031713*	2L	*CG7277*	*COQ6*	Oxidation/reduction process, ubiquinone biosynthetic process	FAD binding, oxidoreductase activity, acting on paired donors, with incorporation or reduction of molecular oxygen, NAD(P)H as one donor, and incorporation of one atom of oxygen
*FBgn0032393*	2L	*CG12264*	*NFS1*	Alanine biosynthetic process, iron/sulfur cluster assembly, [2Fe-2S] cluster assembly	Cystathionine gamma-lyase activity, cysteine desulfurase activity, pyridoxal phosphate binding
*FBgn0037020*	3L	*Pex14*	*PEX14*	Peroxisome organization, protein import into peroxisome matrix, docking, protein targeting to peroxisome	Receptor binding
*FBgn0051864*	2L	*Qtzl*	None	Not known	Not known

ATP, adenosine triphosphate; FAD, flavin-adenine dinucleotide; Irbp, inverted repeat-binding protein.

A GOstat analysis (Beißbarth and Speed, 2004) was run on genes found to be linear at each time point and also separately on overlaps between time points. Full lists of significant gene ontologies from the analysis can be found in Supplementary Table S2A–G. Several of the results suggest that the genes that behave linearly are involved in stress responses. At 2 days postirradiation, 78 genes show linear behavior with dose. These 78 genes contain 13 of the 23 genes related to protein kinase CK2 regulator activity (a *p* value of 2.2 × 10^−24^). The protein kinase CK2 inhibits apoptosis following ionizing radiation (Yamane and Kinsella, 2005). Gene ontologies for spermatogenesis and reproduction are also affected 2 days postirradiation. Genes found to be linear at 10 days postirradiation were statistically overrepresentative of gene ontologies for oxidoreductase activity (a possible response to radiation damage) and growth factor activity. At 20 days postirradiation, overrepresented gene ontologies included stress-related pathways such as response to stress, receptor activity, signal transducer activity, detection of bacterium and biotic stimulus, and response to DNA damage stimulus. In the genes found in the overlap of days 2 and 10, overrepresented gene ontologies included peroxisomal transport and nicotinamide adenine dinucleotide phosphate (NADPH) activity. In the genes found in the overlap of days 2 and 20, overrepresented gene ontologies included several pathways related to the peroxisome, DNA helicase activity, response to hypoxia, and telomere maintenance. The overlap between genes in days 10 and 20 found the gene ontology for stress response to be overrepresented. Gene ontologies overrepresented in genes found to be linear at all three time points (2, 10, and 20 days) included peroxisome, DNA helicase activity, ATPase activity, and telomere maintenance.

### 3.2. Analysis of linear behavior with lowest dose not included

**FIG. 2.**
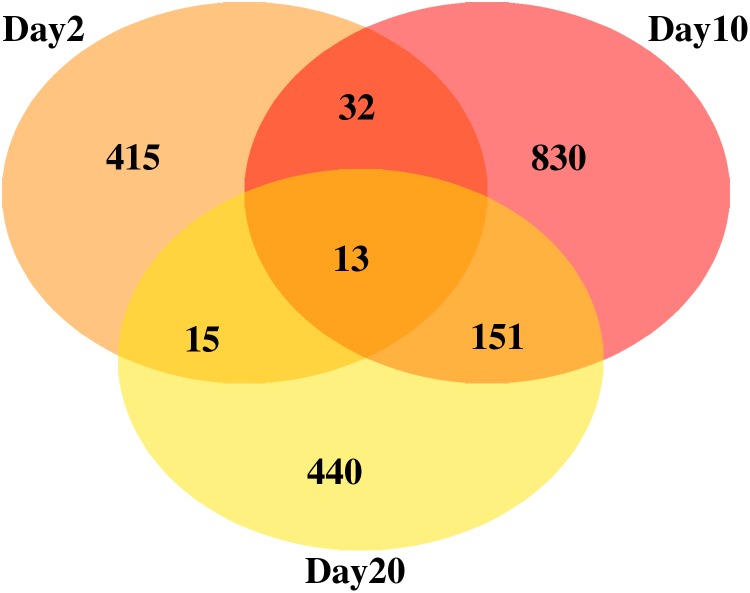
Analysis of linear behavior, not including data from dose 10 roentgen. A number of genes with linear response (R^2^ > 0.9) are given for each time point, with overlaps.

In the life span experiment that accompanied this data set (Antosh et al., 2014), life span effects on fruit flies were not seen until a radiation exposure of 10,000 roentgen (an approximate radiation dose of 100 Gy). The smallest dose in this analysis is 10 roentgen, which is 0.1% of that dose. It is possible that the 10 roentgen dose in this experiment may produce some gene expression at the level of noise. To address that possibility, a secondary analysis for linear behavior was run where the data from 10 roentgen were not included. The results are summarized in Figure 2 and Supplementary Table S3A–G. In this analysis, 13 genes are found to be linear at all three time points. This list includes 5 of the 6 genes found to behave linearly at all three data points when the 10 roentgen data were included in the analysis (Table 1). The sixth gene, *FBgn0031713*, was excluded only because R^2^ = 0.88 at day 20. The 13 genes in the overlap are described in Table 2. Of these 13 genes, 4 have human homologues.

**Table 2.** Genes Found to Behave Linearly at All Three Time Points, if the Lowest Radiation Dose (10 Roentgen) Is Not Included

*FlyBase ID*	*Chromosome (Gramates et al., 2017)*	*Gene symbol (Gramates et al., 2017)*	*Human gene homologue (2016)*	*Biological function (Gramates et al., 2017)*	*Molecular function (Gramates et al., 2017)*
*FBgn0011774*	3R	*Irbp*	*XRCC6*	Double-strand break repair via nonhomologous end joining, telomere maintenance	DNA binding, protein heterodimerization activity, ATP-dependent DNA helicase activity, damaged DNA binding, telomeric DNA binding inferred
*FBgn0024912*	3R	*agt*	None	DNA dealkylation involved in DNA repair	Methylated-DNA-[protein]-cysteine S-methyltransferase activity
*FBgn0027101*	4	*Dyrk3*	*DYRK2*	Protein phosphorylation	ATP binding, protein kinase activity
*FBgn0030189*	X	*CG2909*	None	Not known	Not known
*FBgn0032393*	2L	*CG12264*	*NFS1*	Alanine biosynthetic process, iron/sulfur cluster assembly, [2Fe-2S] cluster assembly	Cystathionine gamma-lyase activity, cysteine desulfurase activity, pyridoxal phosphate binding
*FBgn0033926*	2R	*Arc1*	None	Behavioral response to starvation, muscle system process	Nucleic acid binding, zinc ion binding
*FBgn0033927*	2R	*CR10102*	None	Not known	Not known
*FBgn0034184*	2R	*CG9646*	None	Not known	Not known
*FBgn0036290*	3L	*CG10638*	None	Oxidation/reduction process inferred	Oxidoreductase activity, inferred
*FBgn0037020*	3L	*Pex14*	*PEX14*	Peroxisome organization, protein import into peroxisome matrix, docking, protein targeting to peroxisome	Peroxisome importomer complex, peroxisomal membrane, peroxisome
*FBgn0037850*	3R	*CG14695*	None	Not known	Not known
*FBgn0046763*	3R	*CG17278*	None	Negative regulation of Wnt signaling pathway inferred from genetic interaction	Not known
*FBgn0051864*	2L	*Qtzl*	None	Not known	Not known

Name, homology, and functional information on 13 genes found to respond linearly to radiation at all time points examined.

A GOstat analysis (Beißbarth and Speed, 2004) was run on genes found to be linear at each time point and also separately on overlaps between time points. Full lists of significant gene ontologies from the analysis can be found in Supplementary Table S4A–G. As with the analysis with all radiation doses, several of the results suggest that the genes that behave linearly are involved in stress responses.

At 2 days postirradiation, overrepresented gene ontologies include protein kinase CK2 regulator activity and spermatogenesis. Genes found to be linear at 10 days postirradiation were statistically overrepresentative of gene ontologies for oxidoreductase activity (a possible response to radiation damage), growth factor activity, GTPase activity, hydrolase activity, electron carrier activity, and pathways related to peroxisomes. At 20 days postirradiation, overrepresented gene ontologies included detection of biotic stimulus and bacterium and metabolism of toxins, xenobiotics, insecticides, and water-soluble vitamins. In the genes found in the overlap of days 2 and 20, overrepresented gene ontologies included peroxisomal transport, NADPH regeneration, telomere maintenance, DNA helicase activity, transferase activity, and ATPase activity. The overlap between genes in days 10 and 20 found several gene ontologies related to peroxisomes to be overrepresented. Gene ontologies overrepresented in genes found to be linear at all three time points (2, 10, and 20 days) included pathways related to peroxisomes, telomere maintenance, and Wnt signaling. No gene ontologies were significantly overrepresented in the overlap between genes linear at days 2 and 10 postirradiation.

### 3.3. Analysis of genes for spikes in expression

**FIG. 3.**
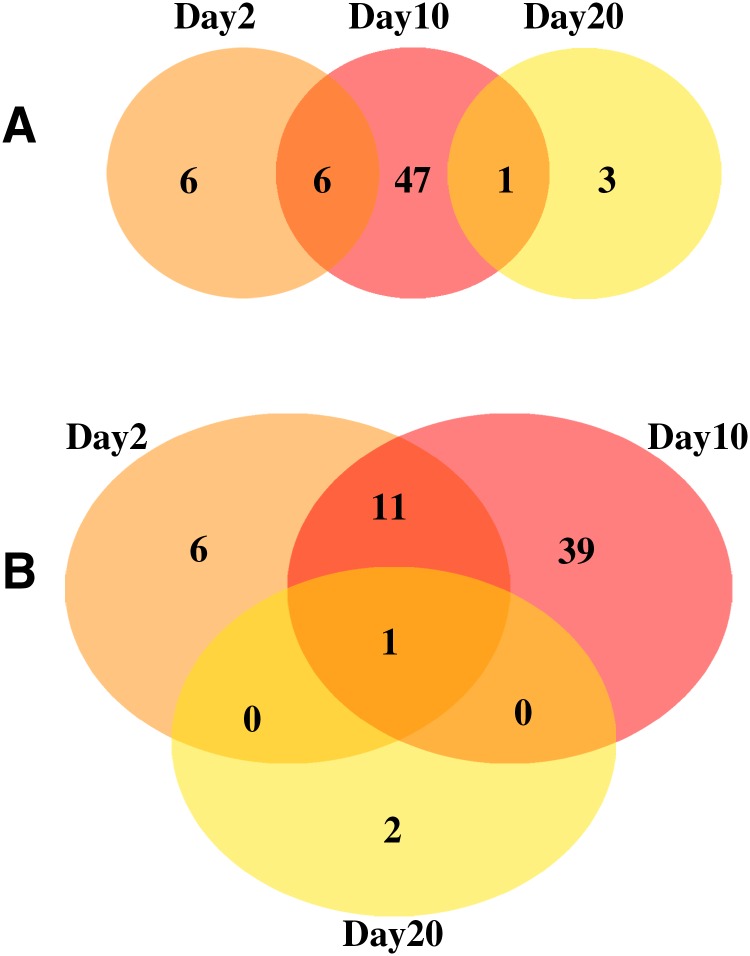
Number of “spike” genes at each time point, with **(3A)** and without **(3B)** the lowest radiation dose.

In addition to linear behavior, another potential methodology for using gene expression as a dosimeter would involve genes that “spike”; meaning that a given gene sees a large amount of expression (compared to control flies) at a given radiation dose. To search for such an effect in this data set, we looked for genes where the fold change was at least five times higher at one radiation dose than at any other radiation dose examined. The results are shown in Figure 3A, and in Supplementary Tables S5A–G and S6A–G. Zero genes were found in the overlap between all three time points, which suggests that there may be no good candidate genes for a biological dosimeter.

Similar to linear analysis, we performed the analysis a second time with the data for 10 roentgen radiation exposure removed. Results are shown in Figure 3B, and Supplementary Table S4H–N. In this analysis, one gene was found to be changing at all three time points. This gene, *FBgn0085364*, has no human homologue and no listed functions in FlyBase (in FlyBase, a search for this gene is directed toward *FBgn0267910*).

For the spike analysis, GOstat analyses were run with the genes being separated by the radiation dose where a spike is found. Any spikes at the largest radiation dose were not considered, because it is likely that a highest expression value at the highest radiation dose is indicative of a gene that is merely increasing with dose (not necessarily in a linear manner). The radiation doses at which each gene spikes are listed in Supplementary Tables S5A–G and S6A–G. Overall, the GOstat results on spiking genes showed an effect on reproduction and some effect on stress responses.

The analysis with all radiation doses included the following:

• For genes with spikes at day 2 postirradiation at radiation dose 5000 roentgen, overrepresented gene ontologies are all due to *FBgn0013745* and are related to reproduction and behavior.
• For genes with spikes at day 2 postirradiation at radiation dose 1000 roentgen, the only two genes are *yolk protein 1* and *yolk protein 2* (note: in this section, all full gene names were found in FlyBase). Overrepresented gene ontologies include vitellogenesis, reproductive development, and chromatin remodeling.
• For genes with spikes at day 10 postirradiation, only five genes are not from the highest radiation dose. These five genes are all from dose 10 roentgen. Overrepresented gene ontologies include those related to chorion (from *chorion protein 15* and *chorion protein 18*) and sensing of chemical stimulus (from *odorant binding protein 19c*).
• For genes with spikes at day 20 postirradiation, only one gene is not from the highest radiation dose. This gene, FBgn53222, spiked at dose 5000 roentgen and gave overrepresented gene ontologies related to ribosomes.
The analysis with the lowest radiation dose (10 roentgen) did not include the following:
• For genes with spikes at day 2 postirradiation, only four genes spiked at doses less than the maximum dose. Two genes spiked at dose 5000 roentgen; all overrepresented pathways in GOstat were due to *FBgn0013745* (similar to the analysis including 10 roentgen). Two genes spiked at dose 10,000 roentgen—yolk protein 1 (as in the analysis including 10 roentgen) and *FBgn0013675*, which resulted in overrepresented gene ontologies related to oxidative response.
• For genes with spikes at day 10 postirradiation, seven genes spiked at dose 10,000 roentgen. Six of these seven genes were related to reproduction and include *yolk proteins 1, 2, and 3*.
• For genes with spikes at day 20 postirradiation, one gene (*FBgn0053222*) spiked at dose 5000 roentgen (the same gene as the analysis including 10 roentgen).

GOstat results related to the results reported above can be found in Supplementary Table S7.

## 4. Discussion

A radiation dosimeter based on gene expression could result in better diagnosis of radiation dose in patients and thus may help in saving lives after a nuclear event or accidental radiation exposure. The results of this article indicate several candidate genes that have potential to be used for that purpose. In particular, it seems that the best candidates may be the genes listed in Tables 1 and 2 that have human homologues.

One particularly interesting candidate gene is *Irbp* (inverted repeat-binding protein), which was found to behave linearly in all three data points, both with the full data set and with the lowest dose removed. *Irbp* is related to DNA repair. It is reasonable to predict that DNA damage is linear with radiation dose; thus, it is logical that some DNA repair genes may respond linearly in expression. *Irbp* has homologues in organisms that are as complex as humans and chimpanzees, and also in organisms such as Japanese rice (2016).

Another possibility, based on the application of GOstat results, is to look at particular cellular functions. In particular, the function of protein kinase CK2 may be useful at time points soon after radiation exposure. Protein kinase CK2 was overrepresented in the GOstat analysis for genes found to behave linearly 2 days after irradiation, with a very high statistical significance. Perhaps the functionality of this protein kinase could be measured directly as a function of radiation to produce a different type of radiation dosimeter.

Several genes listed in Tables 1 and 2 had no known functions in FlyBase (Gramates et al., 2017). These results suggest that they are related to radiation responses and possibly to stress responses.

From a dose/response standpoint, one interesting characteristic of the linear analysis results is that some genes with a linear response in fold change have fold changes that are <1 (radiation expression less than control expression) at lower doses, but then transition to fold changes >1 (radiation expression greater than control expression) at high doses. For example, in the linear analysis with all radiation doses, the genes *FBgn0011774*, *FBgn0030189*, *FBgn0037020*, and *FBgn0051864* are linear at all three time points and exhibit this behavior at day 2 postirradiation. Descriptions of these genes can be found in Table 1. This could be representative of some biological effects being in one direction at lower doses of radiation, and in the opposite direction at higher doses of radiation. Fold changes and R^2^ values are given in Supplementary Table S5A–F.

Future questions related to this research could include the following:

• How well do results in *Drosophila* genes with human homologues translate to results in humans?
• Do the genes in Tables 1 and 2 continue to respond linearly at more times postirradiation, including times <2 days?
• How are these results affected by the energy and type of irradiation?

Further development of this methodology is needed before it can be applied to patients, but these results suggest the possibility of a successful gene expression radiation dosimeter.

## Supplementary Material

Supplemental data
