# Supplementary material for: Gene Expression as a Dosimeter in Irradiated Drosophila melanogaster
Source: J Comput Biol. 2017 Dec 1;24(12):1265–74. doi: 10.1089/cmb.2017.0170 (PMC5729855; doi:10.1089/cmb.2017.0170)
Supplement: Supplemental data [file Supp_Data.docx]

| Supplemental Table 1A. Values of R Squared and Fold Changes for Genes Found to Behave Linearly at Day 2 Post-Irradiation. (Analysis with all data included.) | | | | | |  |
| --- | --- | --- | --- | --- | --- | --- |
|  |  |  |  |  |  |  |
| Flybase ID | R^2 Value Day 2 | 10 R Fold Change | 1000 R Fold Change | 5000 R Fold Change | 10000 R Fold Change | 20000 R Fold Change |
| FBgn0053247 | 0.995 | 0.418 | 0.481 | 0.696 | 1.008 | 1.754 |
| FBgn0038358 | 0.995 | 1.956 | 2.055 | 2.226 | NA | 3.360 |
| FBgn0053237 | 0.994 | 0.410 | 0.454 | 0.697 | 0.941 | 1.669 |
| FBgn0053140 | 0.993 | 1.053 | 1.052 | 0.953 | 0.876 | 0.633 |
| FBgn0053243 | 0.992 | 0.405 | 0.459 | 0.701 | 0.925 | 1.659 |
| FBgn0259817 | 0.990 | 0.711 | 0.713 | 0.809 | 0.952 | 1.293 |
| FBgn0053238 | 0.989 | 0.396 | 0.450 | 0.693 | 0.905 | 1.665 |
| FBgn0053240 | 0.988 | 0.380 | 0.456 | 0.777 | 0.930 | 1.584 |
| FBgn0037020 | 0.988 | 0.914 | 0.938 | 0.953 | 1.001 | 1.089 |
| FBgn0053244 | 0.987 | 0.395 | 0.457 | 0.798 | 0.945 | 1.589 |
| FBgn0053245 | 0.987 | 0.394 | 0.459 | 0.798 | 0.945 | 1.611 |
| FBgn0053241 | 0.987 | 0.405 | 0.442 | 0.706 | 0.881 | 1.619 |
| FBgn0053236 | 0.987 | 0.398 | 0.457 | 0.800 | 0.945 | 1.600 |
| FBgn0031713 | 0.985 | 0.986 | 1.021 | 1.110 | 1.158 | 1.324 |
| FBgn0053239 | 0.985 | 0.396 | 0.450 | 0.776 | 0.908 | 1.604 |
| FBgn0053242 | 0.985 | 0.410 | 0.472 | 0.806 | 0.936 | 1.559 |
| FBgn0053246 | 0.984 | 0.383 | 0.421 | 0.713 | 0.877 | 1.677 |
| FBgn0043578 | 0.982 | 0.587 | 0.595 | 0.574 | 0.556 | 0.510 |
| FBgn0032393 | 0.979 | 1.106 | 1.135 | 1.347 | 1.423 | 1.734 |
| FBgn0037555 | 0.977 | 1.063 | 1.099 | 1.110 | 1.166 | 1.244 |
| FBgn0051029 | 0.975 | 1.017 | 1.027 | 0.984 | 0.898 | 0.816 |
| FBgn0038208 | 0.974 | 1.105 | 1.026 | 1.006 | 0.896 | 0.726 |
| FBgn0000117 | 0.971 | 0.984 | 0.987 | 0.990 | 0.995 | 1.014 |
| FBgn0028675 | 0.971 | 0.908 | 1.051 | 1.236 | 1.315 | 1.775 |
| FBgn0044817 | 0.970 | 0.399 | 0.445 | 0.781 | 0.851 | 1.631 |
| FBgn0030234 | 0.968 | 1.056 | 1.153 | 1.162 | 1.301 | 1.512 |
| FBgn0031265 | 0.968 | 0.958 | 0.986 | 0.940 | 0.867 | 0.720 |
| FBgn0035262 | 0.966 | 1.104 | 1.041 | 1.017 | 0.969 | 0.831 |
| FBgn0029697 | 0.965 | 3.102 | NA | 2.587 | 2.502 | 1.543 |
| FBgn0031945 | 0.962 | 0.907 | 0.902 | 0.918 | 1.005 | 1.158 |
| FBgn0030026 | 0.960 | 1.065 | 1.122 | 1.183 | 1.213 | 1.467 |
| FBgn0036763 | 0.959 | 1.003 | 1.024 | 0.995 | 0.958 | 0.876 |
| FBgn0038221 | 0.959 | 0.871 | 0.922 | 1.272 | 1.799 | 3.892 |
| FBgn0030207 | 0.957 | 1.092 | 1.116 | 1.121 | 1.156 | 1.265 |
| FBgn0035707 | 0.955 | 1.114 | 1.057 | 1.051 | 0.998 | 0.836 |
| FBgn0030507 | 0.955 | 1.124 | 0.963 | 1.195 | 1.540 | 1.943 |
| FBgn0259192 | 0.953 | 0.661 | 0.677 | 0.722 | 0.817 | 0.878 |
| FBgn0033495 | 0.950 | 1.055 | 1.051 | 1.006 | 0.967 | 0.928 |
| FBgn0036962 | 0.948 | 0.999 | 0.997 | 0.924 | 0.914 | 0.833 |
| FBgn0024989 | 0.948 | 0.893 | 1.200 | 1.501 | 1.743 | 3.594 |
| FBgn0033056 | 0.945 | 1.114 | 0.997 | 0.953 | 0.860 | 0.699 |
| FBgn0030189 | 0.944 | 0.725 | 0.777 | 0.848 | 1.010 | 1.108 |
| FBgn0033149 | 0.942 | 1.113 | 1.056 | 0.966 | 0.959 | 0.791 |
| FBgn0050259 | 0.941 | 0.898 | 1.399 | 1.903 | 2.031 | 3.132 |
| FBgn0002905 | 0.939 | 1.085 | 1.063 | 0.946 | 0.840 | 0.747 |
| FBgn0035134 | 0.937 | 0.790 | 0.790 | 0.933 | 0.944 | 1.392 |
| FBgn0051864 | 0.937 | 0.773 | 1.096 | 1.138 | 1.421 | 1.832 |
| FBgn0261996 | 0.934 | 1.013 | 1.072 | 1.019 | 0.767 | 0.565 |
| FBgn0039709 | 0.933 | 1.193 | 1.065 | 1.111 | 1.825 | 2.675 |
| FBgn0004841 | 0.933 | 1.160 | 1.052 | 2.013 | 2.449 | 3.151 |
| FBgn0262029 | 0.929 | 1.007 | 0.967 | 1.074 | 1.091 | 1.376 |
| FBgn0014127 | 0.929 | 0.891 | 0.873 | 0.820 | 0.820 | 0.750 |
| FBgn0038447 | 0.927 | 1.191 | 1.299 | 1.321 | 1.800 | 2.037 |
| FBgn0003435 | 0.927 | 0.974 | 0.963 | 0.883 | 0.843 | 0.784 |
| FBgn0031832 | 0.925 | 1.056 | 1.032 | 1.076 | 1.092 | 1.222 |
| FBgn0051291 | 0.924 | 1.456 | 1.393 | 1.398 | 1.821 | 2.243 |
| FBgn0032428 | 0.923 | 0.887 | 0.847 | 0.857 | 0.732 | 0.660 |
| FBgn0039411 | 0.922 | 0.905 | 1.058 | 0.897 | 1.589 | 2.415 |
| FBgn0038199 | 0.921 | 1.179 | 0.869 | 0.923 | 4.066 | 11.157 |
| FBgn0031432 | 0.921 | 0.757 | 1.021 | 1.073 | 1.408 | 1.667 |
| FBgn0020765 | 0.919 | 0.162 | 0.627 | 1.522 | 1.329 | 3.416 |
| FBgn0050384 | 0.919 | 1.014 | 1.084 | 0.984 | 0.948 | 0.767 |
| FBgn0037921 | 0.919 | 1.128 | 1.091 | 1.095 | 0.927 | 0.844 |
| FBgn0038488 | 0.919 | 1.139 | 1.085 | 1.063 | 1.056 | 0.914 |
| FBgn0261596 | 0.917 | 0.980 | 0.950 | 0.973 | 1.035 | 1.096 |
| FBgn0040060 | 0.917 | 0.859 | 0.939 | 0.960 | 0.978 | 1.144 |
| FBgn0002563 | 0.916 | 1.144 | 1.266 | 1.272 | 1.322 | 1.617 |
| FBgn0028561 | 0.910 | 1.316 | 1.163 | 1.203 | 1.031 | 0.850 |
| FBgn0086368 | 0.909 | 0.847 | 1.007 | 1.174 | 1.135 | 1.730 |
| FBgn0028370 | 0.909 | 1.634 | 1.302 | 2.839 | 4.396 | 5.233 |
| FBgn0259147 | 0.909 | 0.245 | 0.463 | 0.438 | 0.712 | 0.925 |
| FBgn0034502 | 0.908 | 0.921 | 0.875 | 0.875 | 0.847 | 0.668 |
| FBgn0036881 | 0.908 | 0.780 | 1.074 | 1.046 | 1.350 | 1.646 |
| FBgn0003346 | 0.905 | 0.845 | 0.783 | 0.824 | 0.700 | 0.583 |
| FBgn0043005 | 0.905 | 0.672 | 0.975 | 0.945 | 1.813 | 5.466 |
| FBgn0036017 | 0.904 | 1.012 | 0.986 | 1.615 | 1.580 | 4.025 |
| FBgn0011774 | 0.902 | 0.598 | 0.954 | 1.315 | 1.428 | 1.933 |
| FBgn0023001 | 0.900 | 0.919 | 1.004 | 1.008 | 1.106 | 1.177 |

| Supplemental Table 1B. Values of R Squared and Fold Changes for Genes Found to Behave Linearly at Day 10 Post-Irradiation. (Analysis with all data included.) | | | | | | |
| --- | --- | --- | --- | --- | --- | --- |
|  |  |  |  |  |  |  |
| Flybase ID | R Squared Value | 10 R Fold Change | 1000 R Fold Change | 5000 R Fold Change | 10000 R Fold Change | 20000 R Fold Change |
| FBgn0002922 | 0.999 | 1.009 | 1.055 | 1.170 | 1.336 | 1.627 |
| FBgn0051202 | 0.997 | 0.607 | 0.643 | 0.739 | 0.922 | 1.259 |
| FBgn0037561 | 0.997 | 0.981 | 1.001 | 1.049 | 1.151 | 1.309 |
| FBgn0016041 | 0.997 | 1.091 | 1.076 | 1.007 | 0.883 | 0.651 |
| FBgn0038113 | 0.996 | 0.922 | 1.009 | 1.186 | 1.353 | 1.804 |
| FBgn0037057 | 0.996 | 1.109 | 1.090 | 1.047 | 0.992 | 0.900 |
| FBgn0037676 | 0.995 | 0.771 | 0.852 | 0.960 | 1.181 | 1.635 |
| FBgn0031713 | 0.995 | 0.991 | 1.034 | 1.183 | 1.417 | 1.739 |
| FBgn0261975 | 0.995 | 0.808 | 0.814 | 0.949 | 1.081 | 1.311 |
| FBgn0035611 | 0.994 | 0.847 | 0.982 | 1.184 | 1.413 | 1.951 |
| FBgn0036290 | 0.994 | 0.664 | 0.797 | 1.069 | 1.496 | 2.118 |
| FBgn0034748 | 0.993 | 0.853 | 0.908 | 1.104 | 1.241 | 1.612 |
| FBgn0051032 | 0.993 | 1.036 | 1.047 | 1.060 | 1.098 | 1.153 |
| FBgn0032470 | 0.993 | 1.178 | 1.184 | 1.421 | 1.889 | 2.696 |
| FBgn0032514 | 0.992 | 0.958 | 0.976 | 1.101 | 1.253 | 1.464 |
| FBgn0052280 | 0.992 | 0.810 | 0.918 | 1.070 | 1.253 | 1.615 |
| FBgn0051019 | 0.991 | 0.898 | 0.914 | 1.105 | 1.217 | 1.534 |
| FBgn0039179 | 0.991 | 0.992 | 1.011 | 1.062 | 1.174 | 1.304 |
| FBgn0003996 | 0.991 | 0.822 | 0.838 | 0.961 | 1.113 | 1.536 |
| FBgn0038893 | 0.991 | 0.757 | 0.974 | 1.447 | 1.769 | 2.943 |
| FBgn0033928 | 0.991 | 1.144 | 1.364 | 1.837 | 2.217 | 3.148 |
| FBgn0031376 | 0.990 | 1.068 | 1.051 | 1.025 | 0.939 | 0.828 |
| FBgn0023000 | 0.989 | 0.826 | 0.910 | 1.010 | 1.237 | 1.522 |
| FBgn0010348 | 0.989 | 0.825 | 0.872 | 0.941 | 1.061 | 1.219 |
| FBgn0050022 | 0.989 | 0.783 | 0.912 | 1.191 | 1.368 | 2.087 |
| FBgn0030041 | 0.988 | 0.916 | 1.011 | 1.091 | 1.321 | 1.620 |
| FBgn0034184 | 0.988 | 0.986 | 1.031 | 1.072 | 1.137 | 1.313 |
| FBgn0039329 | 0.988 | 1.050 | 1.109 | 1.149 | 1.239 | 1.414 |
| FBgn0053120 | 0.988 | 0.714 | 0.739 | 0.769 | 0.881 | 1.021 |
| FBgn0031779 | 0.988 | 1.111 | 1.108 | 1.058 | 0.917 | 0.721 |
| FBgn0037519 | 0.988 | 0.833 | 0.850 | 1.058 | 1.157 | 1.600 |
| FBgn0037036 | 0.988 | 0.746 | 0.823 | 0.868 | 1.018 | 1.253 |
| FBgn0032021 | 0.988 | 0.813 | 0.933 | 1.018 | 1.208 | 1.648 |
| FBgn0034756 | 0.987 | 0.849 | 1.074 | 1.200 | 1.765 | 2.679 |
| FBgn0016650 | 0.987 | 0.813 | 0.863 | 0.924 | 1.101 | 1.301 |
| FBgn0035028 | 0.987 | 0.879 | 0.922 | 0.954 | 1.042 | 1.240 |
| FBgn0002641 | 0.987 | 0.990 | 1.068 | 1.144 | 1.398 | 1.691 |
| FBgn0262902 | 0.987 | 0.791 | 0.771 | 0.998 | 1.104 | 1.516 |
| FBgn0039161 | 0.986 | 0.728 | 0.741 | 0.837 | 1.139 | 1.598 |
| FBgn0033949 | 0.986 | 1.422 | 1.353 | 1.548 | 1.774 | 2.204 |
| FBgn0052368 | 0.985 | 1.082 | 1.700 | 4.955 | 6.366 | 13.820 |
| FBgn0038035 | 0.985 | 1.008 | 1.266 | 1.400 | 1.801 | 2.690 |
| FBgn0033932 | 0.984 | 0.848 | 0.883 | 1.026 | 1.084 | 1.405 |
| FBgn0038632 | 0.984 | 0.894 | 0.909 | 1.101 | 1.455 | 2.318 |
| FBgn0000241 | 0.984 | 0.887 | 0.950 | 1.105 | 1.199 | 1.661 |
| FBgn0035916 | 0.983 | 0.908 | 0.975 | 1.023 | 1.196 | 1.570 |
| FBgn0033926 | 0.983 | 1.509 | 1.533 | 1.747 | 2.548 | 3.675 |
| FBgn0032538 | 0.983 | 0.841 | 1.209 | 1.610 | 2.392 | 4.681 |
| FBgn0035398 | 0.982 | 0.959 | 1.132 | 1.359 | 1.994 | 3.501 |
| FBgn0046763 | 0.982 | 0.738 | 0.875 | 1.061 | 1.225 | 1.614 |
| FBgn0033927 | 0.982 | 1.496 | 1.570 | 1.781 | 2.694 | 3.778 |
| FBgn0011335 | 0.982 | 0.846 | 0.835 | 0.877 | 0.968 | 1.073 |
| FBgn0020270 | 0.981 | 0.916 | 1.036 | 1.096 | 1.529 | 2.202 |
| FBgn0011676 | 0.981 | 0.953 | 0.976 | 1.027 | 1.190 | 1.531 |
| FBgn0030854 | 0.981 | 0.892 | 0.967 | 0.997 | 1.174 | 1.515 |
| FBgn0032230 | 0.981 | 0.893 | 1.122 | 1.343 | 1.544 | 2.284 |
| FBgn0029664 | 0.981 | 0.857 | 0.932 | 0.979 | 1.056 | 1.247 |
| FBgn0038290 | 0.981 | 0.877 | 1.080 | 1.251 | 1.444 | 1.977 |
| FBgn0040827 | 0.981 | 0.850 | 0.845 | 0.911 | 0.994 | 1.242 |
| FBgn0037491 | 0.981 | 0.908 | 0.963 | 1.064 | 1.117 | 1.408 |
| FBgn0032393 | 0.980 | 0.998 | 1.130 | 1.699 | 2.025 | 2.818 |
| FBgn0000039 | 0.980 | 0.838 | 0.880 | 0.918 | 1.151 | 1.417 |
| FBgn0031054 | 0.980 | 1.066 | 1.058 | 1.114 | 1.185 | 1.391 |
| FBgn0032536 | 0.979 | 0.735 | 0.940 | 1.050 | 1.260 | 1.813 |
| FBgn0051352 | 0.979 | 1.125 | 1.058 | 0.997 | 0.912 | 0.762 |
| FBgn0058100 | 0.979 | 0.497 | 0.699 | 0.967 | 1.151 | 1.745 |
| FBgn0032614 | 0.979 | 0.753 | 0.823 | 0.900 | 1.044 | 1.214 |
| FBgn0033215 | 0.979 | 0.701 | 0.928 | 1.107 | 1.306 | 2.014 |
| FBgn0053099 | 0.979 | 0.867 | 0.806 | 0.974 | 1.087 | 1.421 |
| FBgn0035644 | 0.978 | 1.097 | 1.073 | 0.794 | 0.557 | 0.217 |
| FBgn0034605 | 0.978 | 0.961 | 1.046 | 1.370 | 1.555 | 1.999 |
| FBgn0030026 | 0.978 | 0.927 | 0.938 | 1.052 | 1.397 | 1.718 |
| FBgn0000451 | 0.978 | 1.080 | 1.133 | 1.318 | 1.372 | 1.758 |
| FBgn0013750 | 0.978 | 0.695 | 0.787 | 0.828 | 0.940 | 1.140 |
| FBgn0031317 | 0.977 | 1.013 | 0.900 | 1.148 | 1.436 | 1.877 |
| FBgn0052750 | 0.977 | 0.731 | 0.754 | 0.786 | 0.949 | 1.106 |
| FBgn0020278 | 0.977 | 1.073 | 1.070 | 1.088 | 1.151 | 1.220 |
| FBgn0035165 | 0.976 | 0.980 | 0.944 | 1.058 | 1.119 | 1.286 |
| FBgn0037076 | 0.976 | 1.079 | 1.039 | 1.271 | 1.566 | 1.877 |
| FBgn0024912 | 0.975 | 0.778 | 1.051 | 1.549 | 1.898 | 2.703 |
| FBgn0051105 | 0.975 | 0.660 | 0.801 | 1.012 | 1.105 | 1.654 |
| FBgn0259714 | 0.975 | 0.842 | 0.868 | 0.932 | 1.164 | 1.662 |
| FBgn0035512 | 0.975 | 0.348 | 0.691 | 0.868 | 1.317 | 2.682 |
| FBgn0039638 | 0.975 | 1.014 | 0.975 | 0.960 | 0.797 | 0.615 |
| FBgn0034611 | 0.975 | 0.921 | 0.965 | 1.004 | 1.150 | 1.280 |
| FBgn0044047 | 0.974 | 1.007 | 1.000 | 1.092 | 1.258 | 1.708 |
| FBgn0036547 | 0.974 | 1.011 | 0.993 | 1.052 | 1.098 | 1.173 |
| FBgn0036665 | 0.974 | 0.807 | 0.794 | 0.894 | 1.328 | 1.998 |
| FBgn0037466 | 0.974 | 0.969 | 0.983 | 1.127 | 1.160 | 1.389 |
| FBgn0024360 | 0.973 | 1.227 | 1.127 | 1.062 | 0.904 | 0.709 |
| FBgn0051901 | 0.973 | 0.605 | 1.028 | 1.605 | 1.981 | 4.167 |
| FBgn0033453 | 0.973 | 0.658 | 0.835 | 1.168 | 1.339 | 1.900 |
| FBgn0035673 | 0.973 | 0.659 | 0.876 | 1.001 | 1.263 | 1.677 |
| FBgn0003721 | 0.973 | 1.061 | 1.055 | 1.100 | 1.241 | 1.370 |
| FBgn0260386 | 0.973 | 0.843 | 0.866 | 0.892 | 1.082 | 1.399 |
| FBgn0038761 | 0.972 | 1.239 | 1.197 | 1.361 | 1.459 | 1.653 |
| FBgn0046878 | 0.972 | 0.708 | 1.125 | 1.367 | 1.959 | 2.738 |
| FBgn0050281 | 0.972 | 0.753 | 0.877 | 1.037 | 1.111 | 1.507 |
| FBgn0036145 | 0.972 | 0.635 | 0.748 | 0.879 | 1.177 | 1.453 |
| FBgn0033867 | 0.972 | 0.944 | 1.256 | 1.429 | 1.743 | 2.884 |
| FBgn0032233 | 0.972 | 0.834 | 0.903 | 0.929 | 1.046 | 1.359 |
| FBgn0038092 | 0.972 | 0.584 | 0.740 | 0.838 | 1.017 | 1.681 |
| FBgn0031726 | 0.971 | 1.083 | 0.984 | 1.164 | 1.639 | 2.257 |
| FBgn0037144 | 0.971 | 1.039 | 0.984 | 1.084 | 1.229 | 1.534 |
| FBgn0262366 | 0.971 | 0.971 | 1.050 | 2.472 | 2.858 | 4.778 |
| FBgn0031389 | 0.971 | 0.688 | 0.849 | 0.892 | 1.436 | 1.982 |
| FBgn0052579 | 0.971 | 1.156 | 1.067 | 1.231 | 1.467 | 1.981 |
| FBgn0052533 | 0.971 | 1.168 | 1.167 | 1.142 | 1.113 | 1.003 |
| FBgn0039597 | 0.971 | 0.775 | 0.789 | 0.944 | 0.988 | 1.195 |
| FBgn0046222 | 0.971 | 1.052 | 1.014 | 0.955 | 0.896 | 0.803 |
| FBgn0035577 | 0.971 | 1.273 | 1.178 | 1.358 | 1.910 | 2.622 |
| FBgn0035166 | 0.971 | 0.922 | 1.024 | 1.081 | 1.357 | 1.602 |
| FBgn0031893 | 0.971 | 0.935 | 0.892 | 1.017 | 1.071 | 1.266 |
| FBgn0053635 | 0.971 | 1.006 | 1.066 | 1.151 | 1.603 | 2.526 |
| FBgn0003071 | 0.970 | 1.110 | 1.111 | 0.953 | 0.816 | 0.647 |
| FBgn0052675 | 0.970 | 1.063 | 1.025 | 0.981 | 0.934 | 0.854 |
| FBgn0036646 | 0.970 | 1.201 | 1.165 | 1.050 | 0.849 | 0.679 |
| FBgn0051956 | 0.970 | 0.839 | 0.878 | 0.928 | 1.105 | 1.246 |
| FBgn0030038 | 0.970 | 1.093 | 1.024 | 0.957 | 0.885 | 0.744 |
| FBgn0051864 | 0.970 | 1.100 | 1.072 | 1.596 | 1.711 | 2.806 |
| FBgn0034931 | 0.970 | 0.932 | 0.930 | 1.027 | 1.080 | 1.184 |
| FBgn0034152 | 0.970 | 0.567 | 1.384 | 2.129 | 4.491 | 6.484 |
| FBgn0033240 | 0.969 | 0.670 | 0.748 | 0.934 | 1.014 | 1.291 |
| FBgn0031270 | 0.969 | 0.579 | 0.879 | 0.980 | 1.406 | 1.949 |
| FBgn0034480 | 0.969 | 0.917 | 1.195 | 1.283 | 1.589 | 2.498 |
| FBgn0053105 | 0.969 | 1.126 | 1.073 | 1.068 | 0.942 | 0.804 |
| FBgn0051710 | 0.969 | 0.687 | 0.835 | 1.148 | 1.227 | 1.856 |
| FBgn0000173 | 0.969 | 0.893 | 0.994 | 1.040 | 1.207 | 1.390 |
| FBgn0260463 | 0.969 | 1.126 | 1.073 | 1.068 | 0.942 | 0.807 |
| FBgn0036486 | 0.968 | 1.117 | 1.174 | 0.921 | 0.682 | 0.404 |
| FBgn0032644 | 0.968 | 0.762 | 0.812 | 1.013 | 1.092 | 1.351 |
| FBgn0035523 | 0.968 | 1.023 | 1.022 | 1.057 | 1.270 | 1.486 |
| FBgn0011774 | 0.968 | 1.075 | 1.035 | 1.228 | 1.975 | 2.650 |
| FBgn0039240 | 0.968 | 1.060 | 1.057 | 0.933 | 0.853 | 0.723 |
| FBgn0030799 | 0.968 | 0.781 | 0.864 | 1.023 | 1.168 | 1.386 |
| FBgn0262872 | 0.968 | 1.201 | 1.162 | 1.057 | 0.964 | 0.835 |
| FBgn0036942 | 0.968 | 0.788 | 0.887 | 0.968 | 1.226 | 1.444 |
| FBgn0039599 | 0.967 | 0.782 | 0.809 | 0.940 | 1.055 | 1.199 |
| FBgn0040348 | 0.967 | 0.990 | 1.043 | 1.100 | 1.142 | 1.258 |
| FBgn0262147 | 0.967 | 0.706 | 0.794 | 0.897 | 0.984 | 1.173 |
| FBgn0036489 | 0.967 | 0.940 | 0.902 | 1.050 | 1.090 | 1.340 |
| FBgn0259144 | 0.967 | 1.167 | 1.104 | 1.200 | 1.339 | 1.591 |
| FBgn0037617 | 0.967 | 0.973 | 1.017 | 1.029 | 1.318 | 1.645 |
| FBgn0032684 | 0.967 | 0.867 | 0.963 | 0.966 | 1.137 | 1.483 |
| FBgn0000152 | 0.966 | 0.985 | 0.942 | 1.153 | 1.281 | 1.501 |
| FBgn0015038 | 0.966 | 0.568 | 0.891 | 1.009 | 1.261 | 2.064 |
| FBgn0052544 | 0.966 | 0.975 | 1.020 | 1.025 | 1.078 | 1.193 |
| FBgn0032283 | 0.966 | 1.499 | 1.537 | 1.441 | 1.251 | 1.082 |
| FBgn0039703 | 0.966 | 0.932 | 0.944 | 1.014 | 1.026 | 1.194 |
| FBgn0037684 | 0.965 | 0.861 | 0.798 | 0.931 | 1.163 | 1.390 |
| FBgn0036768 | 0.965 | 0.791 | 0.941 | 0.947 | 1.237 | 1.565 |
| FBgn0032105 | 0.965 | 1.382 | 1.079 | 0.993 | 0.659 | 0.134 |
| FBgn0039015 | 0.964 | 0.776 | 1.032 | 1.162 | 1.324 | 1.965 |
| FBgn0032225 | 0.964 | 0.776 | 0.964 | 0.973 | 1.253 | 1.622 |
| FBgn0010038 | 0.964 | 2.798 | 2.277 | 5.455 | 6.142 | 10.345 |
| FBgn0029672 | 0.963 | 1.031 | 0.920 | 1.171 | 1.332 | 1.974 |
| FBgn0023514 | 0.963 | 1.048 | 0.989 | 1.071 | 1.205 | 1.423 |
| FBgn0031910 | 0.963 | 1.013 | 0.919 | 1.437 | 2.976 | 4.141 |
| FBgn0028399 | 0.963 | 0.873 | 0.934 | 1.071 | 1.157 | 1.331 |
| FBgn0027780 | 0.962 | 0.631 | 0.890 | 0.946 | 1.266 | 1.694 |
| FBgn0028919 | 0.962 | 1.076 | 0.987 | 1.104 | 1.333 | 1.714 |
| FBgn0037882 | 0.961 | 0.977 | 0.853 | 1.114 | 1.427 | 1.767 |
| FBgn0030258 | 0.961 | 0.831 | 0.857 | 0.904 | 1.059 | 1.163 |
| FBgn0033134 | 0.960 | 0.550 | 0.868 | 1.056 | 1.333 | 1.866 |
| FBgn0259740 | 0.960 | NA | 1.263 | 1.291 | 1.443 | 1.847 |
| FBgn0261989 | 0.960 | 1.254 | 1.148 | 2.397 | 3.470 | 8.701 |
| FBgn0038894 | 0.960 | 0.907 | 0.805 | 1.182 | 1.536 | 1.913 |
| FBgn0004378 | 0.960 | 1.389 | 1.292 | 1.032 | 0.708 | 0.410 |
| FBgn0036822 | 0.960 | 0.524 | 0.852 | 0.975 | 1.285 | 1.815 |
| FBgn0038354 | 0.960 | 0.849 | 0.954 | 0.952 | 1.271 | 1.564 |
| FBgn0033397 | 0.960 | 0.749 | 0.927 | 0.889 | 1.347 | 1.992 |
| FBgn0039681 | 0.960 | 0.660 | 0.773 | 0.816 | 0.887 | 1.163 |
| FBgn0035206 | 0.960 | 0.862 | 0.886 | 0.935 | 1.037 | 1.109 |
| FBgn0036629 | 0.960 | 0.978 | 1.061 | 1.047 | 1.419 | 1.840 |
| FBgn0032405 | 0.959 | 0.589 | 0.854 | 1.153 | 1.316 | 1.899 |
| FBgn0029765 | 0.959 | 0.747 | 0.886 | 1.154 | 1.186 | 1.820 |
| FBgn0023395 | 0.959 | 1.130 | 1.202 | 0.938 | 0.541 | 0.227 |
| FBgn0032124 | 0.958 | 0.818 | 0.940 | 1.172 | 1.506 | 1.800 |
| FBgn0262524 | 0.958 | 0.948 | 1.069 | 1.440 | 1.502 | 2.592 |
| FBgn0020312 | 0.958 | 0.953 | 1.006 | 1.124 | 1.237 | 1.373 |
| FBgn0032981 | 0.958 | 0.508 | 0.805 | 0.797 | 1.262 | 2.350 |
| FBgn0030833 | 0.958 | 1.200 | 1.078 | 1.023 | 0.955 | 0.649 |
| FBgn0033744 | 0.958 | 0.748 | 0.902 | 0.913 | 1.058 | 1.414 |
| FBgn0039451 | 0.958 | 1.097 | 1.046 | 0.912 | 0.807 | 0.659 |
| FBgn0037071 | 0.957 | 0.681 | 1.059 | 1.190 | 1.524 | 2.130 |
| FBgn0028411 | 0.957 | 1.292 | 1.246 | 1.759 | 1.816 | 2.514 |
| FBgn0035587 | 0.957 | 0.921 | 0.873 | 0.971 | 1.091 | 1.215 |
| FBgn0036157 | 0.957 | 0.954 | 1.018 | 1.074 | 1.228 | 1.345 |
| FBgn0038069 | 0.957 | 0.561 | 0.737 | 0.822 | 0.913 | 1.327 |
| FBgn0002924 | 0.957 | 1.215 | 1.021 | 0.882 | 0.682 | 0.376 |
| FBgn0030481 | 0.956 | 1.042 | 1.082 | 1.025 | 0.910 | 0.790 |
| FBgn0031650 | 0.956 | 0.515 | 0.456 | 0.823 | 0.867 | 1.381 |
| FBgn0033038 | 0.956 | 0.837 | 0.929 | 0.951 | 1.059 | 1.198 |
| FBgn0030410 | 0.956 | 1.022 | 0.998 | 0.951 | 0.847 | 0.463 |
| FBgn0261284 | 0.956 | 0.923 | 0.938 | 1.284 | 1.435 | 1.767 |
| FBgn0028987 | 0.956 | 0.596 | 0.915 | 0.910 | 1.253 | 1.900 |
| FBgn0035876 | 0.956 | 0.744 | 0.871 | 0.999 | 1.104 | 1.335 |
| FBgn0034139 | 0.956 | 1.080 | 0.890 | 1.173 | 1.422 | 2.064 |
| FBgn0034656 | 0.956 | 0.886 | 0.855 | 0.889 | 1.025 | 1.186 |
| FBgn0039304 | 0.956 | 0.947 | 1.028 | 1.144 | 1.166 | 1.425 |
| FBgn0053139 | 0.955 | 1.103 | 1.123 | 0.969 | 0.862 | 0.726 |
| FBgn0031145 | 0.955 | 0.612 | 0.879 | 1.014 | 1.178 | 1.667 |
| FBgn0038414 | 0.955 | 0.636 | 0.819 | 0.775 | 1.334 | 1.922 |
| FBgn0052590 | 0.955 | 0.741 | 0.665 | 0.754 | 0.924 | 1.205 |
| FBgn0003612 | 0.955 | 0.971 | 1.028 | 0.853 | 0.775 | 0.593 |
| FBgn0003559 | 0.955 | 0.928 | 0.982 | 1.043 | 1.223 | 1.343 |
| FBgn0039670 | 0.955 | 0.718 | 0.942 | 1.234 | 1.551 | 1.961 |
| FBgn0033753 | 0.955 | 0.831 | 1.147 | 1.313 | 1.460 | 2.223 |
| FBgn0034517 | 0.955 | 1.151 | 1.000 | 1.236 | 1.415 | 1.826 |
| FBgn0043536 | 0.955 | 1.151 | 1.000 | 1.236 | 1.415 | 1.826 |
| FBgn0050151 | 0.955 | 0.620 | 0.884 | 1.029 | 1.218 | 1.654 |
| FBgn0037167 | 0.954 | 1.076 | 1.096 | 1.261 | 1.276 | 1.503 |
| FBgn0039467 | 0.954 | 0.745 | 0.855 | 0.853 | 1.003 | 1.394 |
| FBgn0038595 | 0.954 | 0.755 | 0.660 | 0.871 | 0.951 | 1.239 |
| FBgn0260940 | 0.954 | 0.816 | 0.803 | 0.909 | 0.916 | 1.082 |
| FBgn0038366 | 0.954 | 0.537 | 0.871 | 0.954 | 1.198 | 2.177 |
| FBgn0015714 | 0.953 | 0.671 | 0.908 | 0.972 | 1.156 | 1.542 |
| FBgn0032391 | 0.953 | 0.789 | 1.008 | 1.357 | 1.430 | 2.070 |
| FBgn0036848 | 0.953 | 0.946 | 0.905 | 1.035 | 1.171 | 1.296 |
| FBgn0039767 | 0.953 | 1.124 | 1.055 | 1.040 | 0.819 | 0.662 |
| FBgn0038498 | 0.953 | 0.821 | 0.866 | 0.860 | 1.097 | 1.305 |
| FBgn0029836 | 0.953 | 1.349 | 1.330 | 1.460 | 2.054 | 3.484 |
| FBgn0028562 | 0.953 | 0.660 | 0.855 | 1.057 | 1.197 | 1.560 |
| FBgn0026418 | 0.953 | 1.078 | 1.094 | 0.991 | 0.943 | 0.850 |
| FBgn0037473 | 0.953 | 0.885 | 0.881 | 0.981 | 0.985 | 1.136 |
| FBgn0037850 | 0.952 | 0.914 | 1.263 | 2.117 | 2.280 | 3.514 |
| FBgn0036910 | 0.952 | 1.054 | 1.068 | 0.917 | 0.896 | 0.721 |
| FBgn0058160 | 0.952 | 1.061 | 1.064 | 1.074 | 1.415 | 1.775 |
| FBgn0003969 | 0.952 | 0.909 | 1.106 | 1.133 | 1.290 | 1.631 |
| FBgn0087039 | 0.952 | 0.686 | 0.925 | 1.002 | 1.149 | 1.582 |
| FBgn0085819 | 0.952 | 0.746 | 1.045 | 1.321 | 1.399 | 2.310 |
| FBgn0036749 | 0.951 | 0.944 | 1.029 | 1.044 | 1.264 | 1.870 |
| FBgn0039332 | 0.951 | 0.753 | 1.005 | 1.021 | 1.247 | 1.675 |
| FBgn0010470 | 0.951 | 0.858 | 0.868 | 1.044 | 1.145 | 1.300 |
| FBgn0260431 | 0.951 | 0.892 | 1.023 | 1.119 | 1.171 | 1.632 |
| FBgn0027093 | 0.951 | 0.891 | 0.946 | 0.932 | 1.033 | 1.210 |
| FBgn0023416 | 0.951 | 0.647 | 0.885 | 0.897 | 1.102 | 1.541 |
| FBgn0011754 | 0.950 | 0.977 | 0.991 | 1.056 | 1.107 | 1.166 |
| FBgn0042085 | 0.950 | 1.126 | 1.081 | 0.854 | 0.712 | 0.503 |
| FBgn0039068 | 0.950 | 0.860 | 1.089 | 1.204 | 1.360 | 1.721 |
| FBgn0035914 | 0.950 | 1.007 | 1.008 | 1.012 | 1.194 | 1.386 |
| FBgn0052672 | 0.950 | 0.815 | 0.864 | 0.999 | 1.129 | 1.263 |
| FBgn0039635 | 0.949 | 1.098 | 1.022 | 1.038 | 0.924 | 0.768 |
| FBgn0030157 | 0.949 | 0.654 | 0.846 | 0.849 | 1.011 | 1.418 |
| FBgn0261446 | 0.949 | 0.719 | 0.921 | 1.037 | 1.228 | 1.515 |
| FBgn0052428 | 0.949 | 0.887 | 0.915 | 1.028 | 1.263 | 1.403 |
| FBgn0029827 | 0.949 | 0.885 | 0.896 | 0.969 | 1.214 | 1.355 |
| FBgn0011741 | 0.949 | 1.038 | 1.023 | 1.119 | 1.365 | 1.514 |
| FBgn0051769 | 0.949 | 0.681 | 0.781 | 0.964 | 0.992 | 1.300 |
| FBgn0032235 | 0.949 | 0.639 | 0.829 | 1.047 | 1.133 | 1.539 |
| FBgn0039319 | 0.949 | 0.472 | 1.071 | 1.062 | 1.770 | 2.699 |
| FBgn0011694 | 0.949 | 0.477 | 0.721 | 0.843 | 0.962 | 1.773 |
| FBgn0014020 | 0.949 | 0.867 | 0.883 | 0.891 | 0.985 | 1.220 |
| FBgn0032204 | 0.949 | 0.941 | 0.988 | 1.141 | 1.156 | 1.633 |
| FBgn0027538 | 0.948 | 0.869 | 0.997 | 0.973 | 1.165 | 1.583 |
| FBgn0051274 | 0.948 | 0.747 | 0.581 | 0.966 | 1.195 | 2.428 |
| FBgn0038412 | 0.948 | 0.938 | 1.095 | 1.238 | 1.274 | 1.704 |
| FBgn0036058 | 0.948 | 0.940 | 0.945 | 1.030 | 1.129 | 1.203 |
| FBgn0036819 | 0.948 | 0.801 | 0.890 | 0.899 | 0.981 | 1.277 |
| FBgn0025366 | 0.948 | 0.979 | 0.889 | 1.024 | 1.139 | 1.336 |
| FBgn0030241 | 0.948 | 1.655 | 1.277 | 1.188 | 0.716 | 0.217 |
| FBgn0035975 | 0.947 | 0.687 | 0.882 | 0.973 | 1.074 | 1.420 |
| FBgn0013764 | 0.947 | 1.170 | 1.031 | 1.001 | 0.876 | 0.680 |
| FBgn0032023 | 0.947 | 0.949 | 0.834 | 1.028 | 1.189 | 1.823 |
| FBgn0034293 | 0.947 | 0.527 | 0.997 | 1.075 | 1.730 | 4.052 |
| FBgn0052641 | 0.947 | 0.534 | 0.799 | 0.915 | 1.109 | 2.212 |
| FBgn0028424 | 0.947 | 0.754 | 0.776 | 0.854 | 1.168 | 1.345 |
| FBgn0036697 | 0.947 | 0.848 | 0.884 | 0.881 | 0.948 | 1.006 |
| FBgn0011589 | 0.947 | 1.009 | 1.008 | 1.008 | 1.227 | 1.508 |
| FBgn0022246 | 0.946 | 0.665 | 0.948 | 1.101 | 1.300 | 1.722 |
| FBgn0052640 | 0.946 | 0.535 | 0.815 | 0.933 | 1.120 | 2.219 |
| FBgn0035089 | 0.946 | 0.749 | 0.903 | 1.234 | 1.232 | 2.122 |
| FBgn0015351 | 0.946 | 0.617 | 1.072 | 1.131 | 1.446 | 2.242 |
| FBgn0038455 | 0.946 | 0.617 | 1.072 | 1.131 | 1.446 | 2.242 |
| FBgn0067782 | 0.946 | 1.127 | 1.061 | 1.034 | 0.823 | 0.694 |
| FBgn0025628 | 0.946 | 1.061 | 1.142 | 1.139 | 1.222 | 1.351 |
| FBgn0052383 | 0.946 | 0.432 | 0.421 | 0.565 | 0.643 | 1.237 |
| FBgn0083970 | 0.946 | 1.084 | 1.168 | 1.155 | 1.304 | 1.445 |
| FBgn0026737 | 0.946 | 0.726 | 0.936 | 1.112 | 1.157 | 1.737 |
| FBgn0034398 | 0.945 | 0.695 | 0.837 | 1.078 | 1.174 | 1.495 |
| FBgn0054054 | 0.945 | 0.293 | 0.487 | 0.859 | 0.903 | 2.234 |
| FBgn0035157 | 0.945 | 0.773 | 1.207 | 1.276 | 1.572 | 2.291 |
| FBgn0085476 | 0.945 | 0.743 | 0.879 | 0.829 | 1.208 | 1.570 |
| FBgn0034328 | 0.945 | 1.497 | 1.100 | 2.096 | 2.305 | 3.648 |
| FBgn0032682 | 0.945 | 0.673 | 0.908 | 0.869 | 1.246 | 1.639 |
| FBgn0020521 | 0.945 | 1.098 | 1.097 | 1.123 | 1.334 | 1.475 |
| FBgn0008651 | 0.945 | 0.676 | 0.915 | 1.093 | 1.344 | 1.675 |
| FBgn0035477 | 0.945 | 0.947 | 0.982 | 0.900 | 0.746 | 0.643 |
| FBgn0038058 | 0.944 | 0.846 | 1.052 | 1.080 | 1.208 | 1.696 |
| FBgn0022029 | 0.944 | 0.809 | 0.898 | 1.072 | 1.126 | 1.353 |
| FBgn0051036 | 0.944 | 0.960 | 1.026 | 1.277 | 1.386 | 2.519 |
| FBgn0033980 | 0.944 | 0.418 | 0.700 | 0.646 | 1.162 | 1.651 |
| FBgn0010173 | 0.944 | 0.886 | 1.079 | 1.116 | 1.383 | 1.629 |
| FBgn0036154 | 0.943 | 0.648 | 0.763 | 0.817 | 0.897 | 1.399 |
| FBgn0030055 | 0.943 | 0.988 | 1.024 | 1.028 | 1.144 | 1.217 |
| FBgn0030189 | 0.943 | 0.785 | 0.992 | 1.010 | 1.195 | 1.489 |
| FBgn0037020 | 0.943 | 0.897 | 0.927 | 1.089 | 1.107 | 1.298 |
| FBgn0067783 | 0.943 | 1.103 | 1.050 | 1.016 | 0.843 | 0.744 |
| FBgn0037655 | 0.943 | 1.177 | 1.142 | 1.104 | 1.084 | 0.871 |
| FBgn0040358 | 0.943 | 1.066 | 1.085 | 1.058 | NA | 3.059 |
| FBgn0038057 | 0.943 | 0.961 | 0.923 | 0.941 | 1.295 | 1.728 |
| FBgn0039596 | 0.942 | 1.538 | 1.237 | 1.532 | 2.621 | 4.923 |
| FBgn0051189 | 0.942 | 0.430 | 0.546 | 0.913 | 1.015 | 1.418 |
| FBgn0030234 | 0.942 | 0.881 | 1.070 | 1.079 | 1.227 | 1.527 |
| FBgn0010280 | 0.942 | 1.171 | 1.090 | 0.981 | 0.909 | 0.766 |
| FBgn0261722 | 0.942 | 0.932 | 0.952 | 1.081 | 1.224 | 1.330 |
| FBgn0035449 | 0.942 | 1.027 | 1.035 | 1.039 | 1.104 | 1.144 |
| FBgn0043069 | 0.942 | 0.792 | 0.569 | 0.922 | 1.256 | 2.562 |
| FBgn0038734 | 0.942 | 0.845 | 0.973 | 1.021 | 1.392 | 1.611 |
| FBgn0001185 | 0.942 | 0.898 | 0.910 | 1.077 | 1.088 | 1.588 |
| FBgn0034269 | 0.942 | 1.033 | 1.010 | 0.934 | 0.940 | 0.766 |
| FBgn0039481 | 0.942 | 0.896 | 0.848 | 0.932 | 0.979 | 1.215 |
| FBgn0032192 | 0.941 | 0.612 | 0.671 | 1.241 | 1.419 | 1.938 |
| FBgn0051300 | 0.941 | 0.450 | 0.740 | 0.717 | 1.031 | 1.466 |
| FBgn0000370 | 0.941 | 0.761 | 0.986 | 1.005 | 1.195 | 1.514 |
| FBgn0040250 | 0.941 | 0.506 | 1.068 | 1.418 | 1.662 | 3.973 |
| FBgn0010772 | 0.941 | 0.992 | 1.033 | 1.090 | 1.299 | 1.407 |
| FBgn0050055 | 0.940 | 1.064 | 0.899 | 1.143 | 1.361 | 1.674 |
| FBgn0034365 | 0.940 | 0.813 | 0.973 | 1.007 | 1.319 | 1.533 |
| FBgn0015625 | 0.940 | 1.743 | 1.352 | 1.182 | 0.557 | 0.078 |
| FBgn0085353 | 0.940 | 0.936 | 0.871 | 0.957 | 1.029 | 1.167 |
| FBgn0261929 | 0.940 | 0.820 | 0.901 | 1.005 | 1.012 | 1.390 |
| FBgn0030800 | 0.940 | 1.070 | 1.016 | 1.113 | 1.282 | 1.402 |
| FBgn0038465 | 0.939 | 0.731 | 0.862 | 0.839 | 1.064 | 1.269 |
| FBgn0033348 | 0.939 | 0.928 | 1.160 | 1.170 | 1.711 | 3.379 |
| FBgn0260874 | 0.939 | 0.529 | 0.999 | 1.069 | 1.390 | 2.092 |
| FBgn0036396 | 0.939 | 0.802 | 0.744 | 0.814 | 1.047 | 1.223 |
| FBgn0037730 | 0.939 | 0.940 | 0.879 | 0.945 | 1.127 | 1.576 |
| FBgn0003495 | 0.939 | 0.758 | 0.988 | 0.997 | 1.363 | 1.662 |
| FBgn0005616 | 0.938 | 0.994 | 0.990 | 1.012 | 1.063 | 1.251 |
| FBgn0013771 | 0.938 | 0.555 | 0.753 | 0.846 | 1.001 | 1.258 |
| FBgn0052111 | 0.938 | 0.553 | 0.967 | 0.911 | 1.313 | 2.425 |
| FBgn0034002 | 0.938 | 0.961 | 0.938 | 1.205 | 1.186 | 1.650 |
| FBgn0052843 | 0.938 | 0.883 | 0.915 | 0.963 | 1.195 | 1.311 |
| FBgn0085419 | 0.938 | 0.967 | 0.895 | 0.997 | 1.090 | 1.445 |
| FBgn0037244 | 0.938 | 0.892 | 0.977 | 0.979 | 1.037 | 1.192 |
| FBgn0036970 | 0.938 | 0.721 | 0.826 | 0.811 | 1.031 | 1.198 |
| FBgn0021873 | 0.938 | 1.138 | 1.178 | 0.969 | 0.723 | 0.558 |
| FBgn0035998 | 0.938 | 0.790 | 0.959 | 0.911 | 1.118 | 1.493 |
| FBgn0259178 | 0.938 | 1.050 | 1.025 | 1.038 | 1.264 | 1.473 |
| FBgn0012051 | 0.937 | 0.753 | 0.940 | 0.924 | 1.090 | 1.391 |
| FBgn0030984 | 0.937 | 0.949 | 0.931 | 0.938 | 1.113 | 1.287 |
| FBgn0030354 | 0.937 | 1.034 | 1.096 | 0.909 | 0.844 | 0.676 |
| FBgn0037731 | 0.937 | 0.531 | 0.973 | 1.059 | 1.436 | 1.996 |
| FBgn0036101 | 0.936 | 0.527 | 0.791 | 0.757 | 1.112 | 1.479 |
| FBgn0037150 | 0.936 | 0.631 | 0.897 | 0.995 | 1.085 | 1.641 |
| FBgn0261245 | 0.936 | 1.032 | 1.027 | 1.249 | 1.352 | 1.521 |
| FBgn0035661 | 0.936 | 0.628 | 0.868 | 0.971 | 1.260 | 1.536 |
| FBgn0040323 | 0.936 | 0.925 | 0.841 | 0.964 | 1.259 | 1.459 |
| FBgn0030827 | 0.936 | 0.744 | 0.920 | 0.924 | 1.054 | 1.320 |
| FBgn0034093 | 0.935 | 0.911 | 0.999 | 1.051 | 1.348 | 1.501 |
| FBgn0030778 | 0.935 | 0.884 | 0.969 | 0.951 | 1.178 | 1.337 |
| FBgn0004366 | 0.935 | 0.776 | 0.936 | 1.017 | 1.110 | 1.333 |
| FBgn0039690 | 0.935 | 0.857 | 0.956 | 0.948 | 1.170 | 1.319 |
| FBgn0039272 | 0.935 | 0.877 | 0.874 | 0.975 | 1.174 | 2.034 |
| FBgn0029997 | 0.935 | 1.074 | 1.039 | 0.881 | 0.765 | 0.641 |
| FBgn0015818 | 0.935 | 1.154 | 1.041 | 1.054 | 0.953 | 0.774 |
| FBgn0031091 | 0.935 | 1.240 | 1.197 | 1.144 | 0.589 | 0.288 |
| FBgn0036757 | 0.935 | 0.918 | 1.082 | 1.133 | 1.713 | 3.636 |
| FBgn0035677 | 0.934 | 0.910 | 1.038 | 1.019 | 1.134 | 1.353 |
| FBgn0025627 | 0.934 | 0.728 | 1.002 | 0.963 | 1.259 | 1.659 |
| FBgn0038610 | 0.934 | 1.103 | 1.089 | 1.300 | 1.297 | 1.551 |
| FBgn0038172 | 0.934 | 0.822 | 0.849 | 0.893 | 1.100 | 1.198 |
| FBgn0036187 | 0.934 | 1.154 | 1.112 | 1.397 | 1.475 | 1.711 |
| FBgn0039780 | 0.934 | 0.739 | 0.965 | 1.086 | 1.177 | 1.542 |
| FBgn0036428 | 0.934 | 0.898 | 1.013 | 1.060 | 1.201 | 1.329 |
| FBgn0024913 | 0.934 | 0.617 | 0.941 | 1.054 | 1.342 | 3.008 |
| FBgn0033153 | 0.934 | 1.040 | 1.028 | 1.422 | 1.980 | 2.291 |
| FBgn0034537 | 0.933 | 0.782 | 0.818 | 0.835 | 1.052 | 1.164 |
| FBgn0031643 | 0.933 | 0.850 | 0.955 | 0.869 | 1.519 | 2.113 |
| FBgn0051321 | 0.933 | 0.881 | 0.995 | 0.939 | 1.148 | 1.464 |
| FBgn0029861 | 0.933 | 1.265 | 1.108 | 1.032 | 0.820 | 0.646 |
| FBgn0085822 | 0.933 | 0.767 | 1.671 | 2.491 | 2.872 | 7.988 |
| FBgn0050203 | 0.932 | 0.703 | 0.897 | 0.955 | 1.025 | 1.564 |
| FBgn0261882 | 0.932 | 0.636 | 0.487 | 0.844 | 0.881 | 1.546 |
| FBgn0031213 | 0.932 | 0.630 | 1.062 | 1.175 | 1.401 | 2.036 |
| FBgn0027111 | 0.932 | 0.875 | 0.967 | 0.993 | 1.162 | 1.269 |
| FBgn0086913 | 0.932 | 0.889 | 0.864 | 0.977 | 1.005 | 1.101 |
| FBgn0039678 | 0.931 | 1.202 | 1.037 | 0.837 | 0.286 | 0.013 |
| FBgn0040398 | 0.931 | 1.023 | 0.959 | 1.141 | 1.254 | 1.391 |
| FBgn0030556 | 0.931 | 0.995 | 1.044 | 0.994 | 0.872 | 0.767 |
| FBgn0011591 | 0.931 | 0.847 | 0.979 | 1.028 | 1.205 | 1.348 |
| FBgn0030357 | 0.931 | 0.669 | 0.950 | 1.055 | 1.146 | 2.010 |
| FBgn0032793 | 0.931 | 0.858 | 0.997 | 1.138 | 1.155 | 1.815 |
| FBgn0046322 | 0.931 | 0.628 | 1.114 | 1.283 | 1.482 | 3.165 |
| FBgn0038371 | 0.931 | 1.172 | 1.087 | 1.503 | 1.585 | 1.950 |
| FBgn0051157 | 0.931 | 0.607 | 1.225 | 1.427 | 1.621 | 2.873 |
| FBgn0002775 | 0.931 | 1.100 | 1.132 | 1.061 | 0.854 | 0.744 |
| FBgn0010516 | 0.931 | 1.047 | 1.011 | 1.076 | 1.168 | 1.233 |
| FBgn0003965 | 0.931 | 1.069 | 1.153 | 1.167 | 1.372 | 1.487 |
| FBgn0037703 | 0.931 | 0.768 | 0.946 | 0.915 | 1.139 | 1.382 |
| FBgn0024191 | 0.931 | 1.206 | 1.177 | 0.995 | 0.571 | 0.379 |
| FBgn0031996 | 0.930 | 0.904 | 1.053 | 1.196 | 1.270 | 1.506 |
| FBgn0085433 | 0.930 | 0.859 | 1.004 | 0.992 | 1.098 | 1.345 |
| FBgn0032472 | 0.930 | 0.474 | 0.768 | 0.907 | 0.975 | 1.588 |
| FBgn0035400 | 0.930 | 1.060 | 1.054 | 0.885 | 0.777 | 0.660 |
| FBgn0039630 | 0.930 | 0.952 | 1.387 | 1.580 | 2.020 | 2.503 |
| FBgn0036493 | 0.930 | 0.922 | 0.962 | 0.918 | 1.226 | 1.527 |
| FBgn0036775 | 0.930 | 1.141 | 1.077 | 1.113 | 0.864 | 0.671 |
| FBgn0083966 | 0.930 | 0.584 | 0.830 | 0.927 | 0.995 | 1.477 |
| FBgn0032805 | 0.930 | 0.686 | 1.071 | 1.338 | 1.514 | 2.088 |
| FBgn0037707 | 0.930 | 1.216 | 1.110 | 1.112 | 0.745 | 0.543 |
| FBgn0025583 | 0.930 | 0.549 | 0.860 | 1.225 | 1.327 | 1.917 |
| FBgn0034518 | 0.930 | 0.648 | 0.876 | 0.810 | 1.066 | 1.481 |
| FBgn0052155 | 0.929 | 0.557 | 0.777 | 0.717 | 1.088 | 1.416 |
| FBgn0034519 | 0.929 | 0.647 | 0.873 | 0.807 | 1.056 | 1.476 |
| FBgn0036969 | 0.929 | 0.755 | 0.864 | 0.805 | 1.026 | 1.295 |
| FBgn0038460 | 0.929 | 1.007 | 1.042 | 1.238 | 1.209 | 1.708 |
| FBgn0027581 | 0.929 | 1.020 | 1.061 | 1.095 | 1.285 | 1.371 |
| FBgn0002938 | 0.929 | 0.716 | 0.831 | 0.803 | 1.038 | 1.212 |
| FBgn0030331 | 0.929 | 0.962 | 1.057 | 1.307 | 1.587 | 1.787 |
| FBgn0033821 | 0.929 | 0.828 | 1.015 | 0.951 | 1.226 | 1.932 |
| FBgn0033693 | 0.928 | 1.135 | 1.228 | 1.141 | 0.996 | 0.794 |
| FBgn0053964 | 0.928 | 1.135 | 1.228 | 1.141 | 0.996 | 0.794 |
| FBgn0000629 | 0.928 | 0.887 | 0.915 | 0.963 | 1.103 | 1.165 |
| FBgn0036003 | 0.928 | 1.150 | 1.090 | 0.967 | 0.741 | 0.626 |
| FBgn0037855 | 0.928 | 0.987 | 0.955 | 0.998 | 1.102 | 1.170 |
| FBgn0039856 | 0.928 | 1.042 | 1.028 | 1.043 | 0.908 | 0.796 |
| FBgn0034726 | 0.928 | 0.690 | 0.890 | 0.988 | 1.070 | 1.367 |
| FBgn0011762 | 0.927 | 0.797 | 0.822 | 0.816 | 1.930 | 2.657 |
| FBgn0030912 | 0.927 | 1.184 | 1.038 | 1.143 | 1.745 | 2.225 |
| FBgn0032859 | 0.927 | 1.125 | 1.152 | 1.141 | 1.179 | 1.232 |
| FBgn0040972 | 0.926 | 0.500 | 0.829 | 1.270 | 1.317 | 3.444 |
| FBgn0033130 | 0.926 | 0.746 | 1.019 | 1.233 | 1.251 | 1.886 |
| FBgn0027106 | 0.926 | 0.841 | 0.881 | 1.044 | 1.132 | 1.258 |
| FBgn0026374 | 0.926 | 0.729 | 0.968 | 1.124 | 1.323 | 1.603 |
| FBgn0067864 | 0.926 | 1.092 | 1.115 | 0.978 | 0.979 | 0.825 |
| FBgn0020415 | 0.925 | 0.736 | 0.875 | 0.919 | 1.019 | 1.180 |
| FBgn0052183 | 0.925 | 1.051 | 1.147 | 1.119 | 1.215 | 1.440 |
| FBgn0039914 | 0.925 | 0.751 | 0.785 | 0.880 | 0.967 | 1.659 |
| FBgn0034063 | 0.925 | 0.677 | 0.702 | 0.832 | 0.880 | 0.985 |
| FBgn0250907 | 0.925 | 1.060 | 1.163 | 1.326 | 1.397 | 2.400 |
| FBgn0000221 | 0.925 | 1.347 | 1.241 | 1.190 | 1.153 | 0.990 |
| FBgn0002891 | 0.925 | 1.013 | 1.133 | 1.020 | 1.529 | 2.332 |
| FBgn0038795 | 0.925 | 0.651 | 0.952 | 1.052 | 1.142 | 1.678 |
| FBgn0027584 | 0.925 | 0.626 | 1.276 | 1.111 | 1.796 | 3.857 |
| FBgn0027101 | 0.925 | 0.759 | 0.882 | 0.854 | 1.081 | 1.808 |
| FBgn0038639 | 0.924 | 0.789 | 0.937 | 0.971 | 1.033 | 1.262 |
| FBgn0039818 | 0.924 | 0.945 | 1.065 | 1.053 | 1.165 | 1.612 |
| FBgn0033945 | 0.924 | 0.562 | 1.265 | 1.385 | 1.693 | 2.809 |
| FBgn0032036 | 0.924 | 0.898 | 0.945 | 1.024 | 1.262 | 1.362 |
| FBgn0030164 | 0.924 | 0.746 | 0.993 | 1.037 | 1.204 | 1.498 |
| FBgn0023001 | 0.924 | 0.972 | 0.994 | 0.967 | 1.193 | 1.373 |
| FBgn0034967 | 0.923 | 0.603 | 0.897 | 0.927 | 1.192 | 1.513 |
| FBgn0015903 | 0.923 | 1.352 | 1.223 | 1.058 | 0.892 | 0.722 |
| FBgn0035964 | 0.923 | 1.026 | 0.981 | 1.164 | 1.208 | 1.355 |
| FBgn0037680 | 0.923 | 0.637 | 0.899 | 1.090 | 1.094 | 1.914 |
| FBgn0037922 | 0.923 | 1.131 | 1.111 | 1.143 | 0.938 | 0.737 |
| FBgn0030653 | 0.923 | 1.056 | 0.982 | 0.997 | 0.689 | 0.520 |
| FBgn0003353 | 0.923 | 0.696 | 0.966 | 0.921 | 1.177 | 1.545 |
| FBgn0033668 | 0.923 | 0.903 | 0.959 | 1.179 | 1.706 | 4.261 |
| FBgn0002707 | 0.923 | 1.053 | 1.027 | 1.083 | 1.163 | 1.550 |
| FBgn0038071 | 0.923 | 0.820 | 1.454 | 1.936 | 2.034 | 3.233 |
| FBgn0020416 | 0.923 | 0.545 | 0.778 | 0.760 | 0.958 | 1.255 |
| FBgn0260010 | 0.923 | 1.156 | 1.073 | 1.120 | 0.910 | 0.730 |
| FBgn0010314 | 0.922 | 1.334 | 1.098 | 1.002 | 0.376 | 0.083 |
| FBgn0016126 | 0.922 | 0.674 | 1.024 | 0.949 | 1.237 | 2.094 |
| FBgn0036765 | 0.922 | 0.775 | 0.942 | 1.099 | 1.403 | 1.592 |
| FBgn0040466 | 0.922 | 1.174 | 1.094 | 1.322 | 1.495 | 1.648 |
| FBgn0035084 | 0.922 | 0.649 | 0.715 | 1.072 | 1.009 | 1.587 |
| FBgn0085377 | 0.922 | 1.087 | 0.949 | 1.035 | 1.367 | 1.742 |
| FBgn0040732 | 0.922 | 0.530 | 0.603 | 0.820 | 0.910 | 1.093 |
| FBgn0038533 | 0.922 | 0.897 | 0.826 | 0.888 | 1.084 | 1.235 |
| FBgn0039654 | 0.922 | 1.095 | 1.014 | 0.922 | 0.843 | 0.738 |
| FBgn0036777 | 0.922 | 0.752 | 1.039 | 1.122 | 1.252 | 2.394 |
| FBgn0013467 | 0.921 | 0.837 | 0.997 | 0.946 | 1.104 | 1.446 |
| FBgn0031268 | 0.921 | 0.988 | 1.010 | 1.020 | 1.374 | 1.557 |
| FBgn0032987 | 0.921 | 0.809 | 0.860 | 1.055 | 1.103 | 1.277 |
| FBgn0034198 | 0.921 | 0.570 | 0.840 | 0.843 | 0.983 | 1.436 |
| FBgn0037252 | 0.921 | 1.033 | 1.052 | 0.950 | 0.813 | 0.744 |
| FBgn0033395 | 0.921 | 0.399 | 0.967 | 0.880 | 1.457 | 3.702 |
| FBgn0037092 | 0.921 | 0.894 | 0.982 | 0.917 | 1.175 | 1.691 |
| FBgn0037778 | 0.921 | 0.772 | 0.953 | 1.133 | 1.115 | 1.635 |
| FBgn0036587 | 0.921 | 0.524 | 1.131 | 0.836 | 1.696 | 3.112 |
| FBgn0031710 | 0.921 | 0.651 | 0.772 | 0.904 | 0.900 | 1.192 |
| FBgn0010350 | 0.920 | 1.070 | 0.984 | 0.895 | 0.798 | 0.695 |
| FBgn0026415 | 0.920 | 0.971 | 0.975 | 1.005 | 1.226 | 1.325 |
| FBgn0004028 | 0.920 | 0.991 | 1.011 | 1.135 | 1.109 | 1.422 |
| FBgn0029943 | 0.920 | 0.752 | 0.848 | 0.853 | 1.306 | 1.521 |
| FBgn0032601 | 0.920 | 0.983 | 1.242 | 1.160 | 1.465 | 1.852 |
| FBgn0001179 | 0.920 | 0.805 | 0.944 | 0.897 | 1.052 | 1.477 |
| FBgn0025391 | 0.920 | 0.947 | 1.075 | 0.972 | 1.324 | 1.884 |
| FBgn0053109 | 0.919 | 0.711 | 0.962 | 0.996 | 1.082 | 1.642 |
| FBgn0037896 | 0.919 | 0.709 | 0.991 | 1.038 | 1.141 | 1.961 |
| FBgn0020513 | 0.919 | 0.881 | 0.922 | 0.965 | 1.059 | 1.107 |
| FBgn0053144 | 0.919 | 1.172 | 1.103 | 0.967 | 0.896 | 0.772 |
| FBgn0029913 | 0.919 | 0.693 | 0.920 | 1.018 | 1.170 | 1.424 |
| FBgn0052179 | 0.919 | 0.852 | 0.869 | 1.040 | 1.070 | 1.214 |
| FBgn0050035 | 0.919 | 0.947 | 0.995 | 1.104 | 1.206 | 1.289 |
| FBgn0029950 | 0.919 | 0.514 | 0.777 | 1.006 | 1.179 | 1.513 |
| FBgn0039696 | 0.919 | 0.915 | 0.984 | 1.115 | 1.157 | 1.294 |
| FBgn0034804 | 0.919 | 1.048 | 1.073 | 1.223 | 1.229 | 1.382 |
| FBgn0034387 | 0.919 | 0.528 | 0.790 | 0.743 | 0.981 | 1.333 |
| FBgn0042180 | 0.918 | 1.273 | 1.414 | 1.285 | 1.120 | 0.812 |
| FBgn0024975 | 0.918 | 1.032 | 1.126 | 1.051 | 0.749 | 0.533 |
| FBgn0085470 | 0.918 | 1.305 | 1.230 | 1.136 | 0.919 | 0.821 |
| FBgn0250903 | 0.918 | 1.305 | 1.230 | 1.136 | 0.919 | 0.821 |
| FBgn0037383 | 0.918 | 0.960 | 1.025 | 0.965 | 0.707 | 0.548 |
| FBgn0030114 | 0.918 | 1.227 | 1.165 | 1.056 | 0.894 | 0.805 |
| FBgn0034888 | 0.918 | 0.571 | 0.830 | 0.886 | 1.059 | 1.349 |
| FBgn0050345 | 0.918 | 0.660 | 0.923 | 0.773 | 1.254 | 1.796 |
| FBgn0260026 | 0.918 | 1.084 | 1.054 | 0.829 | 0.735 | 0.578 |
| FBgn0260750 | 0.918 | 0.930 | 0.967 | 0.893 | 0.701 | 0.612 |
| FBgn0039008 | 0.917 | 0.882 | 0.990 | 1.177 | 1.282 | 1.457 |
| FBgn0052412 | 0.917 | 0.929 | 0.773 | 0.994 | 1.175 | 1.401 |
| FBgn0039336 | 0.917 | 0.892 | 1.003 | 1.152 | 1.231 | 1.397 |
| FBgn0052594 | 0.917 | 0.908 | 1.072 | 0.988 | 1.224 | 1.687 |
| FBgn0040099 | 0.917 | 0.582 | 0.728 | 0.892 | 0.874 | 1.579 |
| FBgn0035439 | 0.917 | 1.185 | 1.216 | 1.305 | 1.498 | 2.555 |
| FBgn0003884 | 0.917 | 1.097 | 1.182 | 1.040 | 0.719 | 0.557 |
| FBgn0019650 | 0.917 | 0.575 | 0.805 | 0.774 | 0.916 | 1.434 |
| FBgn0035158 | 0.917 | 0.796 | 0.896 | 0.842 | 1.078 | 1.697 |
| FBgn0052056 | 0.917 | 0.833 | 0.975 | 1.077 | 1.184 | 1.345 |
| FBgn0053096 | 0.917 | 1.035 | 1.131 | 0.951 | 0.808 | 0.669 |
| FBgn0030418 | 0.917 | 0.809 | 1.022 | 0.816 | 1.599 | 2.437 |
| FBgn0024733 | 0.917 | 0.697 | 1.017 | 1.065 | 1.341 | 1.665 |
| FBgn0032168 | 0.916 | 0.953 | 0.873 | 1.082 | 1.411 | 1.570 |
| FBgn0035425 | 0.916 | 1.095 | 1.040 | 1.085 | 0.943 | 0.728 |
| FBgn0039827 | 0.916 | 0.998 | 1.014 | 1.051 | 1.205 | 1.261 |
| FBgn0031886 | 0.916 | 1.105 | 1.086 | 1.079 | 0.527 | 0.241 |
| FBgn0033222 | 0.916 | 1.039 | 1.476 | 1.907 | 2.221 | 2.774 |
| FBgn0053508 | 0.916 | 0.514 | 1.205 | 1.150 | 1.677 | 4.222 |
| FBgn0052195 | 0.916 | 0.684 | 0.914 | 0.824 | 1.140 | 1.484 |
| FBgn0039386 | 0.916 | 0.593 | 1.100 | 1.333 | 1.451 | 2.272 |
| FBgn0011288 | 0.916 | 0.583 | 0.817 | 0.935 | 0.953 | 1.684 |
| FBgn0039196 | 0.916 | 0.828 | 0.957 | 0.808 | 1.412 | 2.454 |
| FBgn0038279 | 0.916 | 0.714 | 0.895 | 0.812 | 1.037 | 1.516 |
| FBgn0027500 | 0.915 | 1.729 | 1.291 | 1.043 | 0.793 | 0.277 |
| FBgn0261059 | 0.915 | 0.751 | 0.695 | 0.778 | 1.148 | 1.326 |
| FBgn0051098 | 0.915 | 0.919 | 0.993 | 0.960 | 1.087 | 1.199 |
| FBgn0032305 | 0.915 | 0.948 | 0.907 | 0.927 | 1.087 | 1.213 |
| FBgn0036881 | 0.915 | 0.926 | 1.173 | 0.933 | 1.820 | 2.768 |
| FBgn0030612 | 0.915 | 1.000 | 1.068 | 1.134 | 1.348 | 1.434 |
| FBgn0038079 | 0.915 | 0.752 | 1.125 | 1.396 | 1.413 | 2.173 |
| FBgn0260855 | 0.915 | 0.911 | 0.996 | 1.017 | 1.179 | 1.262 |
| FBgn0024248 | 0.915 | 0.738 | 0.887 | 0.832 | 1.028 | 1.244 |
| FBgn0030742 | 0.915 | 0.889 | 0.954 | 0.976 | 1.028 | 1.093 |
| FBgn0038680 | 0.915 | 0.662 | 0.855 | 0.844 | 1.167 | 1.367 |
| FBgn0034329 | 0.915 | 0.478 | 0.857 | 0.984 | 1.122 | 2.707 |
| FBgn0028426 | 0.915 | 0.835 | 1.111 | 0.991 | 1.399 | 1.829 |
| FBgn0031834 | 0.915 | 0.716 | 0.909 | 0.863 | 1.011 | 1.349 |
| FBgn0034403 | 0.915 | 1.206 | 1.126 | 0.925 | 0.489 | 0.321 |
| FBgn0030029 | 0.914 | 0.826 | 1.040 | 0.899 | 1.313 | 1.833 |
| FBgn0051778 | 0.914 | 0.511 | 0.666 | 0.884 | 0.849 | 1.304 |
| FBgn0053229 | 0.914 | 0.811 | 1.002 | 0.975 | 1.353 | 1.570 |
| FBgn0034889 | 0.914 | 0.981 | 0.910 | 0.964 | 1.310 | 1.521 |
| FBgn0032343 | 0.914 | 0.986 | 0.886 | 1.088 | 1.169 | 1.338 |
| FBgn0262719 | 0.914 | 1.088 | 1.179 | 1.089 | 1.387 | 1.802 |
| FBgn0083981 | 0.914 | 0.949 | 0.932 | 0.950 | 1.139 | 1.729 |
| FBgn0034963 | 0.914 | 0.729 | 0.736 | 1.120 | 1.030 | 1.740 |
| FBgn0025808 | 0.913 | 0.910 | 0.996 | 0.971 | 1.052 | 1.315 |
| FBgn0037794 | 0.913 | 0.751 | 0.925 | 0.952 | 1.078 | 1.943 |
| FBgn0261393 | 0.913 | 0.843 | 0.956 | 0.973 | 1.082 | 1.191 |
| FBgn0031602 | 0.913 | 1.169 | 1.053 | 1.118 | 1.345 | 1.719 |
| FBgn0034029 | 0.913 | 0.721 | 1.023 | 0.888 | 1.236 | 1.934 |
| FBgn0038926 | 0.913 | 1.022 | 0.908 | 1.014 | 1.190 | 1.799 |
| FBgn0002989 | 0.913 | 1.055 | 1.146 | 1.030 | 0.916 | 0.790 |
| FBgn0031468 | 0.912 | 0.995 | 1.038 | 1.267 | 1.217 | 1.903 |
| FBgn0036208 | 0.912 | 1.040 | 1.020 | 1.204 | 1.216 | 1.372 |
| FBgn0027563 | 0.912 | 0.911 | 0.969 | 0.962 | 1.130 | 1.210 |
| FBgn0030704 | 0.912 | 0.814 | 0.981 | 1.173 | 1.291 | 1.512 |
| FBgn0000083 | 0.912 | 1.106 | 1.092 | 1.168 | 1.260 | 1.304 |
| FBgn0037690 | 0.912 | 0.881 | 1.033 | 1.304 | 1.271 | 2.444 |
| FBgn0031260 | 0.912 | 1.034 | 0.988 | 1.045 | 1.075 | 1.165 |
| FBgn0025879 | 0.912 | 0.691 | 0.904 | 0.961 | 1.129 | 1.339 |
| FBgn0031304 | 0.912 | 0.968 | 1.045 | 0.897 | 0.823 | 0.706 |
| FBgn0031220 | 0.912 | 0.787 | 1.006 | 0.958 | 1.191 | 1.450 |
| FBgn0052475 | 0.912 | 0.702 | 0.991 | 1.231 | 1.196 | 1.995 |
| FBgn0027052 | 0.912 | 0.703 | 0.818 | 1.087 | 1.014 | 1.603 |
| FBgn0031598 | 0.911 | 0.744 | 0.910 | 0.888 | 1.027 | 1.224 |
| FBgn0051031 | 0.911 | 0.953 | 1.030 | 0.855 | 1.712 | 2.618 |
| FBgn0250862 | 0.911 | 1.024 | 0.856 | 0.977 | 1.410 | 2.707 |
| FBgn0030745 | 0.911 | 0.780 | 0.976 | 0.950 | 1.061 | 1.417 |
| FBgn0028996 | 0.911 | 0.686 | 1.008 | 0.915 | 1.191 | 1.715 |
| FBgn0030945 | 0.911 | 1.202 | 1.192 | 0.960 | 0.592 | 0.447 |
| FBgn0260991 | 0.911 | 1.719 | 1.325 | 1.065 | 0.435 | 0.074 |
| FBgn0000576 | 0.911 | 0.798 | 0.983 | 1.054 | 1.095 | 1.819 |
| FBgn0028645 | 0.910 | 0.731 | 0.812 | 0.813 | 1.324 | 3.151 |
| FBgn0032586 | 0.910 | 0.981 | 1.034 | 0.964 | 0.828 | 0.749 |
| FBgn0053514 | 0.910 | 0.644 | 0.812 | 0.806 | 1.061 | 1.221 |
| FBgn0038842 | 0.910 | 1.054 | 1.031 | 1.046 | 1.351 | 1.514 |
| FBgn0039920 | 0.910 | 0.716 | 1.130 | 1.066 | 1.335 | 2.537 |
| FBgn0046876 | 0.910 | 0.985 | 0.859 | 1.115 | 1.394 | 1.569 |
| FBgn0085268 | 0.910 | 1.000 | 0.978 | 0.920 | 0.935 | 0.754 |
| FBgn0052397 | 0.909 | 0.839 | 0.970 | 0.864 | 1.199 | 1.548 |
| FBgn0031629 | 0.909 | 1.116 | 1.105 | 1.228 | 1.202 | 1.493 |
| FBgn0034434 | 0.909 | 0.935 | 0.898 | 0.898 | 1.230 | 1.455 |
| FBgn0039489 | 0.909 | 0.698 | 0.842 | 0.945 | 1.046 | 2.182 |
| FBgn0259233 | 0.908 | 0.834 | 0.994 | 0.803 | 1.470 | 2.213 |
| FBgn0039483 | 0.908 | 0.497 | 0.933 | 0.842 | 1.136 | 1.851 |
| FBgn0004429 | 0.908 | 0.916 | 0.786 | 1.025 | 1.188 | 1.364 |
| FBgn0039609 | 0.908 | 0.591 | 0.853 | 0.863 | 1.216 | 1.449 |
| FBgn0051673 | 0.908 | 0.779 | 0.907 | 0.937 | 1.133 | 1.241 |
| FBgn0035941 | 0.908 | 0.854 | 0.848 | 1.232 | 1.644 | 1.849 |
| FBgn0031051 | 0.908 | 1.246 | 1.105 | 0.920 | 0.802 | 0.618 |
| FBgn0037960 | 0.908 | 0.448 | 0.777 | 0.840 | 1.012 | 1.389 |
| FBgn0032895 | 0.908 | 1.004 | 0.978 | 1.225 | 1.191 | 1.490 |
| FBgn0025624 | 0.908 | 0.829 | 0.815 | 0.997 | 1.246 | 1.349 |
| FBgn0032074 | 0.908 | 0.580 | 0.860 | 1.069 | 1.303 | 1.590 |
| FBgn0039411 | 0.908 | 0.853 | 1.192 | 0.994 | 1.483 | 2.494 |
| FBgn0023541 | 0.908 | 0.540 | 0.827 | 0.855 | 1.121 | 1.386 |
| FBgn0039924 | 0.908 | 0.639 | 0.957 | 1.146 | 1.207 | 1.694 |
| FBgn0024273 | 0.907 | 1.118 | 1.022 | 0.996 | 0.777 | 0.685 |
| FBgn0033067 | 0.907 | 0.850 | 0.981 | 1.202 | 1.144 | 1.939 |
| FBgn0032499 | 0.907 | 0.976 | 1.025 | 1.112 | 1.088 | 1.281 |
| FBgn0037755 | 0.907 | 0.859 | 0.901 | 0.822 | 1.132 | 1.513 |
| FBgn0085323 | 0.907 | 0.512 | 0.690 | 0.751 | 0.843 | 1.814 |
| FBgn0036501 | 0.907 | 0.605 | 0.836 | 0.813 | 1.016 | 1.262 |
| FBgn0037552 | 0.907 | 0.651 | 0.868 | 0.790 | 1.006 | 1.722 |
| FBgn0037081 | 0.906 | 0.883 | 0.967 | 0.957 | 1.000 | 1.210 |
| FBgn0040350 | 0.906 | 0.591 | 0.905 | 0.944 | 1.142 | 1.474 |
| FBgn0039740 | 0.906 | 1.163 | 1.054 | 1.040 | 0.927 | 0.832 |
| FBgn0034109 | 0.906 | 0.658 | 0.760 | 0.852 | 1.134 | 1.240 |
| FBgn0046689 | 0.906 | 1.187 | 1.006 | 1.310 | 1.368 | 1.749 |
| FBgn0051103 | 0.906 | 0.798 | 1.065 | 1.303 | 1.404 | 1.764 |
| FBgn0039299 | 0.906 | 0.321 | 0.545 | 0.618 | 0.640 | 1.251 |
| FBgn0011829 | 0.906 | 0.931 | 1.188 | 1.308 | 1.794 | 4.754 |
| FBgn0033769 | 0.906 | 0.766 | 0.892 | 0.981 | 1.101 | 1.222 |
| FBgn0028983 | 0.906 | 1.094 | 1.064 | 1.233 | 1.340 | 1.430 |
| FBgn0052576 | 0.906 | 0.770 | 1.127 | 1.445 | 1.512 | 2.072 |
| FBgn0026479 | 0.905 | 1.018 | 1.060 | 0.936 | 0.722 | 0.638 |
| FBgn0034500 | 0.905 | 0.907 | 0.995 | 1.176 | 1.323 | 1.451 |
| FBgn0001086 | 0.905 | 1.326 | 1.333 | 1.142 | 0.362 | 0.100 |
| FBgn0014366 | 0.905 | 1.174 | 1.108 | 1.086 | 0.887 | 0.814 |
| FBgn0035812 | 0.905 | 1.135 | 1.137 | 1.044 | 1.537 | 2.306 |
| FBgn0260006 | 0.905 | 1.010 | 1.038 | 1.004 | 1.125 | 1.366 |
| FBgn0031307 | 0.905 | 0.958 | 0.952 | 0.994 | 1.707 | 2.013 |
| FBgn0004580 | 0.905 | 0.787 | 0.952 | 0.888 | 1.044 | 1.502 |
| FBgn0034878 | 0.905 | 1.084 | 1.040 | 1.057 | 0.738 | 0.592 |
| FBgn0037935 | 0.905 | 0.482 | 0.749 | 0.684 | 0.976 | 1.272 |
| FBgn0028394 | 0.905 | 0.883 | 0.951 | 1.051 | 1.131 | 1.215 |
| FBgn0032390 | 0.905 | 1.128 | 1.190 | 1.599 | 1.494 | 2.141 |
| FBgn0036725 | 0.904 | 0.874 | 0.846 | 0.803 | 1.128 | 1.539 |
| FBgn0027375 | 0.904 | 0.876 | 1.004 | 0.899 | 1.174 | 1.675 |
| FBgn0030263 | 0.904 | 1.050 | 1.050 | 1.547 | 1.713 | 2.021 |
| FBgn0031093 | 0.904 | 1.108 | 1.071 | 0.953 | 0.641 | 0.543 |
| FBgn0085360 | 0.904 | 0.878 | 0.767 | 1.095 | 1.075 | 1.472 |
| FBgn0016047 | 0.904 | 0.893 | 1.274 | 1.226 | 1.677 | 2.039 |
| FBgn0038916 | 0.904 | 0.947 | 0.938 | 1.210 | 1.162 | 1.499 |
| FBgn0054051 | 0.904 | 0.532 | 0.647 | 0.728 | 0.714 | 1.006 |
| FBgn0028938 | 0.903 | 0.729 | 1.087 | 0.688 | 1.871 | 3.380 |
| FBgn0026878 | 0.903 | 0.847 | 0.736 | 0.964 | 1.074 | 1.234 |
| FBgn0033850 | 0.903 | 1.172 | 1.066 | 1.105 | 1.367 | 1.674 |
| FBgn0034715 | 0.903 | 0.689 | 1.028 | 1.375 | 1.696 | 2.056 |
| FBgn0011701 | 0.903 | 1.111 | 1.141 | 1.108 | 1.349 | 1.489 |
| FBgn0259966 | 0.903 | 0.454 | 1.320 | 1.372 | 1.645 | 3.143 |
| FBgn0001986 | 0.903 | 1.068 | 1.013 | 1.026 | 0.697 | 0.559 |
| FBgn0039051 | 0.903 | 0.850 | 0.871 | 1.039 | 1.220 | 1.307 |
| FBgn0026077 | 0.903 | 0.916 | 0.920 | 0.884 | 1.094 | 1.536 |
| FBgn0022981 | 0.903 | 1.257 | 1.471 | 1.173 | 0.468 | 0.143 |
| FBgn0085797 | 0.903 | 0.719 | 1.837 | 1.905 | 2.356 | 6.287 |
| FBgn0038076 | 0.903 | 0.477 | 0.881 | 0.850 | 1.037 | 2.176 |
| FBgn0036838 | 0.903 | 1.148 | 1.109 | 1.152 | 1.328 | 2.029 |
| FBgn0261429 | 0.903 | 0.661 | 0.933 | 1.163 | 1.296 | 3.513 |
| FBgn0003117 | 0.903 | 0.846 | 1.128 | 0.914 | 1.469 | 2.151 |
| FBgn0020261 | 0.902 | 1.144 | 1.093 | 0.878 | 0.662 | 0.544 |
| FBgn0004569 | 0.902 | 0.980 | 1.104 | 0.980 | 1.504 | 1.887 |
| FBgn0004513 | 0.902 | 0.596 | 0.870 | 0.777 | 1.013 | 1.446 |
| FBgn0043796 | 0.902 | 1.322 | 1.189 | 1.132 | 0.992 | 0.881 |
| FBgn0029161 | 0.902 | 1.073 | 0.946 | 0.992 | 0.870 | 0.682 |
| FBgn0085208 | 0.902 | 1.073 | 0.946 | 0.992 | 0.870 | 0.682 |
| FBgn0039544 | 0.902 | 1.152 | 1.094 | 1.090 | 1.321 | 1.631 |
| FBgn0028699 | 0.902 | 0.943 | 0.891 | 0.951 | 1.233 | 1.357 |
| FBgn0050438 | 0.902 | 0.506 | 0.863 | 0.689 | 1.076 | 2.008 |
| FBgn0020388 | 0.902 | 0.999 | 1.004 | 1.107 | 1.564 | 1.709 |
| FBgn0037743 | 0.902 | 0.757 | 0.909 | 0.838 | 1.058 | 1.265 |
| FBgn0039874 | 0.902 | 0.682 | 0.961 | 0.897 | 1.071 | 1.552 |
| FBgn0030008 | 0.901 | 1.467 | 1.236 | 1.206 | 0.992 | 0.793 |
| FBgn0261564 | 0.901 | 0.996 | 1.048 | 0.929 | 0.919 | 0.800 |
| FBgn0038930 | 0.901 | 0.985 | 0.930 | 0.990 | 1.132 | 1.210 |
| FBgn0033133 | 0.901 | 0.724 | 0.677 | 0.862 | 0.835 | 1.079 |
| FBgn0003326 | 0.901 | 1.121 | 0.993 | 1.152 | 1.649 | 1.869 |
| FBgn0260946 | 0.901 | 1.079 | 1.012 | 1.051 | 1.182 | 1.314 |
| FBgn0037574 | 0.900 | 1.223 | 1.113 | 1.079 | 0.853 | 0.762 |
| FBgn0037039 | 0.900 | 0.656 | 0.769 | 1.014 | 0.953 | 1.372 |
| FBgn0029167 | 0.900 | 0.712 | 1.068 | 0.967 | 1.565 | 1.935 |
| FBgn0034381 | 0.900 | 1.039 | 1.075 | 1.316 | 1.687 | 1.817 |

| Supplemental Table 1C. Values of R Squared and Fold Changes for Genes Found to Behave Linearly at Day 20 Post-Irradiation. (Analysis with all data included.) | | | | | | |
| --- | --- | --- | --- | --- | --- | --- |
|  |  |  |  |  |  |  |
| Flybase ID | R Squared Values | 10R Fold Change | 1000R Fold Change | 5000R Fold Change | 10000R Fold Change | 20000R Fold Change |
| FBgn0263029 | 1.000 | 0.737 | 0.751 | 0.824 | 0.907 | 1.075 |
| FBgn0040972 | 0.999 | 0.761 | 1.042 | 2.190 | 3.282 | 5.736 |
| FBgn0004797 | 0.998 | 0.905 | 0.933 | 1.025 | 1.195 | 1.473 |
| FBgn0004624 | 0.997 | 1.071 | 1.202 | 1.844 | 2.685 | 4.001 |
| FBgn0052939 | 0.996 | 1.096 | 1.180 | 1.382 | 1.724 | 2.216 |
| FBgn0036290 | 0.995 | 1.324 | 1.438 | 1.834 | 2.689 | 4.152 |
| FBgn0025583 | 0.995 | 0.958 | 1.192 | 1.835 | 2.344 | 3.712 |
| FBgn0010038 | 0.994 | 0.497 | 0.766 | 1.195 | 1.706 | 3.165 |
| FBgn0030189 | 0.993 | 1.131 | 1.168 | 1.303 | 1.424 | 1.817 |
| FBgn0040056 | 0.993 | NA | 1.478 | 1.632 | 1.820 | 2.101 |
| FBgn0024989 | 0.993 | 0.553 | 0.561 | 0.587 | 0.655 | 0.750 |
| FBgn0040837 | 0.992 | 1.353 | 1.559 | 1.942 | 2.409 | 3.207 |
| FBgn0002533 | 0.992 | 0.574 | 0.556 | 0.511 | 0.391 | 0.190 |
| FBgn0086604 | 0.992 | 1.084 | 1.071 | 1.153 | 1.217 | 1.390 |
| FBgn0032470 | 0.991 | 0.911 | 1.023 | 1.379 | 1.656 | 2.674 |
| FBgn0034389 | 0.991 | 1.123 | 1.109 | 1.206 | 1.367 | 1.594 |
| FBgn0041627 | 0.989 | 1.086 | 1.148 | 1.313 | 1.730 | 2.554 |
| FBgn0024912 | 0.989 | 1.184 | 1.400 | 1.985 | 2.959 | 4.117 |
| FBgn0037756 | 0.988 | 0.970 | 0.972 | 1.033 | 1.174 | 1.341 |
| FBgn0260431 | 0.988 | 1.208 | 1.163 | 1.351 | 1.688 | 2.237 |
| FBgn0034199 | 0.988 | 1.054 | 1.093 | 1.374 | 1.582 | 1.989 |
| FBgn0052029 | 0.987 | 0.998 | 1.145 | 1.368 | 1.855 | 2.407 |
| FBgn0036681 | 0.987 | 0.308 | 0.282 | 0.371 | 0.516 | 0.725 |
| FBgn0033816 | 0.987 | 0.988 | 0.977 | 1.067 | 1.200 | 1.358 |
| FBgn0037011 | 0.985 | 1.200 | 1.247 | 1.377 | 1.768 | 2.182 |
| FBgn0051075 | 0.985 | 0.940 | 0.944 | 0.918 | 0.851 | 0.752 |
| FBgn0052640 | 0.984 | 1.346 | 1.621 | 1.936 | 2.795 | 3.720 |
| FBgn0030260 | 0.984 | 0.427 | 0.481 | 0.508 | 0.585 | 0.757 |
| FBgn0033327 | 0.984 | 0.863 | 0.835 | 0.677 | 0.618 | 0.282 |
| FBgn0052641 | 0.984 | 1.356 | 1.631 | 1.979 | 2.844 | 3.760 |
| FBgn0262524 | 0.983 | 0.920 | 1.032 | 1.371 | 1.607 | 2.124 |
| FBgn0051272 | 0.983 | 0.966 | 0.973 | 1.005 | 1.127 | 1.278 |
| FBgn0032597 | 0.983 | 1.130 | 1.165 | 1.344 | 1.714 | 2.078 |
| FBgn0031906 | 0.983 | 0.426 | 0.487 | 0.596 | 0.663 | 0.880 |
| FBgn0033875 | 0.983 | 0.929 | 1.213 | 1.564 | 2.179 | 2.948 |
| FBgn0034184 | 0.983 | 0.949 | 0.948 | 1.018 | 1.052 | 1.222 |
| FBgn0015351 | 0.982 | 0.930 | 1.171 | 1.332 | 1.589 | 2.294 |
| FBgn0038455 | 0.982 | 0.930 | 1.171 | 1.332 | 1.589 | 2.294 |
| FBgn0039544 | 0.981 | 1.200 | 1.191 | 1.243 | 1.365 | 1.497 |
| FBgn0023514 | 0.981 | 1.168 | 1.207 | 1.274 | 1.341 | 1.457 |
| FBgn0033395 | 0.981 | 1.664 | 2.672 | 2.987 | 4.758 | 7.566 |
| FBgn0011509 | 0.981 | 1.038 | 1.179 | 1.230 | 1.497 | 1.865 |
| FBgn0031653 | 0.981 | 0.979 | 0.872 | 0.795 | 0.653 | 0.410 |
| FBgn0046763 | 0.981 | 1.292 | 1.436 | 1.584 | 1.813 | 2.153 |
| FBgn0025807 | 0.980 | 1.056 | 1.065 | 1.121 | 1.167 | 1.363 |
| FBgn0039342 | 0.979 | 1.028 | 1.103 | 1.585 | 2.318 | 3.043 |
| FBgn0003525 | 0.979 | 0.892 | 0.974 | 1.112 | 1.370 | 1.633 |
| FBgn0043806 | 0.979 | 0.872 | 1.000 | 1.049 | 1.353 | 1.704 |
| FBgn0052985 | 0.978 | 0.684 | 0.848 | 0.889 | 1.165 | 1.570 |
| FBgn0036527 | 0.977 | NA | 1.584 | 1.729 | 1.960 | 2.704 |
| FBgn0039942 | 0.977 | 0.611 | 0.613 | 0.733 | 0.838 | 0.979 |
| FBgn0036992 | 0.977 | 1.063 | 0.977 | 0.916 | 0.834 | 0.639 |
| FBgn0051864 | 0.976 | 1.061 | 1.066 | 1.287 | 2.116 | 2.943 |
| FBgn0023000 | 0.976 | 0.948 | 1.167 | 1.204 | 1.648 | 2.278 |
| FBgn0039555 | 0.976 | 0.965 | 0.967 | 0.952 | 0.934 | 0.877 |
| FBgn0037850 | 0.975 | 1.568 | 1.411 | 1.769 | 2.408 | 3.722 |
| FBgn0038295 | 0.974 | 0.793 | 0.840 | 0.870 | 0.929 | 1.021 |
| FBgn0052475 | 0.974 | 0.638 | 0.836 | 0.872 | 1.198 | 1.657 |
| FBgn0036126 | 0.974 | 0.821 | 1.341 | 2.107 | 2.679 | 4.026 |
| FBgn0037719 | 0.973 | 1.089 | 1.205 | 1.262 | 1.378 | 1.764 |
| FBgn0036908 | 0.972 | 0.792 | 0.966 | 1.030 | 1.196 | 1.589 |
| FBgn0043576 | 0.972 | 0.852 | 0.804 | 0.666 | 0.626 | 0.257 |
| FBgn0033261 | 0.972 | 1.061 | 1.306 | 1.320 | 1.945 | 2.753 |
| FBgn0024913 | 0.971 | 1.407 | 1.339 | 1.757 | 1.967 | 3.099 |
| FBgn0034512 | 0.971 | 0.941 | 1.267 | 1.347 | 1.709 | 2.406 |
| FBgn0037943 | 0.971 | 1.056 | 1.089 | 1.119 | 1.145 | 1.277 |
| FBgn0032879 | 0.970 | 1.016 | 1.237 | 1.253 | 1.801 | 2.430 |
| FBgn0261429 | 0.970 | 0.887 | 1.048 | 1.079 | 1.849 | 2.800 |
| FBgn0037291 | 0.970 | 0.971 | 1.045 | 0.895 | 0.605 | 0.291 |
| FBgn0030029 | 0.969 | 1.060 | 1.040 | 1.216 | 1.306 | 1.494 |
| FBgn0261387 | 0.968 | 0.913 | 0.971 | 1.238 | 1.591 | 1.917 |
| FBgn0033702 | 0.968 | 0.585 | 0.648 | 0.775 | 0.892 | 1.061 |
| FBgn0087039 | 0.968 | 0.974 | 1.304 | 1.819 | 2.216 | 3.010 |
| FBgn0020660 | 0.967 | 0.824 | 1.097 | 1.312 | 1.729 | 2.223 |
| FBgn0032819 | 0.967 | 1.057 | 1.084 | 1.310 | 1.353 | 1.679 |
| FBgn0031065 | 0.967 | 0.643 | 0.621 | 0.662 | 0.749 | 0.840 |
| FBgn0054002 | 0.966 | 1.164 | 1.265 | 1.325 | 1.526 | 1.714 |
| FBgn0039132 | 0.966 | 1.331 | 1.702 | 1.869 | 2.256 | 2.904 |
| FBgn0025808 | 0.966 | 1.391 | 1.306 | 1.212 | 1.171 | 0.872 |
| FBgn0033928 | 0.965 | 1.548 | 1.694 | 1.700 | 2.473 | 3.460 |
| FBgn0037018 | 0.965 | 0.901 | 0.924 | 0.866 | 0.824 | 0.754 |
| FBgn0037322 | 0.965 | 0.177 | 0.198 | 0.212 | 0.344 | 0.605 |
| FBgn0038660 | 0.965 | 0.772 | 0.984 | 1.030 | 1.267 | 1.646 |
| FBgn0040637 | 0.964 | 1.457 | 1.511 | 2.097 | NA | 14.334 |
| FBgn0037481 | 0.964 | 2.274 | 2.300 | 2.342 | 2.485 | 2.588 |
| FBgn0037100 | 0.963 | 0.681 | 0.766 | 0.885 | 1.308 | 1.608 |
| FBgn0040099 | 0.963 | 2.094 | 2.137 | 2.472 | 3.254 | 3.804 |
| FBgn0087002 | 0.963 | 1.292 | 1.180 | 1.842 | 3.254 | 4.312 |
| FBgn0015037 | 0.963 | 1.606 | 2.247 | 2.401 | 3.224 | 4.294 |
| FBgn0037309 | 0.963 | 1.367 | 1.414 | 1.440 | 1.862 | 2.557 |
| FBgn0039481 | 0.963 | 0.719 | 0.688 | 0.818 | 0.923 | 1.054 |
| FBgn0035779 | 0.963 | 1.074 | 1.020 | 1.094 | 1.272 | 1.492 |
| FBgn0036232 | 0.962 | 1.443 | 1.609 | 1.905 | 2.160 | 2.552 |
| FBgn0004580 | 0.962 | 1.088 | 1.104 | 1.162 | 1.234 | 1.553 |
| FBgn0028394 | 0.962 | 1.260 | 1.317 | 1.445 | 1.478 | 1.682 |
| FBgn0039396 | 0.962 | 3.966 | 4.122 | 4.425 | 4.982 | 7.099 |
| FBgn0040754 | 0.962 | 1.010 | 1.005 | 1.044 | 1.172 | 1.266 |
| FBgn0026576 | 0.961 | 0.788 | 0.723 | 0.819 | 1.008 | 1.222 |
| FBgn0261613 | 0.961 | NA | 1.714 | 1.841 | 2.438 | 4.048 |
| FBgn0051036 | 0.961 | 0.807 | 0.921 | 0.957 | 1.452 | 1.848 |
| FBgn0001202 | 0.961 | 1.185 | 1.265 | 1.337 | 1.474 | 1.612 |
| FBgn0263093 | 0.960 | 3.000 | 3.478 | 3.536 | 6.177 | 8.585 |
| FBgn0039616 | 0.960 | 0.956 | 1.071 | 1.186 | 1.260 | 1.799 |
| FBgn0050281 | 0.960 | 1.172 | 1.190 | 1.415 | 1.633 | 1.853 |
| FBgn0037731 | 0.960 | 1.029 | 1.164 | 1.338 | 1.787 | 2.110 |
| FBgn0262442 | 0.960 | 1.364 | 1.345 | 1.110 | 0.670 | 0.362 |
| FBgn0033134 | 0.960 | 1.279 | 1.832 | 2.253 | 3.542 | 4.562 |
| FBgn0032393 | 0.960 | 0.953 | 1.020 | 1.398 | 2.248 | 2.817 |
| FBgn0022774 | 0.959 | 0.998 | 1.040 | 0.933 | 0.895 | 0.744 |
| FBgn0034726 | 0.959 | 0.908 | 0.967 | 1.211 | 1.327 | 1.594 |
| FBgn0011774 | 0.959 | 1.034 | 1.096 | 1.113 | 1.871 | 2.722 |
| FBgn0031307 | 0.959 | 1.351 | 1.214 | 1.543 | 2.492 | 3.248 |
| FBgn0026570 | 0.959 | 0.980 | 1.202 | 1.265 | 1.692 | 2.069 |
| FBgn0035440 | 0.958 | 1.101 | 1.225 | 1.523 | 1.682 | 2.045 |
| FBgn0016920 | 0.958 | 0.485 | 0.603 | 0.595 | 0.797 | 1.033 |
| FBgn0037020 | 0.958 | 0.978 | 0.996 | 0.993 | 1.100 | 1.229 |
| FBgn0041233 | 0.958 | 0.433 | 0.540 | 0.556 | 0.689 | 0.859 |
| FBgn0027914 | 0.957 | 1.279 | 1.327 | 1.159 | 1.101 | 0.915 |
| FBgn0259236 | 0.957 | 0.732 | 0.780 | 0.807 | 0.908 | 0.988 |
| FBgn0015524 | 0.956 | 2.656 | 2.620 | 2.953 | 3.117 | 3.423 |
| FBgn0022800 | 0.956 | 1.128 | 1.182 | 1.167 | 1.331 | 1.566 |
| FBgn0032198 | 0.956 | 1.069 | 1.103 | 1.124 | 1.222 | 1.291 |
| FBgn0035816 | 0.956 | 0.709 | 0.783 | 0.787 | 0.856 | 1.008 |
| FBgn0259233 | 0.956 | 1.666 | 1.916 | 1.909 | 2.863 | 3.667 |
| FBgn0003356 | 0.956 | 0.991 | 0.967 | 0.963 | 0.828 | 0.721 |
| FBgn0028938 | 0.956 | 1.186 | 1.358 | 1.307 | 2.020 | 3.024 |
| FBgn0032505 | 0.956 | 1.016 | 1.136 | 1.172 | 1.270 | 1.466 |
| FBgn0051104 | 0.955 | 0.776 | 0.976 | 0.997 | 1.416 | 1.770 |
| FBgn0013305 | 0.955 | 0.821 | 0.829 | 0.965 | 1.078 | 1.199 |
| FBgn0032946 | 0.955 | 1.353 | 1.321 | 1.522 | 1.546 | 1.974 |
| FBgn0016126 | 0.954 | 0.813 | 1.401 | 1.555 | 2.394 | 3.246 |
| FBgn0031473 | 0.954 | 1.329 | 1.351 | 1.374 | 1.619 | 1.799 |
| FBgn0040001 | 0.954 | 1.145 | 1.069 | 1.430 | 2.226 | 2.755 |
| FBgn0058045 | 0.954 | 1.245 | 1.516 | 1.849 | 2.347 | 2.823 |
| FBgn0037518 | 0.953 | 1.150 | 1.181 | 1.190 | 1.322 | 1.641 |
| FBgn0025879 | 0.953 | 1.010 | 1.100 | 1.179 | 1.534 | 1.757 |
| FBgn0053301 | 0.953 | 1.265 | 1.310 | 1.309 | 1.664 | 1.982 |
| FBgn0038472 | 0.952 | 0.701 | 0.788 | 0.773 | 0.906 | 1.084 |
| FBgn0028853 | 0.952 | 0.790 | 1.078 | 1.351 | 1.777 | 2.224 |
| FBgn0261871 | 0.952 | 0.953 | 1.063 | 1.118 | 1.223 | 1.382 |
| FBgn0015582 | 0.952 | 1.095 | 1.199 | 1.211 | 1.440 | 1.620 |
| FBgn0035998 | 0.951 | 1.027 | 1.217 | 1.521 | 1.923 | 2.292 |
| FBgn0011591 | 0.951 | 0.830 | 0.871 | 1.046 | 1.338 | 1.530 |
| FBgn0030038 | 0.951 | 1.051 | 1.010 | 0.957 | 0.892 | 0.822 |
| FBgn0030716 | 0.951 | 1.122 | 1.058 | 1.181 | 1.304 | 1.444 |
| FBgn0028411 | 0.950 | 2.203 | 2.019 | 2.891 | 3.009 | 4.087 |
| FBgn0030795 | 0.950 | 1.116 | 1.159 | 1.147 | 1.482 | 1.798 |
| FBgn0086683 | 0.950 | 1.264 | 1.430 | 1.595 | 1.680 | 2.009 |
| FBgn0051769 | 0.950 | 1.180 | 1.228 | 1.276 | 1.293 | 1.504 |
| FBgn0037906 | 0.949 | 1.177 | 1.156 | 1.156 | 1.054 | 0.836 |
| FBgn0035166 | 0.949 | 0.802 | 0.745 | 0.892 | 0.939 | 1.297 |
| FBgn0034029 | 0.949 | 1.041 | 1.188 | 1.234 | 1.842 | 2.237 |
| FBgn0038082 | 0.949 | 0.925 | 1.273 | 1.377 | 2.556 | 3.322 |
| FBgn0038893 | 0.949 | 1.054 | 1.453 | 1.398 | 2.536 | 3.456 |
| FBgn0039202 | 0.948 | 2.686 | 2.456 | 3.627 | 3.805 | 5.141 |
| FBgn0034530 | 0.948 | 0.746 | 0.757 | 0.823 | 1.192 | 1.421 |
| FBgn0034647 | 0.948 | 1.489 | 1.497 | 1.411 | 1.279 | 0.739 |
| FBgn0038304 | 0.947 | 1.215 | 1.213 | 1.102 | 0.943 | 0.840 |
| FBgn0037141 | 0.946 | 1.396 | 1.490 | 1.440 | 1.987 | 2.818 |
| FBgn0032032 | 0.946 | 0.981 | 1.132 | 1.216 | 1.267 | 1.615 |
| FBgn0003495 | 0.946 | 0.871 | 0.925 | 0.914 | 1.135 | 1.602 |
| FBgn0033453 | 0.945 | 0.761 | 1.092 | 1.799 | 2.498 | 3.213 |
| FBgn0085285 | 0.945 | 0.728 | 1.074 | 1.148 | 1.769 | 2.246 |
| FBgn0039970 | 0.945 | 1.522 | 1.098 | 1.834 | 2.174 | 3.265 |
| FBgn0032613 | 0.945 | 1.350 | 1.413 | 1.416 | 2.174 | 2.762 |
| FBgn0020416 | 0.945 | 0.968 | 1.304 | 1.290 | 1.732 | 2.223 |
| FBgn0039647 | 0.945 | 0.964 | 0.986 | 0.977 | 1.194 | 1.384 |
| FBgn0039696 | 0.945 | 0.898 | 0.856 | 0.938 | 1.124 | 1.251 |
| FBgn0000008 | 0.944 | 0.913 | 0.871 | 0.837 | 0.828 | 0.723 |
| FBgn0031710 | 0.944 | 1.059 | 1.211 | 1.238 | 1.327 | 1.611 |
| FBgn0023546 | 0.944 | 0.626 | 0.542 | 0.606 | 0.931 | 1.405 |
| FBgn0260432 | 0.944 | 0.626 | 0.542 | 0.606 | 0.931 | 1.405 |
| FBgn0037076 | 0.944 | 1.051 | 1.320 | 1.791 | 1.788 | 3.083 |
| FBgn0032781 | 0.944 | 1.113 | 1.019 | 1.244 | 1.329 | 1.566 |
| FBgn0050026 | 0.944 | 1.143 | 1.169 | 1.259 | 1.553 | 2.557 |
| FBgn0010173 | 0.944 | 1.079 | 1.309 | 1.238 | 1.770 | 2.273 |
| FBgn0030314 | 0.943 | 0.796 | 0.802 | 0.769 | 0.768 | 0.717 |
| FBgn0032785 | 0.943 | 1.312 | 1.349 | 1.611 | 1.792 | 2.006 |
| FBgn0053158 | 0.942 | 0.953 | 1.108 | 1.056 | 1.306 | 1.746 |
| FBgn0014143 | 0.942 | 1.269 | 1.163 | 1.056 | 1.029 | 0.792 |
| FBgn0261989 | 0.942 | 0.963 | 1.963 | 2.859 | 5.799 | 7.485 |
| FBgn0039421 | 0.941 | 1.621 | 1.543 | 1.174 | 0.514 | 0.144 |
| FBgn0039153 | 0.941 | 1.310 | 1.556 | 1.554 | 1.916 | 2.247 |
| FBgn0052000 | 0.941 | 1.003 | 1.575 | 1.782 | 2.853 | 3.597 |
| FBgn0016919 | 0.941 | 1.276 | 1.460 | 1.391 | 1.892 | 2.339 |
| FBgn0025741 | 0.940 | 1.067 | 0.855 | 1.623 | 2.282 | 2.884 |
| FBgn0003687 | 0.940 | 0.865 | 0.862 | 0.957 | 1.010 | 1.082 |
| FBgn0037249 | 0.940 | 0.945 | 0.906 | 0.911 | 0.874 | 0.803 |
| FBgn0033130 | 0.940 | 0.976 | 1.304 | 1.352 | 1.796 | 2.199 |
| FBgn0020647 | 0.940 | 0.901 | 0.860 | 0.877 | 0.779 | 0.682 |
| FBgn0029664 | 0.940 | 1.243 | 1.441 | 1.489 | 1.631 | 1.906 |
| FBgn0262057 | 0.940 | 1.028 | 1.112 | 1.109 | 1.265 | 1.385 |
| FBgn0037105 | 0.940 | 1.226 | 1.351 | 1.295 | 1.849 | 2.309 |
| FBgn0034454 | 0.940 | 1.022 | 1.161 | 1.573 | 1.585 | 2.178 |
| FBgn0040211 | 0.940 | 0.924 | 0.872 | 0.834 | 0.823 | 0.649 |
| FBgn0037217 | 0.939 | 1.546 | 1.850 | 1.746 | 2.202 | 2.905 |
| FBgn0039958 | 0.939 | 0.959 | 1.161 | 1.097 | 1.609 | 2.035 |
| FBgn0039052 | 0.939 | 0.935 | 1.181 | 1.410 | 2.469 | 3.014 |
| FBgn0260795 | 0.939 | 1.165 | 1.252 | 1.208 | 1.697 | 2.100 |
| FBgn0031001 | 0.939 | 0.739 | 0.792 | 0.625 | 0.578 | 0.420 |
| FBgn0052082 | 0.938 | 1.279 | 1.266 | 1.292 | 1.570 | 1.760 |
| FBgn0033153 | 0.938 | 1.066 | 1.249 | 1.641 | 1.785 | 2.227 |
| FBgn0040697 | 0.938 | 0.819 | 0.804 | 0.930 | 1.012 | 1.103 |
| FBgn0010240 | 0.937 | 0.879 | 0.849 | 0.949 | 0.971 | 1.260 |
| FBgn0051778 | 0.937 | 1.380 | 1.299 | 1.486 | 2.368 | 2.893 |
| FBgn0014396 | 0.937 | 1.224 | 1.202 | 1.330 | 1.635 | 1.793 |
| FBgn0031216 | 0.937 | 0.889 | 1.368 | 1.355 | 1.968 | 2.586 |
| FBgn0035805 | 0.937 | 1.173 | 1.217 | 1.530 | 2.090 | 2.386 |
| FBgn0035165 | 0.937 | 1.005 | 0.974 | 1.099 | 1.115 | 1.249 |
| FBgn0036536 | 0.937 | 0.759 | 0.808 | 0.672 | 0.436 | 0.296 |
| FBgn0037818 | 0.937 | 1.173 | 1.481 | 1.463 | 2.116 | 2.573 |
| FBgn0039597 | 0.937 | 1.019 | 1.029 | 1.246 | 1.228 | 1.561 |
| FBgn0029708 | 0.936 | 0.939 | 0.922 | 0.960 | 1.161 | 1.281 |
| FBgn0038795 | 0.936 | 0.966 | 1.305 | 1.502 | 2.317 | 2.784 |
| FBgn0010348 | 0.936 | 1.104 | 1.132 | 1.179 | 1.417 | 1.534 |
| FBgn0259711 | 0.936 | 1.177 | 1.481 | 1.338 | 2.073 | 2.810 |
| FBgn0052099 | 0.936 | 1.336 | 1.358 | 1.336 | 1.559 | 1.770 |
| FBgn0031418 | 0.936 | 0.949 | 0.986 | 0.943 | 0.898 | 0.817 |
| FBgn0085442 | 0.936 | 1.104 | 1.105 | 1.128 | 1.320 | 1.430 |
| FBgn0032230 | 0.935 | 1.283 | 1.324 | 1.529 | 1.998 | 2.224 |
| FBgn0261588 | 0.935 | 1.136 | 1.092 | 1.016 | 0.898 | 0.822 |
| FBgn0052483 | 0.935 | 1.037 | 0.933 | 1.152 | 1.203 | 1.701 |
| FBgn0003423 | 0.935 | 0.896 | 0.948 | 0.871 | 0.804 | 0.713 |
| FBgn0028546 | 0.935 | 1.077 | 1.310 | 1.397 | 1.858 | 2.146 |
| FBgn0037690 | 0.935 | 2.986 | 2.813 | 3.917 | 4.385 | 5.221 |
| FBgn0032801 | 0.935 | 1.034 | 1.001 | 1.083 | 1.220 | 1.302 |
| FBgn0026084 | 0.935 | 1.031 | 1.003 | 1.044 | 1.072 | 1.193 |
| FBgn0038451 | 0.935 | 0.445 | 0.535 | 0.704 | 0.920 | 1.081 |
| FBgn0034312 | 0.934 | 1.385 | 1.357 | 1.582 | 1.564 | 1.938 |
| FBgn0067779 | 0.934 | 1.574 | 1.751 | 1.637 | 2.156 | 2.948 |
| FBgn0014859 | 0.934 | 0.825 | 0.866 | 0.895 | 1.352 | 1.609 |
| FBgn0034958 | 0.934 | 0.903 | 0.989 | 0.995 | 1.156 | 1.263 |
| FBgn0037622 | 0.933 | 0.832 | 0.929 | 0.945 | 1.055 | 1.165 |
| FBgn0038953 | 0.933 | 1.048 | 1.305 | 1.347 | 1.476 | 1.912 |
| FBgn0032200 | 0.932 | 0.974 | 0.999 | 1.487 | 1.698 | 2.069 |
| FBgn0000079 | 0.932 | 0.979 | 0.959 | 0.875 | 0.846 | 0.771 |
| FBgn0029924 | 0.932 | 0.824 | 0.848 | 0.838 | 0.971 | 1.062 |
| FBgn0262035 | 0.932 | 0.979 | 1.171 | 1.245 | 1.367 | 1.617 |
| FBgn0034398 | 0.932 | 1.173 | 1.259 | 1.287 | 1.804 | 2.078 |
| FBgn0034265 | 0.932 | 1.161 | 1.195 | 1.167 | 1.327 | 1.496 |
| FBgn0010414 | 0.932 | 1.022 | 1.011 | 1.172 | 1.268 | 1.380 |
| FBgn0037794 | 0.932 | 1.068 | 1.111 | 1.097 | 1.674 | 2.066 |
| FBgn0033755 | 0.932 | 0.997 | 1.225 | 1.193 | 1.646 | 1.970 |
| FBgn0035876 | 0.932 | 0.634 | 0.714 | 0.666 | 0.859 | 1.106 |
| FBgn0037782 | 0.932 | 1.017 | 1.597 | 1.773 | 3.540 | 4.456 |
| FBgn0031726 | 0.931 | 1.117 | 1.118 | 1.418 | 1.474 | 1.738 |
| FBgn0034627 | 0.931 | 0.678 | 0.637 | 0.699 | 1.066 | 1.287 |
| FBgn0034628 | 0.931 | 1.000 | 0.995 | 1.008 | 1.318 | 1.520 |
| FBgn0019960 | 0.930 | 0.948 | 0.887 | 0.821 | 0.720 | 0.641 |
| FBgn0037960 | 0.930 | 0.501 | 0.612 | 0.705 | 1.157 | 1.367 |
| FBgn0013303 | 0.930 | 0.612 | 0.705 | 1.169 | 1.479 | 1.825 |
| FBgn0032192 | 0.930 | 0.889 | 1.420 | 1.749 | 1.943 | 2.790 |
| FBgn0025697 | 0.929 | 0.980 | 1.316 | 1.356 | 2.000 | 2.401 |
| FBgn0033479 | 0.929 | 0.955 | 0.993 | 0.844 | 0.575 | 0.439 |
| FBgn0040732 | 0.929 | 1.089 | 0.955 | 1.079 | 1.296 | 1.847 |
| FBgn0020270 | 0.928 | 0.828 | 1.133 | 1.155 | 1.404 | 1.773 |
| FBgn0031630 | 0.928 | 0.910 | 0.875 | 1.104 | 1.203 | 1.365 |
| FBgn0022959 | 0.928 | 0.989 | 0.909 | 0.880 | 0.761 | 0.677 |
| FBgn0033927 | 0.927 | 1.084 | 1.156 | 1.082 | 1.636 | 2.703 |
| FBgn0029737 | 0.927 | 0.915 | 0.933 | 0.924 | 0.833 | 0.753 |
| FBgn0051719 | 0.927 | 0.881 | 0.956 | 1.046 | 1.211 | 1.310 |
| FBgn0032404 | 0.927 | 0.745 | 0.789 | 0.769 | 0.925 | 1.033 |
| FBgn0259222 | 0.927 | 0.894 | 0.973 | 1.170 | 1.199 | 1.425 |
| FBgn0025456 | 0.927 | 1.113 | 1.204 | 1.166 | 1.367 | 1.530 |
| FBgn0026787 | 0.926 | 1.179 | 1.183 | 1.176 | 1.371 | 1.509 |
| FBgn0051915 | 0.926 | 1.031 | 0.975 | 0.985 | 1.300 | 1.661 |
| FBgn0259821 | 0.926 | 1.214 | 1.484 | 1.469 | 1.682 | 2.049 |
| FBgn0053007 | 0.926 | 0.773 | 0.964 | 1.038 | 1.221 | 1.426 |
| FBgn0031640 | 0.926 | 1.124 | 1.173 | 1.208 | 1.237 | 1.303 |
| FBgn0037057 | 0.926 | 0.973 | 0.889 | 0.903 | 0.839 | 0.684 |
| FBgn0026777 | 0.926 | 1.009 | 1.133 | 1.435 | 1.966 | 2.229 |
| FBgn0039531 | 0.926 | 1.005 | 1.000 | 1.030 | 1.026 | 1.082 |
| FBgn0016078 | 0.925 | 1.100 | 1.190 | 1.199 | 1.500 | 1.651 |
| FBgn0040239 | 0.925 | 1.110 | 1.225 | 1.238 | 1.286 | 1.541 |
| FBgn0031738 | 0.925 | 0.915 | 0.925 | 0.937 | 0.937 | 0.993 |
| FBgn0038484 | 0.925 | 1.426 | 1.397 | 1.742 | 1.908 | 2.140 |
| FBgn0031604 | 0.925 | 0.996 | 1.007 | 0.992 | 1.077 | 1.171 |
| FBgn0032719 | 0.925 | 0.903 | 0.869 | 0.887 | 1.025 | 1.151 |
| FBgn0052196 | 0.924 | 2.874 | 4.141 | 3.995 | 5.277 | 6.853 |
| FBgn0085805 | 0.924 | 0.282 | 0.260 | 0.322 | 0.815 | 1.068 |
| FBgn0034182 | 0.923 | 1.124 | 1.401 | 1.555 | 1.997 | 2.282 |
| FBgn0034179 | 0.923 | 0.978 | 1.052 | 1.134 | 1.437 | 1.564 |
| FBgn0051641 | 0.923 | 1.012 | 0.955 | 0.932 | 0.770 | 0.695 |
| FBgn0039747 | 0.923 | 0.794 | 0.797 | 0.780 | 0.716 | 0.688 |
| FBgn0028526 | 0.923 | 1.056 | 1.397 | 1.290 | 1.909 | 2.399 |
| FBgn0028373 | 0.923 | 0.870 | 0.860 | 0.860 | 0.966 | 1.053 |
| FBgn0034381 | 0.923 | 0.574 | 0.632 | 0.686 | 0.982 | 1.105 |
| FBgn0038002 | 0.922 | 1.104 | 1.148 | 1.110 | 1.258 | 1.496 |
| FBgn0044048 | 0.922 | 0.632 | 0.596 | 0.649 | 0.793 | 1.308 |
| FBgn0033782 | 0.922 | 0.616 | 0.630 | 0.776 | 0.923 | 1.017 |
| FBgn0053230 | 0.922 | 1.020 | 1.023 | 1.008 | 1.114 | 1.274 |
| FBgn0004603 | 0.921 | 1.260 | 1.337 | 1.298 | 1.581 | 1.765 |
| FBgn0033347 | 0.921 | 0.984 | 0.976 | 1.104 | 1.320 | 1.415 |
| FBgn0053177 | 0.921 | 2.414 | 3.021 | NA | 3.476 | 4.239 |
| FBgn0003071 | 0.920 | 0.965 | 0.961 | 0.976 | 0.787 | 0.647 |
| FBgn0030647 | 0.920 | 1.466 | 1.138 | 1.017 | 0.358 | 0.018 |
| FBgn0022936 | 0.920 | 0.961 | 1.146 | 1.452 | 1.527 | 1.895 |
| FBgn0036627 | 0.920 | 0.990 | 1.143 | 1.081 | 1.387 | 1.629 |
| FBgn0036023 | 0.920 | 1.001 | 1.146 | 0.879 | 0.750 | 0.509 |
| FBgn0033188 | 0.919 | 0.814 | 0.753 | 0.865 | 1.018 | 1.114 |
| FBgn0010709 | 0.919 | 1.352 | 1.313 | 1.336 | 1.418 | 1.546 |
| FBgn0039925 | 0.919 | 0.561 | 0.729 | 0.798 | 1.712 | 2.104 |
| FBgn0034897 | 0.919 | 1.139 | 1.120 | 1.216 | 1.504 | 1.622 |
| FBgn0039890 | 0.919 | 0.581 | 0.960 | 1.343 | 1.643 | 2.127 |
| FBgn0051176 | 0.919 | 1.131 | 1.250 | 1.178 | 1.394 | 1.783 |
| FBgn0053288 | 0.919 | 1.568 | 1.480 | 1.543 | 1.846 | 2.072 |
| FBgn0034553 | 0.919 | 0.932 | 0.861 | 1.095 | 1.441 | 1.609 |
| FBgn0033926 | 0.919 | 1.041 | 1.163 | 1.042 | 1.610 | 2.730 |
| FBgn0039163 | 0.918 | 1.213 | 1.252 | 1.304 | 1.316 | 1.389 |
| FBgn0250791 | 0.918 | 1.034 | 1.030 | 1.032 | 1.073 | 1.100 |
| FBgn0033836 | 0.918 | 1.100 | 1.059 | 1.070 | 1.296 | 1.789 |
| FBgn0051710 | 0.918 | 1.089 | 1.244 | 1.195 | 1.604 | 1.849 |
| FBgn0032859 | 0.918 | 1.074 | 1.221 | 1.291 | 1.315 | 1.594 |
| FBgn0035505 | 0.917 | 0.756 | 0.825 | 0.891 | 1.186 | 1.300 |
| FBgn0052676 | 0.917 | 1.019 | 0.963 | 0.892 | 0.831 | 0.759 |
| FBgn0003044 | 0.917 | 0.914 | 0.939 | 0.838 | 0.736 | 0.674 |
| FBgn0028399 | 0.917 | 1.065 | 1.062 | 1.178 | 1.405 | 1.495 |
| FBgn0028523 | 0.917 | 1.500 | 2.264 | 2.390 | 2.908 | 3.760 |
| FBgn0262563 | 0.917 | 0.752 | 0.878 | 0.789 | 1.066 | 1.403 |
| FBgn0039056 | 0.916 | 1.121 | 1.242 | 1.232 | 1.411 | 1.537 |
| FBgn0002565 | 0.916 | 0.991 | 1.015 | 0.793 | 0.793 | 0.575 |
| FBgn0033744 | 0.916 | 1.011 | 1.013 | 0.998 | 1.116 | 1.340 |
| FBgn0051265 | 0.916 | 0.626 | 0.576 | 0.718 | 0.722 | 0.885 |
| FBgn0261041 | 0.916 | 1.082 | 1.049 | 1.049 | 1.573 | 1.917 |
| FBgn0032167 | 0.916 | 1.087 | 1.065 | 1.041 | 1.344 | 1.637 |
| FBgn0036665 | 0.916 | 1.334 | 1.358 | 1.627 | 1.638 | 1.892 |
| FBgn0015781 | 0.915 | 1.154 | 1.119 | 1.094 | 1.093 | 1.022 |
| FBgn0051201 | 0.915 | 0.950 | 1.050 | 0.976 | 1.195 | 1.525 |
| FBgn0039911 | 0.915 | 1.198 | 1.479 | 1.895 | 2.654 | 3.019 |
| FBgn0039728 | 0.915 | 1.411 | 1.633 | 1.596 | 1.959 | 3.374 |
| FBgn0033915 | 0.915 | 0.971 | 0.940 | 0.989 | 1.085 | 1.137 |
| FBgn0028664 | 0.915 | 1.095 | 1.145 | 1.225 | 1.763 | 1.971 |
| FBgn0037936 | 0.915 | 0.961 | 1.283 | 1.632 | 2.307 | 2.663 |
| FBgn0082585 | 0.915 | 1.032 | 0.995 | 1.162 | 1.202 | 1.325 |
| FBgn0032036 | 0.915 | 1.090 | 1.174 | 1.245 | 1.436 | 1.525 |
| FBgn0031461 | 0.915 | 0.785 | 0.721 | 0.811 | 0.990 | 1.092 |
| FBgn0036487 | 0.915 | 1.180 | 1.093 | 1.153 | 1.294 | 1.514 |
| FBgn0036556 | 0.915 | 1.260 | 1.307 | 1.355 | 1.924 | 2.162 |
| FBgn0042111 | 0.915 | 1.321 | 1.302 | 1.425 | 1.468 | 1.549 |
| FBgn0030694 | 0.914 | 1.165 | 1.765 | 1.759 | 2.148 | 2.902 |
| FBgn0028675 | 0.914 | 1.548 | 1.682 | 1.604 | 2.434 | 2.905 |
| FBgn0036985 | 0.914 | 0.510 | 0.562 | 0.481 | 1.027 | 2.234 |
| FBgn0003141 | 0.914 | 0.924 | 1.353 | 1.515 | 1.899 | 2.312 |
| FBgn0003357 | 0.913 | 1.056 | 0.970 | 0.996 | 0.858 | 0.753 |
| FBgn0038424 | 0.913 | 1.223 | 1.216 | 1.241 | 1.022 | 0.855 |
| FBgn0260004 | 0.913 | 0.930 | 1.438 | 1.138 | 2.121 | 3.094 |
| FBgn0026869 | 0.913 | 1.007 | 1.438 | 1.414 | 2.343 | 2.819 |
| FBgn0034002 | 0.912 | 0.999 | 1.022 | 0.979 | 1.189 | 1.398 |
| FBgn0015570 | 0.912 | 1.145 | 1.106 | 1.371 | 1.597 | 1.743 |
| FBgn0032943 | 0.912 | 0.999 | 1.459 | 1.697 | 2.054 | 2.521 |
| FBgn0053281 | 0.912 | 0.926 | 0.874 | 0.939 | 1.223 | 1.362 |
| FBgn0011703 | 0.912 | 1.361 | 2.022 | 2.523 | 5.672 | 6.819 |
| FBgn0261882 | 0.912 | 1.148 | 1.400 | 1.472 | 1.515 | 2.163 |
| FBgn0035049 | 0.912 | 1.221 | 1.185 | 1.206 | 1.332 | 1.425 |
| FBgn0021761 | 0.912 | 1.094 | 1.376 | 1.212 | 1.688 | 2.243 |
| FBgn0038536 | 0.912 | 1.351 | 1.290 | 1.506 | 1.539 | 1.715 |
| FBgn0031097 | 0.912 | 0.763 | 0.708 | 0.892 | 0.976 | 1.093 |
| FBgn0034582 | 0.911 | 1.120 | 1.161 | 1.039 | 1.043 | 0.896 |
| FBgn0035519 | 0.911 | 0.982 | 1.012 | 0.918 | 0.885 | 0.820 |
| FBgn0032472 | 0.911 | 0.556 | 0.983 | 1.432 | 1.883 | 2.360 |
| FBgn0053144 | 0.911 | 0.843 | 0.827 | 0.838 | 0.724 | 0.660 |
| FBgn0038035 | 0.911 | 0.892 | 1.031 | 0.990 | 1.763 | 2.133 |
| FBgn0035228 | 0.910 | 0.979 | 0.948 | 0.923 | 0.918 | 0.866 |
| FBgn0016696 | 0.910 | 1.105 | 1.026 | 1.093 | 1.235 | 1.364 |
| FBgn0044510 | 0.910 | 1.910 | 1.215 | 1.815 | 2.577 | 4.759 |
| FBgn0030157 | 0.910 | 1.109 | 1.357 | 1.449 | 1.863 | 2.080 |
| FBgn0030930 | 0.910 | 0.950 | 0.878 | 0.906 | 0.777 | 0.682 |
| FBgn0038880 | 0.909 | 2.205 | 1.876 | 1.919 | 3.213 | 4.606 |
| FBgn0031799 | 0.909 | 1.125 | 1.137 | 1.147 | 1.248 | 1.286 |
| FBgn0086675 | 0.909 | 0.778 | 0.862 | 0.808 | 1.085 | 1.264 |
| FBgn0039564 | 0.909 | 0.780 | 0.808 | 1.011 | 0.960 | 1.294 |
| FBgn0033033 | 0.909 | 0.885 | 0.891 | 0.841 | 1.212 | 1.516 |
| FBgn0034579 | 0.908 | 0.970 | 0.991 | 1.094 | 1.198 | 1.257 |
| FBgn0002578 | 0.908 | 1.115 | 1.237 | 1.156 | 0.973 | 0.681 |
| FBgn0035089 | 0.908 | 1.507 | 1.802 | 2.613 | 2.878 | 3.542 |
| FBgn0035317 | 0.908 | 0.671 | 1.189 | 0.880 | 1.683 | 2.770 |
| FBgn0032614 | 0.908 | 0.942 | 1.014 | 1.034 | 1.294 | 1.392 |
| FBgn0037743 | 0.908 | 1.327 | 1.510 | 1.443 | 1.606 | 2.020 |
| FBgn0035976 | 0.908 | 1.135 | 1.077 | 1.126 | 1.194 | 1.406 |
| FBgn0053178 | 0.908 | 0.845 | 1.255 | 1.414 | 1.605 | 2.080 |
| FBgn0037071 | 0.907 | 1.057 | 1.209 | 1.319 | 2.386 | 2.767 |
| FBgn0030897 | 0.907 | 0.507 | 0.616 | 0.595 | 0.803 | 0.919 |
| FBgn0034605 | 0.907 | 0.931 | 1.206 | 1.464 | 1.628 | 1.963 |
| FBgn0051102 | 0.907 | 1.084 | 1.251 | 1.140 | 1.667 | 2.011 |
| FBgn0030037 | 0.907 | 0.934 | 0.901 | 0.854 | 0.680 | 0.624 |
| FBgn0033615 | 0.906 | 0.742 | 1.351 | 1.150 | 1.724 | 2.623 |
| FBgn0016041 | 0.906 | 0.919 | 0.973 | 0.923 | 0.764 | 0.670 |
| FBgn0038721 | 0.906 | 1.055 | 1.190 | 1.149 | 1.569 | 1.773 |
| FBgn0031693 | 0.906 | 0.812 | 1.237 | 1.309 | 1.934 | 2.292 |
| FBgn0024248 | 0.906 | 1.251 | 1.441 | 1.392 | 1.882 | 2.126 |
| FBgn0031713 | 0.906 | 1.014 | 1.012 | 1.162 | 1.557 | 1.685 |
| FBgn0032233 | 0.906 | 0.742 | 0.671 | 0.868 | 0.901 | 1.062 |
| FBgn0083121 | 0.905 | 0.914 | 0.983 | 1.062 | 1.234 | 1.306 |
| FBgn0027081 | 0.905 | 0.907 | 0.949 | 0.975 | 1.018 | 1.056 |
| FBgn0261574 | 0.905 | 1.187 | 1.462 | 1.270 | 1.972 | 2.519 |
| FBgn0051267 | 0.905 | 0.950 | 0.950 | 1.115 | 1.075 | 1.329 |
| FBgn0040507 | 0.905 | 0.957 | 0.967 | 1.003 | 1.208 | 1.277 |
| FBgn0037697 | 0.905 | 1.098 | 0.990 | 0.862 | 0.853 | 0.655 |
| FBgn0014033 | 0.904 | 1.125 | 1.176 | 1.188 | 2.010 | 2.357 |
| FBgn0034656 | 0.904 | 0.971 | 1.116 | 1.161 | 1.369 | 1.487 |
| FBgn0035170 | 0.904 | 0.878 | 0.930 | 1.169 | 1.178 | 1.400 |
| FBgn0027495 | 0.904 | 1.019 | 0.988 | 0.828 | 0.646 | 0.560 |
| FBgn0039277 | 0.904 | 0.943 | 1.088 | 1.309 | 1.241 | 1.797 |
| FBgn0015569 | 0.904 | 0.987 | 1.188 | 1.142 | 1.422 | 1.626 |
| FBgn0030418 | 0.904 | 1.014 | 1.151 | 1.280 | 1.841 | 2.021 |
| FBgn0250815 | 0.903 | 1.048 | 1.102 | 0.964 | 0.845 | 0.767 |
| FBgn0036587 | 0.903 | 0.833 | 2.082 | 2.106 | 4.194 | 5.274 |
| FBgn0025726 | 0.903 | 0.790 | 1.194 | 1.191 | 2.323 | 2.779 |
| FBgn0259482 | 0.903 | 0.624 | 0.909 | 0.947 | 1.105 | 1.415 |
| FBgn0035412 | 0.902 | 1.754 | 1.669 | 1.742 | 1.412 | 1.191 |
| FBgn0040022 | 0.902 | 1.293 | 1.937 | 1.666 | 2.795 | 3.600 |
| FBgn0053653 | 0.902 | 1.055 | 1.513 | 1.810 | 2.349 | 2.742 |
| FBgn0038966 | 0.902 | 1.243 | 1.321 | 1.307 | 1.775 | 1.967 |
| FBgn0036260 | 0.902 | 0.899 | 0.946 | 0.895 | 1.039 | 1.273 |
| FBgn0034480 | 0.902 | 0.809 | 1.063 | 1.500 | 1.434 | 2.129 |
| FBgn0033438 | 0.902 | 0.896 | 0.835 | 0.889 | 0.964 | 1.059 |
| FBgn0043364 | 0.902 | 0.921 | 0.964 | 0.888 | 1.230 | 1.511 |
| FBgn0001258 | 0.901 | 0.851 | 0.808 | 0.955 | 1.081 | 1.157 |
| FBgn0011288 | 0.901 | 0.553 | 0.596 | 0.716 | 0.948 | 1.025 |
| FBgn0039761 | 0.901 | 1.289 | 1.370 | 1.392 | 2.120 | 2.391 |
| FBgn0036844 | 0.901 | 1.157 | 1.195 | 1.147 | 1.309 | 1.465 |
| FBgn0032673 | 0.901 | 0.780 | 0.777 | 0.856 | 0.903 | 0.944 |
| FBgn0034820 | 0.901 | 1.358 | 1.458 | 1.014 | 0.737 | 0.508 |
| FBgn0034391 | 0.901 | NA | 1.218 | 1.040 | 1.607 | 2.760 |
| FBgn0035343 | 0.901 | 0.654 | 0.842 | 0.958 | 0.991 | 1.267 |
| FBgn0052056 | 0.901 | 0.964 | 1.014 | 1.037 | 1.343 | 1.446 |
| FBgn0040715 | 0.900 | 0.907 | 1.172 | 1.267 | 1.410 | 1.682 |
| FBgn0259717 | 0.900 | 0.907 | 1.172 | 1.267 | 1.410 | 1.682 |
| FBgn0023520 | 0.900 | 0.884 | 0.922 | 0.989 | 1.149 | 2.205 |
| FBgn0032701 | 0.900 | 1.347 | 1.528 | 1.556 | 1.711 | 1.868 |
| FBgn0052407 | 0.900 | 0.977 | 1.339 | 1.515 | 2.245 | 2.541 |
| FBgn0262096 | 0.900 | 0.253 | 0.347 | 0.383 | 0.390 | 0.567 |

| Supplemental Table 1D. Values of R Squared and Fold Changes for Genes Found to Behave Linearly at Days 2 and 10 Post-Irradiation. (Analysis with all data included.) | | | |  | |  | |  | |  | |  | |  | |  | |  | |  | |
| --- | --- | --- | --- | --- | --- | --- | --- | --- | --- | --- | --- | --- | --- | --- | --- | --- | --- | --- | --- | --- | --- |
|  |  |  |  | |  | |  | |  | |  | |  | |  | |  | |  | |  |
| Flybase ID | R Sqaured Value Day2 | R Squared Value Day 10 | Day 2 Fold Change 10 R | | Day 2 Fold Change 1000 R | | Day 2 Fold Change 5000 R | | Day 2 Fold Change 10000 R | | Day 2 Fold Change 20000 R | | Day 10 fold Change 10R | | Day 10 fold Change 1000R | | Day 10 fold Change 5000R | | Day 10 fold Change 10000 R | | Day 10 fold Change 20000R |
| FBgn0011774 | 0.902 | 0.968 | 0.598 | | 0.954 | | 1.315 | | 1.428 | | 1.933 | | 1.075 | | 1.035 | | 1.228 | | 1.975 | | 2.650 |
| FBgn0023001 | 0.900 | 0.924 | 0.919 | | 1.004 | | 1.008 | | 1.106 | | 1.177 | | 0.972 | | 0.994 | | 0.967 | | 1.193 | | 1.373 |
| FBgn0030026 | 0.960 | 0.978 | 1.065 | | 1.122 | | 1.183 | | 1.213 | | 1.467 | | 0.927 | | 0.938 | | 1.052 | | 1.397 | | 1.718 |
| FBgn0030189 | 0.944 | 0.943 | 0.725 | | 0.777 | | 0.848 | | 1.010 | | 1.108 | | 0.785 | | 0.992 | | 1.010 | | 1.195 | | 1.489 |
| FBgn0030234 | 0.968 | 0.942 | 1.056 | | 1.153 | | 1.162 | | 1.301 | | 1.512 | | 0.881 | | 1.070 | | 1.079 | | 1.227 | | 1.527 |
| FBgn0031713 | 0.985 | 0.995 | 0.986 | | 1.021 | | 1.110 | | 1.158 | | 1.324 | | 0.991 | | 1.034 | | 1.183 | | 1.417 | | 1.739 |
| FBgn0032393 | 0.979 | 0.980 | 1.106 | | 1.135 | | 1.347 | | 1.423 | | 1.734 | | 0.998 | | 1.130 | | 1.699 | | 2.025 | | 2.818 |
| FBgn0036881 | 0.908 | 0.915 | 0.780 | | 1.074 | | 1.046 | | 1.350 | | 1.646 | | 0.926 | | 1.173 | | 0.933 | | 1.820 | | 2.768 |
| FBgn0037020 | 0.988 | 0.943 | 0.914 | | 0.938 | | 0.953 | | 1.001 | | 1.089 | | 0.897 | | 0.927 | | 1.089 | | 1.107 | | 1.298 |
| FBgn0039411 | 0.922 | 0.908 | 0.905 | | 1.058 | | 0.897 | | 1.589 | | 2.415 | | 0.853 | | 1.192 | | 0.994 | | 1.483 | | 2.494 |
| FBgn0051864 | 0.937 | 0.970 | 0.773 | | 1.096 | | 1.138 | | 1.421 | | 1.832 | | 1.100 | | 1.072 | | 1.596 | | 1.711 | | 2.806 |

| Supplemental Table 1E. Values of R Squared and Fold Changes for Genes Found to Behave Linearly at Days 10 and 20 Post-Irradiation. (Analysis with all data included.) | | | | | |  | |  | |  | |  | |  | |  | |  | |  |
| --- | --- | --- | --- | --- | --- | --- | --- | --- | --- | --- | --- | --- | --- | --- | --- | --- | --- | --- | --- | --- |
|  |  |  |  |  |  | |  | |  | |  | |  | |  | |  | |  | |
| Flybase ID | R Squared Value Day 10 | R Squared Value Day20 | Day10 Fold Change 10R | Day10 Fold Change 1000R | Day 10 fold Change 5000R | | Day10 fold Change 10000R | | Day10 Fold Change 20000R | | Day 20 Fold Change 10R | | Day 20 Fold Change 1000R | | Day 20 Fold Change 5000R | | Day 20 Fold Change 10000R | | Day 20 Fold Change 20000R | |
| FBgn0003071 | 0.970 | 0.920 | 1.110 | 1.111 | 0.953 | | 0.816 | | 0.647 | | 0.965 | | 0.961 | | 0.976 | | 0.787 | | 0.647 | |
| FBgn0003495 | 0.939 | 0.946 | 0.758 | 0.988 | 0.997 | | 1.363 | | 1.662 | | 0.871 | | 0.925 | | 0.914 | | 1.135 | | 1.602 | |
| FBgn0004580 | 0.905 | 0.962 | 0.787 | 0.952 | 0.888 | | 1.044 | | 1.502 | | 1.088 | | 1.104 | | 1.162 | | 1.234 | | 1.553 | |
| FBgn0010038 | 0.964 | 0.994 | 2.798 | 2.277 | 5.455 | | 6.142 | | 10.345 | | 0.497 | | 0.766 | | 1.195 | | 1.706 | | 3.165 | |
| FBgn0010173 | 0.944 | 0.944 | 0.886 | 1.079 | 1.116 | | 1.383 | | 1.629 | | 1.079 | | 1.309 | | 1.238 | | 1.770 | | 2.273 | |
| FBgn0010348 | 0.989 | 0.936 | 0.825 | 0.872 | 0.941 | | 1.061 | | 1.219 | | 1.104 | | 1.132 | | 1.179 | | 1.417 | | 1.534 | |
| FBgn0011288 | 0.916 | 0.901 | 0.583 | 0.817 | 0.935 | | 0.953 | | 1.684 | | 0.553 | | 0.596 | | 0.716 | | 0.948 | | 1.025 | |
| FBgn0011591 | 0.931 | 0.951 | 0.847 | 0.979 | 1.028 | | 1.205 | | 1.348 | | 0.830 | | 0.871 | | 1.046 | | 1.338 | | 1.530 | |
| FBgn0011774 | 0.968 | 0.959 | 1.075 | 1.035 | 1.228 | | 1.975 | | 2.650 | | 1.034 | | 1.096 | | 1.113 | | 1.871 | | 2.722 | |
| FBgn0015351 | 0.946 | 0.982 | 0.617 | 1.072 | 1.131 | | 1.446 | | 2.242 | | 0.930 | | 1.171 | | 1.332 | | 1.589 | | 2.294 | |
| FBgn0016041 | 0.997 | 0.906 | 1.091 | 1.076 | 1.007 | | 0.883 | | 0.651 | | 0.919 | | 0.973 | | 0.923 | | 0.764 | | 0.670 | |
| FBgn0016126 | 0.922 | 0.954 | 0.674 | 1.024 | 0.949 | | 1.237 | | 2.094 | | 0.813 | | 1.401 | | 1.555 | | 2.394 | | 3.246 | |
| FBgn0020270 | 0.981 | 0.928 | 0.916 | 1.036 | 1.096 | | 1.529 | | 2.202 | | 0.828 | | 1.133 | | 1.155 | | 1.404 | | 1.773 | |
| FBgn0020416 | 0.923 | 0.945 | 0.545 | 0.778 | 0.760 | | 0.958 | | 1.255 | | 0.968 | | 1.304 | | 1.290 | | 1.732 | | 2.223 | |
| FBgn0023000 | 0.989 | 0.976 | 0.826 | 0.910 | 1.010 | | 1.237 | | 1.522 | | 0.948 | | 1.167 | | 1.204 | | 1.648 | | 2.278 | |
| FBgn0023514 | 0.963 | 0.981 | 1.048 | 0.989 | 1.071 | | 1.205 | | 1.423 | | 1.168 | | 1.207 | | 1.274 | | 1.341 | | 1.457 | |
| FBgn0024248 | 0.915 | 0.906 | 0.738 | 0.887 | 0.832 | | 1.028 | | 1.244 | | 1.251 | | 1.441 | | 1.392 | | 1.882 | | 2.126 | |
| FBgn0024912 | 0.975 | 0.989 | 0.778 | 1.051 | 1.549 | | 1.898 | | 2.703 | | 1.184 | | 1.400 | | 1.985 | | 2.959 | | 4.117 | |
| FBgn0024913 | 0.934 | 0.971 | 0.617 | 0.941 | 1.054 | | 1.342 | | 3.008 | | 1.407 | | 1.339 | | 1.757 | | 1.967 | | 3.099 | |
| FBgn0025583 | 0.930 | 0.995 | 0.549 | 0.860 | 1.225 | | 1.327 | | 1.917 | | 0.958 | | 1.192 | | 1.835 | | 2.344 | | 3.712 | |
| FBgn0025808 | 0.913 | 0.966 | 0.910 | 0.996 | 0.971 | | 1.052 | | 1.315 | | 1.391 | | 1.306 | | 1.212 | | 1.171 | | 0.872 | |
| FBgn0025879 | 0.912 | 0.953 | 0.691 | 0.904 | 0.961 | | 1.129 | | 1.339 | | 1.010 | | 1.100 | | 1.179 | | 1.534 | | 1.757 | |
| FBgn0028394 | 0.905 | 0.962 | 0.883 | 0.951 | 1.051 | | 1.131 | | 1.215 | | 1.260 | | 1.317 | | 1.445 | | 1.478 | | 1.682 | |
| FBgn0028399 | 0.963 | 0.917 | 0.873 | 0.934 | 1.071 | | 1.157 | | 1.331 | | 1.065 | | 1.062 | | 1.178 | | 1.405 | | 1.495 | |
| FBgn0028411 | 0.957 | 0.950 | 1.292 | 1.246 | 1.759 | | 1.816 | | 2.514 | | 2.203 | | 2.019 | | 2.891 | | 3.009 | | 4.087 | |
| FBgn0028938 | 0.903 | 0.956 | 0.729 | 1.087 | 0.688 | | 1.871 | | 3.380 | | 1.186 | | 1.358 | | 1.307 | | 2.020 | | 3.024 | |
| FBgn0029664 | 0.981 | 0.940 | 0.857 | 0.932 | 0.979 | | 1.056 | | 1.247 | | 1.243 | | 1.441 | | 1.489 | | 1.631 | | 1.906 | |
| FBgn0030029 | 0.914 | 0.969 | 0.826 | 1.040 | 0.899 | | 1.313 | | 1.833 | | 1.060 | | 1.040 | | 1.216 | | 1.306 | | 1.494 | |
| FBgn0030038 | 0.970 | 0.951 | 1.093 | 1.024 | 0.957 | | 0.885 | | 0.744 | | 1.051 | | 1.010 | | 0.957 | | 0.892 | | 0.822 | |
| FBgn0030157 | 0.949 | 0.910 | 0.654 | 0.846 | 0.849 | | 1.011 | | 1.418 | | 1.109 | | 1.357 | | 1.449 | | 1.863 | | 2.080 | |
| FBgn0030189 | 0.943 | 0.993 | 0.785 | 0.992 | 1.010 | | 1.195 | | 1.489 | | 1.131 | | 1.168 | | 1.303 | | 1.424 | | 1.817 | |
| FBgn0030418 | 0.917 | 0.904 | 0.809 | 1.022 | 0.816 | | 1.599 | | 2.437 | | 1.014 | | 1.151 | | 1.280 | | 1.841 | | 2.021 | |
| FBgn0031307 | 0.905 | 0.959 | 0.958 | 0.952 | 0.994 | | 1.707 | | 2.013 | | 1.351 | | 1.214 | | 1.543 | | 2.492 | | 3.248 | |
| FBgn0031710 | 0.921 | 0.944 | 0.651 | 0.772 | 0.904 | | 0.900 | | 1.192 | | 1.059 | | 1.211 | | 1.238 | | 1.327 | | 1.611 | |
| FBgn0031713 | 0.995 | 0.906 | 0.991 | 1.034 | 1.183 | | 1.417 | | 1.739 | | 1.014 | | 1.012 | | 1.162 | | 1.557 | | 1.685 | |
| FBgn0031726 | 0.971 | 0.931 | 1.083 | 0.984 | 1.164 | | 1.639 | | 2.257 | | 1.117 | | 1.118 | | 1.418 | | 1.474 | | 1.738 | |
| FBgn0032036 | 0.924 | 0.915 | 0.898 | 0.945 | 1.024 | | 1.262 | | 1.362 | | 1.090 | | 1.174 | | 1.245 | | 1.436 | | 1.525 | |
| FBgn0032192 | 0.941 | 0.930 | 0.612 | 0.671 | 1.241 | | 1.419 | | 1.938 | | 0.889 | | 1.420 | | 1.749 | | 1.943 | | 2.790 | |
| FBgn0032230 | 0.981 | 0.935 | 0.893 | 1.122 | 1.343 | | 1.544 | | 2.284 | | 1.283 | | 1.324 | | 1.529 | | 1.998 | | 2.224 | |
| FBgn0032233 | 0.972 | 0.906 | 0.834 | 0.903 | 0.929 | | 1.046 | | 1.359 | | 0.742 | | 0.671 | | 0.868 | | 0.901 | | 1.062 | |
| FBgn0032393 | 0.980 | 0.960 | 0.998 | 1.130 | 1.699 | | 2.025 | | 2.818 | | 0.953 | | 1.020 | | 1.398 | | 2.248 | | 2.817 | |
| FBgn0032470 | 0.993 | 0.991 | 1.178 | 1.184 | 1.421 | | 1.889 | | 2.696 | | 0.911 | | 1.023 | | 1.379 | | 1.656 | | 2.674 | |
| FBgn0032472 | 0.930 | 0.911 | 0.474 | 0.768 | 0.907 | | 0.975 | | 1.588 | | 0.556 | | 0.983 | | 1.432 | | 1.883 | | 2.360 | |
| FBgn0032614 | 0.979 | 0.908 | 0.753 | 0.823 | 0.900 | | 1.044 | | 1.214 | | 0.942 | | 1.014 | | 1.034 | | 1.294 | | 1.392 | |
| FBgn0032859 | 0.927 | 0.918 | 1.125 | 1.152 | 1.141 | | 1.179 | | 1.232 | | 1.074 | | 1.221 | | 1.291 | | 1.315 | | 1.594 | |
| FBgn0033130 | 0.926 | 0.940 | 0.746 | 1.019 | 1.233 | | 1.251 | | 1.886 | | 0.976 | | 1.304 | | 1.352 | | 1.796 | | 2.199 | |
| FBgn0033134 | 0.960 | 0.960 | 0.550 | 0.868 | 1.056 | | 1.333 | | 1.866 | | 1.279 | | 1.832 | | 2.253 | | 3.542 | | 4.562 | |
| FBgn0033153 | 0.934 | 0.938 | 1.040 | 1.028 | 1.422 | | 1.980 | | 2.291 | | 1.066 | | 1.249 | | 1.641 | | 1.785 | | 2.227 | |
| FBgn0033395 | 0.921 | 0.981 | 0.399 | 0.967 | 0.880 | | 1.457 | | 3.702 | | 1.664 | | 2.672 | | 2.987 | | 4.758 | | 7.566 | |
| FBgn0033453 | 0.973 | 0.945 | 0.658 | 0.835 | 1.168 | | 1.339 | | 1.900 | | 0.761 | | 1.092 | | 1.799 | | 2.498 | | 3.213 | |
| FBgn0033744 | 0.958 | 0.916 | 0.748 | 0.902 | 0.913 | | 1.058 | | 1.414 | | 1.011 | | 1.013 | | 0.998 | | 1.116 | | 1.340 | |
| FBgn0033926 | 0.983 | 0.919 | 1.509 | 1.533 | 1.747 | | 2.548 | | 3.675 | | 1.041 | | 1.163 | | 1.042 | | 1.610 | | 2.730 | |
| FBgn0033927 | 0.982 | 0.927 | 1.496 | 1.570 | 1.781 | | 2.694 | | 3.778 | | 1.084 | | 1.156 | | 1.082 | | 1.636 | | 2.703 | |
| FBgn0033928 | 0.991 | 0.965 | 1.144 | 1.364 | 1.837 | | 2.217 | | 3.148 | | 1.548 | | 1.694 | | 1.700 | | 2.473 | | 3.460 | |
| FBgn0034002 | 0.938 | 0.912 | 0.961 | 0.938 | 1.205 | | 1.186 | | 1.650 | | 0.999 | | 1.022 | | 0.979 | | 1.189 | | 1.398 | |
| FBgn0034029 | 0.913 | 0.949 | 0.721 | 1.023 | 0.888 | | 1.236 | | 1.934 | | 1.041 | | 1.188 | | 1.234 | | 1.842 | | 2.237 | |
| FBgn0034184 | 0.988 | 0.983 | 0.986 | 1.031 | 1.072 | | 1.137 | | 1.313 | | 0.949 | | 0.948 | | 1.018 | | 1.052 | | 1.222 | |
| FBgn0034381 | 0.900 | 0.923 | 1.039 | 1.075 | 1.316 | | 1.687 | | 1.817 | | 0.574 | | 0.632 | | 0.686 | | 0.982 | | 1.105 | |
| FBgn0034398 | 0.945 | 0.932 | 0.695 | 0.837 | 1.078 | | 1.174 | | 1.495 | | 1.173 | | 1.259 | | 1.287 | | 1.804 | | 2.078 | |
| FBgn0034480 | 0.969 | 0.902 | 0.917 | 1.195 | 1.283 | | 1.589 | | 2.498 | | 0.809 | | 1.063 | | 1.500 | | 1.434 | | 2.129 | |
| FBgn0034605 | 0.978 | 0.907 | 0.961 | 1.046 | 1.370 | | 1.555 | | 1.999 | | 0.931 | | 1.206 | | 1.464 | | 1.628 | | 1.963 | |
| FBgn0034656 | 0.956 | 0.904 | 0.886 | 0.855 | 0.889 | | 1.025 | | 1.186 | | 0.971 | | 1.116 | | 1.161 | | 1.369 | | 1.487 | |
| FBgn0034726 | 0.928 | 0.959 | 0.690 | 0.890 | 0.988 | | 1.070 | | 1.367 | | 0.908 | | 0.967 | | 1.211 | | 1.327 | | 1.594 | |
| FBgn0035089 | 0.946 | 0.908 | 0.749 | 0.903 | 1.234 | | 1.232 | | 2.122 | | 1.507 | | 1.802 | | 2.613 | | 2.878 | | 3.542 | |
| FBgn0035165 | 0.976 | 0.937 | 0.980 | 0.944 | 1.058 | | 1.119 | | 1.286 | | 1.005 | | 0.974 | | 1.099 | | 1.115 | | 1.249 | |
| FBgn0035166 | 0.971 | 0.949 | 0.922 | 1.024 | 1.081 | | 1.357 | | 1.602 | | 0.802 | | 0.745 | | 0.892 | | 0.939 | | 1.297 | |
| FBgn0035876 | 0.956 | 0.932 | 0.744 | 0.871 | 0.999 | | 1.104 | | 1.335 | | 0.634 | | 0.714 | | 0.666 | | 0.859 | | 1.106 | |
| FBgn0035998 | 0.938 | 0.951 | 0.790 | 0.959 | 0.911 | | 1.118 | | 1.493 | | 1.027 | | 1.217 | | 1.521 | | 1.923 | | 2.292 | |
| FBgn0036290 | 0.994 | 0.995 | 0.664 | 0.797 | 1.069 | | 1.496 | | 2.118 | | 1.324 | | 1.438 | | 1.834 | | 2.689 | | 4.152 | |
| FBgn0036587 | 0.921 | 0.903 | 0.524 | 1.131 | 0.836 | | 1.696 | | 3.112 | | 0.833 | | 2.082 | | 2.106 | | 4.194 | | 5.274 | |
| FBgn0036665 | 0.974 | 0.916 | 0.807 | 0.794 | 0.894 | | 1.328 | | 1.998 | | 1.334 | | 1.358 | | 1.627 | | 1.638 | | 1.892 | |
| FBgn0037020 | 0.943 | 0.958 | 0.897 | 0.927 | 1.089 | | 1.107 | | 1.298 | | 0.978 | | 0.996 | | 0.993 | | 1.100 | | 1.229 | |
| FBgn0037057 | 0.996 | 0.926 | 1.109 | 1.090 | 1.047 | | 0.992 | | 0.900 | | 0.973 | | 0.889 | | 0.903 | | 0.839 | | 0.684 | |
| FBgn0037071 | 0.957 | 0.907 | 0.681 | 1.059 | 1.190 | | 1.524 | | 2.130 | | 1.057 | | 1.209 | | 1.319 | | 2.386 | | 2.767 | |
| FBgn0037076 | 0.976 | 0.944 | 1.079 | 1.039 | 1.271 | | 1.566 | | 1.877 | | 1.051 | | 1.320 | | 1.791 | | 1.788 | | 3.083 | |
| FBgn0037690 | 0.912 | 0.935 | 0.881 | 1.033 | 1.304 | | 1.271 | | 2.444 | | 2.986 | | 2.813 | | 3.917 | | 4.385 | | 5.221 | |
| FBgn0037731 | 0.937 | 0.960 | 0.531 | 0.973 | 1.059 | | 1.436 | | 1.996 | | 1.029 | | 1.164 | | 1.338 | | 1.787 | | 2.110 | |
| FBgn0037743 | 0.902 | 0.908 | 0.757 | 0.909 | 0.838 | | 1.058 | | 1.265 | | 1.327 | | 1.510 | | 1.443 | | 1.606 | | 2.020 | |
| FBgn0037794 | 0.913 | 0.932 | 0.751 | 0.925 | 0.952 | | 1.078 | | 1.943 | | 1.068 | | 1.111 | | 1.097 | | 1.674 | | 2.066 | |
| FBgn0037850 | 0.952 | 0.975 | 0.914 | 1.263 | 2.117 | | 2.280 | | 3.514 | | 1.568 | | 1.411 | | 1.769 | | 2.408 | | 3.722 | |
| FBgn0037960 | 0.908 | 0.930 | 0.448 | 0.777 | 0.840 | | 1.012 | | 1.389 | | 0.501 | | 0.612 | | 0.705 | | 1.157 | | 1.367 | |
| FBgn0038035 | 0.985 | 0.911 | 1.008 | 1.266 | 1.400 | | 1.801 | | 2.690 | | 0.892 | | 1.031 | | 0.990 | | 1.763 | | 2.133 | |
| FBgn0038455 | 0.946 | 0.982 | 0.617 | 1.072 | 1.131 | | 1.446 | | 2.242 | | 0.930 | | 1.171 | | 1.332 | | 1.589 | | 2.294 | |
| FBgn0038795 | 0.925 | 0.936 | 0.651 | 0.952 | 1.052 | | 1.142 | | 1.678 | | 0.966 | | 1.305 | | 1.502 | | 2.317 | | 2.784 | |
| FBgn0038893 | 0.991 | 0.949 | 0.757 | 0.974 | 1.447 | | 1.769 | | 2.943 | | 1.054 | | 1.453 | | 1.398 | | 2.536 | | 3.456 | |
| FBgn0039481 | 0.942 | 0.963 | 0.896 | 0.848 | 0.932 | | 0.979 | | 1.215 | | 0.719 | | 0.688 | | 0.818 | | 0.923 | | 1.054 | |
| FBgn0039544 | 0.902 | 0.981 | 1.152 | 1.094 | 1.090 | | 1.321 | | 1.631 | | 1.200 | | 1.191 | | 1.243 | | 1.365 | | 1.497 | |
| FBgn0039597 | 0.971 | 0.937 | 0.775 | 0.789 | 0.944 | | 0.988 | | 1.195 | | 1.019 | | 1.029 | | 1.246 | | 1.228 | | 1.561 | |
| FBgn0039696 | 0.919 | 0.945 | 0.915 | 0.984 | 1.115 | | 1.157 | | 1.294 | | 0.898 | | 0.856 | | 0.938 | | 1.124 | | 1.251 | |
| FBgn0040099 | 0.917 | 0.963 | 0.582 | 0.728 | 0.892 | | 0.874 | | 1.579 | | 2.094 | | 2.137 | | 2.472 | | 3.254 | | 3.804 | |
| FBgn0040732 | 0.922 | 0.929 | 0.530 | 0.603 | 0.820 | | 0.910 | | 1.093 | | 1.089 | | 0.955 | | 1.079 | | 1.296 | | 1.847 | |
| FBgn0040972 | 0.926 | 0.999 | 0.500 | 0.829 | 1.270 | | 1.317 | | 3.444 | | 0.761 | | 1.042 | | 2.190 | | 3.282 | | 5.736 | |
| FBgn0046763 | 0.982 | 0.981 | 0.738 | 0.875 | 1.061 | | 1.225 | | 1.614 | | 1.292 | | 1.436 | | 1.584 | | 1.813 | | 2.153 | |
| FBgn0050281 | 0.972 | 0.960 | 0.753 | 0.877 | 1.037 | | 1.111 | | 1.507 | | 1.172 | | 1.190 | | 1.415 | | 1.633 | | 1.853 | |
| FBgn0051036 | 0.944 | 0.961 | 0.960 | 1.026 | 1.277 | | 1.386 | | 2.519 | | 0.807 | | 0.921 | | 0.957 | | 1.452 | | 1.848 | |
| FBgn0051710 | 0.969 | 0.918 | 0.687 | 0.835 | 1.148 | | 1.227 | | 1.856 | | 1.089 | | 1.244 | | 1.195 | | 1.604 | | 1.849 | |
| FBgn0051769 | 0.949 | 0.950 | 0.681 | 0.781 | 0.964 | | 0.992 | | 1.300 | | 1.180 | | 1.228 | | 1.276 | | 1.293 | | 1.504 | |
| FBgn0051778 | 0.914 | 0.937 | 0.511 | 0.666 | 0.884 | | 0.849 | | 1.304 | | 1.380 | | 1.299 | | 1.486 | | 2.368 | | 2.893 | |
| FBgn0051864 | 0.970 | 0.976 | 1.100 | 1.072 | 1.596 | | 1.711 | | 2.806 | | 1.061 | | 1.066 | | 1.287 | | 2.116 | | 2.943 | |
| FBgn0052056 | 0.917 | 0.901 | 0.833 | 0.975 | 1.077 | | 1.184 | | 1.345 | | 0.964 | | 1.014 | | 1.037 | | 1.343 | | 1.446 | |
| FBgn0052475 | 0.912 | 0.974 | 0.702 | 0.991 | 1.231 | | 1.196 | | 1.995 | | 0.638 | | 0.836 | | 0.872 | | 1.198 | | 1.657 | |
| FBgn0052640 | 0.946 | 0.984 | 0.535 | 0.815 | 0.933 | | 1.120 | | 2.219 | | 1.346 | | 1.621 | | 1.936 | | 2.795 | | 3.720 | |
| FBgn0052641 | 0.947 | 0.984 | 0.534 | 0.799 | 0.915 | | 1.109 | | 2.212 | | 1.356 | | 1.631 | | 1.979 | | 2.844 | | 3.760 | |
| FBgn0053144 | 0.919 | 0.911 | 1.172 | 1.103 | 0.967 | | 0.896 | | 0.772 | | 0.843 | | 0.827 | | 0.838 | | 0.724 | | 0.660 | |
| FBgn0087039 | 0.952 | 0.968 | 0.686 | 0.925 | 1.002 | | 1.149 | | 1.582 | | 0.974 | | 1.304 | | 1.819 | | 2.216 | | 3.010 | |
| FBgn0259233 | 0.908 | 0.956 | 0.834 | 0.994 | 0.803 | | 1.470 | | 2.213 | | 1.666 | | 1.916 | | 1.909 | | 2.863 | | 3.667 | |
| FBgn0260431 | 0.951 | 0.988 | 0.892 | 1.023 | 1.119 | | 1.171 | | 1.632 | | 1.208 | | 1.163 | | 1.351 | | 1.688 | | 2.237 | |
| FBgn0261429 | 0.903 | 0.970 | 0.661 | 0.933 | 1.163 | | 1.296 | | 3.513 | | 0.887 | | 1.048 | | 1.079 | | 1.849 | | 2.800 | |
| FBgn0261882 | 0.932 | 0.912 | 0.636 | 0.487 | 0.844 | | 0.881 | | 1.546 | | 1.148 | | 1.400 | | 1.472 | | 1.515 | | 2.163 | |
| FBgn0261989 | 0.960 | 0.942 | 1.254 | 1.148 | 2.397 | | 3.470 | | 8.701 | | 0.963 | | 1.963 | | 2.859 | | 5.799 | | 7.485 | |
| FBgn0262524 | 0.958 | 0.983 | 0.948 | 1.069 | 1.440 | | 1.502 | | 2.592 | | 0.920 | | 1.032 | | 1.371 | | 1.607 | | 2.124 | |

| Supplemental Table 1F. Values of R Squared and Fold Changes for Genes Found to Behave Linearly at Days 2 and 20 Post-Irradiation. (Analysis with all data included.) | | | | | | |  |  |  |  |  |  |  |
| --- | --- | --- | --- | --- | --- | --- | --- | --- | --- | --- | --- | --- | --- |
|  |  | |  |  |  |  |  |  |  |  |  |  |  |
| Flybase ID | R Squared Value Day 2 | R Squared Values Day 20 | | Day 2 Fold Change 10R | Day 2 Fold Change 1000R | Day 2 Fold Change 5000R | Day 2 Fold Change 10000R | Day 2 Fold Change 20000R | Day 20 Fold Change 10R | Day 20 Fold Change 1000R | Day 20 Fold Change 5000R | Day 20 Fold Change 10000R | Day 20 Fold Change 20000R |
| FBgn0011774 | 0.902 | 0.959 | | 0.598 | 0.954 | 1.315 | 1.428 | 1.933 | 1.034 | 1.096 | 1.113 | 1.871 | 2.722 |
| FBgn0024989 | 0.948 | 0.989 | | 0.893 | 1.200 | 1.501 | 1.743 | 3.594 | 1.184 | 1.400 | 1.985 | 2.959 | 4.117 |
| FBgn0028675 | 0.971 | 0.914 | | 0.908 | 1.051 | 1.236 | 1.315 | 1.775 | 1.548 | 1.682 | 1.604 | 2.434 | 2.905 |
| FBgn0030189 | 0.944 | 0.999 | | 0.725 | 0.777 | 0.848 | 1.010 | 1.108 | 0.761 | 1.042 | 2.190 | 3.282 | 5.736 |
| FBgn0031713 | 0.985 | 0.906 | | 0.986 | 1.021 | 1.110 | 1.158 | 1.324 | 1.014 | 1.012 | 1.162 | 1.557 | 1.685 |
| FBgn0032393 | 0.979 | 0.960 | | 1.106 | 1.135 | 1.347 | 1.423 | 1.734 | 0.953 | 1.020 | 1.398 | 2.248 | 2.817 |
| FBgn0037020 | 0.988 | 0.958 | | 0.914 | 0.938 | 0.953 | 1.001 | 1.089 | 0.978 | 0.996 | 0.993 | 1.100 | 1.229 |
| FBgn0051864 | 0.937 | 0.976 | | 0.773 | 1.096 | 1.138 | 1.421 | 1.832 | 0.948 | 1.167 | 1.204 | 1.648 | 2.278 |

| Supplemental Table 1G. Values of R Squared and Fold Changes for Genes Found to Behave Linearly at Days 2, 10 and 20 Post-Irradiation. (Analysis with all data included.) | | | | | | | | | | | | |  |  |  |  |  |  |
| --- | --- | --- | --- | --- | --- | --- | --- | --- | --- | --- | --- | --- | --- | --- | --- | --- | --- | --- |
| Flybase ID | R^2 Value Day 2 | R^2 Value Day 10 | R^2 Value Day 20 | Day 2 FC 10R | Day 2 FC 1000R | Day 2 FC 5000R | Day 2 FC 10000R | Day 2 FC 20000R | Day10 FC 10R | Day10 FC 1000R | Day 10 FC 5000R | Day10 FC 10000R | Day10 FC 20000R | Day 20 FC 10R | Day 20 FC 1000R | Day 20 FC 5000R | Day 20 FC 10000R | Day 20 FC 20000R |
| FBgn0011774 | 0.902 | 0.968 | 0.959 | 0.598 | 0.954 | 1.315 | 1.428 | 1.933 | 1.075 | 1.035 | 1.228 | 1.975 | 2.650 | 1.034 | 1.096 | 1.113 | 1.871 | 2.722 |
| FBgn0030189 | 0.943 | 0.943 | 0.993 | 0.785 | 0.992 | 1.010 | 1.195 | 1.489 | 0.785 | 0.992 | 1.010 | 1.195 | 1.489 | 1.131 | 1.168 | 1.303 | 1.424 | 1.817 |
| FBgn0031713 | 0.995 | 0.995 | 0.906 | 0.991 | 1.034 | 1.183 | 1.417 | 1.739 | 0.991 | 1.034 | 1.183 | 1.417 | 1.739 | 1.014 | 1.012 | 1.162 | 1.557 | 1.685 |
| FBgn0032393 | 0.979 | 0.980 | 0.960 | 1.106 | 1.135 | 1.347 | 1.423 | 1.734 | 0.998 | 1.130 | 1.699 | 2.025 | 2.818 | 0.953 | 1.020 | 1.398 | 2.248 | 2.817 |
| FBgn0037020 | 0.988 | 0.943 | 0.958 | 0.914 | 0.938 | 0.953 | 1.001 | 1.089 | 0.897 | 0.927 | 1.089 | 1.107 | 1.298 | 0.978 | 0.996 | 0.993 | 1.100 | 1.229 |
| FBgn0051864 | 0.937 | 0.970 | 0.976 | 0.773 | 1.096 | 1.138 | 1.421 | 1.832 | 1.100 | 1.072 | 1.596 | 1.711 | 2.806 | 1.061 | 1.066 | 1.287 | 2.116 | 2.943 |

| Supplemental Table 2A. Gene Ontologies Overrepresented in Genes Found to Behave Linearly at Day 2 Post-Irradiation. (Analysis with all data included.) | | | | |
| --- | --- | --- | --- | --- |
|  |  |  |  |  |
| GO | Count | Total | P Value | GO Name |
| GO:0008605 | 13 | 23 | 2.21E-24 | protein kinase CK2 regulator activity |
| GO:0005956 | 13 | 23 | 2.21E-24 | protein kinase CK2 complex |
| GO:0043549 | 12 | 35 | 2.19E-19 | regulation of kinase activity |
| GO:0051338 | 12 | 35 | 2.19E-19 | regulation of transferase activity |
| GO:0045859 | 12 | 35 | 2.19E-19 | regulation of protein kinase activity |
| GO:0019887 | 13 | 53 | 5.02E-19 | protein kinase regulator activity |
| GO:0019207 | 13 | 55 | 7.39E-19 | kinase regulator activity |
| GO:0050790 | 12 | 57 | 1.10E-16 | regulation of catalytic activity |
| GO:0065009 | 12 | 70 | 1.41E-15 | regulation of a molecular function |
| GO:0007283 | 10 | 150 | 1.13E-08 | spermatogenesis |
| GO:0048232 | 10 | 150 | 1.13E-08 | male gamete generation |
| GO:0043234 | 19 | 1371 | 8.22E-08 | protein complex |
| GO:0032991 | 20 | 1652 | 1.45E-06 | macromolecular complex |
| GO:0007276 | 11 | 704 | 0.00244 | gamete generation |
| GO:0019953 | 11 | 730 | 0.00307 | sexual reproduction |
| GO:0065007 | 15 | 1459 | 0.00307 | biological regulation |
| GO:0044464 | 30 | 4358 | 0.00372 | cell#cell part |
| GO:0005623 | 30 | 4358 | 0.00372 | cell |
| GO:0005737 | 16 | 1833 | 0.0213 | cytoplasm |
| GO:0044459 | 6 | 305 | 0.0303 | plasma membrane part |
| GO:0035147 | 2 | 14 | 0.0311 | branch fusion, open tracheal system |
| GO:0035146 | 2 | 14 | 0.0311 | branching morphogenesis of a tube#tube fusion |
| GO:0005634 | 13 | 1420 | 0.0416 | nucleus |

| Supplemental Table 2B. Gene Ontologies Overrepresented in Genes Found to Behave Linearly at Day 10 Post-Irradiation. (Analysis with all data included.) | | | | |
| --- | --- | --- | --- | --- |
|  |  |  |  |  |
| GO | Count | Total | P Value | GO Name |
| GO:0008083 | 6 | 17 | 0.0333 | growth factor activity |
| GO:0016491 | 38 | 541 | 0.0333 | oxidoreductase activity |

| Supplemental Table 2C. Gene Ontologies Overrepresented in Genes Found to Behave Linearly at Day 20 Post-Irradiation. (Analysis with all data included.) | | | | |
| --- | --- | --- | --- | --- |
|  |  |  |  |  |
| GO | Count | Total | P Value | GO Name |
| GO:0016021 | 33 | 608 | 0.00106 | integral to membrane |
| GO:0031224 | 33 | 613 | 0.00106 | intrinsic to membrane |
| GO:0016020 | 58 | 1367 | 0.0016 | membrane |
| GO:0006950 | 15 | 211 | 0.0069 | response to stress |
| GO:0044459 | 19 | 305 | 0.0069 | plasma membrane part |
| GO:0005887 | 14 | 169 | 0.0115 | integral to plasma membrane |
| GO:0044425 | 43 | 1002 | 0.0115 | membrane#membrane part |
| GO:0031226 | 14 | 172 | 0.0115 | intrinsic to plasma membrane |
| GO:0004872 | 23 | 424 | 0.0115 | receptor activity |
| GO:0004888 | 20 | 355 | 0.0139 | transmembrane receptor activity |
| GO:0004871 | 26 | 517 | 0.0139 | signal transducer activity |
| GO:0060089 | 26 | 517 | 0.0139 | molecular transducer activity |
| GO:0009595 | 3 | 5 | 0.0139 | detection of biotic stimulus |
| GO:0016045 | 3 | 5 | 0.0139 | detection of bacterium |
| GO:0030425 | 4 | 12 | 0.0139 | dendrite |
| GO:0006281 | 8 | 67 | 0.0164 | DNA repair |
| GO:0001871 | 6 | 36 | 0.0164 | pattern binding |
| GO:0042834 | 4 | 13 | 0.0164 | peptidoglycan binding |
| GO:0000270 | 3 | 7 | 0.0353 | peptidoglycan metabolic process |
| GO:0006974 | 8 | 79 | 0.0448 | response to DNA damage stimulus |

| Supplemental Table 2D. Gene Ontologies Overrepresented in Genes Found to Behave Linearly at Days 2 and 10 Post-Irradiation. (Analysis with all data included.) | | | | |
| --- | --- | --- | --- | --- |
|  |  |  |  |  |
| GO | Count | Total | P Value | GO Name |
| GO:0004123 | 1 | 2 | 0.0243 | cystathionine gamma-lyase activity |
| GO:0006625 | 1 | 2 | 0.0243 | protein targeting to peroxisome |
| GO:0016226 | 1 | 2 | 0.0243 | iron-sulfur cluster assembly |
| GO:0043574 | 1 | 2 | 0.0243 | peroxisomal transport |
| GO:0031163 | 1 | 2 | 0.0243 | metallo-sulfur cluster assembly |
| GO:0004090 | 1 | 3 | 0.0331 | carbonyl reductase (NADPH) activity |

| Supplemental Table 2E. Gene Ontologies Overrepresented in Genes Found to Behave Linearly at Days 10 and 20 Post-Irradiation. (Analysis with all data included.) | | | | |
| --- | --- | --- | --- | --- |
|  |  |  |  |  |
| GO | Count | Total | P Value | GO Name |
| GO:0006950 | 9 | 211 | 0.00111 | response to stress |

| Supplemental Table 2F. Gene Ontologies Overrepresented in Genes Found to Behave Linearly at Days 2 and 20 Post-Irradiation. (Analysis with all data included.) | | | | |
| --- | --- | --- | --- | --- |
|  |  |  |  |  |
| GO | Count | Total | P Value | GO Name |
| GO:0004123 | 1 | 2 | 0.00745 | cystathionine gamma-lyase activity |
| GO:0006625 | 1 | 2 | 0.00745 | protein targeting to peroxisome |
| GO:0016226 | 1 | 2 | 0.00745 | iron-sulfur cluster assembly |
| GO:0043574 | 1 | 2 | 0.00745 | peroxisomal transport |
| GO:0031163 | 1 | 2 | 0.00745 | metallo-sulfur cluster assembly |
| GO:0016846 | 1 | 6 | 0.0206 | carbon-sulfur lyase activity |
| GO:0001666 | 1 | 9 | 0.0268 | response to hypoxia |
| GO:0032200 | 1 | 10 | 0.0268 | telomere organization and biogenesis |
| GO:0000723 | 1 | 10 | 0.0268 | telomere maintenance |
| GO:0044439 | 1 | 12 | 0.0268 | peroxisomal part |
| GO:0044438 | 1 | 12 | 0.0268 | microbody#microbody part |
| GO:0031903 | 1 | 12 | 0.0268 | microbody membrane |
| GO:0005778 | 1 | 12 | 0.0268 | peroxisomal membrane |
| GO:0007031 | 1 | 13 | 0.0276 | peroxisome organization and biogenesis |
| GO:0008652 | 1 | 20 | 0.0388 | amino acid biosynthetic process |
| GO:0004003 | 1 | 20 | 0.0388 | ATP-dependent DNA helicase activity |

| Supplemental Table 2G. Gene Ontologies Overrepresented in Genes Found to Behave Linearly at Days 2, 10 and 20 Post-Irradiation. (Analysis with all data included.) | | | | |
| --- | --- | --- | --- | --- |
|  |  |  |  |  |
| GO | Count | Total | P Value | GO Name |
| GO:0004123 | 1 | 2 | 0.00517 | cystathionine gamma-lyase activity |
| GO:0006625 | 1 | 2 | 0.00517 | protein targeting to peroxisome |
| GO:0016226 | 1 | 2 | 0.00517 | iron-sulfur cluster assembly |
| GO:0043574 | 1 | 2 | 0.00517 | peroxisomal transport |
| GO:0031163 | 1 | 2 | 0.00517 | metallo-sulfur cluster assembly |
| GO:0016846 | 1 | 6 | 0.0141 | carbon-sulfur lyase activity |
| GO:0032200 | 1 | 10 | 0.0182 | telomere organization and biogenesis |
| GO:0000723 | 1 | 10 | 0.0182 | telomere maintenance |
| GO:0044439 | 1 | 12 | 0.0182 | peroxisomal part |
| GO:0044438 | 1 | 12 | 0.0182 | microbody#microbody part |
| GO:0031903 | 1 | 12 | 0.0182 | microbody membrane |
| GO:0005778 | 1 | 12 | 0.0182 | peroxisomal membrane |
| GO:0007031 | 1 | 13 | 0.0187 | peroxisome organization and biogenesis |
| GO:0008652 | 1 | 20 | 0.0258 | amino acid biosynthetic process |
| GO:0004003 | 1 | 20 | 0.0258 | ATP-dependent DNA helicase activity |
| GO:0008094 | 1 | 29 | 0.0331 | DNA-dependent ATPase activity |
| GO:0042579 | 1 | 31 | 0.0331 | microbody |
| GO:0005777 | 1 | 31 | 0.0331 | peroxisome |
| GO:0009309 | 1 | 34 | 0.0331 | amine biosynthetic process |
| GO:0044271 | 1 | 35 | 0.0331 | nitrogen compound biosynthetic process |
| GO:0043231 | 3 | 2283 | 0.0331 | intracellular membrane-bound organelle |
| GO:0043227 | 3 | 2287 | 0.0331 | membrane-bound organelle |
| GO:0003678 | 1 | 36 | 0.0331 | DNA helicase activity |

| Supplemental Table 3A. Values of R Squared and Fold Changes for Genes Found to Behave Linearly at Day 2 Post-Irradiation. (Analysis with all lowest dose discluded.) | | | | | |
| --- | --- | --- | --- | --- | --- |
|  |  |  |  |  |  |
| Flybase ID | R Squared Value Day2 | Day 2 Fold Change 1000R | Day 2 Fold Change 5000R | Day 2 Fold Change 10000R | Day 2 Fold Change 20000R |
| FBgn0050349 | 1.000 | 0.962 | 0.946 | 0.927 | 0.886 |
| FBgn0030511 | 1.000 | 1.134 | 1.176 | 1.230 | 1.343 |
| FBgn0034499 | 1.000 | 0.912 | 0.844 | 0.748 | 0.576 |
| FBgn0033955 | 0.999 | 1.099 | 1.037 | 0.958 | 0.786 |
| FBgn0036763 | 0.999 | 1.024 | 0.995 | 0.958 | 0.876 |
| FBgn0040283 | 0.999 | 0.979 | 0.927 | 0.870 | 0.759 |
| FBgn0037225 | 0.999 | 1.626 | 2.451 | 3.272 | 5.209 |
| FBgn0037654 | 0.999 | 0.968 | 0.922 | 0.855 | 0.716 |
| FBgn0044328 | 0.999 | 1.068 | 1.040 | 1.001 | 0.935 |
| FBgn0038952 | 0.998 | 1.096 | 1.068 | 1.034 | 0.973 |
| FBgn0050222 | 0.998 | 1.028 | 0.901 | 0.785 | 0.505 |
| FBgn0031265 | 0.998 | 0.986 | 0.940 | 0.867 | 0.720 |
| FBgn0038073 | 0.998 | 0.980 | 0.954 | 0.928 | 0.875 |
| FBgn0043578 | 0.998 | 0.595 | 0.574 | 0.556 | 0.510 |
| FBgn0038008 | 0.998 | 1.070 | 0.991 | 0.875 | 0.626 |
| FBgn0033473 | 0.998 | 0.996 | 0.969 | 0.920 | 0.834 |
| FBgn0024234 | 0.998 | 1.031 | 1.074 | 1.154 | 1.286 |
| FBgn0031233 | 0.997 | 1.047 | 1.008 | 0.973 | 0.897 |
| FBgn0004406 | 0.997 | 1.026 | 1.005 | 0.969 | 0.913 |
| FBgn0033819 | 0.997 | 1.008 | 0.966 | 0.915 | 0.789 |
| FBgn0022800 | 0.997 | 1.157 | 1.096 | 1.047 | 0.915 |
| FBgn0051921 | 0.996 | 0.984 | 0.950 | 0.881 | 0.774 |
| FBgn0028542 | 0.996 | 1.058 | 1.108 | 1.205 | 1.350 |
| FBgn0039629 | 0.996 | 0.803 | 0.715 | 0.612 | 0.342 |
| FBgn0023197 | 0.996 | 0.962 | 1.017 | 1.111 | 1.247 |
| FBgn0033056 | 0.996 | 0.997 | 0.953 | 0.860 | 0.699 |
| FBgn0032521 | 0.996 | 1.013 | 0.991 | 0.943 | 0.862 |
| FBgn0032883 | 0.995 | 1.041 | 0.997 | 0.921 | 0.813 |
| FBgn0053247 | 0.995 | 0.481 | 0.696 | 1.008 | 1.754 |
| FBgn0033397 | 0.995 | 0.881 | 0.912 | 0.981 | 1.102 |
| FBgn0052110 | 0.995 | 0.794 | 0.746 | 0.646 | 0.502 |
| FBgn0035078 | 0.995 | 1.025 | 0.949 | 0.895 | 0.735 |
| FBgn0259817 | 0.995 | 0.713 | 0.809 | 0.952 | 1.293 |
| FBgn0038139 | 0.994 | 1.613 | 1.522 | 1.310 | 0.941 |
| FBgn0033188 | 0.994 | 1.038 | 1.077 | 1.160 | 1.276 |
| FBgn0053140 | 0.994 | 1.052 | 0.953 | 0.876 | 0.633 |
| FBgn0053993 | 0.994 | 0.886 | 0.988 | 1.123 | 1.320 |
| FBgn0022770 | 0.994 | 1.246 | 1.286 | 1.384 | 1.535 |
| FBgn0004654 | 0.994 | 1.045 | 1.089 | 1.124 | 1.201 |
| FBgn0053237 | 0.993 | 0.454 | 0.697 | 0.941 | 1.669 |
| FBgn0020617 | 0.993 | 1.258 | 1.321 | 1.395 | 1.602 |
| FBgn0034998 | 0.993 | 0.900 | 0.824 | 0.776 | 0.623 |
| FBgn0035800 | 0.992 | 0.991 | 0.887 | 0.815 | 0.571 |
| FBgn0051803 | 0.992 | 0.889 | 0.839 | 0.777 | 0.688 |
| FBgn0008635 | 0.992 | 1.033 | 1.058 | 1.078 | 1.145 |
| FBgn0051361 | 0.992 | 1.010 | 0.986 | 0.927 | 0.816 |
| FBgn0032626 | 0.992 | 0.999 | 0.951 | 0.845 | 0.710 |
| FBgn0023169 | 0.992 | 1.075 | 1.082 | 1.103 | 1.134 |
| FBgn0039773 | 0.991 | 0.930 | 0.903 | 0.884 | 0.816 |
| FBgn0053243 | 0.991 | 0.459 | 0.701 | 0.925 | 1.659 |
| FBgn0063485 | 0.991 | 1.028 | 1.017 | 1.010 | 0.993 |
| FBgn0030852 | 0.991 | 1.023 | 0.976 | 0.941 | 0.818 |
| FBgn0011725 | 0.991 | 1.024 | 0.984 | 0.936 | 0.794 |
| FBgn0032857 | 0.990 | 1.157 | 1.122 | 1.093 | 0.992 |
| FBgn0031659 | 0.990 | 1.011 | 0.989 | 0.952 | 0.906 |
| FBgn0034289 | 0.990 | 0.705 | 0.769 | 0.826 | 1.021 |
| FBgn0032488 | 0.989 | 1.053 | 1.034 | 0.984 | 0.922 |
| FBgn0039343 | 0.989 | 0.923 | 0.840 | 0.788 | 0.653 |
| FBgn0031713 | 0.989 | 1.021 | 1.110 | 1.158 | 1.324 |
| FBgn0039376 | 0.989 | 1.030 | 1.062 | 1.082 | 1.135 |
| FBgn0031235 | 0.989 | 1.149 | 1.073 | 0.963 | 0.656 |
| FBgn0002715 | 0.989 | 0.889 | 0.845 | 0.809 | 0.742 |
| FBgn0031935 | 0.988 | 1.088 | 1.024 | 0.827 | 0.477 |
| FBgn0038558 | 0.988 | 0.894 | 0.836 | 0.775 | 0.575 |
| FBgn0003889 | 0.988 | 1.005 | 0.967 | 0.931 | 0.872 |
| FBgn0029989 | 0.988 | 1.048 | 1.002 | 0.958 | 0.807 |
| FBgn0053238 | 0.988 | 0.450 | 0.693 | 0.905 | 1.665 |
| FBgn0032974 | 0.987 | 1.007 | 0.931 | 0.891 | 0.723 |
| FBgn0039812 | 0.987 | 0.968 | 0.906 | 0.805 | 0.685 |
| FBgn0029002 | 0.987 | 0.769 | 0.800 | 0.821 | 0.905 |
| FBgn0029133 | 0.987 | 1.008 | 0.964 | 0.922 | 0.856 |
| FBgn0037020 | 0.986 | 0.938 | 0.953 | 1.001 | 1.089 |
| FBgn0030787 | 0.986 | 0.930 | 0.921 | 0.898 | 0.847 |
| FBgn0053240 | 0.986 | 0.456 | 0.777 | 0.930 | 1.584 |
| FBgn0050271 | 0.986 | 1.013 | 0.989 | 0.932 | 0.799 |
| FBgn0029702 | 0.985 | 0.980 | 0.936 | 0.907 | 0.787 |
| FBgn0053241 | 0.985 | 0.442 | 0.706 | 0.881 | 1.619 |
| FBgn0036031 | 0.985 | 1.021 | 0.986 | 0.970 | 0.913 |
| FBgn0037652 | 0.984 | 1.117 | 1.089 | 0.971 | 0.804 |
| FBgn0030507 | 0.984 | 0.963 | 1.195 | 1.540 | 1.943 |
| FBgn0036702 | 0.984 | 1.084 | 1.097 | 1.118 | 1.142 |
| FBgn0053244 | 0.984 | 0.457 | 0.798 | 0.945 | 1.589 |
| FBgn0038575 | 0.984 | 1.035 | 1.008 | 0.916 | 0.810 |
| FBgn0053245 | 0.983 | 0.459 | 0.798 | 0.945 | 1.611 |
| FBgn0051627 | 0.983 | 1.256 | 1.151 | 0.990 | 0.507 |
| FBgn0053236 | 0.983 | 0.457 | 0.800 | 0.945 | 1.600 |
| FBgn0015546 | 0.983 | 0.846 | 0.772 | 0.679 | 0.565 |
| FBgn0004666 | 0.982 | 1.043 | 1.193 | 1.281 | 1.494 |
| FBgn0033367 | 0.982 | 0.972 | 1.094 | 1.400 | 1.724 |
| FBgn0051702 | 0.982 | 1.111 | 1.059 | 0.919 | 0.597 |
| FBgn0037419 | 0.982 | 0.806 | 0.847 | 1.013 | 1.208 |
| FBgn0083987 | 0.982 | 0.626 | 0.690 | 0.847 | 1.010 |
| FBgn0035379 | 0.982 | 1.085 | 1.106 | 1.151 | 1.198 |
| FBgn0037555 | 0.982 | 1.099 | 1.110 | 1.166 | 1.244 |
| FBgn0053096 | 0.981 | 1.076 | 1.006 | 0.943 | 0.847 |
| FBgn0262109 | 0.981 | 1.038 | 1.023 | 0.986 | 0.897 |
| FBgn0031664 | 0.981 | 1.064 | 1.017 | 0.942 | 0.860 |
| FBgn0011823 | 0.981 | 0.993 | 0.983 | 0.977 | 0.947 |
| FBgn0053246 | 0.981 | 0.421 | 0.713 | 0.877 | 1.677 |
| FBgn0036652 | 0.981 | 1.075 | 1.044 | 0.987 | 0.927 |
| FBgn0039417 | 0.981 | 0.969 | 1.137 | 1.454 | 2.349 |
| FBgn0053242 | 0.981 | 0.472 | 0.806 | 0.936 | 1.559 |
| FBgn0053239 | 0.980 | 0.450 | 0.776 | 0.908 | 1.604 |
| FBgn0038208 | 0.980 | 1.026 | 1.006 | 0.896 | 0.726 |
| FBgn0035838 | 0.980 | 0.974 | 0.902 | 0.829 | 0.730 |
| FBgn0013432 | 0.979 | 0.859 | 0.917 | 0.980 | 1.215 |
| FBgn0085454 | 0.979 | 1.027 | 0.945 | 0.851 | 0.735 |
| FBgn0050384 | 0.979 | 1.084 | 0.984 | 0.948 | 0.767 |
| FBgn0038612 | 0.978 | 0.758 | 0.671 | 0.555 | 0.424 |
| FBgn0031945 | 0.978 | 0.902 | 0.918 | 1.005 | 1.158 |
| FBgn0035262 | 0.978 | 1.041 | 1.017 | 0.969 | 0.831 |
| FBgn0028844 | 0.978 | 0.944 | 0.889 | 0.785 | 0.681 |
| FBgn0051864 | 0.978 | 1.096 | 1.138 | 1.421 | 1.832 |
| FBgn0052823 | 0.978 | 0.987 | 0.994 | 1.021 | 1.049 |
| FBgn0033818 | 0.977 | 0.914 | 0.878 | 0.797 | 0.574 |
| FBgn0261596 | 0.977 | 0.950 | 0.973 | 1.035 | 1.096 |
| FBgn0028675 | 0.977 | 1.051 | 1.236 | 1.315 | 1.775 |
| FBgn0015615 | 0.977 | 0.954 | 0.906 | 0.884 | 0.817 |
| FBgn0035107 | 0.977 | 1.008 | 1.027 | 1.089 | 1.150 |
| FBgn0000667 | 0.977 | 1.059 | 1.069 | 1.140 | 1.242 |
| FBgn0039755 | 0.977 | 0.983 | 0.989 | 1.033 | 1.100 |
| FBgn0033392 | 0.977 | 1.075 | 1.022 | 0.951 | 0.874 |
| FBgn0003963 | 0.976 | 1.027 | 1.048 | 1.056 | 1.100 |
| FBgn0051029 | 0.976 | 1.027 | 0.984 | 0.898 | 0.816 |
| FBgn0039659 | 0.976 | 0.814 | 0.746 | 0.700 | 0.475 |
| FBgn0037531 | 0.976 | 0.855 | 0.918 | 0.985 | 1.252 |
| FBgn0025381 | 0.976 | 1.016 | 1.060 | 1.092 | 1.147 |
| FBgn0086604 | 0.975 | 1.080 | 1.102 | 1.158 | 1.210 |
| FBgn0050432 | 0.975 | 1.085 | 0.844 | 0.764 | 0.374 |
| FBgn0050357 | 0.975 | 1.072 | 0.983 | 0.933 | 0.665 |
| FBgn0002673 | 0.974 | 0.968 | 0.955 | 0.847 | 0.707 |
| FBgn0032393 | 0.974 | 1.135 | 1.347 | 1.423 | 1.734 |
| FBgn0016013 | 0.974 | 1.086 | 1.046 | 1.007 | 0.841 |
| FBgn0037916 | 0.974 | 0.817 | 0.773 | 0.643 | 0.523 |
| FBgn0032448 | 0.974 | 0.814 | 0.771 | 0.734 | 0.681 |
| FBgn0027575 | 0.974 | 1.011 | 1.023 | 1.056 | 1.087 |
| FBgn0036443 | 0.973 | 0.993 | 0.943 | 0.826 | 0.490 |
| FBgn0053971 | 0.973 | 0.986 | 1.070 | 1.099 | 1.224 |
| FBgn0024732 | 0.973 | 0.974 | 0.998 | 1.099 | 1.198 |
| FBgn0023496 | 0.973 | 0.946 | 0.990 | 1.345 | 1.956 |
| FBgn0003900 | 0.973 | 1.179 | 1.062 | 1.025 | 0.846 |
| FBgn0050203 | 0.972 | 1.293 | 1.202 | 1.167 | 0.930 |
| FBgn0031335 | 0.972 | 1.011 | 0.992 | 0.860 | 0.714 |
| FBgn0004087 | 0.972 | 1.113 | 1.105 | 1.064 | 0.975 |
| FBgn0000615 | 0.970 | 0.986 | 0.954 | 0.929 | 0.806 |
| FBgn0010238 | 0.970 | 1.001 | 1.008 | 1.086 | 1.215 |
| FBgn0053926 | 0.970 | 1.238 | 1.384 | 1.795 | 2.153 |
| FBgn0033860 | 0.970 | 1.098 | 1.086 | 0.965 | 0.825 |
| FBgn0026257 | 0.969 | 1.164 | 1.049 | 0.960 | 0.826 |
| FBgn0030884 | 0.969 | 1.010 | 0.965 | 0.954 | 0.874 |
| FBgn0029826 | 0.969 | 0.886 | 1.006 | 1.131 | 1.277 |
| FBgn0011774 | 0.969 | 0.954 | 1.315 | 1.428 | 1.933 |
| FBgn0031313 | 0.968 | 1.049 | 1.113 | 1.136 | 1.219 |
| FBgn0042712 | 0.968 | 0.944 | 0.949 | 0.986 | 1.065 |
| FBgn0030234 | 0.968 | 1.153 | 1.162 | 1.301 | 1.512 |
| FBgn0050259 | 0.968 | 1.399 | 1.903 | 2.031 | 3.132 |
| FBgn0261502 | 0.968 | 0.781 | 0.764 | 0.571 | 0.355 |
| FBgn0004858 | 0.968 | 0.989 | 0.994 | 1.056 | 1.163 |
| FBgn0035426 | 0.967 | 0.943 | 0.929 | 0.652 | 0.269 |
| FBgn0033174 | 0.967 | 0.983 | 0.937 | 0.911 | 0.858 |
| FBgn0047000 | 0.967 | 1.051 | 1.297 | 1.431 | 1.713 |
| FBgn0032364 | 0.967 | 1.079 | 1.002 | 0.935 | 0.605 |
| FBgn0039709 | 0.967 | 1.065 | 1.111 | 1.825 | 2.675 |
| FBgn0036449 | 0.966 | 1.191 | 1.126 | 1.101 | 0.912 |
| FBgn0036040 | 0.966 | 1.157 | 1.381 | 1.518 | 1.770 |
| FBgn0020280 | 0.966 | 1.006 | 0.923 | 0.872 | 0.571 |
| FBgn0039380 | 0.966 | 1.160 | 1.001 | 0.940 | 0.743 |
| FBgn0039760 | 0.966 | 1.051 | 1.057 | 1.142 | 1.240 |
| FBgn0032486 | 0.966 | 1.034 | 1.027 | 1.006 | 0.945 |
| FBgn0016650 | 0.966 | 1.020 | 1.093 | 1.171 | 1.257 |
| FBgn0038373 | 0.965 | 1.012 | 1.007 | 0.954 | 0.897 |
| FBgn0005427 | 0.965 | 1.024 | 0.993 | 0.919 | 0.859 |
| FBgn0029831 | 0.965 | 1.055 | 0.985 | 0.969 | 0.862 |
| FBgn0000117 | 0.965 | 0.987 | 0.990 | 0.995 | 1.014 |
| FBgn0032079 | 0.964 | 1.101 | 1.094 | 0.964 | 0.817 |
| FBgn0038221 | 0.964 | 0.922 | 1.272 | 1.799 | 3.892 |
| FBgn0036290 | 0.963 | 0.797 | 0.975 | 1.110 | 1.303 |
| FBgn0041627 | 0.963 | 1.054 | 1.060 | 1.283 | 1.644 |
| FBgn0260962 | 0.963 | 0.996 | 0.936 | 0.924 | 0.791 |
| FBgn0020653 | 0.963 | 1.109 | 1.123 | 1.236 | 1.341 |
| FBgn0031832 | 0.963 | 1.032 | 1.076 | 1.092 | 1.222 |
| FBgn0034292 | 0.962 | 0.958 | 1.077 | 1.104 | 1.389 |
| FBgn0031724 | 0.962 | 1.032 | 1.015 | 0.895 | 0.787 |
| FBgn0036305 | 0.962 | 0.856 | 0.836 | 0.829 | 0.805 |
| FBgn0051920 | 0.962 | 0.963 | 0.957 | 0.861 | 0.760 |
| FBgn0044817 | 0.961 | 0.445 | 0.781 | 0.851 | 1.631 |
| FBgn0033087 | 0.961 | 1.042 | 1.038 | 1.000 | 0.915 |
| FBgn0023416 | 0.961 | 1.053 | 0.985 | 0.973 | 0.835 |
| FBgn0041342 | 0.961 | 1.128 | 1.122 | 1.084 | 1.053 |
| FBgn0085209 | 0.960 | 0.832 | 0.837 | 0.852 | 0.863 |
| FBgn0019662 | 0.960 | 0.962 | 1.021 | 1.030 | 1.138 |
| FBgn0261996 | 0.960 | 1.072 | 1.019 | 0.767 | 0.565 |
| FBgn0035471 | 0.959 | 0.969 | 0.993 | 0.997 | 1.051 |
| FBgn0039058 | 0.959 | 0.646 | 0.599 | 0.484 | 0.397 |
| FBgn0035077 | 0.959 | 1.030 | 1.011 | 0.962 | 0.793 |
| FBgn0002645 | 0.958 | 0.936 | 0.860 | 0.849 | 0.719 |
| FBgn0005322 | 0.958 | 1.018 | 0.963 | 0.900 | 0.840 |
| FBgn0032509 | 0.957 | 0.987 | 0.973 | 0.913 | 0.868 |
| FBgn0037891 | 0.957 | 1.060 | 1.059 | 1.097 | 1.155 |
| FBgn0020440 | 0.957 | 1.045 | 0.983 | 0.964 | 0.890 |
| FBgn0262518 | 0.957 | 1.006 | 1.002 | 0.942 | 0.886 |
| FBgn0051533 | 0.957 | 0.977 | 0.979 | 0.812 | 0.617 |
| FBgn0040099 | 0.956 | 0.737 | 0.810 | 0.831 | 0.917 |
| FBgn0015562 | 0.956 | 0.975 | 0.945 | 0.941 | 0.874 |
| FBgn0038252 | 0.956 | 0.777 | 0.660 | 0.464 | 0.310 |
| FBgn0041775 | 0.955 | 1.001 | 1.008 | 1.024 | 1.085 |
| FBgn0016123 | 0.955 | 1.013 | 1.092 | 1.126 | 1.210 |
| FBgn0032677 | 0.955 | 1.055 | 1.057 | 0.987 | 0.863 |
| FBgn0037837 | 0.955 | 1.479 | 1.278 | 1.243 | 0.744 |
| FBgn0036677 | 0.955 | 1.417 | 1.274 | 0.898 | 0.628 |
| FBgn0053017 | 0.954 | 1.066 | 0.984 | 0.974 | 0.824 |
| FBgn0051291 | 0.954 | 1.393 | 1.398 | 1.821 | 2.243 |
| FBgn0036778 | 0.954 | 1.091 | 1.051 | 1.043 | 0.938 |
| FBgn0034464 | 0.953 | 0.889 | 0.893 | 0.818 | 0.708 |
| FBgn0031161 | 0.953 | 1.062 | 1.035 | 0.961 | 0.909 |
| FBgn0037483 | 0.953 | 1.157 | 1.018 | 0.988 | 0.601 |
| FBgn0028406 | 0.953 | 1.159 | 1.135 | 0.985 | 0.870 |
| FBgn0031432 | 0.953 | 1.021 | 1.073 | 1.408 | 1.667 |
| FBgn0003654 | 0.953 | 0.982 | 0.999 | 1.001 | 1.039 |
| FBgn0027600 | 0.953 | 1.031 | 1.027 | 1.206 | 1.402 |
| FBgn0260481 | 0.953 | 1.047 | 1.011 | 0.874 | 0.776 |
| FBgn0037883 | 0.953 | 1.022 | 1.083 | 1.362 | 1.565 |
| FBgn0031372 | 0.952 | 0.916 | 0.865 | 0.832 | 0.603 |
| FBgn0003279 | 0.952 | 1.118 | 1.193 | 1.236 | 1.310 |
| FBgn0036482 | 0.952 | 1.004 | 0.963 | 0.929 | 0.723 |
| FBgn0034184 | 0.952 | 1.033 | 1.036 | 1.039 | 1.055 |
| FBgn0030441 | 0.951 | 1.041 | 0.983 | 0.965 | 0.772 |
| FBgn0038220 | 0.951 | 0.981 | 0.996 | 1.061 | 1.107 |
| FBgn0029791 | 0.951 | 1.167 | 1.170 | 1.342 | 1.504 |
| FBgn0038199 | 0.951 | 0.869 | 0.923 | 4.066 | 11.157 |
| FBgn0029679 | 0.950 | 1.073 | 1.002 | 0.968 | 0.898 |
| FBgn0050496 | 0.950 | 1.058 | 1.051 | 0.978 | 0.782 |
| FBgn0262029 | 0.950 | 0.967 | 1.074 | 1.091 | 1.376 |
| FBgn0034053 | 0.950 | 1.016 | 0.991 | 0.861 | 0.767 |
| FBgn0033149 | 0.950 | 1.056 | 0.966 | 0.959 | 0.791 |
| FBgn0263143 | 0.949 | 0.789 | 0.798 | 0.664 | 0.445 |
| FBgn0028855 | 0.949 | 0.737 | 0.664 | 0.655 | 0.553 |
| FBgn0034098 | 0.949 | 0.993 | 0.948 | 0.881 | 0.561 |
| FBgn0053499 | 0.948 | 0.996 | 1.018 | 1.020 | 1.057 |
| FBgn0030026 | 0.948 | 1.122 | 1.183 | 1.213 | 1.467 |
| FBgn0033069 | 0.948 | 0.931 | 0.840 | 0.758 | 0.672 |
| FBgn0038536 | 0.948 | 0.741 | 0.780 | 0.800 | 0.967 |
| FBgn0036875 | 0.948 | 1.050 | 1.037 | 1.033 | 0.988 |
| FBgn0030876 | 0.948 | 1.021 | 1.169 | 1.262 | 1.400 |
| FBgn0261267 | 0.948 | 0.732 | 0.887 | 0.913 | 1.111 |
| FBgn0019686 | 0.947 | 0.902 | 0.880 | 0.843 | 0.672 |
| FBgn0039486 | 0.947 | 0.984 | 0.968 | 0.966 | 0.940 |
| FBgn0037922 | 0.947 | 1.061 | 1.066 | 0.957 | 0.840 |
| FBgn0261850 | 0.947 | 0.969 | 0.897 | 0.865 | 0.794 |
| FBgn0033686 | 0.947 | 0.860 | 0.863 | 0.803 | 0.687 |
| FBgn0017566 | 0.947 | 1.109 | 1.106 | 1.174 | 1.242 |
| FBgn0030246 | 0.947 | 1.129 | 1.129 | 1.265 | 1.387 |
| FBgn0024912 | 0.947 | 1.391 | 1.592 | 1.717 | 1.904 |
| FBgn0004374 | 0.946 | 1.001 | 1.042 | 1.052 | 1.097 |
| FBgn0030041 | 0.946 | 1.129 | 1.117 | 1.093 | 0.989 |
| FBgn0038829 | 0.946 | 0.911 | 0.902 | 0.994 | 1.130 |
| FBgn0262690 | 0.946 | 1.104 | 0.860 | 0.661 | 0.437 |
| FBgn0052087 | 0.946 | 1.050 | 1.025 | 0.895 | 0.808 |
| FBgn0051703 | 0.946 | 1.024 | 0.947 | 0.913 | 0.596 |
| FBgn0011207 | 0.945 | 0.933 | 0.932 | 0.982 | 1.029 |
| FBgn0027491 | 0.945 | 1.068 | 1.073 | 1.001 | 0.918 |
| FBgn0261049 | 0.945 | 1.027 | 0.944 | 0.934 | 0.823 |
| FBgn0041184 | 0.945 | 0.921 | 1.060 | 1.070 | 1.286 |
| FBgn0040022 | 0.945 | 1.342 | 1.354 | 1.229 | 1.051 |
| FBgn0260004 | 0.945 | 0.982 | 0.984 | 1.064 | 1.130 |
| FBgn0026593 | 0.945 | 1.015 | 1.062 | 1.070 | 1.209 |
| FBgn0033348 | 0.945 | 0.837 | 0.834 | 0.833 | 0.824 |
| FBgn0030207 | 0.944 | 1.116 | 1.121 | 1.156 | 1.265 |
| FBgn0027836 | 0.944 | 1.053 | 1.056 | 1.121 | 1.171 |
| FBgn0003517 | 0.944 | 1.005 | 1.029 | 1.030 | 1.077 |
| FBgn0262728 | 0.944 | 0.978 | 1.006 | 1.008 | 1.052 |
| FBgn0019957 | 0.944 | 1.024 | 1.058 | 1.077 | 1.108 |
| FBgn0035004 | 0.944 | 1.081 | 0.874 | 0.847 | 0.584 |
| FBgn0053116 | 0.943 | 0.820 | 0.787 | 0.784 | 0.738 |
| FBgn0039886 | 0.943 | 1.050 | 0.999 | 0.994 | 0.857 |
| FBgn0035707 | 0.943 | 1.057 | 1.051 | 0.998 | 0.836 |
| FBgn0000064 | 0.943 | 1.056 | 1.125 | 1.144 | 1.216 |
| FBgn0046763 | 0.943 | 0.940 | 0.958 | 1.078 | 1.159 |
| FBgn0052751 | 0.942 | 1.641 | 1.356 | 1.179 | 0.925 |
| FBgn0261243 | 0.942 | 1.025 | 0.947 | 0.929 | 0.845 |
| FBgn0031240 | 0.942 | 1.026 | 1.012 | 0.934 | 0.666 |
| FBgn0262097 | 0.942 | 1.228 | 1.145 | 0.905 | 0.756 |
| FBgn0031497 | 0.942 | 1.042 | 1.030 | 1.141 | 1.286 |
| FBgn0037850 | 0.941 | 1.320 | 2.346 | 2.565 | 3.692 |
| FBgn0086442 | 0.941 | 1.073 | 1.022 | 1.014 | 0.953 |
| FBgn0027610 | 0.941 | 0.984 | 0.977 | 1.035 | 1.111 |
| FBgn0259192 | 0.940 | 0.677 | 0.722 | 0.817 | 0.878 |
| FBgn0038143 | 0.940 | 0.979 | 0.942 | 0.941 | 0.883 |
| FBgn0033927 | 0.940 | 1.217 | 1.469 | 1.907 | 4.087 |
| FBgn0020521 | 0.940 | 1.092 | 1.087 | 1.208 | 1.316 |
| FBgn0039065 | 0.940 | 0.994 | 0.875 | 0.855 | 0.717 |
| FBgn0030294 | 0.940 | 0.929 | 0.985 | 1.020 | 1.069 |
| FBgn0033792 | 0.940 | 1.509 | 1.477 | 1.357 | 0.895 |
| FBgn0032375 | 0.940 | 0.876 | 0.868 | 0.823 | 0.666 |
| FBgn0053497 | 0.939 | 0.996 | 1.020 | 1.021 | 1.058 |
| FBgn0037164 | 0.938 | 0.956 | 0.967 | 0.865 | 0.748 |
| FBgn0036179 | 0.938 | 1.055 | 1.089 | 1.151 | 1.460 |
| FBgn0030189 | 0.938 | 0.777 | 0.848 | 1.010 | 1.108 |
| FBgn0038583 | 0.938 | 0.883 | 0.794 | 0.726 | 0.649 |
| FBgn0033495 | 0.938 | 1.051 | 1.006 | 0.967 | 0.928 |
| FBgn0037305 | 0.938 | 0.987 | 0.990 | 0.967 | 0.927 |
| FBgn0023001 | 0.938 | 1.004 | 1.008 | 1.106 | 1.177 |
| FBgn0053742 | 0.937 | 0.829 | 0.634 | 0.559 | 0.378 |
| FBgn0053743 | 0.937 | 0.829 | 0.634 | 0.559 | 0.378 |
| FBgn0053744 | 0.937 | 0.829 | 0.634 | 0.559 | 0.378 |
| FBgn0052206 | 0.937 | 1.136 | 1.129 | 1.099 | 0.984 |
| FBgn0002780 | 0.937 | 0.820 | 0.814 | 0.781 | 0.662 |
| FBgn0031490 | 0.937 | 0.994 | 1.163 | 1.161 | 1.499 |
| FBgn0028852 | 0.936 | 0.792 | 0.685 | 0.651 | 0.549 |
| FBgn0031504 | 0.936 | 1.108 | 1.062 | 0.756 | 0.564 |
| FBgn0014863 | 0.936 | 1.310 | 1.348 | 1.347 | 1.413 |
| FBgn0038029 | 0.936 | 1.116 | 1.136 | 1.212 | 1.518 |
| FBgn0004892 | 0.936 | 1.088 | 1.219 | 1.218 | 1.450 |
| FBgn0033028 | 0.936 | 1.090 | 0.979 | 0.975 | 0.687 |
| FBgn0038170 | 0.936 | 1.114 | 1.048 | 0.993 | 0.588 |
| FBgn0023541 | 0.935 | 0.973 | 0.901 | 0.902 | 0.753 |
| FBgn0036881 | 0.935 | 1.074 | 1.046 | 1.350 | 1.646 |
| FBgn0051266 | 0.935 | 1.068 | 1.070 | 1.137 | 1.187 |
| FBgn0050487 | 0.935 | 1.863 | 1.918 | 1.549 | 0.893 |
| FBgn0033427 | 0.935 | 1.074 | 0.907 | 0.860 | 0.699 |
| FBgn0036895 | 0.935 | 1.062 | 1.071 | 0.972 | 0.744 |
| FBgn0260010 | 0.934 | 0.956 | 0.911 | 0.839 | 0.793 |
| FBgn0034317 | 0.934 | 0.422 | 1.015 | 1.055 | 1.814 |
| FBgn0034827 | 0.934 | 1.002 | 0.863 | 0.847 | 0.685 |
| FBgn0036223 | 0.934 | 1.119 | 1.144 | 0.988 | 0.741 |
| FBgn0038701 | 0.934 | 1.118 | 1.071 | 1.068 | 1.007 |
| FBgn0036415 | 0.934 | 0.887 | 0.761 | 0.627 | 0.518 |
| FBgn0024989 | 0.933 | 1.200 | 1.501 | 1.743 | 3.594 |
| FBgn0039303 | 0.933 | 0.937 | 0.985 | 0.986 | 1.057 |
| FBgn0032666 | 0.933 | 0.987 | 0.946 | 0.947 | 0.858 |
| FBgn0036962 | 0.933 | 0.997 | 0.924 | 0.914 | 0.833 |
| FBgn0015230 | 0.933 | 1.071 | 1.073 | 0.937 | 0.835 |
| FBgn0036892 | 0.932 | 0.998 | 0.963 | 0.953 | 0.807 |
| FBgn0031347 | 0.932 | 0.972 | 0.956 | 0.844 | 0.777 |
| FBgn0014009 | 0.932 | 0.971 | 0.968 | 1.017 | 1.060 |
| FBgn0000542 | 0.932 | 0.911 | 0.954 | 1.022 | 1.064 |
| FBgn0035134 | 0.932 | 0.790 | 0.933 | 0.944 | 1.392 |
| FBgn0085360 | 0.932 | 0.928 | 1.022 | 1.115 | 1.193 |
| FBgn0001145 | 0.931 | 1.019 | 1.015 | 1.040 | 1.088 |
| FBgn0033926 | 0.931 | 1.108 | 1.534 | 1.668 | 3.537 |
| FBgn0031888 | 0.930 | 1.018 | 1.225 | 1.216 | 1.592 |
| FBgn0039705 | 0.930 | 0.997 | 0.945 | 0.940 | 0.877 |
| FBgn0036678 | 0.930 | 0.919 | 0.939 | 0.831 | 0.667 |
| FBgn0031821 | 0.930 | 0.925 | 0.914 | 0.989 | 1.072 |
| FBgn0003174 | 0.930 | 1.066 | 1.031 | 1.239 | 1.496 |
| FBgn0025803 | 0.930 | 1.056 | 1.054 | 1.083 | 1.107 |
| FBgn0030223 | 0.930 | 0.968 | 0.994 | 0.994 | 1.071 |
| FBgn0086253 | 0.929 | 0.768 | 0.782 | 0.795 | 0.888 |
| FBgn0015777 | 0.929 | 0.890 | 0.833 | 0.803 | 0.757 |
| FBgn0038826 | 0.929 | 1.038 | 0.935 | 0.918 | 0.811 |
| FBgn0028838 | 0.929 | 1.057 | 1.080 | 0.870 | 0.681 |
| FBgn0002905 | 0.928 | 1.063 | 0.946 | 0.840 | 0.747 |
| FBgn0030692 | 0.928 | 0.981 | 0.987 | 0.956 | 0.916 |
| FBgn0025335 | 0.928 | 1.031 | 1.137 | 1.144 | 1.270 |
| FBgn0262167 | 0.927 | 1.039 | 0.936 | 0.933 | 0.800 |
| FBgn0003067 | 0.927 | 1.029 | 1.085 | 1.191 | 1.252 |
| FBgn0037370 | 0.927 | 0.976 | 1.023 | 1.100 | 1.147 |
| FBgn0029951 | 0.927 | 1.081 | 1.039 | 1.031 | 0.859 |
| FBgn0029747 | 0.927 | 0.961 | 0.914 | 0.917 | 0.831 |
| FBgn0014127 | 0.926 | 0.873 | 0.820 | 0.820 | 0.750 |
| FBgn0029629 | 0.926 | 1.079 | 1.115 | 1.117 | 1.239 |
| FBgn0024294 | 0.925 | 0.833 | 1.139 | 1.278 | 1.526 |
| FBgn0036931 | 0.925 | 1.020 | 0.991 | 0.972 | 0.791 |
| FBgn0032538 | 0.925 | 1.175 | 1.591 | 1.574 | 2.660 |
| FBgn0028744 | 0.925 | 0.917 | 0.901 | 0.902 | 0.860 |
| FBgn0037924 | 0.925 | 1.003 | 0.920 | 0.806 | 0.734 |
| FBgn0033324 | 0.925 | 0.980 | 1.007 | 0.827 | 0.650 |
| FBgn0035217 | 0.924 | 1.067 | 1.085 | 0.911 | 0.766 |
| FBgn0032957 | 0.924 | 0.976 | 0.959 | 0.864 | 0.812 |
| FBgn0035855 | 0.924 | 1.029 | 1.028 | 0.874 | 0.773 |
| FBgn0029766 | 0.924 | 0.891 | 0.864 | 1.043 | 1.218 |
| FBgn0038714 | 0.924 | 0.909 | 0.889 | 0.992 | 1.199 |
| FBgn0086034 | 0.924 | 1.117 | 1.044 | 0.946 | 0.884 |
| FBgn0032176 | 0.924 | 1.000 | 0.868 | 0.858 | 0.710 |
| FBgn0035100 | 0.924 | 0.985 | 0.905 | 0.910 | 0.782 |
| FBgn0031850 | 0.923 | 1.039 | 1.050 | 1.094 | 1.116 |
| FBgn0036620 | 0.923 | 0.888 | 0.930 | 0.927 | 0.991 |
| FBgn0034506 | 0.923 | 0.990 | 0.936 | 0.869 | 0.825 |
| FBgn0259147 | 0.922 | 0.463 | 0.438 | 0.712 | 0.925 |
| FBgn0243486 | 0.922 | 1.082 | 1.077 | 1.048 | 0.923 |
| FBgn0052220 | 0.921 | 0.976 | 0.931 | 0.928 | 0.770 |
| FBgn0026190 | 0.921 | 1.072 | 0.945 | 0.853 | 0.759 |
| FBgn0035380 | 0.921 | 1.078 | 1.120 | 0.921 | 0.670 |
| FBgn0031345 | 0.921 | 2.446 | 2.450 | 2.095 | 0.846 |
| FBgn0033887 | 0.921 | 0.935 | 1.063 | 1.523 | 1.753 |
| FBgn0035404 | 0.920 | 1.124 | 1.173 | 1.188 | 1.230 |
| FBgn0013433 | 0.920 | 0.942 | 0.932 | 1.064 | 1.161 |
| FBgn0025186 | 0.920 | 0.935 | 0.958 | 0.970 | 1.108 |
| FBgn0051902 | 0.920 | 1.424 | 1.115 | 1.051 | 0.766 |
| FBgn0262616 | 0.919 | 1.121 | 1.071 | 1.042 | 0.728 |
| FBgn0038303 | 0.919 | 1.061 | 1.054 | 0.954 | 0.898 |
| FBgn0032636 | 0.919 | 0.989 | 0.924 | 0.922 | 0.693 |
| FBgn0036497 | 0.919 | 0.965 | 0.999 | 0.852 | 0.633 |
| FBgn0034976 | 0.919 | 0.991 | 0.999 | 1.452 | 1.726 |
| FBgn0085408 | 0.919 | 0.991 | 1.013 | 1.019 | 1.127 |
| FBgn0011655 | 0.918 | 1.039 | 1.028 | 1.072 | 1.141 |
| FBgn0029835 | 0.917 | 1.107 | 1.174 | 1.189 | 1.248 |
| FBgn0085337 | 0.916 | 1.016 | 1.045 | 0.869 | 0.710 |
| FBgn0260653 | 0.916 | 0.871 | 0.869 | 0.924 | 1.125 |
| FBgn0031053 | 0.915 | 1.022 | 1.182 | 1.285 | 1.399 |
| FBgn0038165 | 0.915 | 1.232 | 1.173 | 1.180 | 1.056 |
| FBgn0040696 | 0.915 | 1.061 | 1.021 | 0.902 | 0.846 |
| FBgn0036422 | 0.915 | 0.936 | 0.925 | 0.994 | 1.052 |
| FBgn0003435 | 0.915 | 0.963 | 0.883 | 0.843 | 0.784 |
| FBgn0030808 | 0.914 | 0.970 | 0.985 | 1.023 | 1.041 |
| FBgn0003975 | 0.914 | 1.020 | 0.974 | 1.153 | 1.452 |
| FBgn0039411 | 0.914 | 1.058 | 0.897 | 1.589 | 2.415 |
| FBgn0038102 | 0.914 | 0.971 | 0.995 | 0.875 | 0.566 |
| FBgn0040060 | 0.914 | 0.939 | 0.960 | 0.978 | 1.144 |
| FBgn0035211 | 0.914 | 0.939 | 0.808 | 0.814 | 0.643 |
| FBgn0035443 | 0.914 | 1.016 | 1.037 | 0.935 | 0.828 |
| FBgn0031275 | 0.913 | 1.066 | 1.079 | 1.019 | 0.876 |
| FBgn0034274 | 0.913 | 0.842 | 0.800 | 0.653 | 0.584 |
| FBgn0035791 | 0.913 | 1.343 | 1.273 | 1.264 | 0.948 |
| FBgn0040259 | 0.913 | 0.991 | 1.042 | 1.162 | 1.218 |
| FBgn0031944 | 0.913 | 1.001 | 0.937 | 0.868 | 0.822 |
| FBgn0014028 | 0.912 | 1.082 | 1.141 | 1.154 | 1.205 |
| FBgn0050420 | 0.912 | 0.933 | 0.924 | 0.981 | 1.028 |
| FBgn0036712 | 0.912 | 0.827 | 0.793 | 0.797 | 0.705 |
| FBgn0001332 | 0.912 | 0.960 | 0.957 | 1.014 | 1.051 |
| FBgn0004841 | 0.911 | 1.052 | 2.013 | 2.449 | 3.151 |
| FBgn0024248 | 0.911 | 0.985 | 0.893 | 0.905 | 0.749 |
| FBgn0016080 | 0.911 | 1.088 | 1.071 | 1.073 | 1.030 |
| FBgn0028561 | 0.910 | 1.163 | 1.203 | 1.031 | 0.850 |
| FBgn0259878 | 0.910 | 0.949 | 0.977 | 0.877 | 0.695 |
| FBgn0004057 | 0.910 | 1.143 | 1.149 | 1.255 | 1.310 |
| FBgn0034296 | 0.910 | 0.751 | 0.951 | 0.936 | 1.208 |
| FBgn0037291 | 0.909 | 1.082 | 1.107 | 0.910 | 0.767 |
| FBgn0039130 | 0.909 | 1.087 | 1.151 | 1.186 | 1.230 |
| FBgn0085205 | 0.908 | 0.820 | 0.836 | 0.932 | 1.433 |
| FBgn0039088 | 0.908 | 1.107 | 1.039 | 1.043 | 0.798 |
| FBgn0037470 | 0.908 | 0.984 | 0.961 | 0.955 | 0.823 |
| FBgn0035586 | 0.908 | 0.967 | 0.969 | 0.996 | 1.120 |
| FBgn0053880 | 0.908 | 0.807 | 0.775 | 0.433 | 0.266 |
| FBgn0026563 | 0.908 | 1.151 | 1.139 | 0.935 | 0.832 |
| FBgn0029512 | 0.907 | 0.946 | 0.861 | 0.863 | 0.765 |
| FBgn0037015 | 0.907 | 0.895 | 0.921 | 0.947 | 0.965 |
| FBgn0026261 | 0.907 | 0.927 | 0.915 | 0.832 | 0.794 |
| FBgn0043005 | 0.906 | 0.975 | 0.945 | 1.813 | 5.466 |
| FBgn0037581 | 0.906 | 0.947 | 0.782 | 0.610 | 0.499 |
| FBgn0051797 | 0.906 | 0.945 | 0.837 | 0.788 | 0.713 |
| FBgn0027101 | 0.906 | 1.100 | 1.091 | 1.057 | 0.855 |
| FBgn0036666 | 0.906 | 0.940 | 0.950 | 0.829 | 0.753 |
| FBgn0052319 | 0.905 | 0.933 | 0.978 | 0.798 | 0.604 |
| FBgn0250871 | 0.905 | 1.080 | 0.988 | 1.375 | 1.763 |
| FBgn0032484 | 0.905 | 1.521 | 1.095 | 1.002 | 0.654 |
| FBgn0005677 | 0.905 | 1.010 | 1.131 | 1.110 | 1.417 |
| FBgn0035281 | 0.904 | 0.646 | 0.536 | 0.898 | 1.549 |
| FBgn0038447 | 0.904 | 1.299 | 1.321 | 1.800 | 2.037 |
| FBgn0000633 | 0.904 | 0.994 | 1.006 | 0.936 | 0.881 |
| FBgn0033169 | 0.904 | 0.993 | 0.881 | 0.864 | 0.768 |
| FBgn0022160 | 0.904 | 1.035 | 1.011 | 1.115 | 1.212 |
| FBgn0037146 | 0.903 | 1.155 | 1.209 | 1.205 | 1.273 |
| FBgn0053904 | 0.903 | 0.811 | 0.765 | 0.428 | 0.278 |
| FBgn0261705 | 0.903 | 1.261 | 1.130 | 1.133 | 0.987 |
| FBgn0036017 | 0.903 | 0.986 | 1.615 | 1.580 | 4.025 |
| FBgn0029084 | 0.903 | 0.925 | 0.841 | 0.753 | 0.699 |
| FBgn0032647 | 0.902 | 0.970 | 0.903 | 0.916 | 0.748 |
| FBgn0031139 | 0.902 | 1.021 | 0.994 | 0.977 | 0.761 |
| FBgn0035798 | 0.902 | 1.047 | 1.013 | 1.124 | 1.300 |
| FBgn0038488 | 0.902 | 1.085 | 1.063 | 1.056 | 0.914 |
| FBgn0002941 | 0.902 | 0.896 | 0.678 | 2.306 | 3.418 |
| FBgn0033203 | 0.902 | 0.937 | 0.967 | 0.868 | 0.659 |
| FBgn0000482 | 0.902 | 0.922 | 0.942 | 0.944 | 1.048 |
| FBgn0034223 | 0.901 | 1.005 | 0.963 | 1.101 | 1.403 |
| FBgn0036659 | 0.901 | 0.872 | 0.807 | 0.820 | 0.669 |
| FBgn0260776 | 0.901 | 1.628 | 1.209 | 1.240 | 0.727 |
| FBgn0028893 | 0.901 | 0.858 | 0.884 | 0.786 | 0.513 |
| FBgn0050285 | 0.901 | 1.101 | 1.016 | 1.281 | 1.706 |
| FBgn0051776 | 0.901 | 1.003 | 0.887 | 0.688 | 0.598 |
| FBgn0037754 | 0.900 | 0.959 | 0.934 | 1.022 | 1.122 |
| FBgn0023536 | 0.900 | 1.005 | 1.022 | 0.904 | 0.823 |
| FBgn0031261 | 0.900 | 0.871 | 0.878 | 0.968 | 1.008 |
| FBgn0034157 | 0.900 | 1.375 | 1.446 | 1.224 | 0.863 |
| FBgn0033187 | 0.900 | 0.992 | 1.003 | 0.899 | 0.834 |

| Supplemental Table 3B. Values of R Squared and Fold Changes for Genes Found to Behave Linearly at Day 10 Post-Irradiation. (Analysis with all lowest dose discluded.) | | | | | |
| --- | --- | --- | --- | --- | --- |
|  |  |  |  |  |  |
| Flybase ID | R Squared Value Day10 | Day10 Fold Change 1000R | Day10 Fold Change 5000R | Day10 Fold Change 10000R | Day10 Fold Change 20000R |
| FBgn0052280 | 1.000 | 0.918 | 1.070 | 1.253 | 1.615 |
| FBgn0036754 | 1.000 | 1.096 | 1.227 | 1.400 | 1.728 |
| FBgn0042180 | 1.000 | 1.414 | 1.285 | 1.120 | 0.812 |
| FBgn0259144 | 1.000 | 1.104 | 1.200 | 1.339 | 1.591 |
| FBgn0022246 | 1.000 | 0.948 | 1.101 | 1.300 | 1.722 |
| FBgn0033134 | 0.999 | 0.868 | 1.056 | 1.333 | 1.866 |
| FBgn0051352 | 0.999 | 1.058 | 0.997 | 0.912 | 0.762 |
| FBgn0021847 | 0.999 | 1.086 | 1.057 | 1.015 | 0.933 |
| FBgn0002922 | 0.999 | 1.055 | 1.170 | 1.336 | 1.627 |
| FBgn0033949 | 0.999 | 1.353 | 1.548 | 1.774 | 2.204 |
| FBgn0035611 | 0.999 | 0.982 | 1.184 | 1.413 | 1.951 |
| FBgn0011509 | 0.999 | 0.946 | 1.020 | 1.101 | 1.293 |
| FBgn0030038 | 0.999 | 1.024 | 0.957 | 0.885 | 0.744 |
| FBgn0028697 | 0.999 | 1.099 | 1.212 | 1.330 | 1.614 |
| FBgn0029913 | 0.999 | 0.920 | 1.018 | 1.170 | 1.424 |
| FBgn0045038 | 0.999 | 0.850 | 0.933 | 1.064 | 1.281 |
| FBgn0027500 | 0.999 | 1.291 | 1.043 | 0.793 | 0.277 |
| FBgn0037144 | 0.999 | 0.984 | 1.084 | 1.229 | 1.534 |
| FBgn0004366 | 0.999 | 0.936 | 1.017 | 1.110 | 1.333 |
| FBgn0027491 | 0.998 | 1.005 | 1.054 | 1.112 | 1.251 |
| FBgn0002638 | 0.998 | 1.195 | 1.098 | 0.955 | 0.654 |
| FBgn0038578 | 0.998 | 0.905 | 0.927 | 0.950 | 0.997 |
| FBgn0013771 | 0.998 | 0.753 | 0.846 | 1.001 | 1.258 |
| FBgn0050005 | 0.998 | 0.991 | 1.064 | 1.183 | 1.416 |
| FBgn0004597 | 0.998 | 1.047 | 1.132 | 1.209 | 1.391 |
| FBgn0023514 | 0.998 | 0.989 | 1.071 | 1.205 | 1.423 |
| FBgn0052579 | 0.998 | 1.067 | 1.231 | 1.467 | 1.981 |
| FBgn0259748 | 0.998 | 0.870 | 1.018 | 1.168 | 1.465 |
| FBgn0037057 | 0.998 | 1.090 | 1.047 | 0.992 | 0.900 |
| FBgn0050151 | 0.998 | 0.884 | 1.029 | 1.218 | 1.654 |
| FBgn0038966 | 0.998 | 0.822 | 0.888 | 0.974 | 1.118 |
| FBgn0046763 | 0.998 | 0.875 | 1.061 | 1.225 | 1.614 |
| FBgn0031115 | 0.998 | 0.979 | 1.025 | 1.092 | 1.239 |
| FBgn0034139 | 0.998 | 0.890 | 1.173 | 1.422 | 2.064 |
| FBgn0016041 | 0.998 | 1.076 | 1.007 | 0.883 | 0.651 |
| FBgn0037563 | 0.998 | 0.850 | 0.962 | 1.084 | 1.306 |
| FBgn0039068 | 0.998 | 1.089 | 1.204 | 1.360 | 1.721 |
| FBgn0052590 | 0.997 | 0.665 | 0.754 | 0.924 | 1.205 |
| FBgn0067629 | 0.997 | 1.119 | 1.202 | 1.333 | 1.611 |
| FBgn0024732 | 0.997 | 0.802 | 0.931 | 1.057 | 1.394 |
| FBgn0038113 | 0.997 | 1.009 | 1.186 | 1.353 | 1.804 |
| FBgn0028919 | 0.997 | 0.987 | 1.104 | 1.333 | 1.714 |
| FBgn0032470 | 0.997 | 1.184 | 1.421 | 1.889 | 2.696 |
| FBgn0051202 | 0.997 | 0.643 | 0.739 | 0.922 | 1.259 |
| FBgn0261446 | 0.997 | 0.921 | 1.037 | 1.228 | 1.515 |
| FBgn0033032 | 0.997 | 0.909 | 1.018 | 1.108 | 1.358 |
| FBgn0002924 | 0.997 | 1.021 | 0.882 | 0.682 | 0.376 |
| FBgn0033928 | 0.996 | 1.364 | 1.837 | 2.217 | 3.148 |
| FBgn0051674 | 0.996 | 0.882 | 0.942 | 1.066 | 1.278 |
| FBgn0037561 | 0.996 | 1.001 | 1.049 | 1.151 | 1.309 |
| FBgn0035673 | 0.996 | 0.876 | 1.001 | 1.263 | 1.677 |
| FBgn0032719 | 0.996 | 0.928 | 1.064 | 1.195 | 1.445 |
| FBgn0034512 | 0.996 | 0.957 | 1.058 | 1.244 | 1.512 |
| FBgn0036290 | 0.996 | 0.797 | 1.069 | 1.496 | 2.118 |
| FBgn0004237 | 0.996 | 1.011 | 0.950 | 0.836 | 0.670 |
| FBgn0262147 | 0.996 | 0.794 | 0.897 | 0.984 | 1.173 |
| FBgn0036889 | 0.996 | 0.960 | 1.035 | 1.123 | 1.355 |
| FBgn0040348 | 0.996 | 1.043 | 1.100 | 1.142 | 1.258 |
| FBgn0052675 | 0.995 | 1.025 | 0.981 | 0.934 | 0.854 |
| FBgn0038549 | 0.995 | 0.768 | 1.019 | 1.204 | 1.745 |
| FBgn0035876 | 0.995 | 0.871 | 0.999 | 1.104 | 1.335 |
| FBgn0020415 | 0.995 | 0.875 | 0.919 | 1.019 | 1.180 |
| FBgn0036780 | 0.995 | 0.858 | 1.019 | 1.305 | 1.924 |
| FBgn0033693 | 0.995 | 1.228 | 1.141 | 0.996 | 0.794 |
| FBgn0053964 | 0.995 | 1.228 | 1.141 | 0.996 | 0.794 |
| FBgn0038290 | 0.995 | 1.080 | 1.251 | 1.444 | 1.977 |
| FBgn0039215 | 0.994 | 1.100 | 1.070 | 1.000 | 0.886 |
| FBgn0034748 | 0.994 | 0.908 | 1.104 | 1.241 | 1.612 |
| FBgn0029755 | 0.994 | 0.976 | 1.136 | 1.450 | 1.880 |
| FBgn0002466 | 0.994 | 1.118 | 0.909 | 0.723 | 0.365 |
| FBgn0031713 | 0.994 | 1.034 | 1.183 | 1.417 | 1.739 |
| FBgn0053099 | 0.994 | 0.806 | 0.974 | 1.087 | 1.421 |
| FBgn0034517 | 0.994 | 1.000 | 1.236 | 1.415 | 1.826 |
| FBgn0043536 | 0.994 | 1.000 | 1.236 | 1.415 | 1.826 |
| FBgn0058100 | 0.993 | 0.699 | 0.967 | 1.151 | 1.745 |
| FBgn0037676 | 0.993 | 0.852 | 0.960 | 1.181 | 1.635 |
| FBgn0037647 | 0.993 | 1.005 | 1.077 | 1.256 | 1.521 |
| FBgn0028562 | 0.993 | 0.855 | 1.057 | 1.197 | 1.560 |
| FBgn0036822 | 0.993 | 0.852 | 0.975 | 1.285 | 1.815 |
| FBgn0023495 | 0.993 | 1.330 | 1.167 | 1.060 | 0.727 |
| FBgn0261975 | 0.993 | 0.814 | 0.949 | 1.081 | 1.311 |
| FBgn0032805 | 0.993 | 1.071 | 1.338 | 1.514 | 2.088 |
| FBgn0039329 | 0.993 | 1.109 | 1.149 | 1.239 | 1.414 |
| FBgn0010348 | 0.993 | 0.872 | 0.941 | 1.061 | 1.219 |
| FBgn0031317 | 0.993 | 0.900 | 1.148 | 1.436 | 1.877 |
| FBgn0039709 | 0.993 | 1.041 | 1.093 | 1.223 | 1.407 |
| FBgn0003996 | 0.993 | 0.838 | 0.961 | 1.113 | 1.536 |
| FBgn0086909 | 0.992 | 0.918 | 0.959 | 1.002 | 1.132 |
| FBgn0037071 | 0.992 | 1.059 | 1.190 | 1.524 | 2.130 |
| FBgn0010333 | 0.992 | 0.986 | 1.045 | 1.082 | 1.185 |
| FBgn0051102 | 0.992 | 0.821 | 0.865 | 0.987 | 1.188 |
| FBgn0046878 | 0.991 | 1.125 | 1.367 | 1.959 | 2.738 |
| FBgn0085353 | 0.991 | 0.871 | 0.957 | 1.029 | 1.167 |
| FBgn0029664 | 0.991 | 0.932 | 0.979 | 1.056 | 1.247 |
| FBgn0034247 | 0.991 | 0.907 | 0.943 | 1.039 | 1.211 |
| FBgn0051032 | 0.991 | 1.047 | 1.060 | 1.098 | 1.153 |
| FBgn0037652 | 0.991 | 1.087 | 1.135 | 1.229 | 1.349 |
| FBgn0031779 | 0.991 | 1.108 | 1.058 | 0.917 | 0.721 |
| FBgn0026374 | 0.991 | 0.968 | 1.124 | 1.323 | 1.603 |
| FBgn0005596 | 0.991 | 1.109 | 1.011 | 0.801 | 0.534 |
| FBgn0033458 | 0.991 | 1.192 | 1.349 | 1.812 | 2.532 |
| FBgn0040850 | 0.991 | 1.170 | 1.425 | 1.575 | 2.086 |
| FBgn0036232 | 0.991 | 0.771 | 0.863 | 0.975 | 1.135 |
| FBgn0023000 | 0.991 | 0.910 | 1.010 | 1.237 | 1.522 |
| FBgn0037818 | 0.991 | 0.959 | 0.991 | 1.087 | 1.240 |
| FBgn0013750 | 0.991 | 0.787 | 0.828 | 0.940 | 1.140 |
| FBgn0052191 | 0.991 | 1.107 | 1.168 | 1.330 | 1.544 |
| FBgn0038893 | 0.990 | 0.974 | 1.447 | 1.769 | 2.943 |
| FBgn0024912 | 0.990 | 1.051 | 1.549 | 1.898 | 2.703 |
| FBgn0031726 | 0.990 | 0.984 | 1.164 | 1.639 | 2.257 |
| FBgn0038928 | 0.990 | 0.971 | 1.105 | 1.188 | 1.413 |
| FBgn0008651 | 0.990 | 0.915 | 1.093 | 1.344 | 1.675 |
| FBgn0035949 | 0.990 | 0.854 | 0.922 | 1.109 | 1.351 |
| FBgn0085285 | 0.990 | 0.853 | 1.158 | 1.441 | 1.922 |
| FBgn0030742 | 0.990 | 0.954 | 0.976 | 1.028 | 1.093 |
| FBgn0039161 | 0.990 | 0.741 | 0.837 | 1.139 | 1.598 |
| FBgn0031054 | 0.990 | 1.058 | 1.114 | 1.185 | 1.391 |
| FBgn0004921 | 0.990 | 0.893 | 0.996 | 1.136 | 1.321 |
| FBgn0003174 | 0.990 | 0.891 | 0.993 | 1.121 | 1.298 |
| FBgn0034888 | 0.990 | 0.830 | 0.886 | 1.059 | 1.349 |
| FBgn0030041 | 0.990 | 1.011 | 1.091 | 1.321 | 1.620 |
| FBgn0032514 | 0.989 | 0.976 | 1.101 | 1.253 | 1.464 |
| FBgn0031145 | 0.989 | 0.879 | 1.014 | 1.178 | 1.667 |
| FBgn0024360 | 0.989 | 1.127 | 1.062 | 0.904 | 0.709 |
| FBgn0032405 | 0.989 | 0.854 | 1.153 | 1.316 | 1.899 |
| FBgn0033315 | 0.989 | 0.912 | 1.034 | 1.159 | 1.565 |
| FBgn0038632 | 0.989 | 0.909 | 1.101 | 1.455 | 2.318 |
| FBgn0046222 | 0.989 | 1.014 | 0.955 | 0.896 | 0.803 |
| FBgn0037036 | 0.989 | 0.823 | 0.868 | 1.018 | 1.253 |
| FBgn0037712 | 0.989 | 0.906 | 1.139 | 1.280 | 1.665 |
| FBgn0051019 | 0.989 | 0.914 | 1.105 | 1.217 | 1.534 |
| FBgn0040827 | 0.988 | 0.845 | 0.911 | 0.994 | 1.242 |
| FBgn0035577 | 0.988 | 1.178 | 1.358 | 1.910 | 2.622 |
| FBgn0030514 | 0.988 | 1.055 | 1.047 | 1.028 | 0.985 |
| FBgn0052056 | 0.988 | 0.975 | 1.077 | 1.184 | 1.345 |
| FBgn0032614 | 0.988 | 0.823 | 0.900 | 1.044 | 1.214 |
| FBgn0039179 | 0.988 | 1.011 | 1.062 | 1.174 | 1.304 |
| FBgn0034726 | 0.988 | 0.890 | 0.988 | 1.070 | 1.367 |
| FBgn0025879 | 0.988 | 0.904 | 0.961 | 1.129 | 1.339 |
| FBgn0029672 | 0.988 | 0.920 | 1.171 | 1.332 | 1.974 |
| FBgn0011335 | 0.988 | 0.835 | 0.877 | 0.968 | 1.073 |
| FBgn0052225 | 0.988 | 1.034 | 1.120 | 1.202 | 1.333 |
| FBgn0037003 | 0.988 | 0.994 | 1.067 | 1.168 | 1.462 |
| FBgn0085232 | 0.988 | 0.876 | 0.958 | 1.032 | 1.295 |
| FBgn0034184 | 0.988 | 1.031 | 1.072 | 1.137 | 1.313 |
| FBgn0031602 | 0.987 | 1.053 | 1.118 | 1.345 | 1.719 |
| FBgn0050022 | 0.987 | 0.912 | 1.191 | 1.368 | 2.087 |
| FBgn0038432 | 0.987 | 0.975 | 1.107 | 1.329 | 1.920 |
| FBgn0032021 | 0.987 | 0.933 | 1.018 | 1.208 | 1.648 |
| FBgn0031376 | 0.987 | 1.051 | 1.025 | 0.939 | 0.828 |
| FBgn0067318 | 0.987 | 0.919 | 0.983 | 1.212 | 1.518 |
| FBgn0033926 | 0.987 | 1.533 | 1.747 | 2.548 | 3.675 |
| FBgn0033453 | 0.987 | 0.835 | 1.168 | 1.339 | 1.900 |
| FBgn0031996 | 0.986 | 1.053 | 1.196 | 1.270 | 1.506 |
| FBgn0033379 | 0.986 | 0.957 | 0.981 | 1.060 | 1.157 |
| FBgn0029950 | 0.986 | 0.777 | 1.006 | 1.179 | 1.513 |
| FBgn0032105 | 0.986 | 1.079 | 0.993 | 0.659 | 0.134 |
| FBgn0050052 | 0.986 | 1.316 | 1.379 | 1.449 | 1.546 |
| FBgn0025366 | 0.986 | 0.889 | 1.024 | 1.139 | 1.336 |
| FBgn0031904 | 0.986 | 1.014 | 1.062 | 1.213 | 1.392 |
| FBgn0262902 | 0.985 | 0.771 | 0.998 | 1.104 | 1.516 |
| FBgn0015714 | 0.985 | 0.908 | 0.972 | 1.156 | 1.542 |
| FBgn0039324 | 0.985 | 0.777 | 0.867 | 1.041 | 1.236 |
| FBgn0039780 | 0.985 | 0.965 | 1.086 | 1.177 | 1.542 |
| FBgn0032230 | 0.985 | 1.122 | 1.343 | 1.544 | 2.284 |
| FBgn0039911 | 0.985 | 0.853 | 0.923 | 1.045 | 1.378 |
| FBgn0031260 | 0.985 | 0.988 | 1.045 | 1.075 | 1.165 |
| FBgn0037519 | 0.985 | 0.850 | 1.058 | 1.157 | 1.600 |
| FBgn0037960 | 0.985 | 0.777 | 0.840 | 1.012 | 1.389 |
| FBgn0039630 | 0.985 | 1.387 | 1.580 | 2.020 | 2.503 |
| FBgn0013764 | 0.985 | 1.031 | 1.001 | 0.876 | 0.680 |
| FBgn0030164 | 0.985 | 0.993 | 1.037 | 1.204 | 1.498 |
| FBgn0028940 | 0.985 | 0.905 | 1.064 | 1.245 | 1.487 |
| FBgn0053120 | 0.985 | 0.739 | 0.769 | 0.881 | 1.021 |
| FBgn0044047 | 0.985 | 1.000 | 1.092 | 1.258 | 1.708 |
| FBgn0016650 | 0.984 | 0.863 | 0.924 | 1.101 | 1.301 |
| FBgn0032536 | 0.984 | 0.940 | 1.050 | 1.260 | 1.813 |
| FBgn0037731 | 0.984 | 0.973 | 1.059 | 1.436 | 1.996 |
| FBgn0036665 | 0.984 | 0.794 | 0.894 | 1.328 | 1.998 |
| FBgn0002641 | 0.984 | 1.068 | 1.144 | 1.398 | 1.691 |
| FBgn0035975 | 0.984 | 0.882 | 0.973 | 1.074 | 1.420 |
| FBgn0031673 | 0.984 | 0.997 | 0.979 | 0.900 | 0.785 |
| FBgn0036911 | 0.984 | 0.927 | 0.968 | 1.149 | 1.412 |
| FBgn0032023 | 0.984 | 0.834 | 1.028 | 1.189 | 1.823 |
| FBgn0031270 | 0.983 | 0.879 | 0.980 | 1.406 | 1.949 |
| FBgn0034756 | 0.983 | 1.074 | 1.200 | 1.765 | 2.679 |
| FBgn0085419 | 0.983 | 0.895 | 0.997 | 1.090 | 1.445 |
| FBgn0011676 | 0.983 | 0.976 | 1.027 | 1.190 | 1.531 |
| FBgn0035028 | 0.983 | 0.922 | 0.954 | 1.042 | 1.240 |
| FBgn0035165 | 0.983 | 0.944 | 1.058 | 1.119 | 1.286 |
| FBgn0250847 | 0.983 | 0.675 | 0.700 | 0.813 | 0.959 |
| FBgn0051659 | 0.983 | 0.827 | 0.890 | 1.061 | 1.244 |
| FBgn0034656 | 0.983 | 0.855 | 0.889 | 1.025 | 1.186 |
| FBgn0010043 | 0.982 | 0.863 | 0.938 | 1.016 | 1.301 |
| FBgn0029763 | 0.982 | 1.124 | 1.055 | 1.022 | 0.863 |
| FBgn0035517 | 0.982 | 0.925 | 0.994 | 1.042 | 1.137 |
| FBgn0034187 | 0.982 | 4.389 | 5.333 | 9.749 | 17.037 |
| FBgn0033519 | 0.982 | 1.183 | 1.165 | 1.080 | 0.947 |
| FBgn0041205 | 0.982 | 0.998 | 1.073 | 1.129 | 1.231 |
| FBgn0038035 | 0.982 | 1.266 | 1.400 | 1.801 | 2.690 |
| FBgn0033215 | 0.982 | 0.928 | 1.107 | 1.306 | 2.014 |
| FBgn0035035 | 0.982 | 1.124 | 1.068 | 1.004 | 0.778 |
| FBgn0030799 | 0.982 | 0.864 | 1.023 | 1.168 | 1.386 |
| FBgn0033368 | 0.981 | 0.875 | 0.912 | 1.072 | 1.366 |
| FBgn0039861 | 0.981 | 1.101 | 1.036 | 1.011 | 0.887 |
| FBgn0033927 | 0.981 | 1.570 | 1.781 | 2.694 | 3.778 |
| FBgn0032235 | 0.981 | 0.829 | 1.047 | 1.133 | 1.539 |
| FBgn0032393 | 0.981 | 1.130 | 1.699 | 2.025 | 2.818 |
| FBgn0085377 | 0.981 | 0.949 | 1.035 | 1.367 | 1.742 |
| FBgn0052072 | 0.981 | 0.997 | 1.213 | 1.302 | 1.760 |
| FBgn0033038 | 0.981 | 0.929 | 0.951 | 1.059 | 1.198 |
| FBgn0020278 | 0.981 | 1.070 | 1.088 | 1.151 | 1.220 |
| FBgn0030048 | 0.981 | 0.637 | 0.954 | 1.104 | 1.871 |
| FBgn0000173 | 0.981 | 0.994 | 1.040 | 1.207 | 1.390 |
| FBgn0030481 | 0.981 | 1.082 | 1.025 | 0.910 | 0.790 |
| FBgn0000221 | 0.981 | 1.241 | 1.190 | 1.153 | 0.990 |
| FBgn0031893 | 0.981 | 0.892 | 1.017 | 1.071 | 1.266 |
| FBgn0043069 | 0.980 | 0.569 | 0.922 | 1.256 | 2.562 |
| FBgn0040350 | 0.980 | 0.905 | 0.944 | 1.142 | 1.474 |
| FBgn0052368 | 0.980 | 1.700 | 4.955 | 6.366 | 13.820 |
| FBgn0036836 | 0.980 | 0.954 | 0.986 | 1.153 | 1.377 |
| FBgn0032180 | 0.980 | 0.949 | 1.029 | 1.153 | 1.538 |
| FBgn0031434 | 0.980 | 0.959 | 0.892 | 0.767 | 0.637 |
| FBgn0035398 | 0.980 | 1.132 | 1.359 | 1.994 | 3.501 |
| FBgn0030947 | 0.980 | 0.963 | 1.265 | 1.525 | 1.929 |
| FBgn0034605 | 0.980 | 1.046 | 1.370 | 1.555 | 1.999 |
| FBgn0039670 | 0.980 | 0.942 | 1.234 | 1.551 | 1.961 |
| FBgn0039481 | 0.980 | 0.848 | 0.932 | 0.979 | 1.215 |
| FBgn0039965 | 0.980 | 0.987 | 1.045 | 1.118 | 1.205 |
| FBgn0027780 | 0.980 | 0.890 | 0.946 | 1.266 | 1.694 |
| FBgn0031213 | 0.980 | 1.062 | 1.175 | 1.401 | 2.036 |
| FBgn0050281 | 0.980 | 0.877 | 1.037 | 1.111 | 1.507 |
| FBgn0051103 | 0.979 | 1.065 | 1.303 | 1.404 | 1.764 |
| FBgn0039596 | 0.979 | 1.237 | 1.532 | 2.621 | 4.923 |
| FBgn0259714 | 0.979 | 0.868 | 0.932 | 1.164 | 1.662 |
| FBgn0033240 | 0.979 | 0.748 | 0.934 | 1.014 | 1.291 |
| FBgn0033932 | 0.979 | 0.883 | 1.026 | 1.084 | 1.405 |
| FBgn0033812 | 0.979 | 0.970 | 0.989 | 1.038 | 1.086 |
| FBgn0032819 | 0.979 | 0.910 | 0.983 | 1.290 | 1.904 |
| FBgn0010280 | 0.979 | 1.090 | 0.981 | 0.909 | 0.766 |
| FBgn0037730 | 0.979 | 0.879 | 0.945 | 1.127 | 1.576 |
| FBgn0035916 | 0.979 | 0.975 | 1.023 | 1.196 | 1.570 |
| FBgn0000241 | 0.979 | 0.950 | 1.105 | 1.199 | 1.661 |
| FBgn0260946 | 0.978 | 1.012 | 1.051 | 1.182 | 1.314 |
| FBgn0087039 | 0.978 | 0.925 | 1.002 | 1.149 | 1.582 |
| FBgn0033222 | 0.978 | 1.476 | 1.907 | 2.221 | 2.774 |
| FBgn0032074 | 0.978 | 0.860 | 1.069 | 1.303 | 1.590 |
| FBgn0032538 | 0.978 | 1.209 | 1.610 | 2.392 | 4.681 |
| FBgn0038964 | 0.977 | 1.042 | 1.069 | 1.146 | 1.338 |
| FBgn0052533 | 0.977 | 1.167 | 1.142 | 1.113 | 1.003 |
| FBgn0024733 | 0.977 | 1.017 | 1.065 | 1.341 | 1.665 |
| FBgn0034788 | 0.977 | 0.950 | 1.019 | 1.177 | 1.613 |
| FBgn0050055 | 0.977 | 0.899 | 1.143 | 1.361 | 1.674 |
| FBgn0020270 | 0.976 | 1.036 | 1.096 | 1.529 | 2.202 |
| FBgn0003248 | 0.976 | 0.851 | 0.832 | 0.791 | 0.751 |
| FBgn0035293 | 0.976 | 1.231 | 1.251 | 1.265 | 1.332 |
| FBgn0035661 | 0.976 | 0.868 | 0.971 | 1.260 | 1.536 |
| FBgn0263241 | 0.976 | 1.028 | 1.118 | 1.172 | 1.450 |
| FBgn0037978 | 0.976 | 1.100 | 1.041 | 0.909 | 0.784 |
| FBgn0029147 | 0.976 | 1.255 | 1.340 | 1.744 | 2.173 |
| FBgn0028399 | 0.976 | 0.934 | 1.071 | 1.157 | 1.331 |
| FBgn0037491 | 0.976 | 0.963 | 1.064 | 1.117 | 1.408 |
| FBgn0051105 | 0.976 | 0.801 | 1.012 | 1.105 | 1.654 |
| FBgn0036547 | 0.976 | 0.993 | 1.052 | 1.098 | 1.173 |
| FBgn0020277 | 0.976 | 0.832 | 0.871 | 0.988 | 1.279 |
| FBgn0262583 | 0.976 | 0.710 | 0.793 | 0.824 | 1.015 |
| FBgn0000039 | 0.975 | 0.880 | 0.918 | 1.151 | 1.417 |
| FBgn0037684 | 0.975 | 0.798 | 0.931 | 1.163 | 1.390 |
| FBgn0260386 | 0.975 | 0.866 | 0.892 | 1.082 | 1.399 |
| FBgn0033850 | 0.975 | 1.066 | 1.105 | 1.367 | 1.674 |
| FBgn0030854 | 0.975 | 0.967 | 0.997 | 1.174 | 1.515 |
| FBgn0033526 | 0.975 | 1.067 | 0.938 | 0.714 | 0.496 |
| FBgn0037250 | 0.975 | 1.244 | 1.145 | 1.104 | 0.967 |
| FBgn0030241 | 0.975 | 1.277 | 1.188 | 0.716 | 0.217 |
| FBgn0026874 | 0.975 | 1.043 | 1.078 | 1.218 | 1.356 |
| FBgn0036145 | 0.974 | 0.748 | 0.879 | 1.177 | 1.453 |
| FBgn0032477 | 0.974 | 0.946 | 1.048 | 1.192 | 1.342 |
| FBgn0033203 | 0.974 | 0.946 | 0.895 | 0.740 | 0.596 |
| FBgn0038353 | 0.974 | 0.909 | 1.083 | 1.175 | 1.396 |
| FBgn0037004 | 0.974 | 0.972 | 1.004 | 1.191 | 1.555 |
| FBgn0030026 | 0.974 | 0.938 | 1.052 | 1.397 | 1.718 |
| FBgn0053635 | 0.974 | 1.066 | 1.151 | 1.603 | 2.526 |
| FBgn0063494 | 0.974 | 0.901 | 1.091 | 1.162 | 1.434 |
| FBgn0051274 | 0.974 | 0.581 | 0.966 | 1.195 | 2.428 |
| FBgn0260874 | 0.974 | 0.999 | 1.069 | 1.390 | 2.092 |
| FBgn0262872 | 0.974 | 1.162 | 1.057 | 0.964 | 0.835 |
| FBgn0035805 | 0.973 | 0.882 | 0.920 | 1.173 | 1.452 |
| FBgn0036428 | 0.973 | 1.013 | 1.060 | 1.201 | 1.329 |
| FBgn0030734 | 0.973 | 0.948 | 1.053 | 1.085 | 1.304 |
| FBgn0035644 | 0.973 | 1.073 | 0.794 | 0.557 | 0.217 |
| FBgn0086445 | 0.973 | 1.058 | 1.187 | 1.374 | 1.562 |
| FBgn0050394 | 0.973 | 0.954 | 1.023 | 1.098 | 1.186 |
| FBgn0037566 | 0.973 | 0.954 | 1.005 | 1.038 | 1.212 |
| FBgn0003721 | 0.973 | 1.055 | 1.100 | 1.241 | 1.370 |
| FBgn0031703 | 0.972 | 0.763 | 0.860 | 1.007 | 1.152 |
| FBgn0000451 | 0.972 | 1.133 | 1.318 | 1.372 | 1.758 |
| FBgn0034967 | 0.972 | 0.897 | 0.927 | 1.192 | 1.513 |
| FBgn0038595 | 0.972 | 0.660 | 0.871 | 0.951 | 1.239 |
| FBgn0034398 | 0.972 | 0.837 | 1.078 | 1.174 | 1.495 |
| FBgn0030189 | 0.972 | 0.992 | 1.010 | 1.195 | 1.489 |
| FBgn0037076 | 0.971 | 1.039 | 1.271 | 1.566 | 1.877 |
| FBgn0039265 | 0.971 | 0.938 | 1.059 | 1.154 | 1.298 |
| FBgn0000370 | 0.971 | 0.986 | 1.005 | 1.195 | 1.514 |
| FBgn0035523 | 0.971 | 1.022 | 1.057 | 1.270 | 1.486 |
| FBgn0037882 | 0.971 | 0.853 | 1.114 | 1.427 | 1.767 |
| FBgn0051961 | 0.971 | 0.855 | 0.870 | 0.984 | 1.107 |
| FBgn0025583 | 0.971 | 0.860 | 1.225 | 1.327 | 1.917 |
| FBgn0038926 | 0.971 | 0.908 | 1.014 | 1.190 | 1.799 |
| FBgn0036489 | 0.971 | 0.902 | 1.050 | 1.090 | 1.340 |
| FBgn0032409 | 0.971 | 0.844 | 0.950 | 1.147 | 1.326 |
| FBgn0052750 | 0.971 | 0.754 | 0.786 | 0.949 | 1.106 |
| FBgn0039015 | 0.970 | 1.032 | 1.162 | 1.324 | 1.965 |
| FBgn0030093 | 0.970 | 0.961 | 0.801 | 0.685 | 0.497 |
| FBgn0022786 | 0.970 | 1.045 | 0.986 | 0.958 | 0.885 |
| FBgn0001223 | 0.970 | 0.921 | 1.047 | 1.310 | 1.544 |
| FBgn0001228 | 0.970 | 0.920 | 1.046 | 1.309 | 1.543 |
| FBgn0032283 | 0.970 | 1.537 | 1.441 | 1.251 | 1.082 |
| FBgn0038761 | 0.970 | 1.197 | 1.361 | 1.459 | 1.653 |
| FBgn0025807 | 0.970 | 1.012 | 1.145 | 1.209 | 1.612 |
| FBgn0011774 | 0.970 | 1.035 | 1.228 | 1.975 | 2.650 |
| FBgn0259952 | 0.970 | 1.683 | 1.935 | 2.610 | 3.198 |
| FBgn0034611 | 0.970 | 0.965 | 1.004 | 1.150 | 1.280 |
| FBgn0003969 | 0.970 | 1.106 | 1.133 | 1.290 | 1.631 |
| FBgn0041171 | 0.970 | 1.130 | 0.943 | 0.876 | 0.625 |
| FBgn0033769 | 0.970 | 0.892 | 0.981 | 1.101 | 1.222 |
| FBgn0030556 | 0.970 | 1.044 | 0.994 | 0.872 | 0.767 |
| FBgn0031563 | 0.970 | 0.764 | 0.878 | 0.932 | 1.277 |
| FBgn0262057 | 0.969 | 0.997 | 1.008 | 1.117 | 1.245 |
| FBgn0032644 | 0.969 | 0.812 | 1.013 | 1.092 | 1.351 |
| FBgn0051710 | 0.969 | 0.835 | 1.148 | 1.227 | 1.856 |
| FBgn0035157 | 0.969 | 1.207 | 1.276 | 1.572 | 2.291 |
| FBgn0015038 | 0.969 | 0.891 | 1.009 | 1.261 | 2.064 |
| FBgn0261393 | 0.969 | 0.956 | 0.973 | 1.082 | 1.191 |
| FBgn0029836 | 0.969 | 1.330 | 1.460 | 2.054 | 3.484 |
| FBgn0083976 | 0.969 | 0.890 | 0.908 | 0.995 | 1.075 |
| FBgn0083992 | 0.969 | 0.890 | 0.908 | 0.995 | 1.075 |
| FBgn0032085 | 0.968 | 1.297 | 1.463 | 1.544 | 2.066 |
| FBgn0037188 | 0.968 | 1.094 | 1.107 | 1.297 | 1.539 |
| FBgn0030704 | 0.968 | 0.981 | 1.173 | 1.291 | 1.512 |
| FBgn0010406 | 0.968 | 0.972 | 1.016 | 1.076 | 1.134 |
| FBgn0261989 | 0.968 | 1.148 | 2.397 | 3.470 | 8.701 |
| FBgn0039638 | 0.968 | 0.975 | 0.960 | 0.797 | 0.615 |
| FBgn0054057 | 0.968 | 0.739 | 0.908 | 0.951 | 1.319 |
| FBgn0052226 | 0.968 | 1.098 | 1.074 | 1.006 | 0.949 |
| FBgn0085769 | 0.968 | 0.847 | 1.101 | 1.285 | 1.573 |
| FBgn0039654 | 0.968 | 1.014 | 0.922 | 0.843 | 0.738 |
| FBgn0039924 | 0.968 | 0.957 | 1.146 | 1.207 | 1.694 |
| FBgn0038752 | 0.968 | 1.011 | 1.021 | 1.133 | 1.255 |
| FBgn0085455 | 0.968 | 0.894 | 1.178 | 1.546 | 1.913 |
| FBgn0035587 | 0.967 | 0.873 | 0.971 | 1.091 | 1.215 |
| FBgn0036942 | 0.967 | 0.887 | 0.968 | 1.226 | 1.444 |
| FBgn0051106 | 0.967 | 0.923 | 1.011 | 1.293 | 1.531 |
| FBgn0034152 | 0.967 | 1.384 | 2.129 | 4.491 | 6.484 |
| FBgn0035166 | 0.967 | 1.024 | 1.081 | 1.357 | 1.602 |
| FBgn0053178 | 0.967 | 0.863 | 1.005 | 1.038 | 1.265 |
| FBgn0035300 | 0.967 | 0.839 | 0.967 | 1.044 | 1.500 |
| FBgn0053173 | 0.967 | 0.985 | 0.990 | 1.083 | 1.199 |
| FBgn0039342 | 0.966 | 1.054 | 1.147 | 1.616 | 2.035 |
| FBgn0020312 | 0.966 | 1.006 | 1.124 | 1.237 | 1.373 |
| FBgn0029861 | 0.966 | 1.108 | 1.032 | 0.820 | 0.646 |
| FBgn0052576 | 0.966 | 1.127 | 1.445 | 1.512 | 2.072 |
| FBgn0032391 | 0.966 | 1.008 | 1.357 | 1.430 | 2.070 |
| FBgn0003189 | 0.966 | 1.070 | 0.929 | 0.891 | 0.692 |
| FBgn0033155 | 0.966 | 1.133 | 1.021 | 0.990 | 0.717 |
| FBgn0038057 | 0.966 | 0.923 | 0.941 | 1.295 | 1.728 |
| FBgn0037466 | 0.966 | 0.983 | 1.127 | 1.160 | 1.389 |
| FBgn0031716 | 0.965 | 0.880 | 0.899 | 1.092 | 1.477 |
| FBgn0051864 | 0.965 | 1.072 | 1.596 | 1.711 | 2.806 |
| FBgn0038069 | 0.965 | 0.737 | 0.822 | 0.913 | 1.327 |
| FBgn0039451 | 0.965 | 1.046 | 0.912 | 0.807 | 0.659 |
| FBgn0051901 | 0.965 | 1.028 | 1.605 | 1.981 | 4.167 |
| FBgn0023541 | 0.965 | 0.827 | 0.855 | 1.121 | 1.386 |
| FBgn0039332 | 0.965 | 1.005 | 1.021 | 1.247 | 1.675 |
| FBgn0085428 | 0.965 | 0.879 | 0.984 | 1.348 | 2.377 |
| FBgn0030008 | 0.964 | 1.236 | 1.206 | 0.992 | 0.793 |
| FBgn0033867 | 0.964 | 1.256 | 1.429 | 1.743 | 2.884 |
| FBgn0032225 | 0.964 | 0.964 | 0.973 | 1.253 | 1.622 |
| FBgn0035402 | 0.964 | 1.045 | 1.016 | 0.978 | 0.819 |
| FBgn0022029 | 0.964 | 0.898 | 1.072 | 1.126 | 1.353 |
| FBgn0039336 | 0.964 | 1.003 | 1.152 | 1.231 | 1.397 |
| FBgn0034735 | 0.964 | 0.901 | 0.914 | 0.964 | 1.104 |
| FBgn0039597 | 0.964 | 0.789 | 0.944 | 0.988 | 1.195 |
| FBgn0010173 | 0.964 | 1.079 | 1.116 | 1.383 | 1.629 |
| FBgn0032896 | 0.964 | 0.816 | 0.941 | 0.966 | 1.224 |
| FBgn0037848 | 0.964 | 0.981 | 1.096 | 1.120 | 1.302 |
| FBgn0039599 | 0.964 | 0.809 | 0.940 | 1.055 | 1.199 |
| FBgn0038639 | 0.963 | 0.937 | 0.971 | 1.033 | 1.262 |
| FBgn0037779 | 0.963 | 0.831 | 0.949 | 1.099 | 1.244 |
| FBgn0262366 | 0.963 | 1.050 | 2.472 | 2.858 | 4.778 |
| FBgn0010350 | 0.963 | 0.984 | 0.895 | 0.798 | 0.695 |
| FBgn0036646 | 0.963 | 1.165 | 1.050 | 0.849 | 0.679 |
| FBgn0032233 | 0.963 | 0.903 | 0.929 | 1.046 | 1.359 |
| FBgn0043796 | 0.963 | 1.189 | 1.132 | 0.992 | 0.881 |
| FBgn0262720 | 0.963 | 0.594 | 1.220 | 1.335 | 2.536 |
| FBgn0011591 | 0.963 | 0.979 | 1.028 | 1.205 | 1.348 |
| FBgn0040837 | 0.963 | 1.515 | 1.811 | 1.974 | 2.300 |
| FBgn0015351 | 0.963 | 1.072 | 1.131 | 1.446 | 2.242 |
| FBgn0038455 | 0.963 | 1.072 | 1.131 | 1.446 | 2.242 |
| FBgn0038268 | 0.963 | 1.050 | 1.071 | 1.394 | 1.732 |
| FBgn0000182 | 0.963 | 1.133 | 0.862 | 0.404 | 0.017 |
| FBgn0038092 | 0.963 | 0.740 | 0.838 | 1.017 | 1.681 |
| FBgn0037617 | 0.962 | 1.017 | 1.029 | 1.318 | 1.645 |
| FBgn0030985 | 0.962 | 1.058 | 1.186 | 1.334 | 1.483 |
| FBgn0031389 | 0.962 | 0.849 | 0.892 | 1.436 | 1.982 |
| FBgn0034181 | 0.962 | 1.004 | 0.936 | 0.916 | 0.828 |
| FBgn0036486 | 0.962 | 1.174 | 0.921 | 0.682 | 0.404 |
| FBgn0033753 | 0.962 | 1.147 | 1.313 | 1.460 | 2.223 |
| FBgn0039681 | 0.962 | 0.773 | 0.816 | 0.887 | 1.163 |
| FBgn0039386 | 0.962 | 1.100 | 1.333 | 1.451 | 2.272 |
| FBgn0051956 | 0.962 | 0.878 | 0.928 | 1.105 | 1.246 |
| FBgn0030234 | 0.961 | 1.070 | 1.079 | 1.227 | 1.527 |
| FBgn0034480 | 0.961 | 1.195 | 1.283 | 1.589 | 2.498 |
| FBgn0003071 | 0.961 | 1.111 | 0.953 | 0.816 | 0.647 |
| FBgn0053105 | 0.961 | 1.073 | 1.068 | 0.942 | 0.804 |
| FBgn0032124 | 0.961 | 0.940 | 1.172 | 1.506 | 1.800 |
| FBgn0260463 | 0.961 | 1.073 | 1.068 | 0.942 | 0.807 |
| FBgn0030833 | 0.960 | 1.078 | 1.023 | 0.955 | 0.649 |
| FBgn0052544 | 0.960 | 1.020 | 1.025 | 1.078 | 1.193 |
| FBgn0034931 | 0.960 | 0.930 | 1.027 | 1.080 | 1.184 |
| FBgn0039488 | 0.960 | 1.150 | 1.157 | 1.551 | 2.237 |
| FBgn0034634 | 0.960 | 0.887 | 0.958 | 1.000 | 1.271 |
| FBgn0039304 | 0.960 | 1.028 | 1.144 | 1.166 | 1.425 |
| FBgn0038126 | 0.960 | 0.616 | 0.697 | 0.910 | 1.616 |
| FBgn0051119 | 0.960 | 1.131 | 1.218 | 1.318 | 1.417 |
| FBgn0038533 | 0.960 | 0.826 | 0.888 | 1.084 | 1.235 |
| FBgn0250862 | 0.960 | 0.856 | 0.977 | 1.410 | 2.707 |
| FBgn0259740 | 0.960 | 1.263 | 1.291 | 1.443 | 1.847 |
| FBgn0002989 | 0.960 | 1.146 | 1.030 | 0.916 | 0.790 |
| FBgn0011589 | 0.959 | 1.008 | 1.008 | 1.227 | 1.508 |
| FBgn0036768 | 0.959 | 0.941 | 0.947 | 1.237 | 1.565 |
| FBgn0038252 | 0.959 | 1.187 | 1.162 | 0.638 | 0.108 |
| FBgn0030763 | 0.959 | 1.051 | 1.141 | 1.465 | 1.714 |
| FBgn0039240 | 0.959 | 1.057 | 0.933 | 0.853 | 0.723 |
| FBgn0026634 | 0.959 | 0.977 | 0.986 | 1.102 | 1.359 |
| FBgn0001276 | 0.959 | 0.996 | 1.223 | 1.352 | 1.590 |
| FBgn0036157 | 0.959 | 1.018 | 1.074 | 1.228 | 1.345 |
| FBgn0031910 | 0.959 | 0.919 | 1.437 | 2.976 | 4.141 |
| FBgn0051769 | 0.959 | 0.781 | 0.964 | 0.992 | 1.300 |
| FBgn0015625 | 0.959 | 1.352 | 1.182 | 0.557 | 0.078 |
| FBgn0000152 | 0.959 | 0.942 | 1.153 | 1.281 | 1.501 |
| FBgn0040102 | 0.959 | 1.030 | 1.159 | 1.682 | 2.087 |
| FBgn0037850 | 0.959 | 1.263 | 2.117 | 2.280 | 3.514 |
| FBgn0036396 | 0.958 | 0.744 | 0.814 | 1.047 | 1.223 |
| FBgn0058160 | 0.958 | 1.064 | 1.074 | 1.415 | 1.775 |
| FBgn0004378 | 0.958 | 1.292 | 1.032 | 0.708 | 0.410 |
| FBgn0029003 | 0.958 | 1.141 | 1.140 | 1.409 | 1.856 |
| FBgn0039319 | 0.958 | 1.071 | 1.062 | 1.770 | 2.699 |
| FBgn0039740 | 0.958 | 1.054 | 1.040 | 0.927 | 0.832 |
| FBgn0023416 | 0.958 | 0.885 | 0.897 | 1.102 | 1.541 |
| FBgn0038071 | 0.958 | 1.454 | 1.936 | 2.034 | 3.233 |
| FBgn0033214 | 0.958 | 0.887 | 0.885 | 0.968 | 1.085 |
| FBgn0036827 | 0.957 | 0.958 | 1.237 | 1.315 | 2.125 |
| FBgn0034951 | 0.957 | 0.874 | 0.885 | 0.942 | 1.101 |
| FBgn0039544 | 0.957 | 1.094 | 1.090 | 1.321 | 1.631 |
| FBgn0030912 | 0.957 | 1.038 | 1.143 | 1.745 | 2.225 |
| FBgn0034715 | 0.957 | 1.028 | 1.375 | 1.696 | 2.056 |
| FBgn0030410 | 0.957 | 0.998 | 0.951 | 0.847 | 0.463 |
| FBgn0035914 | 0.957 | 1.008 | 1.012 | 1.194 | 1.386 |
| FBgn0052412 | 0.957 | 0.773 | 0.994 | 1.175 | 1.401 |
| FBgn0019932 | 0.957 | 1.473 | 1.607 | 1.714 | 2.339 |
| FBgn0051223 | 0.957 | 0.759 | 0.844 | 0.979 | 1.547 |
| FBgn0034328 | 0.957 | 1.100 | 2.096 | 2.305 | 3.648 |
| FBgn0038499 | 0.957 | 1.039 | 1.100 | 1.153 | 1.216 |
| FBgn0030984 | 0.957 | 0.931 | 0.938 | 1.113 | 1.287 |
| FBgn0085435 | 0.957 | 0.734 | 0.823 | 1.451 | 1.964 |
| FBgn0030827 | 0.957 | 0.920 | 0.924 | 1.054 | 1.320 |
| FBgn0033189 | 0.956 | 0.872 | 1.008 | 1.027 | 1.247 |
| FBgn0259178 | 0.956 | 1.025 | 1.038 | 1.264 | 1.473 |
| FBgn0010038 | 0.956 | 2.277 | 5.455 | 6.142 | 10.345 |
| FBgn0032684 | 0.956 | 0.963 | 0.966 | 1.137 | 1.483 |
| FBgn0005616 | 0.956 | 0.990 | 1.012 | 1.063 | 1.251 |
| FBgn0039703 | 0.955 | 0.944 | 1.014 | 1.026 | 1.194 |
| FBgn0047095 | 0.955 | 0.912 | 1.072 | 1.100 | 1.327 |
| FBgn0034527 | 0.955 | 1.061 | 1.057 | 1.028 | 1.005 |
| FBgn0025628 | 0.955 | 1.142 | 1.139 | 1.222 | 1.351 |
| FBgn0015903 | 0.955 | 1.223 | 1.058 | 0.892 | 0.722 |
| FBgn0031020 | 0.955 | 1.137 | 1.052 | 1.024 | 0.748 |
| FBgn0039132 | 0.955 | 0.908 | 0.979 | 1.039 | 1.390 |
| FBgn0050493 | 0.955 | 0.988 | 1.056 | 1.106 | 1.173 |
| FBgn0031051 | 0.954 | 1.105 | 0.920 | 0.802 | 0.618 |
| FBgn0033945 | 0.954 | 1.265 | 1.385 | 1.693 | 2.809 |
| FBgn0028987 | 0.954 | 0.915 | 0.910 | 1.253 | 1.900 |
| FBgn0250835 | 0.954 | 1.178 | 1.606 | 1.672 | 2.287 |
| FBgn0034199 | 0.954 | 0.885 | 1.064 | 1.242 | 1.424 |
| FBgn0003612 | 0.954 | 1.028 | 0.853 | 0.775 | 0.593 |
| FBgn0033744 | 0.954 | 0.902 | 0.913 | 1.058 | 1.414 |
| FBgn0038741 | 0.953 | 1.002 | 1.020 | 1.188 | 1.324 |
| FBgn0261882 | 0.953 | 0.487 | 0.844 | 0.881 | 1.546 |
| FBgn0038894 | 0.953 | 0.805 | 1.182 | 1.536 | 1.913 |
| FBgn0032305 | 0.953 | 0.907 | 0.927 | 1.087 | 1.213 |
| FBgn0034612 | 0.953 | 0.908 | 0.928 | 1.002 | 1.055 |
| FBgn0052751 | 0.953 | 0.540 | 0.602 | 0.927 | 1.894 |
| FBgn0014020 | 0.953 | 0.883 | 0.891 | 0.985 | 1.220 |
| FBgn0038412 | 0.952 | 1.095 | 1.238 | 1.274 | 1.704 |
| FBgn0023395 | 0.952 | 1.202 | 0.938 | 0.541 | 0.227 |
| FBgn0024975 | 0.952 | 1.126 | 1.051 | 0.749 | 0.533 |
| FBgn0052383 | 0.952 | 0.421 | 0.565 | 0.643 | 1.237 |
| FBgn0035206 | 0.952 | 0.886 | 0.935 | 1.037 | 1.109 |
| FBgn0036629 | 0.951 | 1.061 | 1.047 | 1.419 | 1.840 |
| FBgn0053193 | 0.951 | 1.101 | 1.011 | 1.002 | 0.829 |
| FBgn0052672 | 0.951 | 0.864 | 0.999 | 1.129 | 1.263 |
| FBgn0039280 | 0.951 | 0.991 | 1.019 | 1.149 | 1.557 |
| FBgn0260229 | 0.951 | 0.991 | 1.019 | 1.149 | 1.557 |
| FBgn0010416 | 0.951 | 0.883 | 0.878 | 0.948 | 1.051 |
| FBgn0039557 | 0.951 | 0.877 | 1.050 | 1.146 | 1.314 |
| FBgn0029765 | 0.951 | 0.886 | 1.154 | 1.186 | 1.820 |
| FBgn0003205 | 0.951 | 1.089 | 1.211 | 1.251 | 1.384 |
| FBgn0032166 | 0.951 | 0.927 | 0.930 | 1.027 | 1.115 |
| FBgn0035612 | 0.951 | 1.100 | 1.192 | 1.868 | 3.795 |
| FBgn0038795 | 0.950 | 0.952 | 1.052 | 1.142 | 1.678 |
| FBgn0039286 | 0.950 | 0.996 | 0.981 | 1.470 | 1.987 |
| FBgn0046689 | 0.950 | 1.006 | 1.310 | 1.368 | 1.749 |
| FBgn0002707 | 0.950 | 1.027 | 1.083 | 1.163 | 1.550 |
| FBgn0085819 | 0.949 | 1.045 | 1.321 | 1.399 | 2.310 |
| FBgn0003141 | 0.949 | 0.814 | 0.941 | 0.984 | 1.434 |
| FBgn0037936 | 0.949 | 0.822 | 0.835 | 0.946 | 1.258 |
| FBgn0051300 | 0.949 | 0.740 | 0.717 | 1.031 | 1.466 |
| FBgn0011584 | 0.949 | 0.874 | 0.874 | 1.001 | 1.287 |
| FBgn0040323 | 0.949 | 0.841 | 0.964 | 1.259 | 1.459 |
| FBgn0037304 | 0.949 | 0.959 | 0.994 | 1.268 | 1.471 |
| FBgn0035696 | 0.949 | 0.947 | 0.938 | 1.172 | 1.420 |
| FBgn0030258 | 0.948 | 0.857 | 0.904 | 1.059 | 1.163 |
| FBgn0039656 | 0.948 | 1.166 | 1.121 | 1.689 | 2.497 |
| FBgn0042102 | 0.948 | 0.830 | 0.887 | 1.115 | 1.270 |
| FBgn0004907 | 0.948 | 0.980 | 1.048 | 1.085 | 1.381 |
| FBgn0003559 | 0.948 | 0.982 | 1.043 | 1.223 | 1.343 |
| FBgn0011705 | 0.948 | 0.916 | 0.913 | 1.243 | 1.552 |
| FBgn0036922 | 0.948 | 0.960 | 1.178 | 1.198 | 1.698 |
| FBgn0036294 | 0.948 | 0.997 | 1.148 | 1.163 | 1.388 |
| FBgn0039272 | 0.948 | 0.874 | 0.975 | 1.174 | 2.034 |
| FBgn0033397 | 0.948 | 0.927 | 0.889 | 1.347 | 1.992 |
| FBgn0031650 | 0.948 | 0.456 | 0.823 | 0.867 | 1.381 |
| FBgn0086698 | 0.947 | 0.953 | 0.944 | 1.086 | 1.350 |
| FBgn0051189 | 0.947 | 0.546 | 0.913 | 1.015 | 1.418 |
| FBgn0030800 | 0.947 | 1.016 | 1.113 | 1.282 | 1.402 |
| FBgn0039008 | 0.947 | 0.990 | 1.177 | 1.282 | 1.457 |
| FBgn0035265 | 0.947 | 1.021 | 0.917 | 0.903 | 0.763 |
| FBgn0036848 | 0.947 | 0.905 | 1.035 | 1.171 | 1.296 |
| FBgn0038354 | 0.947 | 0.954 | 0.952 | 1.271 | 1.564 |
| FBgn0052855 | 0.947 | 0.916 | 0.911 | 1.213 | 1.498 |
| FBgn0026737 | 0.947 | 0.936 | 1.112 | 1.157 | 1.737 |
| FBgn0039696 | 0.946 | 0.984 | 1.115 | 1.157 | 1.294 |
| FBgn0020416 | 0.946 | 0.778 | 0.760 | 0.958 | 1.255 |
| FBgn0052196 | 0.946 | 1.079 | 1.214 | 1.504 | 1.699 |
| FBgn0083966 | 0.946 | 0.830 | 0.927 | 0.995 | 1.477 |
| FBgn0036760 | 0.946 | 0.972 | 1.002 | 1.075 | 1.123 |
| FBgn0042085 | 0.946 | 1.081 | 0.854 | 0.712 | 0.503 |
| FBgn0020303 | 0.946 | 0.921 | 0.893 | 1.207 | 1.614 |
| FBgn0037150 | 0.946 | 0.897 | 0.995 | 1.085 | 1.641 |
| FBgn0004169 | 0.946 | 1.088 | 1.086 | 1.208 | 1.320 |
| FBgn0036749 | 0.946 | 1.029 | 1.044 | 1.264 | 1.870 |
| FBgn0260399 | 0.946 | 1.211 | 0.882 | 0.866 | 0.256 |
| FBgn0011754 | 0.946 | 0.991 | 1.056 | 1.107 | 1.166 |
| FBgn0262524 | 0.945 | 1.069 | 1.440 | 1.502 | 2.592 |
| FBgn0030157 | 0.945 | 0.846 | 0.849 | 1.011 | 1.418 |
| FBgn0028411 | 0.945 | 1.246 | 1.759 | 1.816 | 2.514 |
| FBgn0025615 | 0.945 | 0.875 | 0.985 | 1.106 | 1.212 |
| FBgn0032981 | 0.945 | 0.805 | 0.797 | 1.262 | 2.350 |
| FBgn0261284 | 0.945 | 0.938 | 1.284 | 1.435 | 1.767 |
| FBgn0022268 | 0.945 | 0.911 | 0.972 | 1.037 | 1.095 |
| FBgn0035769 | 0.944 | 1.063 | 1.211 | 1.313 | 1.447 |
| FBgn0030554 | 0.944 | 0.951 | 1.008 | 1.127 | 1.206 |
| FBgn0038922 | 0.944 | 1.109 | 0.988 | 0.863 | 0.749 |
| FBgn0034172 | 0.944 | 0.820 | 0.920 | 0.965 | 1.060 |
| FBgn0260940 | 0.944 | 0.803 | 0.909 | 0.916 | 1.082 |
| FBgn0037855 | 0.944 | 0.955 | 0.998 | 1.102 | 1.170 |
| FBgn0036336 | 0.944 | 1.011 | 1.060 | 1.100 | 1.145 |
| FBgn0003495 | 0.944 | 0.988 | 0.997 | 1.363 | 1.662 |
| FBgn0010278 | 0.944 | 1.066 | 1.061 | 1.214 | 1.561 |
| FBgn0031250 | 0.944 | 0.976 | 1.080 | 1.114 | 1.220 |
| FBgn0038631 | 0.944 | 1.313 | 1.299 | 1.567 | 1.830 |
| FBgn0037845 | 0.944 | 1.050 | 1.210 | 1.379 | 1.529 |
| FBgn0003002 | 0.944 | 0.750 | 0.722 | 0.983 | 1.372 |
| FBgn0031080 | 0.944 | 1.398 | 1.385 | 1.558 | 1.747 |
| FBgn0029912 | 0.943 | 1.116 | 1.087 | 1.073 | 0.946 |
| FBgn0012051 | 0.943 | 0.940 | 0.924 | 1.090 | 1.391 |
| FBgn0032472 | 0.943 | 0.768 | 0.907 | 0.975 | 1.588 |
| FBgn0035816 | 0.943 | 0.852 | 0.831 | 1.052 | 1.448 |
| FBgn0036124 | 0.943 | 1.088 | 1.066 | 1.276 | 1.547 |
| FBgn0039868 | 0.943 | 1.133 | 1.151 | 1.211 | 1.250 |
| FBgn0036433 | 0.943 | 0.887 | 0.926 | 1.128 | 1.260 |
| FBgn0035477 | 0.943 | 0.982 | 0.900 | 0.746 | 0.643 |
| FBgn0037244 | 0.943 | 0.977 | 0.979 | 1.037 | 1.192 |
| FBgn0038366 | 0.942 | 0.871 | 0.954 | 1.198 | 2.177 |
| FBgn0037167 | 0.942 | 1.096 | 1.261 | 1.276 | 1.503 |
| FBgn0053144 | 0.942 | 1.103 | 0.967 | 0.896 | 0.772 |
| FBgn0039307 | 0.942 | 0.934 | 1.078 | 1.513 | 1.785 |
| FBgn0035321 | 0.942 | 1.020 | 1.058 | 1.090 | 1.124 |
| FBgn0043900 | 0.942 | 1.049 | 0.855 | 0.807 | 0.599 |
| FBgn0038498 | 0.942 | 0.866 | 0.860 | 1.097 | 1.305 |
| FBgn0029737 | 0.942 | 1.063 | 0.972 | 0.916 | 0.835 |
| FBgn0038414 | 0.942 | 0.819 | 0.775 | 1.334 | 1.922 |
| FBgn0053139 | 0.942 | 1.123 | 0.969 | 0.862 | 0.726 |
| FBgn0030529 | 0.941 | 0.993 | 1.007 | 1.162 | 1.273 |
| FBgn0036318 | 0.941 | 1.014 | 1.022 | 1.027 | 1.064 |
| FBgn0032669 | 0.941 | 1.019 | 1.099 | 1.355 | 1.514 |
| FBgn0261396 | 0.941 | 1.048 | 1.043 | 0.966 | 0.910 |
| FBgn0263025 | 0.941 | 1.076 | 1.156 | 1.163 | 1.273 |
| FBgn0032780 | 0.941 | 1.062 | 1.144 | 1.206 | 1.277 |
| FBgn0083981 | 0.941 | 0.932 | 0.950 | 1.139 | 1.729 |
| FBgn0031021 | 0.941 | 0.888 | 0.928 | 0.998 | 1.045 |
| FBgn0014469 | 0.940 | 0.894 | 0.888 | 1.100 | 1.286 |
| FBgn0034365 | 0.940 | 0.973 | 1.007 | 1.319 | 1.533 |
| FBgn0051157 | 0.940 | 1.225 | 1.427 | 1.621 | 2.873 |
| FBgn0034295 | 0.940 | 1.028 | 1.195 | 1.376 | 1.529 |
| FBgn0000357 | 0.940 | 0.932 | 1.159 | 1.218 | 1.453 |
| FBgn0052054 | 0.940 | 0.827 | 1.033 | 1.092 | 1.300 |
| FBgn0015789 | 0.940 | 0.924 | 0.954 | 1.194 | 1.355 |
| FBgn0036697 | 0.940 | 0.884 | 0.881 | 0.948 | 1.006 |
| FBgn0020521 | 0.939 | 1.097 | 1.123 | 1.334 | 1.475 |
| FBgn0037383 | 0.939 | 1.025 | 0.965 | 0.707 | 0.548 |
| FBgn0039767 | 0.939 | 1.055 | 1.040 | 0.819 | 0.662 |
| FBgn0039467 | 0.939 | 0.855 | 0.853 | 1.003 | 1.394 |
| FBgn0036501 | 0.939 | 0.836 | 0.813 | 1.016 | 1.262 |
| FBgn0034538 | 0.939 | 0.912 | 1.003 | 1.022 | 1.121 |
| FBgn0028853 | 0.939 | 0.774 | 1.011 | 1.155 | 1.362 |
| FBgn0010470 | 0.939 | 0.868 | 1.044 | 1.145 | 1.300 |
| FBgn0015818 | 0.939 | 1.041 | 1.054 | 0.953 | 0.774 |
| FBgn0260431 | 0.939 | 1.023 | 1.119 | 1.171 | 1.632 |
| FBgn0037473 | 0.939 | 0.881 | 0.981 | 0.985 | 1.136 |
| FBgn0033317 | 0.939 | 1.000 | 0.985 | 0.831 | 0.725 |
| FBgn0025627 | 0.939 | 1.002 | 0.963 | 1.259 | 1.659 |
| FBgn0026418 | 0.938 | 1.094 | 0.991 | 0.943 | 0.850 |
| FBgn0052105 | 0.938 | 0.956 | 1.026 | 1.692 | 2.139 |
| FBgn0036101 | 0.938 | 0.791 | 0.757 | 1.112 | 1.479 |
| FBgn0004394 | 0.938 | 0.876 | 1.171 | 1.305 | 1.570 |
| FBgn0011741 | 0.938 | 1.023 | 1.119 | 1.365 | 1.514 |
| FBgn0010516 | 0.938 | 1.011 | 1.076 | 1.168 | 1.233 |
| FBgn0039635 | 0.938 | 1.022 | 1.038 | 0.924 | 0.768 |
| FBgn0033491 | 0.938 | 0.824 | 0.897 | 1.059 | 1.158 |
| FBgn0051036 | 0.938 | 1.026 | 1.277 | 1.386 | 2.519 |
| FBgn0035993 | 0.938 | 1.242 | 1.142 | 1.040 | 0.952 |
| FBgn0030061 | 0.937 | 1.111 | 1.104 | 1.164 | 1.283 |
| FBgn0030240 | 0.937 | 1.099 | 1.038 | 1.037 | 0.899 |
| FBgn0036725 | 0.937 | 0.846 | 0.803 | 1.128 | 1.539 |
| FBgn0261839 | 0.937 | 0.978 | 1.131 | 1.144 | 1.341 |
| FBgn0036910 | 0.937 | 1.068 | 0.917 | 0.896 | 0.721 |
| FBgn0050026 | 0.937 | 0.825 | 0.998 | 0.996 | 1.324 |
| FBgn0037010 | 0.937 | 1.043 | 1.008 | 0.990 | 0.960 |
| FBgn0032682 | 0.937 | 0.908 | 0.869 | 1.246 | 1.639 |
| FBgn0027111 | 0.937 | 0.967 | 0.993 | 1.162 | 1.269 |
| FBgn0038058 | 0.937 | 1.052 | 1.080 | 1.208 | 1.696 |
| FBgn0036368 | 0.937 | 1.240 | 1.172 | 1.658 | 2.512 |
| FBgn0034143 | 0.936 | 0.943 | 0.926 | 1.141 | 1.341 |
| FBgn0083970 | 0.936 | 1.168 | 1.155 | 1.304 | 1.445 |
| FBgn0000057 | 0.936 | 1.126 | 1.045 | 0.869 | 0.763 |
| FBgn0261049 | 0.936 | 0.964 | 0.955 | 1.028 | 1.113 |
| FBgn0036765 | 0.936 | 0.942 | 1.099 | 1.403 | 1.592 |
| FBgn0028394 | 0.936 | 0.951 | 1.051 | 1.131 | 1.215 |
| FBgn0038079 | 0.936 | 1.125 | 1.396 | 1.413 | 2.173 |
| FBgn0033130 | 0.936 | 1.019 | 1.233 | 1.251 | 1.886 |
| FBgn0036838 | 0.936 | 1.109 | 1.152 | 1.328 | 2.029 |
| FBgn0036757 | 0.935 | 1.082 | 1.133 | 1.713 | 3.636 |
| FBgn0027093 | 0.935 | 0.946 | 0.932 | 1.033 | 1.210 |
| FBgn0051030 | 0.935 | 0.962 | 0.978 | 1.106 | 1.186 |
| FBgn0039461 | 0.935 | 1.065 | 1.021 | 0.805 | 0.676 |
| FBgn0011694 | 0.935 | 0.721 | 0.843 | 0.962 | 1.773 |
| FBgn0038476 | 0.935 | 1.122 | 1.124 | 1.060 | 0.882 |
| FBgn0031940 | 0.935 | 0.962 | 0.803 | 1.906 | 3.274 |
| FBgn0038749 | 0.934 | 0.858 | 1.055 | 1.063 | 1.334 |
| FBgn0260234 | 0.934 | 0.858 | 1.055 | 1.063 | 1.334 |
| FBgn0031598 | 0.934 | 0.910 | 0.888 | 1.027 | 1.224 |
| FBgn0032204 | 0.934 | 0.988 | 1.141 | 1.156 | 1.633 |
| FBgn0029827 | 0.934 | 0.896 | 0.969 | 1.214 | 1.355 |
| FBgn0037020 | 0.934 | 0.927 | 1.089 | 1.107 | 1.298 |
| FBgn0032627 | 0.934 | 1.240 | 1.511 | 1.912 | 2.178 |
| FBgn0039915 | 0.934 | 0.783 | 0.788 | 1.187 | 2.389 |
| FBgn0034889 | 0.934 | 0.910 | 0.964 | 1.310 | 1.521 |
| FBgn0033139 | 0.934 | 0.940 | 1.196 | 1.189 | 1.694 |
| FBgn0052428 | 0.934 | 0.915 | 1.028 | 1.263 | 1.403 |
| FBgn0034293 | 0.933 | 0.997 | 1.075 | 1.730 | 4.052 |
| FBgn0051207 | 0.933 | 0.894 | 0.979 | 1.056 | 1.611 |
| FBgn0003888 | 0.933 | 1.080 | 1.003 | 1.005 | 0.863 |
| FBgn0259975 | 0.933 | 2.132 | 2.635 | 2.737 | 4.612 |
| FBgn0027341 | 0.933 | 0.931 | 0.924 | 1.247 | 1.494 |
| FBgn0001104 | 0.933 | 1.036 | 1.155 | 1.480 | 1.665 |
| FBgn0033348 | 0.933 | 1.160 | 1.170 | 1.711 | 3.379 |
| FBgn0001185 | 0.933 | 0.910 | 1.077 | 1.088 | 1.588 |
| FBgn0036887 | 0.933 | 0.908 | 0.983 | 0.998 | 1.076 |
| FBgn0260991 | 0.933 | 1.325 | 1.065 | 0.435 | 0.074 |
| FBgn0033668 | 0.932 | 0.959 | 1.179 | 1.706 | 4.261 |
| FBgn0031472 | 0.932 | 0.594 | 0.625 | 0.717 | 1.131 |
| FBgn0085433 | 0.932 | 1.004 | 0.992 | 1.098 | 1.345 |
| FBgn0035806 | 0.932 | 1.120 | 1.226 | 1.232 | 1.367 |
| FBgn0036058 | 0.932 | 0.945 | 1.030 | 1.129 | 1.203 |
| FBgn0027538 | 0.932 | 0.997 | 0.973 | 1.165 | 1.583 |
| FBgn0051690 | 0.932 | 1.021 | 0.996 | 1.144 | 1.386 |
| FBgn0067782 | 0.932 | 1.061 | 1.034 | 0.823 | 0.694 |
| FBgn0037703 | 0.932 | 0.946 | 0.915 | 1.139 | 1.382 |
| FBgn0033980 | 0.932 | 0.700 | 0.646 | 1.162 | 1.651 |
| FBgn0039617 | 0.932 | 0.782 | 0.829 | 1.013 | 1.794 |
| FBgn0032216 | 0.932 | 1.063 | 1.059 | 0.899 | 0.790 |
| FBgn0031710 | 0.931 | 0.772 | 0.904 | 0.900 | 1.192 |
| FBgn0015618 | 0.931 | 0.939 | 0.927 | 1.008 | 1.103 |
| FBgn0035677 | 0.931 | 1.038 | 1.019 | 1.134 | 1.353 |
| FBgn0017414 | 0.931 | 0.841 | 0.970 | 1.091 | 1.196 |
| FBgn0036819 | 0.931 | 0.890 | 0.899 | 0.981 | 1.277 |
| FBgn0028424 | 0.931 | 0.776 | 0.854 | 1.168 | 1.345 |
| FBgn0036550 | 0.931 | 0.971 | 1.022 | 1.413 | 1.648 |
| FBgn0083952 | 0.931 | 0.687 | 0.723 | 0.751 | 0.976 |
| FBgn0003353 | 0.931 | 0.966 | 0.921 | 1.177 | 1.545 |
| FBgn0030354 | 0.930 | 1.096 | 0.909 | 0.844 | 0.676 |
| FBgn0051087 | 0.930 | 1.305 | 1.612 | 1.621 | 2.028 |
| FBgn0032192 | 0.930 | 0.671 | 1.241 | 1.419 | 1.938 |
| FBgn0038465 | 0.930 | 0.862 | 0.839 | 1.064 | 1.269 |
| FBgn0052641 | 0.930 | 0.799 | 0.915 | 1.109 | 2.212 |
| FBgn0050147 | 0.930 | 1.001 | 1.084 | 1.094 | 1.386 |
| FBgn0034198 | 0.930 | 0.840 | 0.843 | 0.983 | 1.436 |
| FBgn0011570 | 0.930 | 1.007 | 1.083 | 1.209 | 1.286 |
| FBgn0259722 | 0.930 | 0.881 | 0.985 | 0.981 | 1.223 |
| FBgn0067783 | 0.929 | 1.050 | 1.016 | 0.843 | 0.744 |
| FBgn0032088 | 0.929 | 0.983 | 1.252 | 1.240 | 1.697 |
| FBgn0032586 | 0.929 | 1.034 | 0.964 | 0.828 | 0.749 |
| FBgn0035089 | 0.929 | 0.903 | 1.234 | 1.232 | 2.122 |
| FBgn0016131 | 0.929 | 0.865 | 1.117 | 1.105 | 1.545 |
| FBgn0036806 | 0.929 | 0.916 | 0.919 | 1.103 | 1.226 |
| FBgn0052640 | 0.929 | 0.815 | 0.933 | 1.120 | 2.219 |
| FBgn0038095 | 0.929 | 1.149 | 1.705 | 1.705 | 2.492 |
| FBgn0000316 | 0.929 | 0.881 | 0.985 | 0.981 | 1.228 |
| FBgn0038930 | 0.929 | 0.930 | 0.990 | 1.132 | 1.210 |
| FBgn0031282 | 0.929 | 0.938 | 0.956 | 1.094 | 1.176 |
| FBgn0038734 | 0.929 | 0.973 | 1.021 | 1.392 | 1.611 |
| FBgn0039820 | 0.928 | 0.706 | 0.726 | 0.891 | 0.988 |
| FBgn0035812 | 0.928 | 1.137 | 1.044 | 1.537 | 2.306 |
| FBgn0014022 | 0.928 | 1.018 | 1.194 | 1.186 | 1.484 |
| FBgn0038609 | 0.928 | 1.072 | 1.166 | 1.161 | 1.339 |
| FBgn0040732 | 0.928 | 0.603 | 0.820 | 0.910 | 1.093 |
| FBgn0039609 | 0.928 | 0.853 | 0.863 | 1.216 | 1.449 |
| FBgn0261722 | 0.928 | 0.952 | 1.081 | 1.224 | 1.330 |
| FBgn0030005 | 0.928 | 0.998 | 1.048 | 1.288 | 2.306 |
| FBgn0025140 | 0.928 | 0.951 | 1.098 | 1.280 | 1.406 |
| FBgn0029997 | 0.928 | 1.039 | 0.881 | 0.765 | 0.641 |
| FBgn0040929 | 0.928 | 0.899 | 0.896 | 1.093 | 1.712 |
| FBgn0054054 | 0.928 | 0.487 | 0.859 | 0.903 | 2.234 |
| FBgn0038815 | 0.928 | 1.308 | 1.180 | 1.185 | 0.868 |
| FBgn0035213 | 0.928 | 0.935 | 0.878 | 1.193 | 1.588 |
| FBgn0085476 | 0.927 | 0.879 | 0.829 | 1.208 | 1.570 |
| FBgn0035449 | 0.927 | 1.035 | 1.039 | 1.104 | 1.144 |
| FBgn0013432 | 0.927 | 0.739 | 0.865 | 0.881 | 1.342 |
| FBgn0026077 | 0.927 | 0.920 | 0.884 | 1.094 | 1.536 |
| FBgn0028546 | 0.927 | 1.057 | 1.153 | 1.401 | 1.534 |
| FBgn0002775 | 0.926 | 1.132 | 1.061 | 0.854 | 0.744 |
| FBgn0034387 | 0.926 | 0.790 | 0.743 | 0.981 | 1.333 |
| FBgn0034434 | 0.926 | 0.898 | 0.898 | 1.230 | 1.455 |
| FBgn0036493 | 0.926 | 0.962 | 0.918 | 1.226 | 1.527 |
| FBgn0030055 | 0.926 | 1.024 | 1.028 | 1.144 | 1.217 |
| FBgn0037655 | 0.926 | 1.142 | 1.104 | 1.084 | 0.871 |
| FBgn0051673 | 0.926 | 0.907 | 0.937 | 1.133 | 1.241 |
| FBgn0037765 | 0.926 | 0.873 | 0.910 | 1.068 | 1.152 |
| FBgn0038246 | 0.926 | 0.896 | 0.863 | 1.070 | 1.537 |
| FBgn0030749 | 0.926 | 0.986 | 0.976 | 1.071 | 1.151 |
| FBgn0032343 | 0.926 | 0.886 | 1.088 | 1.169 | 1.338 |
| FBgn0036154 | 0.926 | 0.763 | 0.817 | 0.897 | 1.399 |
| FBgn0053096 | 0.926 | 1.131 | 0.951 | 0.808 | 0.669 |
| FBgn0086656 | 0.926 | 0.950 | 1.021 | 1.017 | 1.144 |
| FBgn0037338 | 0.925 | 0.895 | 0.806 | 1.250 | 1.859 |
| FBgn0025720 | 0.925 | 0.929 | 0.927 | 0.942 | 0.975 |
| FBgn0037518 | 0.925 | 0.798 | 0.836 | 1.040 | 1.151 |
| FBgn0034165 | 0.925 | 1.019 | 1.162 | 1.181 | 1.723 |
| FBgn0032200 | 0.925 | 1.214 | 1.219 | 1.290 | 1.557 |
| FBgn0036223 | 0.925 | 0.950 | 0.998 | 1.175 | 1.268 |
| FBgn0035439 | 0.925 | 1.216 | 1.305 | 1.498 | 2.555 |
| FBgn0034002 | 0.925 | 0.938 | 1.205 | 1.186 | 1.650 |
| FBgn0038755 | 0.925 | 0.890 | 0.857 | 1.022 | 1.254 |
| FBgn0038325 | 0.924 | 0.926 | 1.063 | 1.205 | 1.313 |
| FBgn0039914 | 0.924 | 0.785 | 0.880 | 0.967 | 1.659 |
| FBgn0031643 | 0.924 | 0.955 | 0.869 | 1.519 | 2.113 |
| FBgn0032731 | 0.924 | 0.974 | 0.995 | 1.052 | 1.346 |
| FBgn0036970 | 0.924 | 0.826 | 0.811 | 1.031 | 1.198 |
| FBgn0031762 | 0.924 | 0.890 | 1.007 | 1.449 | 1.680 |
| FBgn0004872 | 0.924 | 0.920 | 0.934 | 2.095 | 2.841 |
| FBgn0052199 | 0.924 | 0.866 | 0.818 | 1.117 | 1.418 |
| FBgn0037922 | 0.924 | 1.111 | 1.143 | 0.938 | 0.737 |
| FBgn0038927 | 0.924 | 0.986 | 0.962 | 0.879 | 0.836 |
| FBgn0040262 | 0.924 | 0.428 | 0.374 | 0.674 | 1.332 |
| FBgn0030331 | 0.924 | 1.057 | 1.307 | 1.587 | 1.787 |
| FBgn0037019 | 0.924 | 0.791 | 0.798 | 1.069 | 1.240 |
| FBgn0031974 | 0.924 | 1.073 | 1.343 | 1.524 | 1.729 |
| FBgn0031304 | 0.924 | 1.045 | 0.897 | 0.823 | 0.706 |
| FBgn0031491 | 0.923 | 0.938 | 1.054 | 1.097 | 1.194 |
| FBgn0034269 | 0.923 | 1.010 | 0.934 | 0.940 | 0.766 |
| FBgn0034010 | 0.923 | 1.031 | 1.156 | 1.146 | 1.376 |
| FBgn0010772 | 0.923 | 1.033 | 1.090 | 1.299 | 1.407 |
| FBgn0039856 | 0.923 | 1.028 | 1.043 | 0.908 | 0.796 |
| FBgn0039770 | 0.923 | 0.728 | 0.925 | 0.982 | 1.155 |
| FBgn0261270 | 0.923 | 1.023 | 1.051 | 0.913 | 0.743 |
| FBgn0011762 | 0.923 | 0.822 | 0.816 | 1.930 | 2.657 |
| FBgn0027106 | 0.922 | 0.881 | 1.044 | 1.132 | 1.258 |
| FBgn0035495 | 0.922 | 0.866 | 0.882 | 1.034 | 1.120 |
| FBgn0050035 | 0.922 | 0.995 | 1.104 | 1.206 | 1.289 |
| FBgn0038464 | 0.922 | 1.022 | 0.971 | 0.866 | 0.810 |
| FBgn0038490 | 0.922 | 1.018 | 0.959 | 1.225 | 1.621 |
| FBgn0040250 | 0.922 | 1.068 | 1.418 | 1.662 | 3.973 |
| FBgn0002891 | 0.922 | 1.133 | 1.020 | 1.529 | 2.332 |
| FBgn0040398 | 0.922 | 0.959 | 1.141 | 1.254 | 1.391 |
| FBgn0052683 | 0.921 | 0.960 | 0.959 | 1.125 | 1.710 |
| FBgn0000384 | 0.921 | 0.948 | 1.273 | 1.754 | 2.038 |
| FBgn0004650 | 0.921 | 1.133 | 0.781 | 0.765 | 0.357 |
| FBgn0039993 | 0.921 | 0.952 | 0.980 | 1.098 | 1.656 |
| FBgn0016047 | 0.921 | 1.274 | 1.226 | 1.677 | 2.039 |
| FBgn0028645 | 0.921 | 0.812 | 0.813 | 1.324 | 3.151 |
| FBgn0261929 | 0.921 | 0.901 | 1.005 | 1.012 | 1.390 |
| FBgn0036376 | 0.921 | 1.091 | 1.025 | 1.027 | 0.936 |
| FBgn0035010 | 0.921 | 0.853 | 0.838 | 0.999 | 1.484 |
| FBgn0032381 | 0.921 | 1.021 | 0.989 | 1.179 | 1.367 |
| FBgn0033138 | 0.921 | 0.980 | 1.128 | 1.171 | 1.298 |
| FBgn0039690 | 0.921 | 0.956 | 0.948 | 1.170 | 1.319 |
| FBgn0032987 | 0.921 | 0.860 | 1.055 | 1.103 | 1.277 |
| FBgn0028703 | 0.920 | 0.923 | 0.932 | 1.126 | 1.240 |
| FBgn0027559 | 0.920 | 1.083 | 1.121 | 1.224 | 1.781 |
| FBgn0052447 | 0.920 | 0.880 | 1.029 | 1.223 | 1.344 |
| FBgn0034417 | 0.920 | 0.916 | 0.895 | 1.061 | 1.200 |
| FBgn0021873 | 0.920 | 1.178 | 0.969 | 0.723 | 0.558 |
| FBgn0262444 | 0.920 | 1.426 | 1.148 | 1.175 | 0.679 |
| FBgn0004429 | 0.920 | 0.786 | 1.025 | 1.188 | 1.364 |
| FBgn0016696 | 0.920 | 1.019 | 1.020 | 1.285 | 1.449 |
| FBgn0035998 | 0.920 | 0.959 | 0.911 | 1.118 | 1.493 |
| FBgn0035006 | 0.919 | 1.408 | 1.147 | 0.755 | 0.530 |
| FBgn0052111 | 0.919 | 0.967 | 0.911 | 1.313 | 2.425 |
| FBgn0034093 | 0.919 | 0.999 | 1.051 | 1.348 | 1.501 |
| FBgn0261461 | 0.919 | 1.040 | 0.980 | 1.311 | 2.116 |
| FBgn0053129 | 0.919 | 1.117 | 0.984 | 0.809 | 0.700 |
| FBgn0011236 | 0.919 | 1.110 | 0.972 | 0.864 | 0.764 |
| FBgn0034572 | 0.919 | 0.989 | 0.977 | 1.058 | 1.281 |
| FBgn0039678 | 0.919 | 1.037 | 0.837 | 0.286 | 0.013 |
| FBgn0003884 | 0.919 | 1.182 | 1.040 | 0.719 | 0.557 |
| FBgn0034063 | 0.919 | 0.702 | 0.832 | 0.880 | 0.985 |
| FBgn0035452 | 0.919 | 0.802 | 1.108 | 1.316 | 1.540 |
| FBgn0037737 | 0.919 | 0.937 | 1.029 | 1.132 | 1.927 |
| FBgn0031022 | 0.919 | 0.846 | 0.960 | 1.090 | 2.083 |
| FBgn0033187 | 0.919 | 0.974 | 0.906 | 0.770 | 0.700 |
| FBgn0020513 | 0.919 | 0.922 | 0.965 | 1.059 | 1.107 |
| FBgn0003965 | 0.918 | 1.153 | 1.167 | 1.372 | 1.487 |
| FBgn0036196 | 0.918 | 1.031 | 1.001 | 0.880 | 0.820 |
| FBgn0031091 | 0.918 | 1.197 | 1.144 | 0.589 | 0.288 |
| FBgn0039559 | 0.918 | 1.159 | 0.863 | 0.814 | 0.533 |
| FBgn0052843 | 0.918 | 0.915 | 0.963 | 1.195 | 1.311 |
| FBgn0004644 | 0.918 | 1.116 | 1.107 | 1.197 | 1.483 |
| FBgn0031220 | 0.918 | 1.006 | 0.958 | 1.191 | 1.450 |
| FBgn0031399 | 0.918 | 1.003 | 0.922 | 0.888 | 0.825 |
| FBgn0261385 | 0.918 | 1.155 | 1.146 | 0.783 | 0.568 |
| FBgn0033374 | 0.918 | 0.911 | 1.293 | 1.455 | 1.752 |
| FBgn0023001 | 0.918 | 0.994 | 0.967 | 1.193 | 1.373 |
| FBgn0038830 | 0.917 | 1.014 | 1.231 | 1.225 | 1.509 |
| FBgn0004395 | 0.917 | 0.898 | 0.827 | 1.371 | 1.813 |
| FBgn0042134 | 0.917 | 1.009 | 0.900 | 0.913 | 0.697 |
| FBgn0038959 | 0.917 | 1.056 | 1.023 | 1.373 | 1.631 |
| FBgn0261245 | 0.917 | 1.027 | 1.249 | 1.352 | 1.521 |
| FBgn0011723 | 0.917 | 1.004 | 1.033 | 1.205 | 1.292 |
| FBgn0028986 | 0.917 | 0.852 | 0.825 | 0.955 | 1.265 |
| FBgn0051248 | 0.916 | 0.918 | 0.920 | 1.130 | 1.255 |
| FBgn0039196 | 0.916 | 0.957 | 0.808 | 1.412 | 2.454 |
| FBgn0038145 | 0.916 | 0.972 | 0.994 | 0.836 | 0.704 |
| FBgn0024913 | 0.916 | 0.941 | 1.054 | 1.342 | 3.008 |
| FBgn0032382 | 0.916 | 0.917 | 0.934 | 1.150 | 1.268 |
| FBgn0050203 | 0.916 | 0.897 | 0.955 | 1.025 | 1.564 |
| FBgn0051031 | 0.916 | 1.030 | 0.855 | 1.712 | 2.618 |
| FBgn0053514 | 0.916 | 0.812 | 0.806 | 1.061 | 1.221 |
| FBgn0043456 | 0.916 | 1.002 | 0.958 | 1.206 | 1.438 |
| FBgn0052155 | 0.916 | 0.777 | 0.717 | 1.088 | 1.416 |
| FBgn0004009 | 0.915 | 1.018 | 0.951 | 1.329 | 2.355 |
| FBgn0030778 | 0.915 | 0.969 | 0.951 | 1.178 | 1.337 |
| FBgn0030334 | 0.915 | 1.055 | 1.231 | 1.791 | 2.057 |
| FBgn0262468 | 0.915 | 0.916 | 1.001 | 0.997 | 1.264 |
| FBgn0261059 | 0.915 | 0.695 | 0.778 | 1.148 | 1.326 |
| FBgn0035471 | 0.915 | 0.828 | 0.835 | 0.983 | 1.065 |
| FBgn0259896 | 0.915 | 1.085 | 1.502 | 2.081 | 2.415 |
| FBgn0260855 | 0.915 | 0.996 | 1.017 | 1.179 | 1.262 |
| FBgn0051176 | 0.915 | 0.878 | 0.856 | 1.019 | 1.510 |
| FBgn0036003 | 0.915 | 1.090 | 0.967 | 0.741 | 0.626 |
| FBgn0040373 | 0.915 | 0.866 | 1.541 | 1.910 | 2.400 |
| FBgn0030421 | 0.915 | 0.874 | 0.832 | 0.999 | 1.311 |
| FBgn0034500 | 0.914 | 0.995 | 1.176 | 1.323 | 1.451 |
| FBgn0262115 | 0.914 | 1.048 | 0.969 | 1.281 | 1.720 |
| FBgn0034518 | 0.914 | 0.876 | 0.810 | 1.066 | 1.481 |
| FBgn0033627 | 0.914 | 0.925 | 1.100 | 1.161 | 1.298 |
| FBgn0086913 | 0.914 | 0.864 | 0.977 | 1.005 | 1.101 |
| FBgn0038610 | 0.914 | 1.089 | 1.300 | 1.297 | 1.551 |
| FBgn0027101 | 0.914 | 0.882 | 0.854 | 1.081 | 1.808 |
| FBgn0037092 | 0.914 | 0.982 | 0.917 | 1.175 | 1.691 |
| FBgn0036187 | 0.913 | 1.112 | 1.397 | 1.475 | 1.711 |
| FBgn0034154 | 0.913 | 0.854 | 1.113 | 1.090 | 1.473 |
| FBgn0030114 | 0.913 | 1.165 | 1.056 | 0.894 | 0.805 |
| FBgn0032145 | 0.913 | 0.833 | 0.809 | 1.128 | 1.344 |
| FBgn0034519 | 0.913 | 0.873 | 0.807 | 1.056 | 1.476 |
| FBgn0038172 | 0.913 | 0.849 | 0.893 | 1.100 | 1.198 |
| FBgn0085822 | 0.913 | 1.671 | 2.491 | 2.872 | 7.988 |
| FBgn0038680 | 0.913 | 0.855 | 0.844 | 1.167 | 1.367 |
| FBgn0033153 | 0.913 | 1.028 | 1.422 | 1.980 | 2.291 |
| FBgn0034537 | 0.912 | 0.818 | 0.835 | 1.052 | 1.164 |
| FBgn0037778 | 0.912 | 0.953 | 1.133 | 1.115 | 1.635 |
| FBgn0250907 | 0.912 | 1.163 | 1.326 | 1.397 | 2.400 |
| FBgn0038371 | 0.912 | 1.087 | 1.503 | 1.585 | 1.950 |
| FBgn0052406 | 0.912 | 0.807 | 0.907 | 0.943 | 1.520 |
| FBgn0030357 | 0.912 | 0.950 | 1.055 | 1.146 | 2.010 |
| FBgn0051321 | 0.912 | 0.995 | 0.939 | 1.148 | 1.464 |
| FBgn0038842 | 0.912 | 1.031 | 1.046 | 1.351 | 1.514 |
| FBgn0260750 | 0.912 | 0.967 | 0.893 | 0.701 | 0.612 |
| FBgn0033593 | 0.912 | 1.737 | 1.703 | 2.153 | 2.451 |
| FBgn0033550 | 0.912 | 0.883 | 0.933 | 0.931 | 1.100 |
| FBgn0087013 | 0.912 | 1.032 | 0.957 | 0.934 | 0.875 |
| FBgn0261564 | 0.911 | 1.048 | 0.929 | 0.919 | 0.800 |
| FBgn0002938 | 0.911 | 0.831 | 0.803 | 1.038 | 1.212 |
| FBgn0038129 | 0.911 | 1.040 | 1.186 | 1.313 | 1.413 |
| FBgn0035400 | 0.911 | 1.054 | 0.885 | 0.777 | 0.660 |
| FBgn0003867 | 0.911 | 0.972 | 1.129 | 1.120 | 1.326 |
| FBgn0001083 | 0.911 | 0.909 | 0.845 | 1.115 | 1.773 |
| FBgn0030748 | 0.911 | 0.865 | 0.908 | 1.326 | 1.534 |
| FBgn0022981 | 0.911 | 1.471 | 1.173 | 0.468 | 0.143 |
| FBgn0038828 | 0.911 | 0.782 | 0.845 | 0.963 | 1.774 |
| FBgn0030077 | 0.911 | 1.183 | 1.116 | 1.360 | 1.775 |
| FBgn0036775 | 0.910 | 1.077 | 1.113 | 0.864 | 0.671 |
| FBgn0037755 | 0.910 | 0.901 | 0.822 | 1.132 | 1.513 |
| FBgn0086676 | 0.910 | 1.032 | 1.058 | 1.198 | 1.264 |
| FBgn0035733 | 0.910 | 0.866 | 0.776 | 1.106 | 1.765 |
| FBgn0037935 | 0.909 | 0.749 | 0.684 | 0.976 | 1.272 |
| FBgn0033821 | 0.909 | 1.015 | 0.951 | 1.226 | 1.932 |
| FBgn0036999 | 0.909 | 0.988 | 0.954 | 1.076 | 1.296 |
| FBgn0051778 | 0.909 | 0.666 | 0.884 | 0.849 | 1.304 |
| FBgn0039817 | 0.909 | 0.619 | 0.593 | 1.001 | 2.571 |
| FBgn0028526 | 0.909 | 0.870 | 0.800 | 1.058 | 1.413 |
| FBgn0035091 | 0.909 | 0.951 | 0.924 | 1.023 | 1.163 |
| FBgn0038460 | 0.909 | 1.042 | 1.238 | 1.209 | 1.708 |
| FBgn0046322 | 0.909 | 1.114 | 1.283 | 1.482 | 3.165 |
| FBgn0010314 | 0.909 | 1.098 | 1.002 | 0.376 | 0.083 |
| FBgn0039523 | 0.909 | 1.236 | 1.042 | 1.729 | 2.992 |
| FBgn0032793 | 0.909 | 0.997 | 1.138 | 1.155 | 1.815 |
| FBgn0035158 | 0.909 | 0.896 | 0.842 | 1.078 | 1.697 |
| FBgn0040466 | 0.909 | 1.094 | 1.322 | 1.495 | 1.648 |
| FBgn0003326 | 0.909 | 0.993 | 1.152 | 1.649 | 1.869 |
| FBgn0050008 | 0.908 | 0.812 | 0.790 | 0.983 | 1.119 |
| FBgn0024191 | 0.908 | 1.177 | 0.995 | 0.571 | 0.379 |
| FBgn0035445 | 0.908 | 1.337 | 1.314 | 1.766 | 2.040 |
| FBgn0013433 | 0.908 | 0.902 | 0.918 | 1.100 | 1.189 |
| FBgn0026878 | 0.908 | 0.736 | 0.964 | 1.074 | 1.234 |
| FBgn0023546 | 0.908 | 0.935 | 0.788 | 1.422 | 2.073 |
| FBgn0260432 | 0.908 | 0.935 | 0.788 | 1.422 | 2.073 |
| FBgn0000629 | 0.908 | 0.915 | 0.963 | 1.103 | 1.165 |
| FBgn0054056 | 0.908 | 0.853 | 1.135 | 1.235 | 1.446 |
| FBgn0085470 | 0.908 | 1.230 | 1.136 | 0.919 | 0.821 |
| FBgn0250903 | 0.908 | 1.230 | 1.136 | 0.919 | 0.821 |
| FBgn0085313 | 0.908 | 0.845 | 1.091 | 1.120 | 1.350 |
| FBgn0260006 | 0.908 | 1.038 | 1.004 | 1.125 | 1.366 |
| FBgn0032601 | 0.908 | 1.242 | 1.160 | 1.465 | 1.852 |
| FBgn0035623 | 0.908 | 1.295 | 1.236 | 1.907 | 2.343 |
| FBgn0037707 | 0.908 | 1.110 | 1.112 | 0.745 | 0.543 |
| FBgn0031914 | 0.907 | 0.935 | 0.940 | 1.128 | 1.227 |
| FBgn0035041 | 0.907 | 0.896 | 0.855 | 1.033 | 1.214 |
| FBgn0053109 | 0.907 | 0.962 | 0.996 | 1.082 | 1.642 |
| FBgn0037908 | 0.907 | 1.008 | 1.279 | 1.351 | 1.567 |
| FBgn0050047 | 0.907 | 0.825 | 0.854 | 0.860 | 1.015 |
| FBgn0027581 | 0.907 | 1.061 | 1.095 | 1.285 | 1.371 |
| FBgn0259966 | 0.907 | 1.320 | 1.372 | 1.645 | 3.143 |
| FBgn0036969 | 0.907 | 0.864 | 0.805 | 1.026 | 1.295 |
| FBgn0040992 | 0.907 | 1.061 | 1.096 | 1.094 | 1.136 |
| FBgn0260228 | 0.907 | 1.061 | 1.096 | 1.094 | 1.136 |
| FBgn0067864 | 0.907 | 1.115 | 0.978 | 0.979 | 0.825 |
| FBgn0046876 | 0.906 | 0.859 | 1.115 | 1.394 | 1.569 |
| FBgn0025391 | 0.906 | 1.075 | 0.972 | 1.324 | 1.884 |
| FBgn0029105 | 0.906 | 0.934 | 0.944 | 1.114 | 1.199 |
| FBgn0028699 | 0.906 | 0.891 | 0.951 | 1.233 | 1.357 |
| FBgn0000413 | 0.906 | 1.131 | 1.047 | 1.057 | 0.792 |
| FBgn0039816 | 0.906 | 1.067 | 1.026 | 1.165 | 1.379 |
| FBgn0040972 | 0.905 | 0.829 | 1.270 | 1.317 | 3.444 |
| FBgn0031268 | 0.905 | 1.010 | 1.020 | 1.374 | 1.557 |
| FBgn0035193 | 0.905 | 1.503 | 1.683 | 1.799 | 1.918 |
| FBgn0037680 | 0.905 | 0.899 | 1.090 | 1.094 | 1.914 |
| FBgn0010315 | 0.905 | 1.043 | 1.131 | 1.532 | 1.706 |
| FBgn0038292 | 0.905 | 0.980 | 1.194 | 1.429 | 1.574 |
| FBgn0039761 | 0.905 | 0.863 | 1.062 | 1.261 | 1.393 |
| FBgn0039310 | 0.905 | 0.857 | 1.129 | 1.240 | 1.434 |
| FBgn0032859 | 0.905 | 1.152 | 1.141 | 1.179 | 1.232 |
| FBgn0038180 | 0.905 | 0.809 | 0.922 | 1.168 | 1.277 |
| FBgn0037531 | 0.905 | 1.120 | 1.413 | 1.461 | 2.993 |
| FBgn0003346 | 0.905 | 1.042 | 0.983 | 0.729 | 0.620 |
| FBgn0035964 | 0.905 | 0.981 | 1.164 | 1.208 | 1.355 |
| FBgn0002780 | 0.904 | 0.805 | 0.784 | 1.091 | 1.276 |
| FBgn0052698 | 0.904 | 0.823 | 0.926 | 1.135 | 1.228 |
| FBgn0026263 | 0.904 | 1.033 | 1.003 | 1.359 | 1.580 |
| FBgn0026565 | 0.904 | 1.068 | 1.054 | 0.715 | 0.545 |
| FBgn0033911 | 0.904 | 0.890 | 1.075 | 1.124 | 1.268 |
| FBgn0025185 | 0.904 | 1.034 | 1.156 | 1.267 | 1.346 |
| FBgn0033872 | 0.904 | 1.001 | 0.965 | 1.094 | 1.417 |
| FBgn0033890 | 0.904 | 1.182 | 1.128 | 1.079 | 0.578 |
| FBgn0034804 | 0.904 | 1.073 | 1.223 | 1.229 | 1.382 |
| FBgn0001319 | 0.904 | 0.637 | 0.712 | 0.917 | 2.283 |
| FBgn0031886 | 0.904 | 1.086 | 1.079 | 0.527 | 0.241 |
| FBgn0038436 | 0.904 | 0.835 | 0.692 | 1.186 | 2.305 |
| FBgn0040805 | 0.903 | 0.822 | 0.821 | 0.874 | 1.115 |
| FBgn0033047 | 0.903 | 0.804 | 0.870 | 1.098 | 1.195 |
| FBgn0039197 | 0.903 | 0.956 | 0.807 | 1.391 | 2.978 |
| FBgn0260026 | 0.903 | 1.054 | 0.829 | 0.735 | 0.578 |
| FBgn0032168 | 0.903 | 0.873 | 1.082 | 1.411 | 1.570 |
| FBgn0038274 | 0.903 | 1.038 | 0.885 | 0.872 | 0.729 |
| FBgn0261871 | 0.903 | 0.960 | 0.868 | 1.165 | 1.729 |
| FBgn0035152 | 0.903 | 0.960 | 1.086 | 1.064 | 1.285 |
| FBgn0262117 | 0.903 | 0.971 | 1.000 | 1.219 | 2.411 |
| FBgn0011829 | 0.903 | 1.188 | 1.308 | 1.794 | 4.754 |
| FBgn0010339 | 0.903 | 1.067 | 1.037 | 0.829 | 0.738 |
| FBgn0039598 | 0.903 | 0.909 | 0.878 | 1.008 | 1.134 |
| FBgn0026415 | 0.903 | 0.975 | 1.005 | 1.226 | 1.325 |
| FBgn0032015 | 0.903 | 0.904 | 0.889 | 0.938 | 1.016 |
| FBgn0085399 | 0.903 | 0.808 | 0.729 | 0.991 | 1.362 |
| FBgn0052183 | 0.903 | 1.147 | 1.119 | 1.215 | 1.440 |
| FBgn0038980 | 0.903 | 0.792 | 0.734 | 1.106 | 1.379 |
| FBgn0262719 | 0.903 | 1.179 | 1.089 | 1.387 | 1.802 |
| FBgn0028879 | 0.902 | 0.887 | 0.805 | 1.084 | 1.452 |
| FBgn0002733 | 0.902 | 1.546 | 1.468 | 1.818 | 2.895 |
| FBgn0032036 | 0.902 | 0.945 | 1.024 | 1.262 | 1.362 |
| FBgn0085360 | 0.902 | 0.767 | 1.095 | 1.075 | 1.472 |
| FBgn0261258 | 0.902 | 1.060 | 0.997 | 1.268 | 1.521 |
| FBgn0052179 | 0.902 | 0.869 | 1.040 | 1.070 | 1.214 |
| FBgn0085261 | 0.902 | 0.898 | 1.082 | 1.056 | 1.332 |
| FBgn0039818 | 0.902 | 1.065 | 1.053 | 1.165 | 1.612 |
| FBgn0034627 | 0.902 | 0.834 | 0.840 | 0.871 | 1.055 |
| FBgn0030418 | 0.902 | 1.022 | 0.816 | 1.599 | 2.437 |
| FBgn0031695 | 0.902 | 0.828 | 0.843 | 1.118 | 2.510 |
| FBgn0034808 | 0.902 | 1.083 | 1.037 | 1.214 | 1.401 |
| FBgn0027584 | 0.901 | 1.276 | 1.111 | 1.796 | 3.857 |
| FBgn0030703 | 0.901 | 0.887 | 0.828 | 1.011 | 1.337 |
| FBgn0260642 | 0.901 | 0.990 | 0.936 | 1.138 | 1.701 |
| FBgn0030753 | 0.901 | 0.981 | 0.902 | 0.748 | 0.681 |
| FBgn0036450 | 0.901 | 1.064 | 0.983 | 0.999 | 0.839 |
| FBgn0034530 | 0.900 | 1.050 | 1.041 | 1.150 | 1.617 |
| FBgn0035425 | 0.900 | 1.040 | 1.085 | 0.943 | 0.728 |
| FBgn0027843 | 0.900 | 0.959 | 0.964 | 1.254 | 1.398 |

| Supplemental Table 3C. Values of R Squared and Fold Changes for Genes Found to Behave Linearly at Day 20 Post-Irradiation. (Analysis with all lowest dose discluded.) | | | | | |
| --- | --- | --- | --- | --- | --- |
|  |  |  |  |  |  |
| Flybase ID | R Squared Value Day20 | Day 20 Fold Change 1000R | Day 20 Fold Change 5000R | Day 20 Fold Change 10000R | Day 20 Fold Change 20000R |
| FBgn0051472 | 1.000 | 1.039 | 1.037 | 1.034 | 1.027 |
| FBgn0262177 | 1.000 | 0.052 | 0.077 | 0.110 | 0.178 |
| FBgn0000259 | 1.000 | 1.111 | 1.162 | 1.220 | 1.344 |
| FBgn0263029 | 1.000 | 0.751 | 0.824 | 0.907 | 1.075 |
| FBgn0040715 | 0.999 | 1.172 | 1.267 | 1.410 | 1.682 |
| FBgn0259717 | 0.999 | 1.172 | 1.267 | 1.410 | 1.682 |
| FBgn0033117 | 0.999 | 1.134 | 1.184 | 1.250 | 1.366 |
| FBgn0083972 | 0.999 | 1.506 | 1.740 | 2.077 | 2.644 |
| FBgn0040972 | 0.999 | 1.042 | 2.190 | 3.282 | 5.736 |
| FBgn0014024 | 0.998 | 0.892 | 0.900 | 0.908 | 0.926 |
| FBgn0040837 | 0.998 | 1.559 | 1.942 | 2.409 | 3.207 |
| FBgn0260431 | 0.998 | 1.163 | 1.351 | 1.688 | 2.237 |
| FBgn0031653 | 0.998 | 0.872 | 0.795 | 0.653 | 0.410 |
| FBgn0004797 | 0.998 | 0.933 | 1.025 | 1.195 | 1.473 |
| FBgn0262035 | 0.997 | 1.171 | 1.245 | 1.367 | 1.617 |
| FBgn0033312 | 0.997 | 0.485 | 0.539 | 0.596 | 0.744 |
| FBgn0053178 | 0.997 | 1.255 | 1.414 | 1.605 | 2.080 |
| FBgn0036992 | 0.997 | 0.977 | 0.916 | 0.834 | 0.639 |
| FBgn0042213 | 0.997 | 1.446 | 1.502 | 1.569 | 1.681 |
| FBgn0038295 | 0.997 | 0.840 | 0.870 | 0.929 | 1.021 |
| FBgn0016053 | 0.997 | 0.934 | 1.011 | 1.099 | 1.249 |
| FBgn0036485 | 0.997 | 0.882 | 0.920 | 0.963 | 1.073 |
| FBgn0261871 | 0.997 | 1.063 | 1.118 | 1.223 | 1.382 |
| FBgn0025583 | 0.997 | 1.192 | 1.835 | 2.344 | 3.712 |
| FBgn0046763 | 0.996 | 1.436 | 1.584 | 1.813 | 2.153 |
| FBgn0031418 | 0.996 | 0.986 | 0.943 | 0.898 | 0.817 |
| FBgn0004624 | 0.996 | 1.202 | 1.844 | 2.685 | 4.001 |
| FBgn0086604 | 0.996 | 1.071 | 1.153 | 1.217 | 1.390 |
| FBgn0037850 | 0.996 | 1.411 | 1.769 | 2.408 | 3.722 |
| FBgn0052939 | 0.996 | 1.180 | 1.382 | 1.724 | 2.216 |
| FBgn0036290 | 0.996 | 1.438 | 1.834 | 2.689 | 4.152 |
| FBgn0036681 | 0.996 | 0.282 | 0.371 | 0.516 | 0.725 |
| FBgn0036873 | 0.996 | 1.093 | 0.935 | 0.602 | 0.098 |
| FBgn0031640 | 0.996 | 1.173 | 1.208 | 1.237 | 1.303 |
| FBgn0002578 | 0.995 | 1.237 | 1.156 | 0.973 | 0.681 |
| FBgn0034389 | 0.995 | 1.109 | 1.206 | 1.367 | 1.594 |
| FBgn0039132 | 0.995 | 1.702 | 1.869 | 2.256 | 2.904 |
| FBgn0036487 | 0.995 | 1.093 | 1.153 | 1.294 | 1.514 |
| FBgn0036126 | 0.994 | 1.341 | 2.107 | 2.679 | 4.026 |
| FBgn0035996 | 0.994 | 1.706 | 2.355 | 2.894 | 4.604 |
| FBgn0023514 | 0.993 | 1.207 | 1.274 | 1.341 | 1.457 |
| FBgn0051648 | 0.993 | 0.635 | 0.662 | 0.697 | 0.791 |
| FBgn0030617 | 0.993 | 0.491 | 0.515 | 0.535 | 0.574 |
| FBgn0040056 | 0.993 | 1.478 | 1.632 | 1.820 | 2.101 |
| FBgn0051075 | 0.993 | 0.944 | 0.918 | 0.851 | 0.752 |
| FBgn0002533 | 0.992 | 0.556 | 0.511 | 0.391 | 0.190 |
| FBgn0010038 | 0.992 | 0.766 | 1.195 | 1.706 | 3.165 |
| FBgn0024989 | 0.992 | 0.561 | 0.587 | 0.655 | 0.750 |
| FBgn0031906 | 0.992 | 0.487 | 0.596 | 0.663 | 0.880 |
| FBgn0030189 | 0.992 | 1.168 | 1.303 | 1.424 | 1.817 |
| FBgn0051961 | 0.991 | 1.189 | 1.251 | 1.379 | 1.658 |
| FBgn0040388 | 0.991 | 1.575 | 1.539 | 1.465 | 1.303 |
| FBgn0032943 | 0.991 | 1.459 | 1.697 | 2.054 | 2.521 |
| FBgn0044510 | 0.991 | 1.215 | 1.815 | 2.577 | 4.759 |
| FBgn0033875 | 0.990 | 1.213 | 1.564 | 2.179 | 2.948 |
| FBgn0036667 | 0.990 | 1.170 | 1.275 | 1.568 | 1.958 |
| FBgn0041627 | 0.990 | 1.148 | 1.313 | 1.730 | 2.554 |
| FBgn0035779 | 0.990 | 1.020 | 1.094 | 1.272 | 1.492 |
| FBgn0024740 | 0.990 | 1.124 | 1.150 | 1.171 | 1.211 |
| FBgn0022986 | 0.990 | 0.936 | 0.998 | 1.048 | 1.143 |
| FBgn0086445 | 0.989 | 2.810 | 3.141 | 3.365 | 3.890 |
| FBgn0087039 | 0.989 | 1.304 | 1.819 | 2.216 | 3.010 |
| FBgn0040732 | 0.989 | 0.955 | 1.079 | 1.296 | 1.847 |
| FBgn0032470 | 0.989 | 1.023 | 1.379 | 1.656 | 2.674 |
| FBgn0031697 | 0.988 | 1.294 | 1.531 | 1.656 | 2.089 |
| FBgn0020660 | 0.988 | 1.097 | 1.312 | 1.729 | 2.223 |
| FBgn0037756 | 0.988 | 0.972 | 1.033 | 1.174 | 1.341 |
| FBgn0032505 | 0.988 | 1.136 | 1.172 | 1.270 | 1.466 |
| FBgn0034909 | 0.988 | 0.875 | 0.941 | 0.992 | 1.091 |
| FBgn0262524 | 0.988 | 1.032 | 1.371 | 1.607 | 2.124 |
| FBgn0024728 | 0.988 | 1.449 | 1.618 | 2.017 | 2.485 |
| FBgn0033438 | 0.988 | 0.835 | 0.889 | 0.964 | 1.059 |
| FBgn0086683 | 0.988 | 1.430 | 1.595 | 1.680 | 2.009 |
| FBgn0038473 | 0.987 | 0.969 | 1.012 | 1.074 | 1.251 |
| FBgn0024912 | 0.987 | 1.400 | 1.985 | 2.959 | 4.117 |
| FBgn0029664 | 0.987 | 1.441 | 1.489 | 1.631 | 1.906 |
| FBgn0015351 | 0.987 | 1.171 | 1.332 | 1.589 | 2.294 |
| FBgn0038455 | 0.987 | 1.171 | 1.332 | 1.589 | 2.294 |
| FBgn0010709 | 0.987 | 1.313 | 1.336 | 1.418 | 1.546 |
| FBgn0086676 | 0.987 | 0.680 | 0.710 | 0.811 | 0.943 |
| FBgn0039555 | 0.987 | 0.967 | 0.952 | 0.934 | 0.877 |
| FBgn0035065 | 0.987 | 0.832 | 0.862 | 0.935 | 1.019 |
| FBgn0010350 | 0.987 | 0.787 | 0.764 | 0.694 | 0.611 |
| FBgn0052029 | 0.986 | 1.145 | 1.368 | 1.855 | 2.407 |
| FBgn0026576 | 0.986 | 0.723 | 0.819 | 1.008 | 1.222 |
| FBgn0259143 | 0.986 | 0.832 | 0.885 | 0.928 | 1.005 |
| FBgn0034199 | 0.986 | 1.093 | 1.374 | 1.582 | 1.989 |
| FBgn0033816 | 0.985 | 0.977 | 1.067 | 1.200 | 1.358 |
| FBgn0053007 | 0.985 | 0.964 | 1.038 | 1.221 | 1.426 |
| FBgn0035083 | 0.985 | 1.032 | 1.072 | 1.148 | 1.232 |
| FBgn0032192 | 0.985 | 1.420 | 1.749 | 1.943 | 2.790 |
| FBgn0085435 | 0.985 | 0.308 | 0.330 | 0.416 | 0.566 |
| FBgn0028523 | 0.984 | 2.264 | 2.390 | 2.908 | 3.760 |
| FBgn0030592 | 0.984 | 0.945 | 1.063 | 1.187 | 1.622 |
| FBgn0051272 | 0.984 | 0.973 | 1.005 | 1.127 | 1.278 |
| FBgn0035147 | 0.984 | 0.990 | 0.968 | 0.938 | 0.845 |
| FBgn0003141 | 0.983 | 1.353 | 1.515 | 1.899 | 2.312 |
| FBgn0039544 | 0.983 | 1.191 | 1.243 | 1.365 | 1.497 |
| FBgn0037291 | 0.983 | 1.045 | 0.895 | 0.605 | 0.291 |
| FBgn0035976 | 0.983 | 1.077 | 1.126 | 1.194 | 1.406 |
| FBgn0052640 | 0.983 | 1.621 | 1.936 | 2.795 | 3.720 |
| FBgn0040629 | 0.983 | 0.829 | 0.718 | 0.589 | 0.423 |
| FBgn0052641 | 0.983 | 1.631 | 1.979 | 2.844 | 3.760 |
| FBgn0030260 | 0.983 | 0.481 | 0.508 | 0.585 | 0.757 |
| FBgn0026084 | 0.982 | 1.003 | 1.044 | 1.072 | 1.193 |
| FBgn0036908 | 0.982 | 0.966 | 1.030 | 1.196 | 1.589 |
| FBgn0031065 | 0.982 | 0.621 | 0.662 | 0.749 | 0.840 |
| FBgn0023546 | 0.982 | 0.542 | 0.606 | 0.931 | 1.405 |
| FBgn0260432 | 0.982 | 0.542 | 0.606 | 0.931 | 1.405 |
| FBgn0034184 | 0.982 | 0.948 | 1.018 | 1.052 | 1.222 |
| FBgn0035587 | 0.982 | 1.160 | 1.180 | 1.235 | 1.365 |
| FBgn0037011 | 0.981 | 1.247 | 1.377 | 1.768 | 2.182 |
| FBgn0011509 | 0.981 | 1.179 | 1.230 | 1.497 | 1.865 |
| FBgn0038660 | 0.981 | 0.984 | 1.030 | 1.267 | 1.646 |
| FBgn0032322 | 0.981 | 0.822 | 0.868 | 0.896 | 1.028 |
| FBgn0037372 | 0.981 | 0.532 | 0.546 | 0.618 | 0.726 |
| FBgn0041342 | 0.980 | 0.942 | 0.975 | 0.996 | 1.094 |
| FBgn0039970 | 0.980 | 1.098 | 1.834 | 2.174 | 3.265 |
| FBgn0037061 | 0.980 | 1.855 | 1.933 | 2.137 | 2.346 |
| FBgn0015037 | 0.980 | 2.247 | 2.401 | 3.224 | 4.294 |
| FBgn0259482 | 0.980 | 0.909 | 0.947 | 1.105 | 1.415 |
| FBgn0052121 | 0.980 | 0.981 | 0.858 | 0.770 | 0.607 |
| FBgn0033702 | 0.980 | 0.648 | 0.775 | 0.892 | 1.061 |
| FBgn0033395 | 0.980 | 2.672 | 2.987 | 4.758 | 7.566 |
| FBgn0036232 | 0.979 | 1.609 | 1.905 | 2.160 | 2.552 |
| FBgn0003525 | 0.979 | 0.974 | 1.112 | 1.370 | 1.633 |
| FBgn0031987 | 0.979 | 1.051 | 1.126 | 1.154 | 1.312 |
| FBgn0034512 | 0.979 | 1.267 | 1.347 | 1.709 | 2.406 |
| FBgn0001202 | 0.979 | 1.265 | 1.337 | 1.474 | 1.612 |
| FBgn0025807 | 0.979 | 1.065 | 1.121 | 1.167 | 1.363 |
| FBgn0010278 | 0.978 | 0.886 | 0.917 | 1.016 | 1.244 |
| FBgn0033327 | 0.978 | 0.835 | 0.677 | 0.618 | 0.282 |
| FBgn0028853 | 0.978 | 1.078 | 1.351 | 1.777 | 2.224 |
| FBgn0037465 | 0.978 | 1.030 | 1.064 | 1.080 | 1.127 |
| FBgn0034605 | 0.978 | 1.206 | 1.464 | 1.628 | 1.963 |
| FBgn0024913 | 0.978 | 1.339 | 1.757 | 1.967 | 3.099 |
| FBgn0032597 | 0.978 | 1.165 | 1.344 | 1.714 | 2.078 |
| FBgn0050122 | 0.978 | 0.897 | 0.878 | 0.870 | 0.825 |
| FBgn0052985 | 0.978 | 0.848 | 0.889 | 1.165 | 1.570 |
| FBgn0013772 | 0.977 | 1.753 | 1.867 | 1.905 | 2.128 |
| FBgn0036527 | 0.977 | 1.584 | 1.729 | 1.960 | 2.704 |
| FBgn0039890 | 0.977 | 0.960 | 1.343 | 1.643 | 2.127 |
| FBgn0031143 | 0.976 | 1.083 | 1.141 | 1.225 | 1.313 |
| FBgn0051864 | 0.976 | 1.066 | 1.287 | 2.116 | 2.943 |
| FBgn0023181 | 0.976 | 1.060 | 1.027 | 0.889 | 0.747 |
| FBgn0043806 | 0.976 | 1.000 | 1.049 | 1.353 | 1.704 |
| FBgn0030851 | 0.975 | 1.317 | 1.349 | 1.470 | 1.748 |
| FBgn0025837 | 0.974 | 0.884 | 0.913 | 1.038 | 1.162 |
| FBgn0030659 | 0.974 | 0.915 | 0.904 | 0.849 | 0.734 |
| FBgn0259139 | 0.974 | 1.035 | 1.078 | 1.092 | 1.184 |
| FBgn0052475 | 0.974 | 0.836 | 0.872 | 1.198 | 1.657 |
| FBgn0031159 | 0.974 | 2.799 | 2.818 | 2.988 | 3.216 |
| FBgn0064123 | 0.974 | 2.799 | 2.818 | 2.988 | 3.216 |
| FBgn0054002 | 0.973 | 1.265 | 1.325 | 1.526 | 1.714 |
| FBgn0041233 | 0.973 | 0.540 | 0.556 | 0.689 | 0.859 |
| FBgn0036397 | 0.973 | 0.845 | 0.859 | 0.876 | 0.894 |
| FBgn0013987 | 0.973 | 1.137 | 1.180 | 1.257 | 1.330 |
| FBgn0030038 | 0.973 | 1.010 | 0.957 | 0.892 | 0.822 |
| FBgn0016696 | 0.973 | 1.026 | 1.093 | 1.235 | 1.364 |
| FBgn0039342 | 0.973 | 1.103 | 1.585 | 2.318 | 3.043 |
| FBgn0032701 | 0.973 | 1.528 | 1.556 | 1.711 | 1.868 |
| FBgn0016126 | 0.972 | 1.401 | 1.555 | 2.394 | 3.246 |
| FBgn0037018 | 0.972 | 0.924 | 0.866 | 0.824 | 0.754 |
| FBgn0022774 | 0.972 | 1.040 | 0.933 | 0.895 | 0.744 |
| FBgn0020270 | 0.971 | 1.133 | 1.155 | 1.404 | 1.773 |
| FBgn0031805 | 0.971 | 1.160 | 1.246 | 1.276 | 1.397 |
| FBgn0039601 | 0.971 | 1.104 | 1.166 | 1.187 | 1.275 |
| FBgn0035440 | 0.971 | 1.225 | 1.523 | 1.682 | 2.045 |
| FBgn0023000 | 0.971 | 1.167 | 1.204 | 1.648 | 2.278 |
| FBgn0003423 | 0.971 | 0.948 | 0.871 | 0.804 | 0.713 |
| FBgn0069973 | 0.970 | 1.124 | 1.135 | 1.283 | 1.494 |
| FBgn0039942 | 0.970 | 0.613 | 0.733 | 0.838 | 0.979 |
| FBgn0039298 | 0.970 | 1.611 | 1.835 | 1.987 | 2.249 |
| FBgn0038846 | 0.969 | 1.309 | 1.422 | 1.523 | 1.656 |
| FBgn0058045 | 0.969 | 1.516 | 1.849 | 2.347 | 2.823 |
| FBgn0035166 | 0.969 | 0.745 | 0.892 | 0.939 | 1.297 |
| FBgn0035887 | 0.969 | 1.621 | 1.568 | 0.958 | 0.234 |
| FBgn0028394 | 0.969 | 1.317 | 1.445 | 1.478 | 1.682 |
| FBgn0050154 | 0.969 | 1.142 | 1.261 | 1.305 | 1.460 |
| FBgn0037622 | 0.968 | 0.929 | 0.945 | 1.055 | 1.165 |
| FBgn0033826 | 0.968 | 0.966 | 0.962 | 0.914 | 0.826 |
| FBgn0035343 | 0.968 | 0.842 | 0.958 | 0.991 | 1.267 |
| FBgn0037719 | 0.968 | 1.205 | 1.262 | 1.378 | 1.764 |
| FBgn0027081 | 0.967 | 0.949 | 0.975 | 1.018 | 1.056 |
| FBgn0037309 | 0.967 | 1.414 | 1.440 | 1.862 | 2.557 |
| FBgn0036889 | 0.967 | 1.294 | 1.344 | 1.463 | 1.562 |
| FBgn0014143 | 0.967 | 1.163 | 1.056 | 1.029 | 0.792 |
| FBgn0037322 | 0.967 | 0.198 | 0.212 | 0.344 | 0.605 |
| FBgn0038318 | 0.966 | 0.755 | 0.786 | 0.826 | 0.866 |
| FBgn0038098 | 0.966 | 1.193 | 1.050 | 1.000 | 0.603 |
| FBgn0034380 | 0.965 | 1.390 | 1.387 | 1.354 | 1.318 |
| FBgn0037279 | 0.965 | 1.065 | 1.066 | 1.088 | 1.123 |
| FBgn0033422 | 0.965 | 1.069 | 1.063 | 0.906 | 0.706 |
| FBgn0038548 | 0.965 | 0.914 | 0.953 | 1.059 | 1.385 |
| FBgn0261429 | 0.964 | 1.048 | 1.079 | 1.849 | 2.800 |
| FBgn0052483 | 0.964 | 0.933 | 1.152 | 1.203 | 1.701 |
| FBgn0039584 | 0.964 | 1.044 | 1.050 | 1.163 | 1.365 |
| FBgn0030716 | 0.964 | 1.058 | 1.181 | 1.304 | 1.444 |
| FBgn0036147 | 0.964 | 1.686 | 1.693 | 1.956 | 2.339 |
| FBgn0025808 | 0.964 | 1.306 | 1.212 | 1.171 | 0.872 |
| FBgn0037943 | 0.963 | 1.089 | 1.119 | 1.145 | 1.277 |
| FBgn0039805 | 0.963 | 1.845 | 1.927 | 3.320 | 4.828 |
| FBgn0031307 | 0.963 | 1.214 | 1.543 | 2.492 | 3.248 |
| FBgn0043576 | 0.963 | 0.804 | 0.666 | 0.626 | 0.257 |
| FBgn0033261 | 0.963 | 1.306 | 1.320 | 1.945 | 2.753 |
| FBgn0029962 | 0.963 | 1.054 | 1.090 | 1.487 | 1.877 |
| FBgn0039260 | 0.963 | 0.914 | 0.954 | 0.969 | 1.016 |
| FBgn0004101 | 0.962 | 1.346 | 1.348 | 1.459 | 1.631 |
| FBgn0035228 | 0.962 | 0.948 | 0.923 | 0.918 | 0.866 |
| FBgn0033134 | 0.962 | 1.832 | 2.253 | 3.542 | 4.562 |
| FBgn0029663 | 0.962 | 1.119 | 1.270 | 1.359 | 1.522 |
| FBgn0032719 | 0.962 | 0.869 | 0.887 | 1.025 | 1.151 |
| FBgn0030925 | 0.962 | 0.951 | 1.020 | 1.032 | 1.167 |
| FBgn0026570 | 0.962 | 1.202 | 1.265 | 1.692 | 2.069 |
| FBgn0004580 | 0.962 | 1.104 | 1.162 | 1.234 | 1.553 |
| FBgn0051915 | 0.962 | 0.975 | 0.985 | 1.300 | 1.661 |
| FBgn0261387 | 0.961 | 0.971 | 1.238 | 1.591 | 1.917 |
| FBgn0032879 | 0.961 | 1.237 | 1.253 | 1.801 | 2.430 |
| FBgn0261613 | 0.961 | 1.714 | 1.841 | 2.438 | 4.048 |
| FBgn0259236 | 0.961 | 0.780 | 0.807 | 0.908 | 0.988 |
| FBgn0037764 | 0.961 | 1.809 | 1.819 | 2.325 | 3.184 |
| FBgn0026263 | 0.961 | 0.808 | 0.849 | 1.168 | 1.454 |
| FBgn0035462 | 0.961 | 1.530 | 1.488 | 1.473 | 1.344 |
| FBgn0035998 | 0.961 | 1.217 | 1.521 | 1.923 | 2.292 |
| FBgn0034647 | 0.961 | 1.497 | 1.411 | 1.279 | 0.739 |
| FBgn0033928 | 0.961 | 1.694 | 1.700 | 2.473 | 3.460 |
| FBgn0039396 | 0.960 | 4.122 | 4.425 | 4.982 | 7.099 |
| FBgn0033130 | 0.960 | 1.304 | 1.352 | 1.796 | 2.199 |
| FBgn0038160 | 0.960 | 2.556 | 2.109 | 1.813 | 1.342 |
| FBgn0034726 | 0.960 | 0.967 | 1.211 | 1.327 | 1.594 |
| FBgn0003317 | 0.960 | 1.149 | 1.128 | 1.067 | 1.020 |
| FBgn0039163 | 0.960 | 1.252 | 1.304 | 1.316 | 1.389 |
| FBgn0011774 | 0.960 | 1.096 | 1.113 | 1.871 | 2.722 |
| FBgn0019948 | 0.960 | 1.132 | 1.182 | 1.351 | 1.482 |
| FBgn0037535 | 0.960 | 1.263 | 1.360 | 1.393 | 1.685 |
| FBgn0038880 | 0.960 | 1.876 | 1.919 | 3.213 | 4.606 |
| FBgn0030029 | 0.959 | 1.040 | 1.216 | 1.306 | 1.494 |
| FBgn0032819 | 0.959 | 1.084 | 1.310 | 1.353 | 1.679 |
| FBgn0034523 | 0.958 | 1.221 | 1.218 | 0.959 | 0.669 |
| FBgn0037937 | 0.958 | 2.778 | 2.767 | 3.838 | 5.568 |
| FBgn0031710 | 0.958 | 1.211 | 1.238 | 1.327 | 1.611 |
| FBgn0033648 | 0.958 | 0.000 | 0.013 | 0.019 | 0.066 |
| FBgn0029737 | 0.958 | 0.933 | 0.924 | 0.833 | 0.753 |
| FBgn0032949 | 0.958 | 1.302 | 1.409 | 1.979 | 2.432 |
| FBgn0032472 | 0.958 | 0.983 | 1.432 | 1.883 | 2.360 |
| FBgn0036340 | 0.957 | 0.920 | 0.913 | 0.870 | 0.836 |
| FBgn0039328 | 0.957 | 0.550 | 0.566 | 0.756 | 0.926 |
| FBgn0026749 | 0.957 | 1.096 | 1.153 | 1.264 | 1.349 |
| FBgn0038236 | 0.957 | 1.573 | 1.734 | 2.262 | 2.655 |
| FBgn0000316 | 0.957 | 1.052 | 1.050 | 1.192 | 1.383 |
| FBgn0035816 | 0.957 | 0.783 | 0.787 | 0.856 | 1.008 |
| FBgn0033153 | 0.957 | 1.249 | 1.641 | 1.785 | 2.227 |
| FBgn0032731 | 0.957 | 0.935 | 1.001 | 1.097 | 1.177 |
| FBgn0036872 | 0.956 | 2.079 | 1.558 | 1.310 | 0.762 |
| FBgn0087002 | 0.956 | 1.180 | 1.842 | 3.254 | 4.312 |
| FBgn0033836 | 0.956 | 1.059 | 1.070 | 1.296 | 1.789 |
| FBgn0040754 | 0.956 | 1.005 | 1.044 | 1.172 | 1.266 |
| FBgn0259722 | 0.956 | 1.052 | 1.049 | 1.192 | 1.381 |
| FBgn0032032 | 0.956 | 1.132 | 1.216 | 1.267 | 1.615 |
| FBgn0039099 | 0.956 | 1.090 | 1.236 | 1.355 | 1.502 |
| FBgn0032283 | 0.956 | 1.380 | 1.388 | 1.109 | 0.673 |
| FBgn0053109 | 0.955 | 1.104 | 1.265 | 1.508 | 1.707 |
| FBgn0039481 | 0.955 | 0.688 | 0.818 | 0.923 | 1.054 |
| FBgn0052676 | 0.955 | 0.963 | 0.892 | 0.831 | 0.759 |
| FBgn0083228 | 0.955 | 0.811 | 0.828 | 1.035 | 1.212 |
| FBgn0037731 | 0.955 | 1.164 | 1.338 | 1.787 | 2.110 |
| FBgn0052220 | 0.955 | 1.045 | 1.248 | 1.448 | 1.656 |
| FBgn0038953 | 0.954 | 1.305 | 1.347 | 1.476 | 1.912 |
| FBgn0053288 | 0.954 | 1.480 | 1.543 | 1.846 | 2.072 |
| FBgn0032946 | 0.954 | 1.321 | 1.522 | 1.546 | 1.974 |
| FBgn0039670 | 0.954 | 0.928 | 1.205 | 1.273 | 1.613 |
| FBgn0037020 | 0.954 | 0.996 | 0.993 | 1.100 | 1.229 |
| FBgn0044048 | 0.954 | 0.596 | 0.649 | 0.793 | 1.308 |
| FBgn0027914 | 0.954 | 1.327 | 1.159 | 1.101 | 0.915 |
| FBgn0025825 | 0.954 | 1.062 | 1.064 | 1.112 | 1.219 |
| FBgn0037518 | 0.954 | 1.181 | 1.190 | 1.322 | 1.641 |
| FBgn0039807 | 0.953 | 1.549 | 1.802 | 1.859 | 2.176 |
| FBgn0037481 | 0.953 | 2.300 | 2.342 | 2.485 | 2.588 |
| FBgn0033453 | 0.953 | 1.092 | 1.799 | 2.498 | 3.213 |
| FBgn0030694 | 0.953 | 1.765 | 1.759 | 2.148 | 2.902 |
| FBgn0029913 | 0.953 | 1.689 | 1.713 | 1.799 | 2.087 |
| FBgn0050026 | 0.953 | 1.169 | 1.259 | 1.553 | 2.557 |
| FBgn0022936 | 0.952 | 1.146 | 1.452 | 1.527 | 1.895 |
| FBgn0037100 | 0.952 | 0.766 | 0.885 | 1.308 | 1.608 |
| FBgn0010240 | 0.952 | 0.849 | 0.949 | 0.971 | 1.260 |
| FBgn0034415 | 0.952 | 1.158 | 1.178 | 1.203 | 1.326 |
| FBgn0000008 | 0.952 | 0.871 | 0.837 | 0.828 | 0.723 |
| FBgn0040099 | 0.952 | 2.137 | 2.472 | 3.254 | 3.804 |
| FBgn0020416 | 0.952 | 1.304 | 1.290 | 1.732 | 2.223 |
| FBgn0032781 | 0.951 | 1.019 | 1.244 | 1.329 | 1.566 |
| FBgn0016920 | 0.951 | 0.603 | 0.595 | 0.797 | 1.033 |
| FBgn0019960 | 0.951 | 0.887 | 0.821 | 0.720 | 0.641 |
| FBgn0022959 | 0.951 | 0.909 | 0.880 | 0.761 | 0.677 |
| FBgn0037906 | 0.951 | 1.156 | 1.156 | 1.054 | 0.836 |
| FBgn0263093 | 0.951 | 3.478 | 3.536 | 6.177 | 8.585 |
| FBgn0053653 | 0.951 | 1.513 | 1.810 | 2.349 | 2.742 |
| FBgn0032810 | 0.951 | 0.711 | 0.708 | 0.754 | 0.817 |
| FBgn0035049 | 0.950 | 1.185 | 1.206 | 1.332 | 1.425 |
| FBgn0016698 | 0.950 | 0.915 | 0.942 | 0.944 | 0.990 |
| FBgn0085285 | 0.950 | 1.074 | 1.148 | 1.769 | 2.246 |
| FBgn0031216 | 0.950 | 1.368 | 1.355 | 1.968 | 2.586 |
| FBgn0028938 | 0.950 | 1.358 | 1.307 | 2.020 | 3.024 |
| FBgn0050281 | 0.950 | 1.190 | 1.415 | 1.633 | 1.853 |
| FBgn0051104 | 0.950 | 0.976 | 0.997 | 1.416 | 1.770 |
| FBgn0032198 | 0.949 | 1.103 | 1.124 | 1.222 | 1.291 |
| FBgn0032669 | 0.949 | 1.916 | 2.019 | 2.718 | 3.233 |
| FBgn0036017 | 0.949 | 1.277 | 1.375 | 1.605 | 1.762 |
| FBgn0039696 | 0.949 | 0.856 | 0.938 | 1.124 | 1.251 |
| FBgn0024987 | 0.949 | 1.010 | 1.061 | 1.141 | 1.201 |
| FBgn0035189 | 0.949 | 0.863 | 0.834 | 0.734 | 0.666 |
| FBgn0015527 | 0.949 | 0.890 | 0.901 | 0.959 | 1.000 |
| FBgn0063497 | 0.949 | 1.421 | 1.420 | 1.690 | 1.945 |
| FBgn0259821 | 0.948 | 1.484 | 1.469 | 1.682 | 2.049 |
| FBgn0051036 | 0.948 | 0.921 | 0.957 | 1.452 | 1.848 |
| FBgn0052000 | 0.948 | 1.575 | 1.782 | 2.853 | 3.597 |
| FBgn0039616 | 0.948 | 1.071 | 1.186 | 1.260 | 1.799 |
| FBgn0028691 | 0.948 | 1.066 | 1.016 | 0.959 | 0.908 |
| FBgn0029912 | 0.948 | 0.984 | 0.988 | 0.938 | 0.875 |
| FBgn0039405 | 0.947 | 0.970 | 1.097 | 1.118 | 1.279 |
| FBgn0260224 | 0.947 | 0.970 | 1.097 | 1.118 | 1.279 |
| FBgn0039153 | 0.947 | 1.556 | 1.554 | 1.916 | 2.247 |
| FBgn0037305 | 0.947 | 1.040 | 1.110 | 1.115 | 1.238 |
| FBgn0262442 | 0.947 | 1.345 | 1.110 | 0.670 | 0.362 |
| FBgn0032393 | 0.947 | 1.020 | 1.398 | 2.248 | 2.817 |
| FBgn0033988 | 0.947 | 0.934 | 0.935 | 1.013 | 1.082 |
| FBgn0085768 | 0.946 | 1.165 | 1.405 | 1.703 | 1.947 |
| FBgn0031361 | 0.946 | 2.656 | 2.746 | 3.385 | 3.840 |
| FBgn0037141 | 0.946 | 1.490 | 1.440 | 1.987 | 2.818 |
| FBgn0011764 | 0.946 | 0.943 | 1.041 | 1.113 | 1.202 |
| FBgn0022800 | 0.946 | 1.182 | 1.167 | 1.331 | 1.566 |
| FBgn0053301 | 0.946 | 1.310 | 1.309 | 1.664 | 1.982 |
| FBgn0040001 | 0.946 | 1.069 | 1.430 | 2.226 | 2.755 |
| FBgn0033206 | 0.945 | 0.970 | 1.005 | 1.106 | 1.171 |
| FBgn0037697 | 0.945 | 0.990 | 0.862 | 0.853 | 0.655 |
| FBgn0031881 | 0.945 | 0.814 | 0.843 | 0.895 | 1.132 |
| FBgn0003495 | 0.945 | 0.925 | 0.914 | 1.135 | 1.602 |
| FBgn0000625 | 0.945 | 0.928 | 0.990 | 1.116 | 1.200 |
| FBgn0015582 | 0.945 | 1.199 | 1.211 | 1.440 | 1.620 |
| FBgn0038454 | 0.945 | 0.880 | 0.956 | 0.961 | 1.073 |
| FBgn0034182 | 0.945 | 1.401 | 1.555 | 1.997 | 2.282 |
| FBgn0027930 | 0.944 | 0.955 | 0.951 | 1.090 | 1.219 |
| FBgn0031473 | 0.944 | 1.351 | 1.374 | 1.619 | 1.799 |
| FBgn0052564 | 0.944 | 0.825 | 0.733 | 0.725 | 0.494 |
| FBgn0030795 | 0.944 | 1.159 | 1.147 | 1.482 | 1.798 |
| FBgn0030266 | 0.944 | 0.802 | 0.745 | 0.691 | 0.638 |
| FBgn0003356 | 0.943 | 0.967 | 0.963 | 0.828 | 0.721 |
| FBgn0001234 | 0.943 | 0.840 | 0.887 | 1.200 | 1.412 |
| FBgn0015524 | 0.943 | 2.620 | 2.953 | 3.117 | 3.423 |
| FBgn0013305 | 0.942 | 0.829 | 0.965 | 1.078 | 1.199 |
| FBgn0038472 | 0.942 | 0.788 | 0.773 | 0.906 | 1.084 |
| FBgn0259233 | 0.942 | 1.916 | 1.909 | 2.863 | 3.667 |
| FBgn0039993 | 0.942 | 1.144 | 1.310 | 1.722 | 1.980 |
| FBgn0028411 | 0.941 | 2.019 | 2.891 | 3.009 | 4.087 |
| FBgn0024362 | 0.941 | 0.754 | 0.752 | 0.766 | 0.791 |
| FBgn0036024 | 0.941 | 0.986 | 0.818 | 0.794 | 0.589 |
| FBgn0028518 | 0.941 | 1.345 | 1.345 | 1.422 | 1.484 |
| FBgn0023477 | 0.941 | 0.880 | 0.878 | 0.845 | 0.747 |
| FBgn0028373 | 0.941 | 0.860 | 0.860 | 0.966 | 1.053 |
| FBgn0031229 | 0.941 | 1.242 | 1.321 | 1.480 | 1.582 |
| FBgn0025879 | 0.940 | 1.100 | 1.179 | 1.534 | 1.757 |
| FBgn0052196 | 0.940 | 4.141 | 3.995 | 5.277 | 6.853 |
| FBgn0032859 | 0.940 | 1.221 | 1.291 | 1.315 | 1.594 |
| FBgn0040010 | 0.940 | 1.212 | 1.195 | 1.344 | 1.604 |
| FBgn0035767 | 0.940 | 0.944 | 0.946 | 1.059 | 1.146 |
| FBgn0031973 | 0.940 | 0.955 | 1.075 | 1.192 | 1.299 |
| FBgn0032034 | 0.940 | 2.641 | 2.671 | 3.171 | 3.532 |
| FBgn0036023 | 0.940 | 1.146 | 0.879 | 0.750 | 0.509 |
| FBgn0085408 | 0.939 | 0.965 | 0.968 | 1.004 | 1.030 |
| FBgn0039647 | 0.939 | 0.986 | 0.977 | 1.194 | 1.384 |
| FBgn0039777 | 0.939 | 1.069 | 1.088 | 0.942 | 0.754 |
| FBgn0052082 | 0.939 | 1.266 | 1.292 | 1.570 | 1.760 |
| FBgn0052203 | 0.939 | 1.254 | 1.634 | 1.779 | 2.135 |
| FBgn0010315 | 0.939 | 1.145 | 1.205 | 1.298 | 1.361 |
| FBgn0028546 | 0.938 | 1.310 | 1.397 | 1.858 | 2.146 |
| FBgn0032167 | 0.938 | 1.065 | 1.041 | 1.344 | 1.637 |
| FBgn0032613 | 0.938 | 1.413 | 1.416 | 2.174 | 2.762 |
| FBgn0035452 | 0.938 | 1.710 | 2.162 | 3.029 | 3.575 |
| FBgn0038451 | 0.938 | 0.535 | 0.704 | 0.920 | 1.081 |
| FBgn0053230 | 0.938 | 1.023 | 1.008 | 1.114 | 1.274 |
| FBgn0011591 | 0.938 | 0.871 | 1.046 | 1.338 | 1.530 |
| FBgn0034530 | 0.937 | 0.757 | 0.823 | 1.192 | 1.421 |
| FBgn0031910 | 0.937 | 0.726 | 0.800 | 0.894 | 0.964 |
| FBgn0262740 | 0.937 | 0.925 | 0.885 | 0.768 | 0.699 |
| FBgn0033744 | 0.937 | 1.013 | 0.998 | 1.116 | 1.340 |
| FBgn0016041 | 0.937 | 0.973 | 0.923 | 0.764 | 0.670 |
| FBgn0035374 | 0.937 | 0.890 | 0.897 | 0.841 | 0.777 |
| FBgn0259222 | 0.936 | 0.973 | 1.170 | 1.199 | 1.425 |
| FBgn0051781 | 0.936 | 1.017 | 1.273 | 1.385 | 1.614 |
| FBgn0030314 | 0.936 | 0.802 | 0.769 | 0.768 | 0.717 |
| FBgn0033927 | 0.936 | 1.156 | 1.082 | 1.636 | 2.703 |
| FBgn0034454 | 0.935 | 1.161 | 1.573 | 1.585 | 2.178 |
| FBgn0010651 | 0.935 | 0.798 | 0.858 | 1.069 | 1.192 |
| FBgn0051778 | 0.935 | 1.299 | 1.486 | 2.368 | 2.893 |
| FBgn0037249 | 0.935 | 0.906 | 0.911 | 0.874 | 0.803 |
| FBgn0038439 | 0.935 | 0.840 | 0.733 | 0.634 | 0.544 |
| FBgn0030969 | 0.935 | 1.038 | 0.955 | 0.895 | 0.826 |
| FBgn0051769 | 0.934 | 1.228 | 1.276 | 1.293 | 1.504 |
| FBgn0015781 | 0.934 | 1.119 | 1.094 | 1.093 | 1.022 |
| FBgn0029708 | 0.934 | 0.922 | 0.960 | 1.161 | 1.281 |
| FBgn0033543 | 0.934 | 0.933 | 0.929 | 1.088 | 1.212 |
| FBgn0038533 | 0.934 | 1.035 | 1.087 | 1.091 | 1.243 |
| FBgn0035656 | 0.934 | 1.017 | 1.114 | 1.116 | 1.254 |
| FBgn0262057 | 0.934 | 1.112 | 1.109 | 1.265 | 1.385 |
| FBgn0038082 | 0.934 | 1.273 | 1.377 | 2.556 | 3.322 |
| FBgn0261989 | 0.934 | 1.963 | 2.859 | 5.799 | 7.485 |
| FBgn0034627 | 0.934 | 0.637 | 0.699 | 1.066 | 1.287 |
| FBgn0051516 | 0.934 | 0.980 | 1.020 | 1.020 | 1.112 |
| FBgn0086368 | 0.934 | 1.082 | 1.047 | 1.265 | 1.628 |
| FBgn0052099 | 0.934 | 1.358 | 1.336 | 1.559 | 1.770 |
| FBgn0261588 | 0.933 | 1.092 | 1.016 | 0.898 | 0.822 |
| FBgn0041605 | 0.933 | 0.844 | 0.892 | 0.947 | 0.989 |
| FBgn0033668 | 0.933 | 0.802 | 1.056 | 1.865 | 2.324 |
| FBgn0032785 | 0.933 | 1.349 | 1.611 | 1.792 | 2.006 |
| FBgn0034029 | 0.933 | 1.188 | 1.234 | 1.842 | 2.237 |
| FBgn0038795 | 0.933 | 1.305 | 1.502 | 2.317 | 2.784 |
| FBgn0038893 | 0.932 | 1.453 | 1.398 | 2.536 | 3.456 |
| FBgn0022069 | 0.932 | 1.130 | 1.122 | 0.988 | 0.900 |
| FBgn0051719 | 0.932 | 0.956 | 1.046 | 1.211 | 1.310 |
| FBgn0010053 | 0.932 | 1.239 | 1.264 | 1.532 | 1.701 |
| FBgn0031001 | 0.932 | 0.792 | 0.625 | 0.578 | 0.420 |
| FBgn0033919 | 0.932 | 1.094 | 1.136 | 1.210 | 1.255 |
| FBgn0063494 | 0.931 | 1.382 | 1.376 | 1.879 | 2.249 |
| FBgn0034958 | 0.931 | 0.989 | 0.995 | 1.156 | 1.263 |
| FBgn0260946 | 0.931 | 0.954 | 1.032 | 1.067 | 1.133 |
| FBgn0029997 | 0.931 | 0.870 | 0.873 | 0.736 | 0.632 |
| FBgn0037603 | 0.931 | 1.325 | 1.459 | 1.559 | 1.667 |
| FBgn0032150 | 0.931 | 0.869 | 0.842 | 0.810 | 0.786 |
| FBgn0261089 | 0.931 | 0.623 | 0.661 | 0.984 | 1.180 |
| FBgn0000079 | 0.930 | 0.959 | 0.875 | 0.846 | 0.771 |
| FBgn0025366 | 0.930 | 1.068 | 1.106 | 1.269 | 1.361 |
| FBgn0034656 | 0.930 | 1.116 | 1.161 | 1.369 | 1.487 |
| FBgn0038304 | 0.930 | 1.213 | 1.102 | 0.943 | 0.840 |
| FBgn0033458 | 0.930 | 1.099 | 1.058 | 1.356 | 1.660 |
| FBgn0037227 | 0.929 | 0.737 | 0.570 | 0.378 | 0.236 |
| FBgn0030056 | 0.929 | 0.862 | 0.870 | 0.938 | 0.980 |
| FBgn0034628 | 0.929 | 0.995 | 1.008 | 1.318 | 1.520 |
| FBgn0250791 | 0.929 | 1.030 | 1.032 | 1.073 | 1.100 |
| FBgn0031461 | 0.929 | 0.721 | 0.811 | 0.990 | 1.092 |
| FBgn0036536 | 0.929 | 0.808 | 0.672 | 0.436 | 0.296 |
| FBgn0033017 | 0.928 | 1.379 | 1.500 | 1.757 | 1.901 |
| FBgn0031114 | 0.928 | 0.976 | 1.013 | 1.278 | 1.431 |
| FBgn0032801 | 0.928 | 1.001 | 1.083 | 1.220 | 1.302 |
| FBgn0037076 | 0.928 | 1.320 | 1.791 | 1.788 | 3.083 |
| FBgn0037203 | 0.927 | 1.002 | 0.991 | 1.143 | 1.266 |
| FBgn0025697 | 0.927 | 1.316 | 1.356 | 2.000 | 2.401 |
| FBgn0025741 | 0.927 | 0.855 | 1.623 | 2.282 | 2.884 |
| FBgn0033812 | 0.927 | 0.923 | 0.969 | 0.993 | 1.029 |
| FBgn0260748 | 0.927 | 0.962 | 1.015 | 1.069 | 1.111 |
| FBgn0260795 | 0.926 | 1.252 | 1.208 | 1.697 | 2.100 |
| FBgn0028956 | 0.926 | 1.113 | 1.090 | 1.205 | 1.409 |
| FBgn0010173 | 0.926 | 1.309 | 1.238 | 1.770 | 2.273 |
| FBgn0033876 | 0.926 | 0.839 | 0.954 | 0.986 | 1.091 |
| FBgn0032919 | 0.926 | 1.209 | 1.192 | 1.294 | 1.523 |
| FBgn0085442 | 0.926 | 1.105 | 1.128 | 1.320 | 1.430 |
| FBgn0035153 | 0.926 | 0.952 | 0.988 | 1.113 | 1.178 |
| FBgn0035886 | 0.926 | 1.797 | 1.932 | 1.270 | 0.216 |
| FBgn0035165 | 0.925 | 0.974 | 1.099 | 1.115 | 1.249 |
| FBgn0037818 | 0.925 | 1.481 | 1.463 | 2.116 | 2.573 |
| FBgn0031693 | 0.925 | 1.237 | 1.309 | 1.934 | 2.292 |
| FBgn0010333 | 0.925 | 0.894 | 0.910 | 0.922 | 0.935 |
| FBgn0040211 | 0.925 | 0.872 | 0.834 | 0.823 | 0.649 |
| FBgn0014022 | 0.925 | 0.551 | 0.635 | 0.631 | 0.762 |
| FBgn0262539 | 0.925 | 1.714 | 2.163 | 2.730 | 3.107 |
| FBgn0026787 | 0.925 | 1.183 | 1.176 | 1.371 | 1.509 |
| FBgn0037007 | 0.925 | 1.078 | 1.058 | 1.167 | 1.399 |
| FBgn0036921 | 0.925 | 0.904 | 0.899 | 0.965 | 1.015 |
| FBgn0037105 | 0.924 | 1.351 | 1.295 | 1.849 | 2.309 |
| FBgn0031604 | 0.924 | 1.007 | 0.992 | 1.077 | 1.171 |
| FBgn0004057 | 0.924 | 0.870 | 0.869 | 0.857 | 0.809 |
| FBgn0039421 | 0.924 | 1.543 | 1.174 | 0.514 | 0.144 |
| FBgn0053158 | 0.924 | 1.108 | 1.056 | 1.306 | 1.746 |
| FBgn0036768 | 0.924 | 0.879 | 0.873 | 0.910 | 0.998 |
| FBgn0038676 | 0.924 | 0.187 | 0.249 | 1.302 | 1.934 |
| FBgn0261041 | 0.923 | 1.049 | 1.049 | 1.573 | 1.917 |
| FBgn0012344 | 0.923 | 0.812 | 0.823 | 0.730 | 0.647 |
| FBgn0030157 | 0.923 | 1.357 | 1.449 | 1.863 | 2.080 |
| FBgn0033188 | 0.923 | 0.753 | 0.865 | 1.018 | 1.114 |
| FBgn0037794 | 0.923 | 1.111 | 1.097 | 1.674 | 2.066 |
| FBgn0033915 | 0.923 | 0.940 | 0.989 | 1.085 | 1.137 |
| FBgn0034265 | 0.922 | 1.195 | 1.167 | 1.327 | 1.496 |
| FBgn0067779 | 0.922 | 1.751 | 1.637 | 2.156 | 2.948 |
| FBgn0003742 | 0.922 | 1.529 | 1.535 | 1.382 | 1.275 |
| FBgn0020303 | 0.922 | 0.977 | 0.950 | 1.081 | 1.248 |
| FBgn0014396 | 0.922 | 1.202 | 1.330 | 1.635 | 1.793 |
| FBgn0003687 | 0.922 | 0.862 | 0.957 | 1.010 | 1.082 |
| FBgn0003071 | 0.922 | 0.961 | 0.976 | 0.787 | 0.647 |
| FBgn0016919 | 0.922 | 1.460 | 1.391 | 1.892 | 2.339 |
| FBgn0034312 | 0.922 | 1.357 | 1.582 | 1.564 | 1.938 |
| FBgn0050158 | 0.922 | 1.147 | 1.247 | 1.513 | 1.648 |
| FBgn0013303 | 0.921 | 0.705 | 1.169 | 1.479 | 1.825 |
| FBgn0039052 | 0.921 | 1.181 | 1.410 | 2.469 | 3.014 |
| FBgn0033926 | 0.921 | 1.163 | 1.042 | 1.610 | 2.730 |
| FBgn0039056 | 0.921 | 1.242 | 1.232 | 1.411 | 1.537 |
| FBgn0063493 | 0.921 | 1.288 | 1.347 | 1.623 | 1.764 |
| FBgn0020647 | 0.921 | 0.860 | 0.877 | 0.779 | 0.682 |
| FBgn0054012 | 0.921 | 1.122 | 0.965 | 0.411 | 0.133 |
| FBgn0004666 | 0.921 | 1.112 | 1.065 | 1.274 | 1.567 |
| FBgn0037217 | 0.921 | 1.850 | 1.746 | 2.202 | 2.905 |
| FBgn0030647 | 0.920 | 1.138 | 1.017 | 0.358 | 0.018 |
| FBgn0028879 | 0.920 | 1.049 | 1.246 | 1.267 | 1.473 |
| FBgn0260475 | 0.920 | 1.034 | 1.000 | 1.214 | 1.768 |
| FBgn0013263 | 0.920 | 0.966 | 0.964 | 0.895 | 0.853 |
| FBgn0037936 | 0.920 | 1.283 | 1.632 | 2.307 | 2.663 |
| FBgn0039958 | 0.920 | 1.161 | 1.097 | 1.609 | 2.035 |
| FBgn0038917 | 0.920 | 1.138 | 1.181 | 1.348 | 1.432 |
| FBgn0033755 | 0.919 | 1.225 | 1.193 | 1.646 | 1.970 |
| FBgn0003326 | 0.919 | 0.832 | 0.858 | 0.881 | 1.077 |
| FBgn0015618 | 0.919 | 0.888 | 1.080 | 1.144 | 1.302 |
| FBgn0039531 | 0.918 | 1.000 | 1.030 | 1.026 | 1.082 |
| FBgn0263132 | 0.918 | 0.724 | 0.649 | 1.070 | 1.480 |
| FBgn0014859 | 0.918 | 0.866 | 0.895 | 1.352 | 1.609 |
| FBgn0040697 | 0.918 | 0.804 | 0.930 | 1.012 | 1.103 |
| FBgn0035089 | 0.918 | 1.802 | 2.613 | 2.878 | 3.542 |
| FBgn0004177 | 0.918 | 1.000 | 1.033 | 1.105 | 1.142 |
| FBgn0035805 | 0.917 | 1.217 | 1.530 | 2.090 | 2.386 |
| FBgn0026056 | 0.917 | 1.258 | 1.337 | 1.357 | 1.426 |
| FBgn0053281 | 0.917 | 0.874 | 0.939 | 1.223 | 1.362 |
| FBgn0051265 | 0.917 | 0.576 | 0.718 | 0.722 | 0.885 |
| FBgn0029113 | 0.917 | 1.155 | 1.147 | 1.183 | 1.262 |
| FBgn0039597 | 0.916 | 1.029 | 1.246 | 1.228 | 1.561 |
| FBgn0010348 | 0.916 | 1.132 | 1.179 | 1.417 | 1.534 |
| FBgn0033033 | 0.916 | 0.891 | 0.841 | 1.212 | 1.516 |
| FBgn0085805 | 0.916 | 0.260 | 0.322 | 0.815 | 1.068 |
| FBgn0032200 | 0.916 | 0.999 | 1.487 | 1.698 | 2.069 |
| FBgn0031238 | 0.916 | 1.338 | 1.463 | 1.635 | 1.735 |
| FBgn0029924 | 0.916 | 0.848 | 0.838 | 0.971 | 1.062 |
| FBgn0032834 | 0.916 | 1.130 | 1.119 | 1.330 | 1.469 |
| FBgn0036606 | 0.916 | 0.346 | 0.200 | 0.117 | 0.011 |
| FBgn0259711 | 0.916 | 1.481 | 1.338 | 2.073 | 2.810 |
| FBgn0037057 | 0.916 | 0.889 | 0.903 | 0.839 | 0.684 |
| FBgn0031769 | 0.916 | 1.059 | 1.103 | 1.237 | 1.300 |
| FBgn0010424 | 0.915 | 0.932 | 0.972 | 1.103 | 1.166 |
| FBgn0002931 | 0.915 | 0.919 | 0.905 | 0.789 | 0.729 |
| FBgn0038424 | 0.915 | 1.216 | 1.241 | 1.022 | 0.855 |
| FBgn0016700 | 0.915 | 1.150 | 1.225 | 1.219 | 1.330 |
| FBgn0032230 | 0.915 | 1.324 | 1.529 | 1.998 | 2.224 |
| FBgn0040239 | 0.915 | 1.225 | 1.238 | 1.286 | 1.541 |
| FBgn0037690 | 0.914 | 2.813 | 3.917 | 4.385 | 5.221 |
| FBgn0038002 | 0.914 | 1.148 | 1.110 | 1.258 | 1.496 |
| FBgn0037782 | 0.914 | 1.597 | 1.773 | 3.540 | 4.456 |
| FBgn0037081 | 0.914 | 1.054 | 1.023 | 1.144 | 1.325 |
| FBgn0034367 | 0.914 | 0.944 | 0.939 | 1.090 | 1.184 |
| FBgn0038388 | 0.914 | 0.997 | 0.994 | 1.037 | 1.190 |
| FBgn0027101 | 0.914 | 2.271 | 2.196 | 2.979 | 3.538 |
| FBgn0036145 | 0.914 | 1.367 | 1.740 | 1.701 | 2.287 |
| FBgn0035977 | 0.914 | 1.138 | 1.486 | 1.440 | 2.087 |
| FBgn0032036 | 0.913 | 1.174 | 1.245 | 1.436 | 1.525 |
| FBgn0033479 | 0.913 | 0.993 | 0.844 | 0.575 | 0.439 |
| FBgn0038679 | 0.913 | 0.904 | 0.880 | 1.031 | 1.156 |
| FBgn0035876 | 0.913 | 0.714 | 0.666 | 0.859 | 1.106 |
| FBgn0034002 | 0.913 | 1.022 | 0.979 | 1.189 | 1.398 |
| FBgn0039031 | 0.912 | 1.026 | 1.050 | 1.053 | 1.159 |
| FBgn0262096 | 0.912 | 0.347 | 0.383 | 0.390 | 0.567 |
| FBgn0034398 | 0.911 | 1.259 | 1.287 | 1.804 | 2.078 |
| FBgn0031726 | 0.911 | 1.118 | 1.418 | 1.474 | 1.738 |
| FBgn0010014 | 0.911 | 1.071 | 1.201 | 1.401 | 1.507 |
| FBgn0033497 | 0.911 | 1.028 | 0.989 | 1.154 | 1.336 |
| FBgn0039161 | 0.911 | 0.380 | 0.376 | 0.459 | 0.775 |
| FBgn0010414 | 0.911 | 1.011 | 1.172 | 1.268 | 1.380 |
| FBgn0037960 | 0.911 | 0.612 | 0.705 | 1.157 | 1.367 |
| FBgn0034582 | 0.910 | 1.161 | 1.039 | 1.043 | 0.896 |
| FBgn0026777 | 0.910 | 1.133 | 1.435 | 1.966 | 2.229 |
| FBgn0035490 | 0.910 | 0.728 | 0.693 | 0.865 | 1.026 |
| FBgn0039663 | 0.909 | 1.263 | 1.167 | 0.954 | 0.855 |
| FBgn0032166 | 0.909 | 1.593 | 1.666 | 2.018 | 2.178 |
| FBgn0039911 | 0.909 | 1.479 | 1.895 | 2.654 | 3.019 |
| FBgn0262115 | 0.909 | 1.188 | 1.405 | 1.948 | 2.191 |
| FBgn0011570 | 0.908 | 1.151 | 1.131 | 1.223 | 1.312 |
| FBgn0023520 | 0.908 | 0.922 | 0.989 | 1.149 | 2.205 |
| FBgn0031955 | 0.907 | 0.912 | 0.883 | 1.013 | 1.369 |
| FBgn0037009 | 0.907 | 1.281 | 1.444 | 1.959 | 2.184 |
| FBgn0051641 | 0.907 | 0.955 | 0.932 | 0.770 | 0.695 |
| FBgn0039747 | 0.907 | 0.797 | 0.780 | 0.716 | 0.688 |
| FBgn0028424 | 0.906 | 1.005 | 1.010 | 1.300 | 1.454 |
| FBgn0036662 | 0.906 | 1.056 | 0.959 | 0.852 | 0.786 |
| FBgn0005696 | 0.906 | 1.001 | 0.963 | 0.789 | 0.713 |
| FBgn0034468 | 0.906 | 1.269 | 1.326 | 0.997 | 0.733 |
| FBgn0035611 | 0.905 | 1.274 | 1.151 | 1.731 | 2.261 |
| FBgn0036578 | 0.905 | 1.036 | 1.084 | 1.446 | 1.612 |
| FBgn0028526 | 0.905 | 1.397 | 1.290 | 1.909 | 2.399 |
| FBgn0037670 | 0.905 | 2.581 | 2.504 | 3.133 | 3.564 |
| FBgn0031630 | 0.905 | 0.875 | 1.104 | 1.203 | 1.365 |
| FBgn0083971 | 0.905 | 0.912 | 0.881 | 1.015 | 1.144 |
| FBgn0032404 | 0.905 | 0.789 | 0.769 | 0.925 | 1.033 |
| FBgn0039728 | 0.904 | 1.633 | 1.596 | 1.959 | 3.374 |
| FBgn0025456 | 0.904 | 1.204 | 1.166 | 1.367 | 1.530 |
| FBgn0023542 | 0.904 | 1.125 | 1.086 | 1.295 | 1.468 |
| FBgn0027356 | 0.904 | 0.865 | 0.854 | 1.047 | 1.160 |
| FBgn0032586 | 0.904 | 1.038 | 1.017 | 0.896 | 0.843 |
| FBgn0036587 | 0.903 | 2.082 | 2.106 | 4.194 | 5.274 |
| FBgn0052170 | 0.903 | 1.347 | 1.392 | 1.507 | 1.557 |
| FBgn0034480 | 0.903 | 1.063 | 1.500 | 1.434 | 2.129 |
| FBgn0016078 | 0.903 | 1.190 | 1.199 | 1.500 | 1.651 |
| FBgn0034179 | 0.903 | 1.052 | 1.134 | 1.437 | 1.564 |
| FBgn0031738 | 0.903 | 0.925 | 0.937 | 0.937 | 0.993 |
| FBgn0085227 | 0.903 | 0.890 | 0.782 | 0.796 | 0.635 |
| FBgn0086687 | 0.903 | 0.931 | 0.922 | 0.971 | 1.138 |
| FBgn0047334 | 0.902 | 1.475 | 1.467 | 1.431 | 1.218 |
| FBgn0036820 | 0.902 | 1.339 | 1.766 | 2.240 | 2.521 |
| FBgn0032388 | 0.902 | 1.055 | 1.028 | 1.266 | 1.421 |
| FBgn0034804 | 0.902 | 1.072 | 1.046 | 1.312 | 1.479 |
| FBgn0031250 | 0.902 | 0.895 | 0.933 | 1.094 | 1.161 |
| FBgn0033032 | 0.901 | 1.239 | 1.335 | 1.806 | 2.005 |
| FBgn0261872 | 0.901 | 1.352 | 1.311 | 1.485 | 1.647 |
| FBgn0038484 | 0.901 | 1.397 | 1.742 | 1.908 | 2.140 |
| FBgn0037617 | 0.901 | 1.217 | 1.343 | 1.320 | 1.548 |
| FBgn0034553 | 0.901 | 0.861 | 1.095 | 1.441 | 1.609 |
| FBgn0034950 | 0.901 | 1.714 | 1.583 | 2.065 | 2.592 |
| FBgn0035309 | 0.901 | 0.799 | 0.883 | 1.235 | 1.380 |
| FBgn0033782 | 0.901 | 0.630 | 0.776 | 0.923 | 1.017 |
| FBgn0033945 | 0.901 | 1.244 | 1.631 | 2.660 | 3.085 |
| FBgn0010238 | 0.901 | 1.034 | 1.005 | 1.208 | 1.348 |
| FBgn0034897 | 0.901 | 1.120 | 1.216 | 1.504 | 1.622 |
| FBgn0036576 | 0.900 | 0.769 | 0.926 | 1.076 | 1.175 |
| FBgn0038590 | 0.900 | 1.324 | 1.338 | 1.365 | 1.579 |
| FBgn0038420 | 0.900 | 1.816 | 1.945 | 2.763 | 3.114 |
| FBgn0052227 | 0.900 | 1.092 | 1.097 | 1.132 | 1.330 |
| FBgn0015569 | 0.900 | 1.188 | 1.142 | 1.422 | 1.626 |
| FBgn0040600 | 0.900 | 1.115 | 1.113 | 1.163 | 1.192 |

| Supplemental Table 3D. Values of R Squared and Fold Changes for Genes Found to Behave Linearly at Days 2 and 10 Post-Irradiation. (Analysis with all lowest dose discluded.) | | | | | |  |  |  |  |  |
| --- | --- | --- | --- | --- | --- | --- | --- | --- | --- | --- |
|  |  |  |  |  |  |  |  |  |  |  |
| Flybase ID | R Squared Value Day2 | R Squared Value Day10 | Day 2 Fold Change 1000R | Day 2 Fold Change 5000R | Day 2 Fold Change 10000R | Day 2 Fold Change 20000R | Day10 Fold Change 1000R | Day10 Fold Change 5000R | Day10 Fold Change 10000R | Day10 Fold Change 20000R |
| FBgn0002780 | 0.937 | 0.904 | 0.820 | 0.814 | 0.781 | 0.662 | 0.805 | 0.784 | 1.091 | 1.276 |
| FBgn0003174 | 0.930 | 0.990 | 1.066 | 1.031 | 1.239 | 1.496 | 0.891 | 0.993 | 1.121 | 1.298 |
| FBgn0011774 | 0.969 | 0.970 | 0.954 | 1.315 | 1.428 | 1.933 | 1.035 | 1.228 | 1.975 | 2.650 |
| FBgn0013432 | 0.979 | 0.927 | 0.859 | 0.917 | 0.980 | 1.215 | 0.739 | 0.865 | 0.881 | 1.342 |
| FBgn0013433 | 0.920 | 0.908 | 0.942 | 0.932 | 1.064 | 1.161 | 0.902 | 0.918 | 1.100 | 1.189 |
| FBgn0016650 | 0.966 | 0.984 | 1.020 | 1.093 | 1.171 | 1.257 | 0.863 | 0.924 | 1.101 | 1.301 |
| FBgn0020521 | 0.940 | 0.939 | 1.092 | 1.087 | 1.208 | 1.316 | 1.097 | 1.123 | 1.334 | 1.475 |
| FBgn0023001 | 0.938 | 0.918 | 1.004 | 1.008 | 1.106 | 1.177 | 0.994 | 0.967 | 1.193 | 1.373 |
| FBgn0023416 | 0.961 | 0.958 | 1.053 | 0.985 | 0.973 | 0.835 | 0.885 | 0.897 | 1.102 | 1.541 |
| FBgn0023541 | 0.935 | 0.965 | 0.973 | 0.901 | 0.902 | 0.753 | 0.827 | 0.855 | 1.121 | 1.386 |
| FBgn0024732 | 0.973 | 0.997 | 0.974 | 0.998 | 1.099 | 1.198 | 0.802 | 0.931 | 1.057 | 1.394 |
| FBgn0024912 | 0.947 | 0.990 | 1.391 | 1.592 | 1.717 | 1.904 | 1.051 | 1.549 | 1.898 | 2.703 |
| FBgn0027101 | 0.906 | 0.914 | 1.100 | 1.091 | 1.057 | 0.855 | 0.882 | 0.854 | 1.081 | 1.808 |
| FBgn0027491 | 0.945 | 0.998 | 1.068 | 1.073 | 1.001 | 0.918 | 1.005 | 1.054 | 1.112 | 1.251 |
| FBgn0030026 | 0.948 | 0.974 | 1.122 | 1.183 | 1.213 | 1.467 | 0.938 | 1.052 | 1.397 | 1.718 |
| FBgn0030041 | 0.946 | 0.990 | 1.129 | 1.117 | 1.093 | 0.989 | 1.011 | 1.091 | 1.321 | 1.620 |
| FBgn0030189 | 0.938 | 0.972 | 0.777 | 0.848 | 1.010 | 1.108 | 0.992 | 1.010 | 1.195 | 1.489 |
| FBgn0030234 | 0.968 | 0.961 | 1.153 | 1.162 | 1.301 | 1.512 | 1.070 | 1.079 | 1.227 | 1.527 |
| FBgn0031713 | 0.989 | 0.994 | 1.021 | 1.110 | 1.158 | 1.324 | 1.034 | 1.183 | 1.417 | 1.739 |
| FBgn0032393 | 0.974 | 0.981 | 1.135 | 1.347 | 1.423 | 1.734 | 1.130 | 1.699 | 2.025 | 2.818 |
| FBgn0032538 | 0.925 | 0.978 | 1.175 | 1.591 | 1.574 | 2.660 | 1.209 | 1.610 | 2.392 | 4.681 |
| FBgn0033187 | 0.900 | 0.919 | 0.992 | 1.003 | 0.899 | 0.834 | 0.974 | 0.906 | 0.770 | 0.700 |
| FBgn0033203 | 0.902 | 0.974 | 0.937 | 0.967 | 0.868 | 0.659 | 0.946 | 0.895 | 0.740 | 0.596 |
| FBgn0033348 | 0.945 | 0.933 | 0.837 | 0.834 | 0.833 | 0.824 | 1.160 | 1.170 | 1.711 | 3.379 |
| FBgn0033397 | 0.995 | 0.948 | 0.881 | 0.912 | 0.981 | 1.102 | 0.927 | 0.889 | 1.347 | 1.992 |
| FBgn0033926 | 0.931 | 0.987 | 1.108 | 1.534 | 1.668 | 3.537 | 1.533 | 1.747 | 2.548 | 3.675 |
| FBgn0033927 | 0.940 | 0.981 | 1.217 | 1.469 | 1.907 | 4.087 | 1.570 | 1.781 | 2.694 | 3.778 |
| FBgn0034184 | 0.952 | 0.988 | 1.033 | 1.036 | 1.039 | 1.055 | 1.031 | 1.072 | 1.137 | 1.313 |
| FBgn0035471 | 0.959 | 0.915 | 0.969 | 0.993 | 0.997 | 1.051 | 0.828 | 0.835 | 0.983 | 1.065 |
| FBgn0036223 | 0.934 | 0.925 | 1.119 | 1.144 | 0.988 | 0.741 | 0.950 | 0.998 | 1.175 | 1.268 |
| FBgn0036290 | 0.963 | 0.996 | 0.797 | 0.975 | 1.110 | 1.303 | 0.797 | 1.069 | 1.496 | 2.118 |
| FBgn0037020 | 0.986 | 0.934 | 0.938 | 0.953 | 1.001 | 1.089 | 0.927 | 1.089 | 1.107 | 1.298 |
| FBgn0037531 | 0.976 | 0.905 | 0.855 | 0.918 | 0.985 | 1.252 | 1.120 | 1.413 | 1.461 | 2.993 |
| FBgn0037652 | 0.984 | 0.991 | 1.117 | 1.089 | 0.971 | 0.804 | 1.087 | 1.135 | 1.229 | 1.349 |
| FBgn0037850 | 0.941 | 0.959 | 1.320 | 2.346 | 2.565 | 3.692 | 1.263 | 2.117 | 2.280 | 3.514 |
| FBgn0037922 | 0.947 | 0.924 | 1.061 | 1.066 | 0.957 | 0.840 | 1.111 | 1.143 | 0.938 | 0.737 |
| FBgn0038252 | 0.956 | 0.959 | 0.777 | 0.660 | 0.464 | 0.310 | 1.187 | 1.162 | 0.638 | 0.108 |
| FBgn0039709 | 0.967 | 0.993 | 1.065 | 1.111 | 1.825 | 2.675 | 1.041 | 1.093 | 1.223 | 1.407 |
| FBgn0046763 | 0.943 | 0.998 | 0.940 | 0.958 | 1.078 | 1.159 | 0.875 | 1.061 | 1.225 | 1.614 |
| FBgn0050203 | 0.972 | 0.916 | 1.293 | 1.202 | 1.167 | 0.930 | 0.897 | 0.955 | 1.025 | 1.564 |
| FBgn0051864 | 0.978 | 0.965 | 1.096 | 1.138 | 1.421 | 1.832 | 1.072 | 1.596 | 1.711 | 2.806 |
| FBgn0052751 | 0.942 | 0.953 | 1.641 | 1.356 | 1.179 | 0.925 | 0.540 | 0.602 | 0.927 | 1.894 |
| FBgn0053096 | 0.981 | 0.926 | 1.076 | 1.006 | 0.943 | 0.847 | 1.131 | 0.951 | 0.808 | 0.669 |
| FBgn0085360 | 0.932 | 0.902 | 0.928 | 1.022 | 1.115 | 1.193 | 0.767 | 1.095 | 1.075 | 1.472 |
| FBgn0261049 | 0.945 | 0.936 | 1.027 | 0.944 | 0.934 | 0.823 | 0.964 | 0.955 | 1.028 | 1.113 |

| Supplemental Table 3E. Values of R Squared and Fold Changes for Genes Found to Behave Linearly at Days 10 and 20 Post-Irradiation. (Analysis with all lowest dose discluded.) | | | | | | | |  |  |  |
| --- | --- | --- | --- | --- | --- | --- | --- | --- | --- | --- |
|  |  |  |  |  |  |  |  |  |  |  |
| Flybase ID | R Squared Value Day10 | r Squared Value Day20 | Day10 Fold Change 1000R | Day10 Fold Change 5000R | Day10 Fold Change 10000R | Day10 Fold Change 20000R | Day 20 Fold Change 1000R | Day 20 Fold Change 5000R | Day 20 Fold Change 10000R | Day 20 Fold Change 20000R |
| FBgn0000316 | 0.929 | 0.957 | 0.881 | 0.985 | 0.981 | 1.228 | 1.052 | 1.050 | 1.192 | 1.383 |
| FBgn0003071 | 0.961 | 0.922 | 1.111 | 0.953 | 0.816 | 0.647 | 0.961 | 0.976 | 0.787 | 0.647 |
| FBgn0003141 | 0.949 | 0.983 | 0.814 | 0.941 | 0.984 | 1.434 | 1.353 | 1.515 | 1.899 | 2.312 |
| FBgn0003326 | 0.909 | 0.919 | 0.993 | 1.152 | 1.649 | 1.869 | 0.832 | 0.858 | 0.881 | 1.077 |
| FBgn0003495 | 0.944 | 0.945 | 0.988 | 0.997 | 1.363 | 1.662 | 0.925 | 0.914 | 1.135 | 1.602 |
| FBgn0010038 | 0.956 | 0.992 | 2.277 | 5.455 | 6.142 | 10.345 | 0.766 | 1.195 | 1.706 | 3.165 |
| FBgn0010173 | 0.964 | 0.926 | 1.079 | 1.116 | 1.383 | 1.629 | 1.309 | 1.238 | 1.770 | 2.273 |
| FBgn0010278 | 0.944 | 0.978 | 1.066 | 1.061 | 1.214 | 1.561 | 0.886 | 0.917 | 1.016 | 1.244 |
| FBgn0010315 | 0.905 | 0.939 | 1.043 | 1.131 | 1.532 | 1.706 | 1.145 | 1.205 | 1.298 | 1.361 |
| FBgn0010333 | 0.992 | 0.925 | 0.986 | 1.045 | 1.082 | 1.185 | 0.894 | 0.910 | 0.922 | 0.935 |
| FBgn0010348 | 0.993 | 0.916 | 0.872 | 0.941 | 1.061 | 1.219 | 1.132 | 1.179 | 1.417 | 1.534 |
| FBgn0010350 | 0.963 | 0.987 | 0.984 | 0.895 | 0.798 | 0.695 | 0.787 | 0.764 | 0.694 | 0.611 |
| FBgn0011509 | 0.999 | 0.981 | 0.946 | 1.020 | 1.101 | 1.293 | 1.179 | 1.230 | 1.497 | 1.865 |
| FBgn0011570 | 0.930 | 0.908 | 1.007 | 1.083 | 1.209 | 1.286 | 1.151 | 1.131 | 1.223 | 1.312 |
| FBgn0011591 | 0.963 | 0.938 | 0.979 | 1.028 | 1.205 | 1.348 | 0.871 | 1.046 | 1.338 | 1.530 |
| FBgn0011774 | 0.970 | 0.960 | 1.035 | 1.228 | 1.975 | 2.650 | 1.096 | 1.113 | 1.871 | 2.722 |
| FBgn0014022 | 0.928 | 0.925 | 1.018 | 1.194 | 1.186 | 1.484 | 0.551 | 0.635 | 0.631 | 0.762 |
| FBgn0015351 | 0.963 | 0.987 | 1.072 | 1.131 | 1.446 | 2.242 | 1.171 | 1.332 | 1.589 | 2.294 |
| FBgn0015618 | 0.931 | 0.919 | 0.939 | 0.927 | 1.008 | 1.103 | 0.888 | 1.080 | 1.144 | 1.302 |
| FBgn0016041 | 0.998 | 0.937 | 1.076 | 1.007 | 0.883 | 0.651 | 0.973 | 0.923 | 0.764 | 0.670 |
| FBgn0016696 | 0.920 | 0.973 | 1.019 | 1.020 | 1.285 | 1.449 | 1.026 | 1.093 | 1.235 | 1.364 |
| FBgn0020270 | 0.976 | 0.971 | 1.036 | 1.096 | 1.529 | 2.202 | 1.133 | 1.155 | 1.404 | 1.773 |
| FBgn0020303 | 0.946 | 0.922 | 0.921 | 0.893 | 1.207 | 1.614 | 0.977 | 0.950 | 1.081 | 1.248 |
| FBgn0020416 | 0.946 | 0.952 | 0.778 | 0.760 | 0.958 | 1.255 | 1.304 | 1.290 | 1.732 | 2.223 |
| FBgn0023000 | 0.991 | 0.971 | 0.910 | 1.010 | 1.237 | 1.522 | 1.167 | 1.204 | 1.648 | 2.278 |
| FBgn0023514 | 0.998 | 0.993 | 0.989 | 1.071 | 1.205 | 1.423 | 1.207 | 1.274 | 1.341 | 1.457 |
| FBgn0023546 | 0.908 | 0.982 | 0.935 | 0.788 | 1.422 | 2.073 | 0.542 | 0.606 | 0.931 | 1.405 |
| FBgn0024912 | 0.990 | 0.987 | 1.051 | 1.549 | 1.898 | 2.703 | 1.400 | 1.985 | 2.959 | 4.117 |
| FBgn0024913 | 0.916 | 0.978 | 0.941 | 1.054 | 1.342 | 3.008 | 1.339 | 1.757 | 1.967 | 3.099 |
| FBgn0025366 | 0.986 | 0.930 | 0.889 | 1.024 | 1.139 | 1.336 | 1.068 | 1.106 | 1.269 | 1.361 |
| FBgn0025583 | 0.971 | 0.997 | 0.860 | 1.225 | 1.327 | 1.917 | 1.192 | 1.835 | 2.344 | 3.712 |
| FBgn0025807 | 0.970 | 0.979 | 1.012 | 1.145 | 1.209 | 1.612 | 1.065 | 1.121 | 1.167 | 1.363 |
| FBgn0025879 | 0.988 | 0.940 | 0.904 | 0.961 | 1.129 | 1.339 | 1.100 | 1.179 | 1.534 | 1.757 |
| FBgn0026263 | 0.904 | 0.961 | 1.033 | 1.003 | 1.359 | 1.580 | 0.808 | 0.849 | 1.168 | 1.454 |
| FBgn0027101 | 0.914 | 0.914 | 0.882 | 0.854 | 1.081 | 1.808 | 2.271 | 2.196 | 2.979 | 3.538 |
| FBgn0028394 | 0.936 | 0.969 | 0.951 | 1.051 | 1.131 | 1.215 | 1.317 | 1.445 | 1.478 | 1.682 |
| FBgn0028411 | 0.945 | 0.941 | 1.246 | 1.759 | 1.816 | 2.514 | 2.019 | 2.891 | 3.009 | 4.087 |
| FBgn0028424 | 0.931 | 0.906 | 0.776 | 0.854 | 1.168 | 1.345 | 1.005 | 1.010 | 1.300 | 1.454 |
| FBgn0028526 | 0.909 | 0.905 | 0.870 | 0.800 | 1.058 | 1.413 | 1.397 | 1.290 | 1.909 | 2.399 |
| FBgn0028546 | 0.927 | 0.938 | 1.057 | 1.153 | 1.401 | 1.534 | 1.310 | 1.397 | 1.858 | 2.146 |
| FBgn0028853 | 0.939 | 0.978 | 0.774 | 1.011 | 1.155 | 1.362 | 1.078 | 1.351 | 1.777 | 2.224 |
| FBgn0028879 | 0.902 | 0.920 | 0.887 | 0.805 | 1.084 | 1.452 | 1.049 | 1.246 | 1.267 | 1.473 |
| FBgn0029664 | 0.991 | 0.987 | 0.932 | 0.979 | 1.056 | 1.247 | 1.441 | 1.489 | 1.631 | 1.906 |
| FBgn0029737 | 0.942 | 0.958 | 1.063 | 0.972 | 0.916 | 0.835 | 0.933 | 0.924 | 0.833 | 0.753 |
| FBgn0029912 | 0.943 | 0.948 | 1.116 | 1.087 | 1.073 | 0.946 | 0.984 | 0.988 | 0.938 | 0.875 |
| FBgn0029913 | 0.999 | 0.953 | 0.920 | 1.018 | 1.170 | 1.424 | 1.689 | 1.713 | 1.799 | 2.087 |
| FBgn0029997 | 0.928 | 0.931 | 1.039 | 0.881 | 0.765 | 0.641 | 0.870 | 0.873 | 0.736 | 0.632 |
| FBgn0030038 | 0.999 | 0.973 | 1.024 | 0.957 | 0.885 | 0.744 | 1.010 | 0.957 | 0.892 | 0.822 |
| FBgn0030157 | 0.945 | 0.923 | 0.846 | 0.849 | 1.011 | 1.418 | 1.357 | 1.449 | 1.863 | 2.080 |
| FBgn0030189 | 0.972 | 0.992 | 0.992 | 1.010 | 1.195 | 1.489 | 1.168 | 1.303 | 1.424 | 1.817 |
| FBgn0031250 | 0.944 | 0.902 | 0.976 | 1.080 | 1.114 | 1.220 | 0.895 | 0.933 | 1.094 | 1.161 |
| FBgn0031710 | 0.931 | 0.958 | 0.772 | 0.904 | 0.900 | 1.192 | 1.211 | 1.238 | 1.327 | 1.611 |
| FBgn0031726 | 0.990 | 0.911 | 0.984 | 1.164 | 1.639 | 2.257 | 1.118 | 1.418 | 1.474 | 1.738 |
| FBgn0031910 | 0.959 | 0.937 | 0.919 | 1.437 | 2.976 | 4.141 | 0.726 | 0.800 | 0.894 | 0.964 |
| FBgn0032036 | 0.902 | 0.913 | 0.945 | 1.024 | 1.262 | 1.362 | 1.174 | 1.245 | 1.436 | 1.525 |
| FBgn0032166 | 0.951 | 0.909 | 0.927 | 0.930 | 1.027 | 1.115 | 1.593 | 1.666 | 2.018 | 2.178 |
| FBgn0032192 | 0.930 | 0.985 | 0.671 | 1.241 | 1.419 | 1.938 | 1.420 | 1.749 | 1.943 | 2.790 |
| FBgn0032200 | 0.925 | 0.916 | 1.214 | 1.219 | 1.290 | 1.557 | 0.999 | 1.487 | 1.698 | 2.069 |
| FBgn0032230 | 0.985 | 0.915 | 1.122 | 1.343 | 1.544 | 2.284 | 1.324 | 1.529 | 1.998 | 2.224 |
| FBgn0032283 | 0.970 | 0.956 | 1.537 | 1.441 | 1.251 | 1.082 | 1.380 | 1.388 | 1.109 | 0.673 |
| FBgn0032393 | 0.981 | 0.947 | 1.130 | 1.699 | 2.025 | 2.818 | 1.020 | 1.398 | 2.248 | 2.817 |
| FBgn0032470 | 0.997 | 0.989 | 1.184 | 1.421 | 1.889 | 2.696 | 1.023 | 1.379 | 1.656 | 2.674 |
| FBgn0032472 | 0.943 | 0.958 | 0.768 | 0.907 | 0.975 | 1.588 | 0.983 | 1.432 | 1.883 | 2.360 |
| FBgn0032586 | 0.929 | 0.904 | 1.034 | 0.964 | 0.828 | 0.749 | 1.038 | 1.017 | 0.896 | 0.843 |
| FBgn0032669 | 0.941 | 0.949 | 1.019 | 1.099 | 1.355 | 1.514 | 1.916 | 2.019 | 2.718 | 3.233 |
| FBgn0032719 | 0.996 | 0.962 | 0.928 | 1.064 | 1.195 | 1.445 | 0.869 | 0.887 | 1.025 | 1.151 |
| FBgn0032731 | 0.924 | 0.957 | 0.974 | 0.995 | 1.052 | 1.346 | 0.935 | 1.001 | 1.097 | 1.177 |
| FBgn0032819 | 0.979 | 0.959 | 0.910 | 0.983 | 1.290 | 1.904 | 1.084 | 1.310 | 1.353 | 1.679 |
| FBgn0032859 | 0.905 | 0.940 | 1.152 | 1.141 | 1.179 | 1.232 | 1.221 | 1.291 | 1.315 | 1.594 |
| FBgn0033032 | 0.997 | 0.901 | 0.909 | 1.018 | 1.108 | 1.358 | 1.239 | 1.335 | 1.806 | 2.005 |
| FBgn0033130 | 0.936 | 0.960 | 1.019 | 1.233 | 1.251 | 1.886 | 1.304 | 1.352 | 1.796 | 2.199 |
| FBgn0033134 | 0.999 | 0.962 | 0.868 | 1.056 | 1.333 | 1.866 | 1.832 | 2.253 | 3.542 | 4.562 |
| FBgn0033153 | 0.913 | 0.957 | 1.028 | 1.422 | 1.980 | 2.291 | 1.249 | 1.641 | 1.785 | 2.227 |
| FBgn0033453 | 0.987 | 0.953 | 0.835 | 1.168 | 1.339 | 1.900 | 1.092 | 1.799 | 2.498 | 3.213 |
| FBgn0033458 | 0.991 | 0.930 | 1.192 | 1.349 | 1.812 | 2.532 | 1.099 | 1.058 | 1.356 | 1.660 |
| FBgn0033668 | 0.932 | 0.933 | 0.959 | 1.179 | 1.706 | 4.261 | 0.802 | 1.056 | 1.865 | 2.324 |
| FBgn0033744 | 0.954 | 0.937 | 0.902 | 0.913 | 1.058 | 1.414 | 1.013 | 0.998 | 1.116 | 1.340 |
| FBgn0033812 | 0.979 | 0.927 | 0.970 | 0.989 | 1.038 | 1.086 | 0.923 | 0.969 | 0.993 | 1.029 |
| FBgn0033926 | 0.987 | 0.921 | 1.533 | 1.747 | 2.548 | 3.675 | 1.163 | 1.042 | 1.610 | 2.730 |
| FBgn0033927 | 0.981 | 0.936 | 1.570 | 1.781 | 2.694 | 3.778 | 1.156 | 1.082 | 1.636 | 2.703 |
| FBgn0033928 | 0.996 | 0.961 | 1.364 | 1.837 | 2.217 | 3.148 | 1.694 | 1.700 | 2.473 | 3.460 |
| FBgn0033945 | 0.954 | 0.901 | 1.265 | 1.385 | 1.693 | 2.809 | 1.244 | 1.631 | 2.660 | 3.085 |
| FBgn0034002 | 0.925 | 0.913 | 0.938 | 1.205 | 1.186 | 1.650 | 1.022 | 0.979 | 1.189 | 1.398 |
| FBgn0034184 | 0.988 | 0.982 | 1.031 | 1.072 | 1.137 | 1.313 | 0.948 | 1.018 | 1.052 | 1.222 |
| FBgn0034199 | 0.954 | 0.986 | 0.885 | 1.064 | 1.242 | 1.424 | 1.093 | 1.374 | 1.582 | 1.989 |
| FBgn0034398 | 0.972 | 0.911 | 0.837 | 1.078 | 1.174 | 1.495 | 1.259 | 1.287 | 1.804 | 2.078 |
| FBgn0034480 | 0.961 | 0.903 | 1.195 | 1.283 | 1.589 | 2.498 | 1.063 | 1.500 | 1.434 | 2.129 |
| FBgn0034512 | 0.996 | 0.979 | 0.957 | 1.058 | 1.244 | 1.512 | 1.267 | 1.347 | 1.709 | 2.406 |
| FBgn0034530 | 0.900 | 0.937 | 1.050 | 1.041 | 1.150 | 1.617 | 0.757 | 0.823 | 1.192 | 1.421 |
| FBgn0034605 | 0.980 | 0.978 | 1.046 | 1.370 | 1.555 | 1.999 | 1.206 | 1.464 | 1.628 | 1.963 |
| FBgn0034627 | 0.902 | 0.934 | 0.834 | 0.840 | 0.871 | 1.055 | 0.637 | 0.699 | 1.066 | 1.287 |
| FBgn0034656 | 0.983 | 0.930 | 0.855 | 0.889 | 1.025 | 1.186 | 1.116 | 1.161 | 1.369 | 1.487 |
| FBgn0034726 | 0.988 | 0.960 | 0.890 | 0.988 | 1.070 | 1.367 | 0.967 | 1.211 | 1.327 | 1.594 |
| FBgn0034804 | 0.904 | 0.902 | 1.073 | 1.223 | 1.229 | 1.382 | 1.072 | 1.046 | 1.312 | 1.479 |
| FBgn0035089 | 0.929 | 0.918 | 0.903 | 1.234 | 1.232 | 2.122 | 1.802 | 2.613 | 2.878 | 3.542 |
| FBgn0035165 | 0.983 | 0.925 | 0.944 | 1.058 | 1.119 | 1.286 | 0.974 | 1.099 | 1.115 | 1.249 |
| FBgn0035166 | 0.967 | 0.969 | 1.024 | 1.081 | 1.357 | 1.602 | 0.745 | 0.892 | 0.939 | 1.297 |
| FBgn0035452 | 0.919 | 0.938 | 0.802 | 1.108 | 1.316 | 1.540 | 1.710 | 2.162 | 3.029 | 3.575 |
| FBgn0035587 | 0.967 | 0.982 | 0.873 | 0.971 | 1.091 | 1.215 | 1.160 | 1.180 | 1.235 | 1.365 |
| FBgn0035611 | 0.999 | 0.905 | 0.982 | 1.184 | 1.413 | 1.951 | 1.274 | 1.151 | 1.731 | 2.261 |
| FBgn0035805 | 0.973 | 0.917 | 0.882 | 0.920 | 1.173 | 1.452 | 1.217 | 1.530 | 2.090 | 2.386 |
| FBgn0035816 | 0.943 | 0.957 | 0.852 | 0.831 | 1.052 | 1.448 | 0.783 | 0.787 | 0.856 | 1.008 |
| FBgn0035876 | 0.995 | 0.913 | 0.871 | 0.999 | 1.104 | 1.335 | 0.714 | 0.666 | 0.859 | 1.106 |
| FBgn0035998 | 0.920 | 0.961 | 0.959 | 0.911 | 1.118 | 1.493 | 1.217 | 1.521 | 1.923 | 2.292 |
| FBgn0036145 | 0.974 | 0.914 | 0.748 | 0.879 | 1.177 | 1.453 | 1.367 | 1.740 | 1.701 | 2.287 |
| FBgn0036232 | 0.991 | 0.979 | 0.771 | 0.863 | 0.975 | 1.135 | 1.609 | 1.905 | 2.160 | 2.552 |
| FBgn0036290 | 0.996 | 0.996 | 0.797 | 1.069 | 1.496 | 2.118 | 1.438 | 1.834 | 2.689 | 4.152 |
| FBgn0036768 | 0.959 | 0.924 | 0.941 | 0.947 | 1.237 | 1.565 | 0.879 | 0.873 | 0.910 | 0.998 |
| FBgn0036889 | 0.996 | 0.967 | 0.960 | 1.035 | 1.123 | 1.355 | 1.294 | 1.344 | 1.463 | 1.562 |
| FBgn0037020 | 0.934 | 0.954 | 0.927 | 1.089 | 1.107 | 1.298 | 0.996 | 0.993 | 1.100 | 1.229 |
| FBgn0037057 | 0.998 | 0.916 | 1.090 | 1.047 | 0.992 | 0.900 | 0.889 | 0.903 | 0.839 | 0.684 |
| FBgn0037076 | 0.971 | 0.928 | 1.039 | 1.271 | 1.566 | 1.877 | 1.320 | 1.791 | 1.788 | 3.083 |
| FBgn0037518 | 0.925 | 0.954 | 0.798 | 0.836 | 1.040 | 1.151 | 1.181 | 1.190 | 1.322 | 1.641 |
| FBgn0037617 | 0.962 | 0.901 | 1.017 | 1.029 | 1.318 | 1.645 | 1.217 | 1.343 | 1.320 | 1.548 |
| FBgn0037731 | 0.984 | 0.955 | 0.973 | 1.059 | 1.436 | 1.996 | 1.164 | 1.338 | 1.787 | 2.110 |
| FBgn0037818 | 0.991 | 0.925 | 0.959 | 0.991 | 1.087 | 1.240 | 1.481 | 1.463 | 2.116 | 2.573 |
| FBgn0037850 | 0.959 | 0.996 | 1.263 | 2.117 | 2.280 | 3.514 | 1.411 | 1.769 | 2.408 | 3.722 |
| FBgn0037936 | 0.949 | 0.920 | 0.822 | 0.835 | 0.946 | 1.258 | 1.283 | 1.632 | 2.307 | 2.663 |
| FBgn0037960 | 0.985 | 0.911 | 0.777 | 0.840 | 1.012 | 1.389 | 0.612 | 0.705 | 1.157 | 1.367 |
| FBgn0038455 | 0.963 | 0.987 | 1.072 | 1.131 | 1.446 | 2.242 | 1.171 | 1.332 | 1.589 | 2.294 |
| FBgn0038533 | 0.960 | 0.934 | 0.826 | 0.888 | 1.084 | 1.235 | 1.035 | 1.087 | 1.091 | 1.243 |
| FBgn0038795 | 0.950 | 0.933 | 0.952 | 1.052 | 1.142 | 1.678 | 1.305 | 1.502 | 2.317 | 2.784 |
| FBgn0038893 | 0.990 | 0.932 | 0.974 | 1.447 | 1.769 | 2.943 | 1.453 | 1.398 | 2.536 | 3.456 |
| FBgn0039132 | 0.955 | 0.995 | 0.908 | 0.979 | 1.039 | 1.390 | 1.702 | 1.869 | 2.256 | 2.904 |
| FBgn0039161 | 0.990 | 0.911 | 0.741 | 0.837 | 1.139 | 1.598 | 0.380 | 0.376 | 0.459 | 0.775 |
| FBgn0039342 | 0.966 | 0.973 | 1.054 | 1.147 | 1.616 | 2.035 | 1.103 | 1.585 | 2.318 | 3.043 |
| FBgn0039481 | 0.980 | 0.955 | 0.848 | 0.932 | 0.979 | 1.215 | 0.688 | 0.818 | 0.923 | 1.054 |
| FBgn0039544 | 0.957 | 0.983 | 1.094 | 1.090 | 1.321 | 1.631 | 1.191 | 1.243 | 1.365 | 1.497 |
| FBgn0039597 | 0.964 | 0.916 | 0.789 | 0.944 | 0.988 | 1.195 | 1.029 | 1.246 | 1.228 | 1.561 |
| FBgn0039670 | 0.980 | 0.954 | 0.942 | 1.234 | 1.551 | 1.961 | 0.928 | 1.205 | 1.273 | 1.613 |
| FBgn0039696 | 0.946 | 0.949 | 0.984 | 1.115 | 1.157 | 1.294 | 0.856 | 0.938 | 1.124 | 1.251 |
| FBgn0039911 | 0.985 | 0.909 | 0.853 | 0.923 | 1.045 | 1.378 | 1.479 | 1.895 | 2.654 | 3.019 |
| FBgn0039993 | 0.921 | 0.942 | 0.952 | 0.980 | 1.098 | 1.656 | 1.144 | 1.310 | 1.722 | 1.980 |
| FBgn0040732 | 0.928 | 0.989 | 0.603 | 0.820 | 0.910 | 1.093 | 0.955 | 1.079 | 1.296 | 1.847 |
| FBgn0040837 | 0.963 | 0.998 | 1.515 | 1.811 | 1.974 | 2.300 | 1.559 | 1.942 | 2.409 | 3.207 |
| FBgn0040972 | 0.905 | 0.999 | 0.829 | 1.270 | 1.317 | 3.444 | 1.042 | 2.190 | 3.282 | 5.736 |
| FBgn0046763 | 0.998 | 0.996 | 0.875 | 1.061 | 1.225 | 1.614 | 1.436 | 1.584 | 1.813 | 2.153 |
| FBgn0050026 | 0.937 | 0.953 | 0.825 | 0.998 | 0.996 | 1.324 | 1.169 | 1.259 | 1.553 | 2.557 |
| FBgn0050281 | 0.980 | 0.950 | 0.877 | 1.037 | 1.111 | 1.507 | 1.190 | 1.415 | 1.633 | 1.853 |
| FBgn0051036 | 0.938 | 0.948 | 1.026 | 1.277 | 1.386 | 2.519 | 0.921 | 0.957 | 1.452 | 1.848 |
| FBgn0051769 | 0.959 | 0.934 | 0.781 | 0.964 | 0.992 | 1.300 | 1.228 | 1.276 | 1.293 | 1.504 |
| FBgn0051778 | 0.909 | 0.935 | 0.666 | 0.884 | 0.849 | 1.304 | 1.299 | 1.486 | 2.368 | 2.893 |
| FBgn0051864 | 0.965 | 0.976 | 1.072 | 1.596 | 1.711 | 2.806 | 1.066 | 1.287 | 2.116 | 2.943 |
| FBgn0051961 | 0.971 | 0.991 | 0.855 | 0.870 | 0.984 | 1.107 | 1.189 | 1.251 | 1.379 | 1.658 |
| FBgn0052196 | 0.946 | 0.940 | 1.079 | 1.214 | 1.504 | 1.699 | 4.141 | 3.995 | 5.277 | 6.853 |
| FBgn0052640 | 0.929 | 0.983 | 0.815 | 0.933 | 1.120 | 2.219 | 1.621 | 1.936 | 2.795 | 3.720 |
| FBgn0052641 | 0.930 | 0.983 | 0.799 | 0.915 | 1.109 | 2.212 | 1.631 | 1.979 | 2.844 | 3.760 |
| FBgn0053109 | 0.907 | 0.955 | 0.962 | 0.996 | 1.082 | 1.642 | 1.104 | 1.265 | 1.508 | 1.707 |
| FBgn0053178 | 0.967 | 0.997 | 0.863 | 1.005 | 1.038 | 1.265 | 1.255 | 1.414 | 1.605 | 2.080 |
| FBgn0063494 | 0.974 | 0.931 | 0.901 | 1.091 | 1.162 | 1.434 | 1.382 | 1.376 | 1.879 | 2.249 |
| FBgn0085285 | 0.990 | 0.950 | 0.853 | 1.158 | 1.441 | 1.922 | 1.074 | 1.148 | 1.769 | 2.246 |
| FBgn0085435 | 0.957 | 0.985 | 0.734 | 0.823 | 1.451 | 1.964 | 0.308 | 0.330 | 0.416 | 0.566 |
| FBgn0086445 | 0.973 | 0.989 | 1.058 | 1.187 | 1.374 | 1.562 | 2.810 | 3.141 | 3.365 | 3.890 |
| FBgn0086676 | 0.910 | 0.987 | 1.032 | 1.058 | 1.198 | 1.264 | 0.680 | 0.710 | 0.811 | 0.943 |
| FBgn0087039 | 0.978 | 0.989 | 0.925 | 1.002 | 1.149 | 1.582 | 1.304 | 1.819 | 2.216 | 3.010 |
| FBgn0259722 | 0.930 | 0.956 | 0.881 | 0.985 | 0.981 | 1.223 | 1.052 | 1.049 | 1.192 | 1.381 |
| FBgn0260431 | 0.939 | 0.998 | 1.023 | 1.119 | 1.171 | 1.632 | 1.163 | 1.351 | 1.688 | 2.237 |
| FBgn0260432 | 0.908 | 0.982 | 0.935 | 0.788 | 1.422 | 2.073 | 0.542 | 0.606 | 0.931 | 1.405 |
| FBgn0260946 | 0.978 | 0.931 | 1.012 | 1.051 | 1.182 | 1.314 | 0.954 | 1.032 | 1.067 | 1.133 |
| FBgn0261871 | 0.903 | 0.997 | 0.960 | 0.868 | 1.165 | 1.729 | 1.063 | 1.118 | 1.223 | 1.382 |
| FBgn0261989 | 0.968 | 0.934 | 1.148 | 2.397 | 3.470 | 8.701 | 1.963 | 2.859 | 5.799 | 7.485 |
| FBgn0262057 | 0.969 | 0.934 | 0.997 | 1.008 | 1.117 | 1.245 | 1.112 | 1.109 | 1.265 | 1.385 |
| FBgn0262115 | 0.914 | 0.909 | 1.048 | 0.969 | 1.281 | 1.720 | 1.188 | 1.405 | 1.948 | 2.191 |
| FBgn0262524 | 0.945 | 0.988 | 1.069 | 1.440 | 1.502 | 2.592 | 1.032 | 1.371 | 1.607 | 2.124 |

| Supplemental Table 3F. Values of R Squared and Fold Changes for Genes Found to Behave Linearly at Days 2 and 20 Post-Irradiation. (Analysis with all lowest dose discluded.) | | | | | | |  |  |  |  |
| --- | --- | --- | --- | --- | --- | --- | --- | --- | --- | --- |
|  |  |  |  |  |  |  |  |  |  |  |
| Flybase ID | R Squared Value Day2 | R Squared Value Day20 | Day 2 Fold Change 1000R | Day 2 Fold Change 5000R | Day 2 Fold Change 10000R | Day 2 Fold Change 20000R | Day 20 Fold Change 1000R | Day 20 Fold Change 5000R | Day 20 Fold Change 10000R | Day 20 Fold Change 20000R |
| FBgn0004057 | 0.910 | 0.924 | 1.143 | 1.149 | 1.255 | 1.310 | 0.870 | 0.869 | 0.857 | 0.809 |
| FBgn0004666 | 0.982 | 0.921 | 1.043 | 1.193 | 1.281 | 1.494 | 1.112 | 1.065 | 1.274 | 1.567 |
| FBgn0010238 | 0.970 | 0.901 | 1.001 | 1.008 | 1.086 | 1.215 | 1.034 | 1.005 | 1.208 | 1.348 |
| FBgn0011774 | 0.969 | 0.960 | 0.954 | 1.315 | 1.428 | 1.933 | 1.096 | 1.113 | 1.871 | 2.722 |
| FBgn0022800 | 0.997 | 0.946 | 1.157 | 1.096 | 1.047 | 0.915 | 1.182 | 1.167 | 1.331 | 1.566 |
| FBgn0024912 | 0.947 | 0.987 | 1.391 | 1.592 | 1.717 | 1.904 | 1.400 | 1.985 | 2.959 | 4.117 |
| FBgn0024989 | 0.933 | 0.992 | 1.200 | 1.501 | 1.743 | 3.594 | 0.561 | 0.587 | 0.655 | 0.750 |
| FBgn0027101 | 0.906 | 0.914 | 1.100 | 1.091 | 1.057 | 0.855 | 2.271 | 2.196 | 2.979 | 3.538 |
| FBgn0030189 | 0.938 | 0.992 | 0.777 | 0.848 | 1.010 | 1.108 | 1.168 | 1.303 | 1.424 | 1.817 |
| FBgn0032393 | 0.974 | 0.947 | 1.135 | 1.347 | 1.423 | 1.734 | 1.020 | 1.398 | 2.248 | 2.817 |
| FBgn0033188 | 0.994 | 0.923 | 1.038 | 1.077 | 1.160 | 1.276 | 0.753 | 0.865 | 1.018 | 1.114 |
| FBgn0033926 | 0.931 | 0.921 | 1.108 | 1.534 | 1.668 | 3.537 | 1.163 | 1.042 | 1.610 | 2.730 |
| FBgn0033927 | 0.940 | 0.936 | 1.217 | 1.469 | 1.907 | 4.087 | 1.156 | 1.082 | 1.636 | 2.703 |
| FBgn0034184 | 0.952 | 0.982 | 1.033 | 1.036 | 1.039 | 1.055 | 0.948 | 1.018 | 1.052 | 1.222 |
| FBgn0036017 | 0.903 | 0.949 | 0.986 | 1.615 | 1.580 | 4.025 | 1.277 | 1.375 | 1.605 | 1.762 |
| FBgn0036290 | 0.963 | 0.996 | 0.797 | 0.975 | 1.110 | 1.303 | 1.438 | 1.834 | 2.689 | 4.152 |
| FBgn0037020 | 0.986 | 0.954 | 0.938 | 0.953 | 1.001 | 1.089 | 0.996 | 0.993 | 1.100 | 1.229 |
| FBgn0037291 | 0.909 | 0.983 | 1.082 | 1.107 | 0.910 | 0.767 | 1.045 | 0.895 | 0.605 | 0.291 |
| FBgn0037305 | 0.938 | 0.947 | 0.987 | 0.990 | 0.967 | 0.927 | 1.040 | 1.110 | 1.115 | 1.238 |
| FBgn0037850 | 0.941 | 0.996 | 1.320 | 2.346 | 2.565 | 3.692 | 1.411 | 1.769 | 2.408 | 3.722 |
| FBgn0040099 | 0.956 | 0.952 | 0.737 | 0.810 | 0.831 | 0.917 | 2.137 | 2.472 | 3.254 | 3.804 |
| FBgn0041342 | 0.961 | 0.980 | 1.128 | 1.122 | 1.084 | 1.053 | 0.942 | 0.975 | 0.996 | 1.094 |
| FBgn0041627 | 0.963 | 0.990 | 1.054 | 1.060 | 1.283 | 1.644 | 1.148 | 1.313 | 1.730 | 2.554 |
| FBgn0046763 | 0.943 | 0.996 | 0.940 | 0.958 | 1.078 | 1.159 | 1.436 | 1.584 | 1.813 | 2.153 |
| FBgn0051864 | 0.978 | 0.976 | 1.096 | 1.138 | 1.421 | 1.832 | 1.066 | 1.287 | 2.116 | 2.943 |
| FBgn0052220 | 0.921 | 0.955 | 0.976 | 0.931 | 0.928 | 0.770 | 1.045 | 1.248 | 1.448 | 1.656 |
| FBgn0085408 | 0.919 | 0.939 | 0.991 | 1.013 | 1.019 | 1.127 | 0.965 | 0.968 | 1.004 | 1.030 |
| FBgn0086604 | 0.975 | 0.996 | 1.080 | 1.102 | 1.158 | 1.210 | 1.071 | 1.153 | 1.217 | 1.390 |

| Supplemental Table 3G. Values of R Squared and Fold Changes for Genes Found to Behave Linearly at Days 2, 10 and 20 Post-Irradiation. (Analysis with all lowest dose discluded.) | | | | | |  |  |  |  |  |  |  |  |  |  |  |  |  |
| --- | --- | --- | --- | --- | --- | --- | --- | --- | --- | --- | --- | --- | --- | --- | --- | --- | --- | --- |
|  |  |  |  |  |  |  |  |  |  |  |  |  |  |  |  |  |  |  |
| Flybase ID | R Squared Value Day2 | R Squared Value Day10 | R Squared Value Day20 |  | Day 2 Fold Change 1000R | Day 2 Fold Change 5000R | Day 2 Fold Change 10000R | Day 2 Fold Change 20000R |  | Day10 Fold Change 1000R | Day10 Fold Change 5000R | Day10 Fold Change 10000R | Day10 Fold Change 20000R |  | Day 20 Fold Change 1000R | Day 20 Fold Change 5000R | Day 20 Fold Change 10000R | Day 20 Fold Change 20000R |
| FBgn0011774 | 0.969 | 0.970 | 0.960 |  | 0.954 | 1.315 | 1.428 | 1.933 |  | 1.035 | 1.228 | 1.975 | 2.650 |  | 1.096 | 1.113 | 1.871 | 2.722 |
| FBgn0024912 | 0.947 | 0.990 | 0.987 |  | 1.391 | 1.592 | 1.717 | 1.904 |  | 1.051 | 1.549 | 1.898 | 2.703 |  | 1.400 | 1.985 | 2.959 | 4.117 |
| FBgn0027101 | 0.906 | 0.914 | 0.914 |  | 1.100 | 1.091 | 1.057 | 0.855 |  | 0.882 | 0.854 | 1.081 | 1.808 |  | 2.271 | 2.196 | 2.979 | 3.538 |
| FBgn0030189 | 0.938 | 0.972 | 0.992 |  | 0.777 | 0.848 | 1.010 | 1.108 |  | 0.992 | 1.010 | 1.195 | 1.489 |  | 1.168 | 1.303 | 1.424 | 1.817 |
| FBgn0032393 | 0.974 | 0.981 | 0.947 |  | 1.135 | 1.347 | 1.423 | 1.734 |  | 1.130 | 1.699 | 2.025 | 2.818 |  | 1.020 | 1.398 | 2.248 | 2.817 |
| FBgn0033926 | 0.931 | 0.987 | 0.921 |  | 1.108 | 1.534 | 1.668 | 3.537 |  | 1.533 | 1.747 | 2.548 | 3.675 |  | 1.163 | 1.042 | 1.610 | 2.730 |
| FBgn0033927 | 0.940 | 0.981 | 0.936 |  | 1.217 | 1.469 | 1.907 | 4.087 |  | 1.570 | 1.781 | 2.694 | 3.778 |  | 1.156 | 1.082 | 1.636 | 2.703 |
| FBgn0034184 | 0.952 | 0.988 | 0.982 |  | 1.033 | 1.036 | 1.039 | 1.055 |  | 1.031 | 1.072 | 1.137 | 1.313 |  | 0.948 | 1.018 | 1.052 | 1.222 |
| FBgn0036290 | 0.963 | 0.996 | 0.996 |  | 0.797 | 0.975 | 1.110 | 1.303 |  | 0.797 | 1.069 | 1.496 | 2.118 |  | 1.438 | 1.834 | 2.689 | 4.152 |
| FBgn0037020 | 0.986 | 0.934 | 0.954 |  | 0.938 | 0.953 | 1.001 | 1.089 |  | 0.927 | 1.089 | 1.107 | 1.298 |  | 0.996 | 0.993 | 1.100 | 1.229 |
| FBgn0037850 | 0.941 | 0.959 | 0.996 |  | 1.320 | 2.346 | 2.565 | 3.692 |  | 1.263 | 2.117 | 2.280 | 3.514 |  | 1.411 | 1.769 | 2.408 | 3.722 |
| FBgn0046763 | 0.943 | 0.998 | 0.996 |  | 0.940 | 0.958 | 1.078 | 1.159 |  | 0.875 | 1.061 | 1.225 | 1.614 |  | 1.436 | 1.584 | 1.813 | 2.153 |
| FBgn0051864 | 0.978 | 0.965 | 0.976 |  | 1.096 | 1.138 | 1.421 | 1.832 |  | 1.072 | 1.596 | 1.711 | 2.806 |  | 1.066 | 1.287 | 2.116 | 2.943 |

| Supplemental Table 4A. Gene Ontologies Overrepresented in Genes Found to Behave Linearly at Day 2 Post-Irradiation. (Analysis with lowest dose discluded.) | | | | |
| --- | --- | --- | --- | --- |
|  |  |  |  |  |
| GO | Count | Total | P Value | GO Name |
| GO:0008605 | 13 | 23 | 5.14E-13 | xprotein kinase CK2 regulator activity |
| GO:0005956 | 13 | 23 | 5.14E-13 | protein kinase CK2 complex |
| GO:0043549 | 12 | 35 | 4.92E-09 | regulation of kinase activity |
| GO:0051338 | 12 | 35 | 4.92E-09 | regulation of transferase activity |
| GO:0045859 | 12 | 35 | 4.92E-09 | regulation of protein kinase activity |
| GO:0019887 | 13 | 53 | 6.60E-08 | protein kinase regulator activity |
| GO:0019207 | 13 | 55 | 9.35E-08 | kinase regulator activity |
| GO:0050790 | 12 | 57 | 1.63E-06 | regulation of catalytic activity |
| GO:0065009 | 13 | 70 | 1.72E-06 | regulation of a molecular function |
| GO:0007283 | 17 | 150 | 1.68E-05 | xspermatogenesis |
| GO:0048232 | 17 | 150 | 1.68E-05 | male gamete generation |
| GO:0022411 | 5 | 13 | 0.00115 | cellular component disassembly |

| Supplemental Table 4B. Gene Ontologies Overrepresented in Genes Found to Behave Linearly at Day 10 Post-Irradiation. (Analysis with lowest dose discluded.) | | | | |
| --- | --- | --- | --- | --- |
|  |  |  |  |  |
| GO | Count | Total | P Value | GO Name |
| GO:0003924 | 17 | 102 | 0.0028 | GTPase activity |
| GO:0016798 | 16 | 102 | 0.0118 | hydrolase activity, acting on glycosyl bonds |
| GO:0005102 | 19 | 136 | 0.0129 | receptor binding |
| GO:0004553 | 15 | 96 | 0.0129 | hydrolase activity, hydrolyzing O-glycosyl compounds |
| GO:0009055 | 21 | 166 | 0.0282 | electron carrier activity |
| GO:0005624 | 14 | 93 | 0.0334 | membrane fraction |
| GO:0005515 | 79 | 974 | 0.0334 | protein binding |
| GO:0000267 | 14 | 96 | 0.0411 | cell fraction |
| GO:0016740 | 70 | 851 | 0.0411 | transferase activity |
| GO:0008083 | 6 | 17 | 0.0411 | growth factor activity |
| GO:0005778 | 5 | 12 | 0.0427 | peroxisomal membrane |
| GO:0044438 | 5 | 12 | 0.0427 | microbody#microbody part |
| GO:0031903 | 5 | 12 | 0.0427 | microbody membrane |
| GO:0044439 | 5 | 12 | 0.0427 | peroxisomal part |
| GO:0016491 | 48 | 541 | 0.0499 | oxidoreductase activity |

| Supplemental Table 4C. Gene Ontologies Overrepresented in Genes Found to Behave Linearly at Day 20 Post-Irradiation. (Analysis with lowest dose discluded.) | | | | |
| --- | --- | --- | --- | --- |
|  |  |  |  |  |
| GO | Count | Total | P Value | GO Name |
| GO:0005887 | 16 | 169 | 0.0205 | integral to plasma membrane |
| GO:0031226 | 16 | 172 | 0.0205 | intrinsic to plasma membrane |
| GO:0000270 | 4 | 7 | 0.0205 | peptidoglycan metabolic process |
| GO:0042834 | 5 | 13 | 0.0205 | peptidoglycan binding |
| GO:0017143 | 4 | 9 | 0.0328 | insecticide metabolic process |
| GO:0006805 | 4 | 9 | 0.0328 | xenobiotic metabolic process |
| GO:0009404 | 4 | 9 | 0.0328 | toxin metabolic process |
| GO:0009410 | 4 | 9 | 0.0328 | response to xenobiotic stimulus |
| GO:0016020 | 70 | 1367 | 0.0355 | membrane |
| GO:0046903 | 15 | 172 | 0.0361 | secretion |
| GO:0019752 | 14 | 156 | 0.0361 | carboxylic acid metabolic process |
| GO:0006082 | 14 | 156 | 0.0361 | organic acid metabolic process |
| GO:0009056 | 15 | 174 | 0.037 | catabolic process |
| GO:0044459 | 22 | 305 | 0.0377 | plasma membrane part |
| GO:0016192 | 26 | 386 | 0.0377 | vesicle-mediated transport |
| GO:0006767 | 4 | 11 | 0.0377 | water-soluble vitamin metabolic process |
| GO:0009595 | 3 | 5 | 0.0377 | detection of biotic stimulus |
| GO:0016045 | 3 | 5 | 0.0377 | detection of bacterium |
| GO:0030425 | 4 | 12 | 0.0486 | dendrite |
| GO:0016021 | 36 | 608 | 0.0486 | integral to membrane |

| Supplemental Table 4D. Gene Ontologies Overrepresented in Genes Found to Behave Linearly at Days 2 and 10 Post-Irradiation. (Analysis with lowest dose discluded.) | | | | | |
| --- | --- | --- | --- | --- | --- |
|  |  |  |  |  |  |
| (none) |  |  |  |  |  |

| Supplemental Table 4E. Gene Ontologies Overrepresented in Genes Found to Behave Linearly at Days 10 and 20 Post-Irradiation. (Analysis with lowest dose discluded.) | | | | |
| --- | --- | --- | --- | --- |
|  |  |  |  |  |
| GO | Count | Total | P Value | GO Name |
| GO:0005778 | 3 | 12 | 0.0165 | peroxisomal membrane |
| GO:0044438 | 3 | 12 | 0.0165 | microbody#microbody part |
| GO:0044439 | 3 | 12 | 0.0165 | xperoxisomal part |
| GO:0031903 | 3 | 12 | 0.0165 | microbody membrane |
| GO:0007031 | 3 | 13 | 0.0171 | peroxisome organization and biogenesis |

| Supplemental Table 4F. Gene Ontologies Overrepresented in Genes Found to Behave Linearly at Days 2 and 20 Post-Irradiation. (Analysis with lowest dose discluded.) | | | | |
| --- | --- | --- | --- | --- |
|  |  |  |  |  |
| GO | Count | Total | P Value | GO Name |
| GO:0000723 | 2 | 10 | 0.0169 | telomere maintenance |
| GO:0032200 | 2 | 10 | 0.0169 | telomere organization and biogenesis |
| GO:0004003 | 2 | 20 | 0.0217 | ATP-dependent DNA helicase activity |
| GO:0016772 | 5 | 446 | 0.0217 | transferase activity, transferring phosphorus-containing groups |
| GO:0008094 | 2 | 29 | 0.0217 | DNA-dependent ATPase activity |
| GO:0043296 | 2 | 36 | 0.0217 | apicolateral plasma membrane#apical junction complex |
| GO:0003678 | 2 | 36 | 0.0217 | DNA helicase activity |
| GO:0016740 | 6 | 851 | 0.0217 | transferase activity |
| GO:0016327 | 2 | 40 | 0.0237 | apicolateral plasma membrane |
| GO:0005911 | 2 | 50 | 0.0302 | intercellular junction |
| GO:0006081 | 1 | 2 | 0.0302 | aldehyde metabolic process |
| GO:0004105 | 1 | 2 | 0.0302 | choline-phosphate cytidylyltransferase activity |
| GO:0031163 | 1 | 2 | 0.0302 | metallo-sulfur cluster assembly |
| GO:0004345 | 1 | 2 | 0.0302 | glucose-6-phosphate dehydrogenase activity |
| GO:0004123 | 1 | 2 | 0.0302 | cystathionine gamma-lyase activity |
| GO:0006625 | 1 | 2 | 0.0302 | protein targeting to peroxisome |
| GO:0016226 | 1 | 2 | 0.0302 | iron-sulfur cluster assembly |
| GO:0043574 | 1 | 2 | 0.0302 | peroxisomal transport |
| GO:0016337 | 2 | 59 | 0.0361 | cell-cell adhesion |
| GO:0017116 | 1 | 3 | 0.0415 | single-stranded DNA-dependent ATP-dependent DNA helicase activity |
| GO:0043142 | 1 | 3 | 0.0415 | single-stranded DNA-dependent ATPase activity |
| GO:0016779 | 2 | 68 | 0.0438 | nucleotidyltransferase activity |
| GO:0008026 | 2 | 70 | 0.0443 | ATP-dependent helicase activity |
| GO:0051186 | 2 | 75 | 0.0443 | cofactor metabolic process |
| GO:0030054 | 2 | 75 | 0.0443 | cell junction |
| GO:0006740 | 1 | 4 | 0.0443 | NADPH regeneration |
| GO:0009225 | 1 | 4 | 0.0443 | nucleotide-sugar metabolic process |
| GO:0006098 | 1 | 4 | 0.0443 | pentose-phosphate shunt |
| GO:0006739 | 1 | 4 | 0.0443 | NADP metabolic process |
| GO:0005923 | 1 | 4 | 0.0443 | apicolateral plasma membrane#apical junction complex#tight junction |
| GO:0016616 | 2 | 79 | 0.0471 | oxidoreductase activity, acting on the CH-OH group of donors, NAD or NADP as acceptor |

| Supplemental Table 4G. Gene Ontologies Overrepresented in Genes Found to Behave Linearly at Days 2, 10 and 20 Post-Irradiation. (Analysis with lowest dose discluded.) | | | | |
| --- | --- | --- | --- | --- |
|  |  |  |  |  |
| GO | Count | Total | P Value | GO Name |
| GO:0004123 | 1 | 2 | 0.0123 | cystathionine gamma-lyase activity |
| GO:0006625 | 1 | 2 | 0.0123 | protein targeting to peroxisome |
| GO:0006081 | 1 | 2 | 0.0123 | aldehyde metabolic process |
| GO:0016226 | 1 | 2 | 0.0123 | iron-sulfur cluster assembly |
| GO:0043574 | 1 | 2 | 0.0123 | xperoxisomal transport |
| GO:0031163 | 1 | 2 | 0.0123 | metallo-sulfur cluster assembly |
| GO:0004032 | 1 | 6 | 0.0323 | aldehyde reductase activity |
| GO:0016846 | 1 | 6 | 0.0323 | carbon-sulfur lyase activity |
| GO:0004033 | 1 | 8 | 0.0405 | aldo-keto reductase activity |
| GO:0004674 | 2 | 190 | 0.0412 | protein serine/threonine kinase activity |
| GO:0032200 | 1 | 10 | 0.0412 | telomere organization and biogenesis |
| GO:0000723 | 1 | 10 | 0.0412 | xtelomere maintenance |
| GO:0030178 | 1 | 11 | 0.0412 | xnegative regulation of Wnt receptor signaling pathway |
| GO:0044439 | 1 | 12 | 0.0412 | peroxisomal part |
| GO:0044438 | 1 | 12 | 0.0412 | microbody#microbody part |
| GO:0031903 | 1 | 12 | 0.0412 | microbody membrane |
| GO:0005778 | 1 | 12 | 0.0412 | peroxisomal membrane |
| GO:0007031 | 1 | 13 | 0.0429 | peroxisome organization and biogenesis |

| Supplemental Table 5A. Values of Spike Ratio and Fold Changes for Genes Found to Spike at Day 2 Post-Irradiation. (Analysis with all data included.) Spike ratio = (largest fold change)/(second largest fold change) | | | | | | |  |
| --- | --- | --- | --- | --- | --- | --- | --- |
|  |  |  |  |  |  |  |  |
| Flybase ID | Spike Ratio | Dose Causing Spike | Day 2 Fold Change 10R | Day 2 Fold Change 1000R | Day 2 Fold Change 50000R | Day 2 Fold Change 10000R | Day 2 Fold Change 20000R |
| FBgn0005391 | 6.018 | 10000R | 2.573 | 0.888 | 0.545 | 15.486 | 1.268 |
| FBgn0004045 | 15.787 | 10000R | 0.936 | 0.447 | 0.849 | 16.168 | 1.024 |
| FBgn0038191 | 5.379 | 1000R | 0.248 | 1.332 | 0.141 | 0.183 | 0.154 |
| FBgn0013672 | 7.952 | 20000R | 1.114 | 0.656 | 0.783 | 0.651 | 8.855 |
| FBgn0013678 | 7.046 | 20000R | 1.028 | 0.774 | 0.681 | 0.729 | 7.242 |
| FBgn0034152 | 6.826 | 20000R | 0.780 | 6.282 | 2.196 | 6.885 | 46.999 |
| FBgn0013676 | 6.461 | 20000R | 1.278 | 0.876 | 1.026 | 0.772 | 8.256 |
| FBgn0013674 | 6.025 | 20000R | 1.271 | 1.155 | 0.874 | 0.985 | 7.656 |
| FBgn0259968 | 5.361 | 20000R | 0.945 | 1.163 | 0.673 | 0.789 | 6.237 |
| FBgn0013675 | 13.108 | 20000R | 1.336 | 0.673 | 0.855 | 0.522 | 17.518 |
| FBgn0013745 | 7.176 | 5000R | 0.371 | 0.966 | 11.772 | 1.155 | 1.640 |
| FBgn0262099 | 6.609 | 5000R | 0.591 | 5.446 | 44.237 | 6.693 | 5.023 |

| Supplemental Table 5B. Values of Spike Ratio and Fold Changes for Genes Found to Spike at Day 10 Post-Irradiation. (Analysis with all data included.) Spike ratio = (largest fold change)/(second largest fold change) | | | | | | |  |
| --- | --- | --- | --- | --- | --- | --- | --- |
|  |  |  |  |  |  |  |  |
| Flybase ID | SpikeRatio | Dose Causing Spike | Day10 Fold Change 10R | Day10 Fold Change 1000R | Day10 Fold Change 5000R | Day10 Fold Change 10000R | Day10 Fold Change 20000R |
| FBgn0013679 | 9.785 | 20000R | 2.051 | 1.087 | 1.137 | 1.197 | 20.065 |
| FBgn0025740 | 9.617 | 20000R | 0.429 | 0.708 | 0.469 | 0.462 | 6.805 |
| FBgn0013672 | 9.472 | 20000R | 0.758 | 0.864 | 1.744 | 0.981 | 16.520 |
| FBgn0085364 | 9.183 | 20000R | 1.107 | 1.655 | 1.378 | 1.306 | 15.201 |
| FBgn0013676 | 8.690 | 20000R | 0.921 | 0.799 | 1.255 | 1.007 | 10.907 |
| FBgn0031111 | 8.280 | 10R | 13.211 | 1.114 | 1.485 | 1.011 | 1.596 |
| FBgn0013681 | 7.360 | 20000R | 1.370 | 0.709 | 1.617 | 0.388 | 11.902 |
| FBgn0000427 | 68.582 | 20000R | 164.035 | 1.051 | 1.101 | 1.066 | 2.392 |
| FBgn0259968 | 6.846 | 20000R | 1.012 | 1.200 | 1.482 | 1.239 | 10.143 |
| FBgn0041709 | 6.434 | 10R | 24.479 | 0.793 | 0.771 | 1.388 | 3.805 |
| FBgn0013686 | 6.416 | 20000R | 0.563 | 0.882 | 0.650 | 0.863 | 5.657 |
| FBgn0021738 | 6.346 | 20000R | 0.478 | 0.673 | 0.860 | 0.708 | 5.455 |
| FBgn0262972 | 6.089 | 20000R | 0.770 | 0.886 | 0.821 | 0.256 | 5.397 |
| FBgn0000357 | 52.726 | 10R | 76.612 | 0.932 | 1.159 | 1.218 | 1.453 |
| FBgn0039916 | 5.769 | 20000R | 0.351 | 0.749 | 1.016 | 0.939 | 5.862 |
| FBgn0037836 | 5.715 | 20000R | 0.869 | 1.050 | 1.071 | 0.741 | 6.122 |
| FBgn0036790 | 5.703 | 20000R | 0.355 | 0.705 | 0.575 | 0.469 | 4.018 |
| FBgn0036985 | 5.546 | 20000R | 0.568 | 1.104 | 0.578 | 0.905 | 6.126 |
| FBgn0032946 | 5.427 | 20000R | 1.689 | 1.590 | 1.541 | 1.264 | 9.167 |
| FBgn0086782 | 5.393 | 20000R | 0.684 | 0.802 | 0.592 | 0.965 | 5.205 |
| FBgn0052350 | 5.357 | 20000R | 0.610 | 0.803 | 0.900 | 1.126 | 6.034 |
| FBgn0013675 | 5.229 | 20000R | 0.857 | 0.937 | 2.220 | 0.813 | 11.611 |
| FBgn0039925 | 35.389 | 20000R | 0.305 | 0.413 | 0.446 | 0.518 | 18.324 |
| FBgn0013680 | 28.243 | 20000R | 1.126 | 1.065 | 1.218 | 0.802 | 34.413 |
| FBgn0053855 | 21.401 | 20000R | 2.643 | 0.651 | 0.897 | 2.320 | 56.555 |
| FBgn0053858 | 21.234 | 20000R | 2.791 | 0.722 | 0.928 | 2.390 | 59.260 |
| FBgn0053810 | 20.386 | 20000R | 2.449 | 0.632 | 0.869 | 1.988 | 49.934 |
| FBgn0053831 | 19.800 | 20000R | 2.574 | 0.582 | 0.855 | 1.974 | 50.972 |
| FBgn0053837 | 19.797 | 20000R | 2.442 | 0.651 | 0.838 | 1.921 | 48.348 |
| FBgn0053822 | 19.649 | 20000R | 2.621 | 0.607 | 0.794 | 2.017 | 51.495 |
| FBgn0053816 | 19.527 | 20000R | 2.529 | 0.583 | 0.874 | 1.958 | 49.377 |
| FBgn0053861 | 19.524 | 20000R | 3.118 | 0.737 | 0.932 | 2.407 | 60.868 |
| FBgn0053819 | 19.465 | 20000R | 2.609 | 0.640 | 0.853 | 2.004 | 50.776 |
| FBgn0053807 | 19.436 | 20000R | 2.337 | 0.593 | 0.804 | 1.836 | 45.416 |
| FBgn0053828 | 19.260 | 20000R | 2.519 | 0.617 | 0.848 | 1.931 | 48.524 |
| FBgn0053825 | 19.255 | 20000R | 2.568 | 0.628 | 0.829 | 1.966 | 49.449 |
| FBgn0053843 | 19.255 | 20000R | 2.712 | 0.715 | 0.846 | 1.989 | 52.223 |
| FBgn0053864 | 19.048 | 20000R | 2.565 | 0.597 | 0.861 | 1.925 | 48.861 |
| FBgn0053852 | 19.034 | 20000R | 2.534 | 0.624 | 0.825 | 1.921 | 48.235 |
| FBgn0053813 | 18.977 | 20000R | 2.776 | 0.680 | 0.875 | 2.202 | 52.672 |
| FBgn0053846 | 18.904 | 20000R | 2.613 | 0.603 | 0.845 | 1.988 | 49.390 |
| FBgn0051617 | 18.804 | 20000R | 2.440 | 0.540 | 0.811 | 1.893 | 45.879 |
| FBgn0053801 | 18.653 | 20000R | 2.549 | 0.612 | 0.815 | 1.928 | 47.551 |
| FBgn0053849 | 18.323 | 20000R | 2.771 | 0.623 | 0.858 | 1.975 | 50.782 |
| FBgn0053840 | 17.922 | 20000R | 2.677 | 0.660 | 0.830 | 1.901 | 47.978 |
| FBgn0053804 | 17.761 | 20000R | 2.622 | 0.601 | 0.851 | 1.913 | 46.573 |
| FBgn0013678 | 15.720 | 20000R | 0.806 | 0.784 | 1.009 | 0.988 | 15.862 |
| FBgn0000355 | 141.389 | 10R | 130.305 | 0.922 | 0.687 | 0.127 | 0.165 |
| FBgn0013685 | 14.707 | 20000R | 1.367 | 0.670 | 0.853 | 0.396 | 20.105 |
| FBgn0052602 | 13.143 | 10R | 20.045 | 0.874 | 0.626 | 0.624 | 1.525 |
| FBgn0052580 | 124.695 | 20000R | 0.208 | 0.620 | 0.240 | 0.751 | 93.597 |
| FBgn0013674 | 12.216 | 20000R | 0.910 | 0.963 | 0.947 | 1.071 | 13.085 |
| FBgn0262952 | 12.152 | 20000R | 1.639 | 1.050 | 0.703 | 1.111 | 19.912 |
| FBgn0013684 | 11.816 | 20000R | 1.266 | 0.696 | 0.642 | 0.729 | 14.954 |

| Supplemental Table 5C. Values of Spike Ratio and Fold Changes for Genes Found to Spike at Day 20 Post-Irradiation. (Analysis with all data included.) Spike ratio = (largest fold change)/(second largest fold change) | | | | | | |  |
| --- | --- | --- | --- | --- | --- | --- | --- |
|  |  |  |  |  |  |  |  |
| Flybase ID | SpikeRatio | Dose Causing Spike | Day 20 Fold Change 10R | Day 20 Fold Change 1000R | Day 20 Fold Change 5000R | Day 20 Fold Change 10000R | Day 20 Fold Change 20000R |
| FBgn0053222 | 9.209 | 5000R | 1.164 | 1.180 | 10.869 | 0.146 | 0.026 |
| FBgn0085364 | 8.050 | 20000R | 1.094 | 1.610 | 0.895 | 1.364 | 12.963 |
| FBgn0039480 | 6.902 | 20000R | 0.158 | 0.105 | 0.090 | 0.173 | 1.193 |
| FBgn0040637 | 6.835 | 20000R | 1.457 | 1.511 | 2.097 | NA | 14.334 |

| Supplemental Table 5D. Values of Spike Ratio and Fold Changes for Genes Found to Spike at Days 2 and 10 Post-Irradiation. (Analysis with all data included.) Spike ratio = (largest fold change)/(second largest fold change) | | | | | | | | |  |  |  |  |  |  |
| --- | --- | --- | --- | --- | --- | --- | --- | --- | --- | --- | --- | --- | --- | --- |
|  |  |  |  |  |  |  |  |  |  |  |  |  |  |  |
| FlyBase ID | Spike Ratio Day2 | Dose Causing Spike Day2 | SpikeRatio Day10 | Dose Causing Spike Day10 | Day 2 Fold Change 10R | Day 2 Fold Change 1000R | Day 2 Fold Change 50000R | Day 2 Fold Change 10000R | Day 2 Fold Change 20000R | Day10 Fold Change 10R | Day10 Fold Change 1000R | Day10 Fold Change 5000R | Day10 Fold Change 10000R | Day10 Fold Change 20000R |
| FBgn0013672 | 7.952 | 20000R | 9.472 | 20000R | 1.114 | 0.656 | 0.783 | 0.651 | 8.855 | 0.758 | 0.864 | 1.744 | 0.981 | 16.520 |
| FBgn0013678 | 7.046 | 20000R | 8.690 | 20000R | 1.028 | 0.774 | 0.681 | 0.729 | 7.242 | 0.921 | 0.799 | 1.255 | 1.007 | 10.907 |
| FBgn0013676 | 6.461 | 20000R | 6.846 | 20000R | 1.278 | 0.876 | 1.026 | 0.772 | 8.256 | 1.012 | 1.200 | 1.482 | 1.239 | 10.143 |
| FBgn0013674 | 6.025 | 20000R | 5.229 | 20000R | 1.271 | 1.155 | 0.874 | 0.985 | 7.656 | 0.857 | 0.937 | 2.220 | 0.813 | 11.611 |
| FBgn0259968 | 5.361 | 20000R | 15.720 | 20000R | 0.945 | 1.163 | 0.673 | 0.789 | 6.237 | 0.806 | 0.784 | 1.009 | 0.988 | 15.862 |
| FBgn0013675 | 13.108 | 20000R | 12.216 | 20000R | 1.336 | 0.673 | 0.855 | 0.522 | 17.518 | 0.910 | 0.963 | 0.947 | 1.071 | 13.085 |

| Supplemental Table 5E. Values of Spike Ratio and Fold Changes for Genes Found to Spike at Days 10 and 20 Post-Irradiation. (Analysis with all data included.) Spike ratio = (largest fold change)/(second largest fold change) | | | | | | | |  |  |  |  |  |  |  |
| --- | --- | --- | --- | --- | --- | --- | --- | --- | --- | --- | --- | --- | --- | --- |
|  |  |  |  |  |  |  |  |  |  |  |  |  |  |  |
| Flybase ID | SpikeRatio Day 10 | Dose Causing Spike Day10 | SpikeRatio Day 20 | Dose Causing Spike Day20 | Day10 Fold Change 10R | Day10 Fold Change 1000R | Day10 Fold Change 5000R | Day10 Fold Change 10000R | Day10 Fold Change 20000R | Day 20 Fold Change 10R | Day 20 Fold Change 1000R | Day 20 Fold Change 5000R | Day 20 Fold Change 10000R | Day 20 Fold Change 20000R |
| FBgn0085364 | 9.183 | 20000R | 8.050 | 20000R | 1.107 | 1.655 | 1.378 | 1.306 | 15.201 | 1.094 | 1.610 | 0.895 | 1.364 | 12.963 |

| Supplemental Table 5F. Values of Spike Ratio and Fold Changes for Genes Found to Spike at Days 2 and 20 Post-Irradiation. (Analysis with all data included.) Spike ratio = (largest fold change)/(second largest fold change) | | | | | | |
| --- | --- | --- | --- | --- | --- | --- |
|  |  |  |  |  |  |  |
| (none) |  |  |  |  |  |  |

| Supplemental Table 5G. Values of Spike Ratio and Fold Changes for Genes Found to Spike at Days 2, 10 and 20 Post-Irradiation. (Analysis with all data included.) Spike ratio = (largest fold change)/(second largest fold change) | | | | |
| --- | --- | --- | --- | --- |
|  |  |  |  |  |
| (none) |  |  |  |  |

| Supplemental Table 6A. Values of Spike Ratio and Fold Changes for Genes Found to Spike at Day 2 Post-Irradiation. (Analysis with lowest dose discluded.) Spike ratio = (largest fold change)/(second largest fold change) | | | | | |  |
| --- | --- | --- | --- | --- | --- | --- |
|  |  |  |  |  |  |  |
| Flybase ID | SpikeRatio | Dose Causing Spike | Day 2 Fold Change 1000R | Day 2 Fold Change 5000R | Day 2 Fold Change 10000R | Day 2 Fold Change 20000R |
| FBgn0013678 | 9.356 | 20000R | 0.774 | 0.681 | 0.729 | 7.242 |
| FBgn0013680 | 9.132 | 20000R | 0.563 | 0.683 | 0.420 | 6.233 |
| FBgn0013676 | 8.044 | 20000R | 0.876 | 1.026 | 0.772 | 8.256 |
| FBgn0013673 | 7.347 | 20000R | 0.466 | 0.685 | 0.372 | 5.031 |
| FBgn0013683 | 7.239 | 20000R | 0.944 | 0.695 | 0.318 | 6.832 |
| FBgn0013745 | 7.176 | 5000R | 0.966 | 11.772 | 1.155 | 1.640 |
| FBgn0034152 | 6.826 | 20000R | 6.282 | 2.196 | 6.885 | 46.999 |
| FBgn0013674 | 6.631 | 20000R | 1.155 | 0.874 | 0.985 | 7.656 |
| FBgn0262099 | 6.609 | 5000R | 5.446 | 44.237 | 6.693 | 5.023 |
| FBgn0013681 | 6.558 | 20000R | 0.798 | 0.916 | 0.515 | 6.005 |
| FBgn0013684 | 6.476 | 20000R | 0.788 | 0.633 | 0.788 | 5.102 |
| FBgn0013679 | 5.956 | 20000R | 1.004 | 0.879 | 0.867 | 5.981 |
| FBgn0085364 | 5.415 | 20000R | 1.298 | 0.718 | 0.916 | 7.026 |
| FBgn0259968 | 5.361 | 20000R | 1.163 | 0.673 | 0.789 | 6.237 |
| FBgn0013685 | 24.186 | 20000R | 0.338 | 0.246 | 0.237 | 8.163 |
| FBgn0013675 | 20.490 | 10000R | 0.673 | 0.855 | 0.522 | 17.518 |
| FBgn0004045 | 15.787 | 10000R | 0.447 | 0.849 | 16.168 | 1.024 |
| FBgn0013672 | 11.306 | 20000R | 0.656 | 0.783 | 0.651 | 8.855 |

| Supplemental Table 6B. Values of Spike Ratio and Fold Changes for Genes Found to Spike at Day 10 Post-Irradiation. (Analysis with lowest dose discluded.) Spike ratio = (largest fold change)/(second largest fold change) | | | | | | |
| --- | --- | --- | --- | --- | --- | --- |
|  |  |  |  |  |  |  |
| Flybase ID | Spike Ratio | Dose Causing Spike | Day 10 Fold Change 1000R | Day 10 Fold Change 5000R | Day 10 Fold Change 10000R | Day 10 Fold Change 20000R |
| FBgn0004045 | 87.528 | 10000R | 1.684 | 2.465 | 515.653 | 5.891 |
| FBgn0260780 | 7.925 | 10000R | 1.245 | 1.084 | 19.861 | 2.506 |
| FBgn0002962 | 7.160 | 10000R | 1.413 | 2.257 | 16.159 | 1.356 |
| FBgn0003023 | 5.911 | 10000R | 0.741 | 0.755 | 8.156 | 1.380 |
| FBgn0004047 | 34.886 | 10000R | 0.937 | 1.088 | 37.960 | 1.078 |
| FBgn0004649 | 19.854 | 10000R | 1.338 | 0.734 | 26.571 | 0.523 |
| FBgn0005391 | 158.647 | 10000R | 1.399 | 1.805 | 391.716 | 2.469 |
| FBgn0025740 | 9.617 | 20000R | 0.708 | 0.469 | 0.462 | 6.805 |
| FBgn0013672 | 9.472 | 20000R | 0.864 | 1.744 | 0.981 | 16.520 |
| FBgn0085364 | 9.183 | 20000R | 1.655 | 1.378 | 1.306 | 15.201 |
| FBgn0013676 | 8.690 | 20000R | 0.799 | 1.255 | 1.007 | 10.907 |
| FBgn0013681 | 7.360 | 20000R | 0.709 | 1.617 | 0.388 | 11.902 |
| FBgn0259968 | 6.846 | 20000R | 1.200 | 1.482 | 1.239 | 10.143 |
| FBgn0013686 | 6.416 | 20000R | 0.882 | 0.650 | 0.863 | 5.657 |
| FBgn0262972 | 6.089 | 20000R | 0.886 | 0.821 | 0.256 | 5.397 |
| FBgn0032946 | 5.765 | 20000R | 1.590 | 1.541 | 1.264 | 9.167 |
| FBgn0037836 | 5.715 | 20000R | 1.050 | 1.071 | 0.741 | 6.122 |
| FBgn0036790 | 5.703 | 20000R | 0.705 | 0.575 | 0.469 | 4.018 |
| FBgn0086782 | 5.393 | 20000R | 0.802 | 0.592 | 0.965 | 5.205 |
| FBgn0052350 | 5.357 | 20000R | 0.803 | 0.900 | 1.126 | 6.034 |
| FBgn0013675 | 5.229 | 20000R | 0.937 | 2.220 | 0.813 | 11.611 |
| FBgn0039925 | 35.389 | 20000R | 0.413 | 0.446 | 0.518 | 18.324 |
| FBgn0013680 | 28.243 | 20000R | 1.065 | 1.218 | 0.802 | 34.413 |
| FBgn0053843 | 26.253 | 20000R | 0.715 | 0.846 | 1.989 | 52.223 |
| FBgn0053831 | 25.818 | 20000R | 0.582 | 0.855 | 1.974 | 50.972 |
| FBgn0053849 | 25.717 | 20000R | 0.623 | 0.858 | 1.975 | 50.782 |
| FBgn0053822 | 25.530 | 20000R | 0.607 | 0.794 | 2.017 | 51.495 |
| FBgn0053864 | 25.387 | 20000R | 0.597 | 0.861 | 1.925 | 48.861 |
| FBgn0053819 | 25.332 | 20000R | 0.640 | 0.853 | 2.004 | 50.776 |
| FBgn0053861 | 25.293 | 20000R | 0.737 | 0.932 | 2.407 | 60.868 |
| FBgn0053840 | 25.241 | 20000R | 0.660 | 0.830 | 1.901 | 47.978 |
| FBgn0053816 | 25.222 | 20000R | 0.583 | 0.874 | 1.958 | 49.377 |
| FBgn0053837 | 25.174 | 20000R | 0.651 | 0.838 | 1.921 | 48.348 |
| FBgn0053825 | 25.150 | 20000R | 0.628 | 0.829 | 1.966 | 49.449 |
| FBgn0053828 | 25.132 | 20000R | 0.617 | 0.848 | 1.931 | 48.524 |
| FBgn0053810 | 25.121 | 20000R | 0.632 | 0.869 | 1.988 | 49.934 |
| FBgn0053852 | 25.107 | 20000R | 0.624 | 0.825 | 1.921 | 48.235 |
| FBgn0053846 | 24.838 | 20000R | 0.603 | 0.845 | 1.988 | 49.390 |
| FBgn0053858 | 24.791 | 20000R | 0.722 | 0.928 | 2.390 | 59.260 |
| FBgn0053807 | 24.743 | 20000R | 0.593 | 0.804 | 1.836 | 45.416 |
| FBgn0053801 | 24.666 | 20000R | 0.612 | 0.815 | 1.928 | 47.551 |
| FBgn0053855 | 24.373 | 20000R | 0.651 | 0.897 | 2.320 | 56.555 |
| FBgn0053804 | 24.341 | 20000R | 0.601 | 0.851 | 1.913 | 46.573 |
| FBgn0051617 | 24.233 | 20000R | 0.540 | 0.811 | 1.893 | 45.879 |
| FBgn0053813 | 23.919 | 20000R | 0.680 | 0.875 | 2.202 | 52.672 |
| FBgn0013684 | 20.510 | 20000R | 0.696 | 0.642 | 0.729 | 14.954 |
| FBgn0262952 | 17.927 | 20000R | 1.050 | 0.703 | 1.111 | 19.912 |
| FBgn0013679 | 16.768 | 20000R | 1.087 | 1.137 | 1.197 | 20.065 |
| FBgn0013678 | 15.720 | 20000R | 0.784 | 1.009 | 0.988 | 15.862 |
| FBgn0052580 | 124.695 | 20000R | 0.620 | 0.240 | 0.751 | 93.597 |
| FBgn0013674 | 12.216 | 20000R | 0.963 | 0.947 | 1.071 | 13.085 |

| Supplemental Table 6C. Values of Spike Ratio and Fold Changes for Genes Found to Spike at Day 20 Post-Irradiation. (Analysis with lowest dose discluded.) Spike ratio = (largest fold change)/(second largest fold change) | | | | | | |
| --- | --- | --- | --- | --- | --- | --- |
|  |  |  |  |  |  |  |
| Flybase ID | Spike Ratio | Dose Causing Spike | Day 20 Fold Change 1000R | Day 20 Fold Change 5000R | Day 20 Fold Change 10000R | Day 20 Fold Change 20000R |
| FBgn0053222 | 9.209 | 5000R | 1.180 | 10.869 | 0.146 | 0.026 |
| FBgn0085364 | 8.050 | 20000R | 1.610 | 0.895 | 1.364 | 12.963 |
| FBgn0039480 | 6.902 | 20000R | 0.105 | 0.090 | 0.173 | 1.193 |

| Supplemental Table 6D. Values of Spike Ratio and Fold Changes for Genes Found to Spike at Days 2 and 10 Post-Irradiation. (Analysis with lowest dose discluded.) Spike ratio = (largest fold change)/(second largest fold change) | | | | | | |  |  |  | | | |
| --- | --- | --- | --- | --- | --- | --- | --- | --- | --- | --- | --- | --- |
|  |  |  |  |  |  |  |  |  |  |  |  |  |
| Flybase ID | Spike Ratio Day2 | Dose Causing Spike Day2 | Spike Ratio Day10 | Dose Causing Spike Day10 | Day 2 Fold Change 1000R | Day 2 Fold Change 5000R | Day 2 Fold Change 10000R | Day 2 Fold Change 20000R | Day 10 Fold Change 1000R | Day 10 Fold Change 5000R | Day 10 Fold Change 10000R | Day 10 Fold Change 20000R |
| FBgn0004045 | 15.787 | 10000R | 87.528 | 10000R | 0.447 | 0.849 | 16.168 | 1.024 | 1.684 | 2.465 | 515.653 | 5.891 |
| FBgn0013672 | 11.306 | 20000R | 9.472 | 20000R | 0.656 | 0.783 | 0.651 | 8.855 | 0.864 | 1.744 | 0.981 | 16.520 |
| FBgn0013674 | 6.631 | 20000R | 12.216 | 20000R | 1.155 | 0.874 | 0.985 | 7.656 | 0.963 | 0.947 | 1.071 | 13.085 |
| FBgn0013675 | 20.490 | 20000R | 5.229 | 20000R | 0.673 | 0.855 | 0.522 | 17.518 | 0.937 | 2.220 | 0.813 | 11.611 |
| FBgn0013676 | 8.044 | 20000R | 8.690 | 20000R | 0.876 | 1.026 | 0.772 | 8.256 | 0.799 | 1.255 | 1.007 | 10.907 |
| FBgn0013678 | 9.356 | 20000R | 15.720 | 20000R | 0.774 | 0.681 | 0.729 | 7.242 | 0.784 | 1.009 | 0.988 | 15.862 |
| FBgn0013679 | 5.956 | 20000R | 16.768 | 20000R | 1.004 | 0.879 | 0.867 | 5.981 | 1.087 | 1.137 | 1.197 | 20.065 |
| FBgn0013680 | 9.132 | 20000R | 28.243 | 20000R | 0.563 | 0.683 | 0.420 | 6.233 | 1.065 | 1.218 | 0.802 | 34.413 |
| FBgn0013681 | 6.558 | 20000R | 7.360 | 20000R | 0.798 | 0.916 | 0.515 | 6.005 | 0.709 | 1.617 | 0.388 | 11.902 |
| FBgn0013684 | 6.476 | 20000R | 20.510 | 20000R | 0.788 | 0.633 | 0.788 | 5.102 | 0.696 | 0.642 | 0.729 | 14.954 |
| FBgn0085364 | 5.415 | 20000R | 9.183 | 20000R | 1.298 | 0.718 | 0.916 | 7.026 | 1.655 | 1.378 | 1.306 | 15.201 |
| FBgn0259968 | 5.361 | 20000R | 6.846 | 20000R | 1.163 | 0.673 | 0.789 | 6.237 | 1.200 | 1.482 | 1.239 | 10.143 |

| Supplemental Table 6E. Values of Spike Ratio and Fold Changes for Genes Found to Spike at Days 10 and 20 Post-Irradiation. (Analysis with lowest dose discluded.) Spike ratio = (largest fold change)/(second largest fold change) | | | | | | | |  |  |  |  |  |
| --- | --- | --- | --- | --- | --- | --- | --- | --- | --- | --- | --- | --- |
|  |  |  |  |  |  |  |  |  |  |  |  |  |
|  | Spike Ratio Day 10 | Dose Causing Spike Day 10 | Spike Ratio Day 20 | Dose Causing Spike Day 20 | Day 10 Fold Change 1000R | Day 10 Fold Change 5000R | Day 10 Fold Change 10000R | Day 10 Fold Change 20000R | Day 20 Fold Change 1000R | Day 20 Fold Change 5000R | Day 20 Fold Change 10000R | Day 20 Fold Change 20000R |
| FBgn0085364 | 9.183 | 20000R | 8.050 | 20000R | 1.655 | 1.378 | 1.306 | 15.201 | 1.610 | 0.895 | 1.364 | 12.963 |

| Supplemental Table 6F. Values of Spike Ratio and Fold Changes for Genes Found to Spike at Days 2 and 20 Post-Irradiation. (Analysis with lowest dose discluded.) Spike ratio = (largest fold change)/(second largest fold change) | | | | | | | |  |  |  |  |  |
| --- | --- | --- | --- | --- | --- | --- | --- | --- | --- | --- | --- | --- |
|  |  |  |  |  |  |  |  |  |  |  |  |  |
| Flybase ID | Spike Ratio Day 2 | Dose Causing Spike Day 2 | Spike Ratio Day 20 | Dose Causing Spike Day20 | Day 2 Fold Change 1000R | Day 2 Fold Change 5000R | Day 2 Fold Change 10000R | Day 2 Fold Change 20000R | Day 20 Fold Change 1000R | Day 20 Fold Change 5000R | Day 20 Fold Change 10000R | Day 20 Fold Change 20000R |
| FBgn0085364 | 5.415 | 20000R | 8.050 | 20000R | 1.298 | 0.718 | 0.916 | 7.026 | 1.610 | 0.895 | 1.364 | 12.963 |

| Supplemental Table 6G. Values of Spike Ratio and Fold Changes (FC) for Genes Found to Spike at Days 2, 10 and 20 Post-Irradiation. (Analysis with lowest dose discluded.) Spike ratio = (largest fold change)/(second largest fold change) | | | | | | | | |  |  |  |  |  |  |  |  |  |  |
| --- | --- | --- | --- | --- | --- | --- | --- | --- | --- | --- | --- | --- | --- | --- | --- | --- | --- | --- |
|  |  |  |  |  |  |  |  |  |  |  |  |  |  |  |  |  |  |  |
| Flybase ID | Spike Ratio Day 2 | Dose Causing Spike Day 2 | SpikeRatio Day 10 | Dose Causing Spike Day 10 | Spike Ratio Day 20 | Dose Causing Spike Day 20 | Day 2 Fold Change 1000R | Day 2 FC 5000R | Day 2 FC 10000R | Day 2 FC 20000R | Day 10 FC 1000R | Day 10 FC 5000R | Day 10 FC 10000R | Day 10 FC 20000R | Day 20 FC 1000R | Day 20 FC 5000R | Day 20 FC 10000R | Day 20 FC 20000R |
| FBgn0085364 | 5.415 | 20000R | 9.183 | 20000R | 8.050 | 20000R | 1.298 | 0.718 | 0.916 | 7.026 | 1.655 | 1.378 | 1.306 | 15.201 | 1.610 | 0.895 | 1.364 | 12.963 |

| Supplemental Table 7A. Overrepresented Gene Ontologies in Genes Found to Spike at Day 2 Post-Irradiation at Dose 5000 R. (Analysis with all data included.) | | | | |
| --- | --- | --- | --- | --- |
|  |  |  |  |  |
| GO | Count | Total | P Value | GO Name |
| GO:0045434 | 1 | 7 | 0.00511 | negative regulation of female receptivity, post-mating |
| GO:0007621 | 1 | 8 | 0.00511 | negative regulation of female receptivity |
| GO:0048521 | 1 | 8 | 0.00511 | negative regulation of behavior |
| GO:0046008 | 1 | 10 | 0.00511 | regulation of female receptivity, post-mating |
| GO:0045924 | 1 | 16 | 0.00511 | regulation of female receptivity |
| GO:0060180 | 1 | 16 | 0.00511 | mating behavior#female mating behavior |
| GO:0060181 | 1 | 16 | 0.00511 | female receptivity |
| GO:0045297 | 1 | 18 | 0.00511 | post-mating behavior |
| GO:0050795 | 1 | 18 | 0.00511 | regulation of behavior |
| GO:0005179 | 1 | 47 | 0.012 | hormone activity |
| GO:0033057 | 1 | 69 | 0.0139 | reproductive behavior in a multicellular organism |
| GO:0032504 | 1 | 81 | 0.0139 | multicellular organism reproduction |
| GO:0048609 | 1 | 81 | 0.0139 | multicellular organism reproduction#reproductive process in a multicellular organism |
| GO:0007617 | 1 | 82 | 0.0139 | mating behavior |
| GO:0051705 | 1 | 82 | 0.0139 | behavioral interaction between organisms |
| GO:0019098 | 1 | 87 | 0.0139 | reproductive behavior |
| GO:0007618 | 1 | 94 | 0.0141 | mating |
| GO:0005102 | 1 | 136 | 0.0193 | receptor binding |
| GO:0022414 | 1 | 203 | 0.0271 | reproduction#reproductive process |
| GO:0051704 | 1 | 212 | 0.0271 | multi-organism process |

| Supplemental Table 7B. Overrepresented Gene Ontologies in Genes Found to Spike at Day 2 Post-Irradiation at Dose 10,000 R. (Analysis with all data included.) | | | | |
| --- | --- | --- | --- | --- |
|  |  |  |  |  |
| GO | Count | Total | P Value | GO Name |
| GO:0007296 | 2 | 8 | 1.81E-05 | vitellogenesis |
| GO:0007548 | 2 | 57 | 0.000515 | sex differentiation |
| GO:0003006 | 2 | 77 | 0.00063 | reproductive developmental process |
| GO:0007028 | 2 | 109 | 0.000951 | cytoplasm organization and biogenesis |
| GO:0005811 | 2 | 129 | 0.00107 | lipid particle |
| GO:0022414 | 2 | 203 | 0.00221 | reproduction#reproductive process |
| GO:0016590 | 1 | 6 | 0.00584 | ACF complex |
| GO:0005678 | 1 | 9 | 0.00767 | chromatin assembly complex |
| GO:0007292 | 2 | 528 | 0.00999 | female gamete generation |
| GO:0007276 | 2 | 704 | 0.0156 | gamete generation |
| GO:0019953 | 2 | 730 | 0.0156 | sexual reproduction |
| GO:0016585 | 1 | 33 | 0.0187 | chromatin remodeling complex |

| Supplemental Table 7C. Overrepresented Gene Ontologies in Genes Found to Spike at Day 10 Post-Irradiation at Dose 10,000 R. (Analysis with all data included.) | | | | |
| --- | --- | --- | --- | --- |
|  |  |  |  |  |
| GO | Count | Total | P Value | GO Name |
| GO:0005213 | 2 | 9 | 8.52E-05 | structural constituent of chorion |
| GO:0007306 | 2 | 64 | 0.00229 | ovarian follicle cell development#chorion-containing eggshell formation#eggshell chorion formation |
| GO:0007304 | 2 | 88 | 0.00229 | ovarian follicle cell development#chorion-containing eggshell formation |
| GO:0030703 | 2 | 89 | 0.00229 | eggshell formation |
| GO:0030707 | 2 | 184 | 0.00779 | ovarian follicle cell development |
| GO:0048646 | 2 | 235 | 0.0105 | anatomical structure development#anatomical structure morphogenesis#anatomical structure formation |
| GO:0048477 | 2 | 519 | 0.0385 | oogenesis |
| GO:0007292 | 2 | 528 | 0.0385 | female gamete generation |
| GO:0005549 | 1 | 54 | 0.0496 | odorant binding |

| Supplemental Table 7D. Overrepresented Gene Ontologies in Genes Found to Spike at Day 2 Post-Irradiation at Dose 5000 R. (Analysis with all data included.) | | | | |
| --- | --- | --- | --- | --- |
|  |  |  |  |  |
| (none) |  |  |  |  |

| Supplemental Table 7E. Overrepresented Gene Ontologies in Genes Found to Spike at Day 2 Post-Irradiation at Dose 5000 R. (Analysis with lowest dose discluded.) | | | | |
| --- | --- | --- | --- | --- |
|  |  |  |  |  |
| GO | Count | Total | P Value | GO Name |
| GO:0045434 | 1 | 7 | 0.00511 | negative regulation of female receptivity, post-mating |
| GO:0007621 | 1 | 8 | 0.00511 | negative regulation of female receptivity |
| GO:0048521 | 1 | 8 | 0.00511 | negative regulation of behavior |
| GO:0046008 | 1 | 10 | 0.00511 | regulation of female receptivity, post-mating |
| GO:0045924 | 1 | 16 | 0.00511 | regulation of female receptivity |
| GO:0060180 | 1 | 16 | 0.00511 | mating behavior#female mating behavior |
| GO:0060181 | 1 | 16 | 0.00511 | female receptivity |
| GO:0045297 | 1 | 18 | 0.00511 | post-mating behavior |
| GO:0050795 | 1 | 18 | 0.00511 | regulation of behavior |
| GO:0005179 | 1 | 47 | 0.012 | hormone activity |
| GO:0033057 | 1 | 69 | 0.0139 | reproductive behavior in a multicellular organism |
| GO:0032504 | 1 | 81 | 0.0139 | multicellular organism reproduction |
| GO:0048609 | 1 | 81 | 0.0139 | multicellular organism reproduction#reproductive process in a multicellular organism |
| GO:0007617 | 1 | 82 | 0.0139 | mating behavior |
| GO:0051705 | 1 | 82 | 0.0139 | behavioral interaction between organisms |
| GO:0019098 | 1 | 87 | 0.0139 | reproductive behavior |
| GO:0007618 | 1 | 94 | 0.0141 | mating |
| GO:0005102 | 1 | 136 | 0.0193 | receptor binding |
| GO:0022414 | 1 | 203 | 0.0271 | reproduction#reproductive process |
| GO:0051704 | 1 | 212 | 0.0271 | multi-organism process |

| Supplemental Table 7F. Overrepresented Gene Ontologies in Genes Found to Spike at Day 2 Post-Irradiation at Dose 10,000 R. (Analysis with lowest dose discluded.) | | | | |
| --- | --- | --- | --- | --- |
|  |  |  |  |  |
| GO | Count | Total | P Value | GO Name |
| GO:0007296 | 1 | 8 | 0.0268 | vitellogenesis |
| GO:0006123 | 1 | 14 | 0.0268 | organelle ATP synthesis coupled electron transport#mitochondrial electron transport, cytochrome c to oxygen |
| GO:0005751 | 1 | 17 | 0.0268 | mitochondrial inner membrane#mitochondrial respiratory chain#mitochondrial respiratory chain complex IV |
| GO:0045277 | 1 | 17 | 0.0268 | respiratory chain complex IV |
| GO:0004129 | 1 | 18 | 0.0268 | cytochrome-c oxidase activity |
| GO:0016675 | 1 | 18 | 0.0268 | oxidoreductase activity, acting on heme group of donors |
| GO:0016676 | 1 | 18 | 0.0268 | oxidoreductase activity, acting on heme group of donors, oxygen as acceptor |
| GO:0015002 | 1 | 18 | 0.0268 | heme-copper terminal oxidase activity |

| Supplemental Table 7G. Overrepresented Gene Ontologies in Genes Found to Spike at Day 10 Post-Irradiation at Dose 10,000 R. (Analysis with lowest dose discluded.) | | | | |
| --- | --- | --- | --- | --- |
|  |  |  |  |  |
| GO | Count | Total | P Value | GO Name |
| GO:0007296 | 4 | 8 | 4.14E-10 | vitellogenesis |
| GO:0007292 | 6 | 528 | 1.55E-06 | female gamete generation |
| GO:0003006 | 4 | 77 | 2.64E-06 | reproductive developmental process |
| GO:0007276 | 6 | 704 | 4.37E-06 | gamete generation |
| GO:0019953 | 6 | 730 | 4.37E-06 | sexual reproduction |
| GO:0007028 | 4 | 109 | 5.40E-06 | cytoplasm organization and biogenesis |
| GO:0022414 | 4 | 203 | 5.63E-05 | reproduction#reproductive process |
| GO:0007548 | 3 | 57 | 7.52E-05 | sex differentiation |
| GO:0005737 | 6 | 1833 | 0.000616 | cytoplasm |
| GO:0005811 | 3 | 129 | 0.000708 | lipid particle |
| GO:0048134 | 2 | 20 | 0.000944 | germ cell development#germ-line cyst formation |
| GO:0048477 | 4 | 519 | 0.00136 | oogenesis |
| GO:0016043 | 5 | 1962 | 0.0134 | cellular component organization and biogenesis |
| GO:0044424 | 6 | 3454 | 0.0134 | intracellular#intracellular part |
| GO:0005622 | 6 | 3465 | 0.0134 | intracellular |
| GO:0032502 | 5 | 2243 | 0.0217 | developmental process |
| GO:0008283 | 2 | 133 | 0.0222 | cell proliferation |
| GO:0007542 | 1 | 5 | 0.0252 | multicellular organismal development#germ-line sex determination#primary sex determination, germ-line |
| GO:0019099 | 1 | 5 | 0.0252 | female germ-line sex determination |
| GO:0016590 | 1 | 6 | 0.0278 | ACF complex |
| GO:0018992 | 1 | 6 | 0.0278 | multicellular organismal development#germ-line sex determination |
| GO:0007281 | 2 | 170 | 0.0291 | germ cell development |
| GO:0016709 | 1 | 7 | 0.0297 | oxidoreductase activity, acting on paired donors, with incorporation or reduction of molecular oxygen, NADH or NADPH as one donor, and incorporation of one atom of oxygen |
| GO:0008156 | 1 | 8 | 0.0297 | negative regulation of DNA replication |
| GO:0020037 | 1 | 8 | 0.0297 | heme binding |
| GO:0046906 | 1 | 8 | 0.0297 | tetrapyrrole binding |
| GO:0044464 | 6 | 4358 | 0.0297 | cell#cell part |
| GO:0005623 | 6 | 4358 | 0.0297 | cell |
| GO:0051053 | 1 | 9 | 0.0297 | negative regulation of DNA metabolic process |
| GO:0005678 | 1 | 9 | 0.0297 | chromatin assembly complex |
| GO:0006275 | 1 | 9 | 0.0297 | regulation of DNA replication |
| GO:0044444 | 4 | 1537 | 0.0297 | cytoplasm#cytoplasmic part |
| GO:0030237 | 1 | 11 | 0.0344 | multicellular organismal development#female sex determination |
| GO:0006378 | 1 | 12 | 0.0356 | mRNA polyadenylation |
| GO:0043631 | 1 | 12 | 0.0356 | RNA polyadenylation |
| GO:0045495 | 1 | 14 | 0.0405 | pole plasm |
| GO:0031124 | 1 | 16 | 0.0451 | mRNA 3'-end processing |
| GO:0007280 | 1 | 17 | 0.0457 | pole cell migration |
| GO:0031123 | 1 | 17 | 0.0457 | RNA 3'-end processing |
| GO:0007538 | 1 | 18 | 0.0472 | multicellular organismal development#primary sex determination |

| Supplemental Table 7H. Overrepresented Gene Ontologies in Genes Found to Spike at Day 20 Post-Irradiation at Dose 5000 R. (Analysis with lowest dose discluded.) | | | | |
| --- | --- | --- | --- | --- |
|  |  |  |  |  |
| (none) |  |  |  |  |
